# ICEs Are the Main Reservoirs of the Ciprofloxacin-Modifying *crpP* Gene in *Pseudomonas aeruginosa*

**DOI:** 10.3390/genes11080889

**Published:** 2020-08-04

**Authors:** João Botelho, Filipa Grosso, Luísa Peixe

**Affiliations:** 1 Antibiotic Resistance Evolution Group, Max-Planck-Institute for Evolutionary Biology, 24306 Plön, Germany; 2 Department of Evolutionary Ecology and Genetics, Zoological Institute, Christian-Albrechts-Universität zu Kiel, 24118 Kiel, Germany; 3 UCIBIO/REQUIMTE, Laboratório de Microbiologia, Faculdade de Farmácia, Universidade do Porto, 4050-313 Porto, Portugal; filipagrosso@gmail.com

**Keywords:** integrative and conjugative elements, *Pseudomonas aeruginosa*, ciprofloxacin resistance, *crpP*, pangenome

## Abstract

The ciprofloxacin-modifying *crpP* gene was recently identified in a plasmid isolated from a *Pseudomonas aeruginosa* clinical isolate. Homologues of this gene were also identified in *Escherichia coli*, *Klebsiella pneumoniae* and *Acinetobacter baumannii*. We set out to explore the mobile elements involved in the acquisition and spread of this gene in publicly available and complete genomes of *Pseudomonas* spp. All *Pseudomonas* complete genomes were downloaded from NCBI’s Refseq library and were inspected for the presence of the *crpP* gene. The mobile elements carrying this gene were further characterized. The *crpP* gene was identified only in *P. aeruginosa*, in more than half of the complete chromosomes (61.9%, *n* = 133/215) belonging to 52 sequence types, of which the high-risk clone ST111 was the most frequent. We identified 136 *crpP*-harboring integrative and conjugative elements (ICEs), with 93.4% belonging to the mating-pair formation G (MPF_G_) family. The ICEs were integrated at the end of a tRNA^Lys^ gene and were all flanked by highly conserved 45-bp direct repeats. The *crpP*-carrying ICEs contain 26 core genes (2.2% of all 1193 genes found in all the ICEs together), which are present in 99% or more of the *crpP*-harboring ICEs. The most frequently encoded traits on these ICEs include replication, transcription, intracellular trafficking and cell motility. Our work suggests that ICEs are the main vectors promoting the dissemination of the ciprofloxacin-modifying *crpP* gene in *P. aeruginosa*.

## 1. Introduction

*Pseudomonas aeruginosa* is a frequent cause of severe nosocomial infections and is one of the six ESKAPE pathogens (*Enterococcus faecium*, *Staphylococcus aureus*, *Klebsiella pneumoniae*, *Acinetobacter baumannii*, *P. aeruginosa*, and *Enterobacter* species) [1,2]. Ciprofloxacin is an antibiotic of the fluoroquinolone class that is active against *P. aeruginosa* infections [3,4]. In this species, a high proportion of clinical isolates are resistant to ciprofloxacin [5,6]. Commonly reported mechanisms of ciprofloxacin resistance include mutations in DNA gyrase and topoisomerase IV-encoding genes *gyrA*, *gyrB*, *parC* and *parE* and efflux pumps regulatory genes such as *nalC* and *nfxB* [6,7,8].

Besides chromosomal mutations, ciprofloxacin resistance can be mediated by horizontally transferred genes, such as the quinolone resistance *qnr* gene [9] and the ciprofloxacin-modifying *crpP* gene [10]. The *crpP* gene induces ATP-dependent phosphorylation of the antibiotic and was originally identified in a plasmid isolated from a *P. aeruginosa* clinical isolate [11]. This plasmid conferred resistance to ciprofloxacin when transferred to *P. aeruginosa* PAO1 strain. The authors also cloned the gene into a shuttle vector and the resulting recombinant plasmid conferred an increased minimum inhibitory concentration to the same antibiotic in *Escherichia coli*. Homologous *crpP* genes were also identified in *A. baumannii* [12] and in *E. coli* and *K. pneumoniae* clinical isolates from Mexican hospitals and conferred decreased susceptibility to ciprofloxacin [13,14].

Integrative and conjugative elements (ICEs) are mobile elements involved in vertical and horizontal transmission of antibiotic resistance genes (ARGs) and other beneficial genes in bacterial communities [2,15,16,17,18]. Like plasmids, these elements are transferred horizontally by conjugation and present variable sizes, from the small ~20 kb Tn*916*-family ICEs identified mostly in firmicutes to the large ~500 kb elements found in *Mesorhizobium* spp. (more examples can be browsed on the ICEberg database: https://db-mml.sjtu.edu.cn/ICEberg2/) [19]. Besides the conjugation apparatus, ICEs also carry modules responsible for the maintenance, excision and integration within a new host genome (the integrase mediates site-specific recombination between direct repeats located in the host and the ICE) [18]. ICEs can be classified into eight mating-pair formation (MPF) classes based on the classification of the type-IV secretion systems (T4SS) involved in conjugation [17]. Four MPF classes cover most proteobacterial species: MPF_G_, MPF_T_, MPF_F_ and MPF_I_. MPF_T_ is evenly distributed in plasmids and ICEs, while MPF_G_ is more prevalent in ICEs and MPF_F_ in plasmids. MPF_I_ is seldom identified [20]. Recently, Ruiz verified that several CrpP proteins were encoded next to genes typically present in ICEs [12]. Building on this observation, we set out to trace and characterize the *crpP*-harboring ICEs present in all complete *Pseudomonas* genomes available on NCBI.

## 2. Materials and Methods

### 2.1. Pangenome and Whole-Genome Analyses

All complete *Pseudomonas* genomes (*n* = 577 chromosomes and 163 plasmids) were downloaded from NCBI’s Refseq library on 01/02/2020 using ncbi-genome-download v0.2.11 (https://github.com/kblin/ncbi-genome-download) (Table S1). We used mlst v2.18.0 (https://github.com/tseemann/mlst) to scan the genomes against the PubMLST *Pseudomonas* typing schemes (https://pubmlst.org/) [21]. The genomes were annotated using prokka v1.14.5 (https://github.com/tseemann/prokka) [22]. We used the gff files created by Prokka to calculate the *P. aeruginosa* pangenome in roary v3.13.0 (https://github.com/sanger-pathogens/Roary) [23]. The core genome and *crpP*-harboring ICE core alignments created by Roary were used as input in fasttree v2.1.10 (http://www.microbesonline.org/fasttree/) [24] to create an approximately-maximum-likelihood phylogenetic tree using a generalized time-reversible (GTR) model of nucleotide evolution. We used the newick file created by FastTree to create a phylogenetic tree in iTOL (https://itol.embl.de/) [25]. We performed functional annotation based on orthology assignments (COGs) of protein files created by Prokka using eggNOG-mapper v2 (http://eggnog-mapper.embl.de/) [26].

### 2.2. Mining ICEs in Complete Genomes

We used the ICEfinder standalone version (http://202.120.12.136/ICEfinder/ICEfinder.html) [19] and manual curation to extract putative ICEs. We also used IslandViewer 4 (http://www.pathogenomics.sfu.ca/islandviewer/) [27] to browse pre-computed genomic islands. We searched for ARGs on extracted ICEs using amrfinder v3.6.7 and default parameters (50% minimum coverage of the reference protein and 90% minimum identity) (https://github.com/ncbi/amr/wiki/AMRFinder-database) [28]. Positive ICE hits for *crpP*-encoding genes were further characterized; we used fastANI v1.3 (https://github.com/ParBLiSS/FastANI) [29] to compute whole-genome average nucleotide identity (ANI) of non-*P. aeruginosa crpP*-positive hits, antismash online tool (https://antismash.secondarymetabolites.org/#!/start) [30] to look for secondary metabolite biosynthesis gene clusters, BAGEL (http://bagel4.molgenrug.nl/) [31] to trace bacteriocins, macsyfinder v1.0.5 (https://github.com/gem-pasteur/macsyfinder) [32,33] to assess the MPF family, MOBscan standalone version (https://castillo.dicom.unican.es/mobscan_about/) [34] to check relaxase (MOB) families and the CRISPRCasFinder online tool (https://crisprcas.i2bc.paris-saclay.fr/) [35] to detect clustered regularly interspaced short palindromic repeats and CRISPR-associated proteins (CRISPR-Cas). We used Roary to infer the total *crpP*-harboring ICE content. Variation in GC content between the ICE and the host’s chromosome was visualized in violin plots using ggplot2.

## 3. Results

### 3.1. The crpP Gene Is Widespread in P. aeruginosa

**Figure 1:**
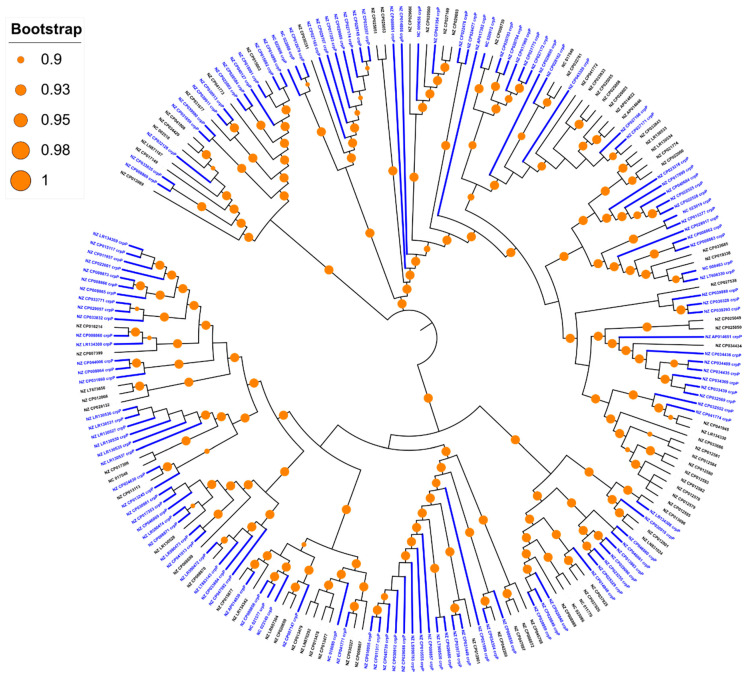
Approximately-maximum-likelihood phylogenetic tree base on the core genome alignment of 215 complete *P. aeruginosa* genomes. Branches and labels from *crpP*-positive hits are colored blue and the branches have twice the standard width. Branch lengths are ignored. Bootstrap values between 0.9 and 1 are represented by orange circles.

**Figure 2:**
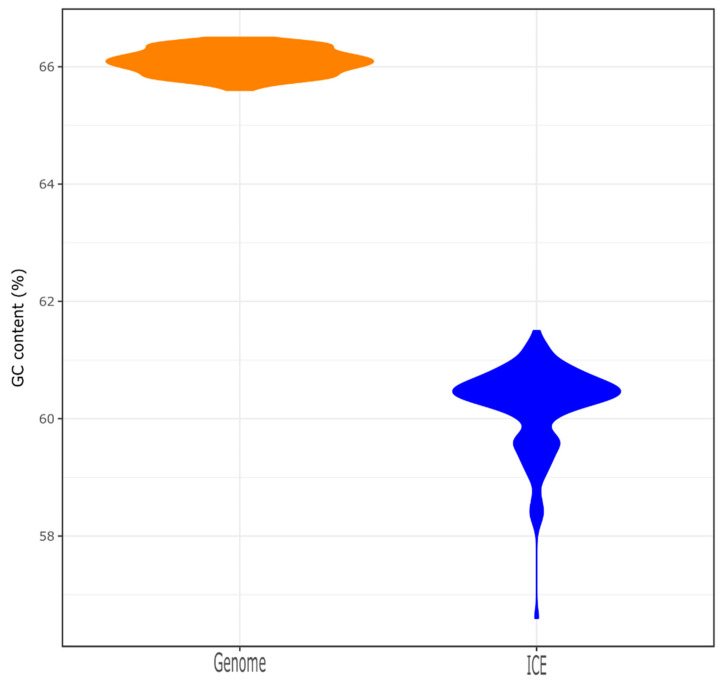
Distribution of the GC content of the chromosomes and integrative and conjugative elements (ICEs).

The CrpP-encoding gene was identified in 23.1% of the *Pseudomonas* spp. chromosomes (*n* = 133/577, including 131 *P. aeruginosa*, 1 *Pseudomonas fluorescens* and 1 *Pseudomonas* sp.) and 1 *P. aeruginosa* plasmid (accession number NZ_CP030914.1), which is different to the pUM505 plasmid reported by Chávez-Jacobo and colleagues [11]. These strains were collected from different sources, including the hospital setting and environmental samples (Table S2). However, we compared the ANI between non-*P. aeruginosa* hits (the *P. fluorescens* strain NCTC10783 and *Pseudomonas* sp. AK6U strains with accession numbers NZ_LR134300.1 and NZ_CP025229.1, respectively) and the *P. aeruginosa* DSM 50071 type strain (accession number NZ_CP012001.1) and we realized that these strains belong to the *P. aeruginosa* species, as the ANI value is above the 95% cutoff for species delineation (Table S3) [29]. All *crpP*-hits were only identified in *P. aeruginosa* complete genomes, so from here on, we focused our attention on *P. aeruginosa* genomes. Indeed, the percentage increases if we only consider the *P. aeruginosa* chromosomes (61.9%, *n* = 133/215), meaning that the *crpP* gene is present in more than half of the *P. aeruginosa* complete genomes (Figure 1). The GC content of *crpP*-harboring chromosomes varies from 65.6% to 66.5% (Figure 2) and the sequence length from 6.3 to 7.5 Mb. We identified several clones (*n* = 52 sequence types), of which the high-risk clone ST111 [36] was the most frequent (Table S4).

### 3.2. The Majority of crpP-Harboring ICEs belong to the MPF_G_ Family

A total of 316 putative ICEs (carrying the *crpP* gene or not) were identified among the 133 chromosomes. Each chromosome carries at least one ICE. Almost all *crpP* genes present in the chromosome were associated with an ICE. The only exception was observed for *P. aeruginosa* strains W60856 and B17932 (Table S1). The *crpP* was located within genomic islands for these two isolates. We found that 43.0% of the total putative ICEs (*n* = 136/316) harbor the ciprofloxacin-modifying gene. In *P. aeruginosa* RW109 strain (accession number NZ_LT969520.1, position 5629923-5820312 bp), we identified two *crpP*-harboring ICEs in tandem (Table S4). ICE size varied from 81.6 to 145.5 kb, and the GC content from 56.6% to 61.5% (Figure 2). The GC content of the *crpP* genes identified in these ICEs varies from 57.1% to 60.1%. We also found high GC values for the *crpP* homologous identified previously in *K. pneumoniae*, *E. coli* and *A. baumannii* (56.8%–64.7%) [12,13,14]. Most of the ICEs (*n* = 129/136) belong to the MPF_G_ family (Tables S4 and S5). For the remaining seven ICEs, no MPF family could be determined. All ICEs carried a MOB_H_ family relaxase.

### 3.3. The crpP-Carrying ICEs Integrate into a Specific Hotspot

All *crpP*-carrying ICEs identified in this study were integrated at the end of a tRNA^Lys^ gene that was found alone or in a tRNA cluster with tRNA^Pro^ and tRNA^Asn^. The only exception was observed in the two contiguous ICEs identified in *P. aeruginosa* RW109 strain, where the second ICE was integrated at the end of the first one. The tRNA^Lys^ genes shared the exact same sequence. All ICEs were flanked on both sides by 45-bp (5′-TGGTGGGTCGTGTAGGATTCGAACCTACGACCAATTGGTTAAAAG-3′) highly conserved direct repeats. The 136 integrases identified in this study, however, only shared 36.1% amino acid identity.

### 3.4. The P. aeruginosa Pangenome and the crpP-Carrying ICEs

**Figure 3:**
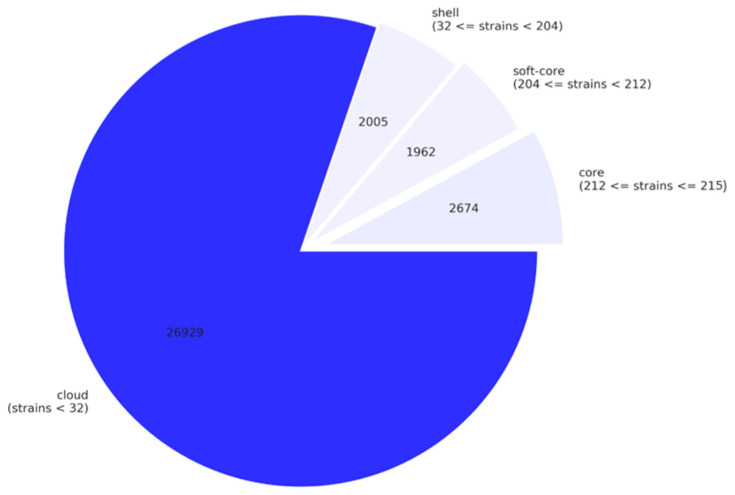
Pie chart of the breakdown of genes and the number of *P. aeruginosa* genomes in which they are present. Core genes are present in 99% or more of the genomes, soft core genes in 95% or more of the genomes and less than 99%, shell genes in between 15% and 95% and cloud genes in less than 15% of the genomes. This figure was created using the contributed Python script roary_plots.py in https://github.com/sanger-pathogens/Roary/blob/master/contrib/roary_plots/roary_plots.py.

The total number of genes among the 215 *P. aeruginosa* complete genomes (pangenome) is 33,570. The core genome (genes present in 99% or more of the genomes) contains 2674 genes (8.0% of all genes, Figure 3 and Figure S1). We also identified 11,241 unique genes, which are found in only one strain [37].

**Figure 4:**
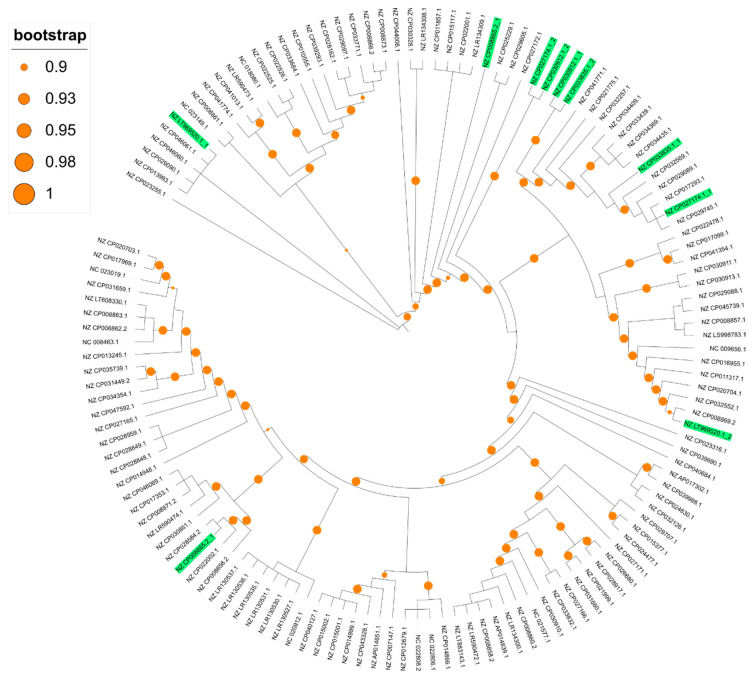
Approximately-maximum-likelihood phylogenetic tree base on the alignment of the 26 core genes identified in the *crpP*-harboring ICEs. Genome accession numbers containing more than one *crpP*-harboring ICE are highlighted in green. Branch lengths are ignored. Bootstrap values between 0.9 and 1 are represented by orange circles.

The total number of genes in the 136 *crpP*-harboring ICEs is 1193. We found 26 core genes (2.2% of all genes). The soft core content (genes present in 95% or more of the ICEs and less than 99%) includes 11 genes, 129 shell genes (between 15 and 95%) and 1027 cloud genes (less than 15%). We identified 451 unique genes among the *crpP*-harboring ICEs. Phylogenetic analysis of the *crpP*-harboring ICEs core alignment reveals that these ICEs are very diverse and the core genes only share 28.0% nucleotide identity (Figure 4).

### 3.5. The crpP-Carrying ICEs Encode Other Beneficial Genes

**Figure 5:**
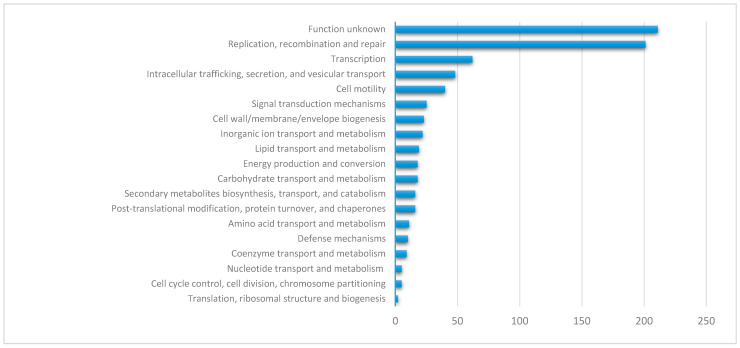
Functional annotation of proteins encoded by the *crpP*-harboring ICEs.

The most frequently encoded traits on *crpP*-harboring ICEs include replication, transcription, intracellular trafficking, and cell motility (Figure 5). However, nearly one-third of the proteins for which a COG category was attributed (30.7%, *n* = 211/687) encode for an unknown function (Table S6).

The *crpP* gene encoded for proteins ranging from 65 to 68 amino acids and is present in a single copy, with no flanking mobile units such as integrons, insertion sequences and/or transposons found on the close vicinity of the gene. The 136 CrpP proteins here identified share 19.7% amino acid identity. We also observed few co-resident ARGs within the *crpP*-harboring ICEs. Seven ICEs carry a type I-C CRISPR-Cas system and ten harbor pyocin S5-encoding genes (Table S4). The pyocins here identified are highly identical, sharing 95% amino acid identity. Besides pyocins, no secondary metabolites were identified within the extracted ICEs.

## 4. Discussion

Our results show that ICEs are the main drivers for the spread of the ciprofloxacin-modifying *crpP* gene in *P. aeruginosa*. The *crpP*-harboring ICEs are variable in composition, based on the small number of core genes and high number of cloud genes. A selective pressure exerted by the use of ciprofloxacin to treat *P. aeruginosa* infections may have promoted the dissemination of *crpP*-harboring ICEs among several clones and may have led to the emergence of previous minor *crpP*-harboring clones, with resistance as a key driver.

Curiously, and in opposition to several ARGs, no integrons, insertion sequences or transposons were found in the vicinity of the *crpP* gene. Pyocins produced by *P. aeruginosa* are important for niche competition. The pyocin S5 possesses bactericidal activity against clinical *P. aeruginosa* isolates [38,39]. The presence of these bacteriocins among some *crpP*-harboring ICEs can help to eliminate possible competitors and indirectly enhance ICE maintenance.

As expected for foreign DNA, the GC content of the ICEs (and that of the *crpP* gene) is lower than that from the chromosomes. Nevertheless, given the high GC content of these mobile elements (compared to the GC content of mobile elements from other species), and the biological barrier for gene acquisition between donor–recipient pairs having >5% difference in GC values [40], we suspect that the ancestor of the *crpP* gene derives from GC-rich taxa.

We identified highly conserved direct repeats that can be used to accurately delimit *crpP*-carrying ICEs in *P. aeruginosa*. Moreover, even though the *crpP* gene was first reported in a plasmid in *P. aeruginosa* [11], we found here a perfect match for the 45-bp direct repeat next to an integrase-encoding gene. We only found one match for the direct repeat in this plasmid, meaning that the ICE is degenerated and most likely depends on the plasmid to be mobilized. The same was observed for the *P. aeruginosa crpP*-carrying plasmid detected in this study (accession number NZ_CP030914.1).

The *crpP*-carrying ICEs were traced in several clones, taking advantage of the highly conserved attachment site in the tRNA^Lys^ gene. Interestingly, we found in a previous study that MPF_G_ class ICEs carrying specific ARGs were integrated at the end of tRNA^Gly^ genes [16]. In fact, this class of ICEs is frequently integrated at the end of a tRNA gene [41]. This behavior suggests that selection for the maintenance of these non-coding integration sites and seamless site-specific integration of ICEs in the chromosome will incur a lower burden to the recipient cell and therefore increase its fitness [18]. Consequently, conferring decreased susceptibility or low levels of ciprofloxacin resistance without incurring any fitness cost can help to explain the high frequency of *crpP*-carrying ICEs observed in this study. Future studies should explore the possible synergistic effects between commonly reported mutations leading to ciprofloxacin resistance and the presence of *crpP*-harboring ICEs.

As we were writing this manuscript, Ortiz de la Rosa et al. used a PCR approach to screen the presence of the *crpP* gene among a collection of clonally unrelated and multidrug-resistant *P. aeruginosa* isolates recovered from hospitals in France and Switzerland [42]. The gene was present in around half the isolates screened, which is in agreement with our findings. However, the authors used a different nomenclature to classify these elements: the PAGI-like pathogenicity islands are in fact ICEs. Examples of these elements can be found on Kung et al. review [43] and can be browsed on the ICEberg database (https://db-mml.sjtu.edu.cn/ICEberg2/) [19]. Additionally, the authors identified the 45-bp direct repeats we describe here flanking the ICEs and used a PCR approach to circularize these elements, confirming that these elements are indeed ICEs.

One of the caveats of our study is that we restricted the analyses to complete genomes. Given that the majority of the assemblies present in public databases such as NCBI correspond to draft genomes, we probably missed the identification of several *crpP*-carrying ICEs among *P. aeruginosa*. However, mobile elements such as ICEs tend to be fragmented in genomes sequenced with short-read technologies due to the presence of repetitive regions. Moreover, the identification of ICE fragments within the draft genome would require similar ICE sequences to use as a reference, which would then bias the study. Sequencing bacteria with long-read approaches is increasing, which will improve the accurate delimitation of an ever-growing number of mobile elements.

## 5. Conclusions

Our work demonstrates that ICEs are the main promoters involved in the dissemination of the ciprofloxacin-modifying *crpP* gene among clonally unrelated *P. aeruginosa* strains. We also identified highly conserved 45-bp direct repeats that can be used to accurately trace *crpP*-harboring ICEs on *P. aeruginosa* genomes. Future studies should explore the mobile elements involved in the acquisition of the *crpP* homologues identified in species other than *P. aeruginosa* and the possible synergistic effects between commonly reported mutations leading to ciprofloxacin resistance and the presence of *crpP*-carrying ICEs.

## Supplementary Materials

The following are available online at https://www.mdpi.com/2073-4425/11/8/889/s1, Figure S1: Graph with the frequency of genes versus the 215 *P. aeruginosa* complete genomes, Table S1: Accession numbers for the complete Pseudomonas genomes, Table S2: General features of the 134 *crpP*-harboring hits, Table S3: Average nucleotide identity (ANI) comparison between non-*Pseudomonas aeruginosa* strains identified in this study and the *P. aeruginosa* reference strain, Table S4: General features of the *crpP*-carrying ICEs, Table S5: Macsyfinder reports for the ICE proteomes, Table S6: Functional annotation of the ICE proteomes.

## Data Bibliography

The DNA sequences used in this study are available in NCBI, under the accession numbers provided in Supplementary Tables S1–S5.

## Data Summary

All the bacterial genomes scanned in this study have been deposited previously in the National Center for Biotechnology Information (NCBI) genome database and are listed in the Supplementary Materials. The newick files used to create the trees in Figure 1 and Figure 4 are deposited on figshare at https://figshare.com/projects/ICEs_are_the_main_reservoirs_of_the_ciprofloxacin-modifying_crpP_gene_in_Pseudomonas_aeruginosa/79308.
