# Supplementary material for: ICEs Are the Main Reservoirs of the Ciprofloxacin-Modifying crpP Gene in Pseudomonas aeruginosa
Source: Genes (Basel). 2020 Aug 4;11(8):889. doi: 10.3390/genes11080889 (PMC7463715; doi:10.3390/genes11080889)
Supplement: Supplementary file 1 [file genes-11-00889-s001.zip › Table_S2.docx]

**Table S2**. General features of the 134 crpP-harboring hits identified in this study.

| **Collection Date** | **Country** | **Culture collection** | **Host** | **Isolation Source** | **%GC** | **Sequence Length** | **Accession** |
| --- | --- | --- | --- | --- | --- | --- | --- |
| NA | NA | NA | Homo sapiens | infant with community-acquired diarrhea | 66.4 | 6421010 | NC_020912.1 |
| 05/12/1985 | Germany | NA | Homo sapiens sapiens | Cystic fibrosis lung | 66.1 | 6902967 | NZ_LT883143.1 |
| May-1997 | France | NA | Homo sapiens | Patient (nose) | 65.8 | 7055752 | NZ_CP013993.1 |
| NA | NA | NA | NA | NA | 66.3 | 6402658 | NC_018080.1 |
| 22/11/1989 | Japan | NA | Homo sapiens | NA | 66.2 | 6613260 | NZ_AP014839.1 |
| 2012 | Nepal: Kathmandu | NA | Homo sapiens | Urinary catheter | 66.0 | 6897018 | NZ_AP017302.1 |
| 2004-11 | NA | NA | Homo sapiens | Midstream urine | 65.9 | 7090694 | NZ_AP014651.1 |
| NA | NA | NA | NA | NA | 65.6 | 7207057 | NZ_LS998783.1 |
| jun/08 | USA: Honolulu | NA | NA | wastewater | 66.2 | 6723378 | NZ_CP017969.1 |
| NA | NA | NA | NA | NA | 66.3 | 6541482 | NZ_LT608330.1 |
| 16/09/2015 | Switzerland | NA | Homo sapiens | Hospital | 66.4 | 6451470 | NZ_LR130527.1 |
| 2015 | Switzerland | NA | Homo sapiens | Hospital | 66.4 | 6433962 | NZ_LR130530.1 |
| 16/09/2015 | Switzerland | NA | Homo sapiens | Hospital | 66.4 | 6452809 | NZ_LR130531.1 |
| 16/09/2015 | Switzerland | NA | Homo sapiens | Hospital | 66.4 | 6433960 | NZ_LR130536.1 |
| 16/09/2015 | Switzerland | NA | Homo sapiens | Hospital | 66.4 | 6434133 | NZ_LR130535.1 |
| 16/09/2015 | Switzerland | NA | Homo sapiens | Hospital | 66.4 | 6434020 | NZ_LR130537.1 |
| NA | NA | NA | NA | NA | 65.8 | 7049347 | NZ_LT969520.1 |
| NA | India | NA | NA | t-HCH contaminated soil | 66.2 | 6580038 | NC_023019.1 |
| NA | NA | NA | NA | NA | 66.4 | 6498072 | NC_022808.2 |
| NA | NA | NA | NA | NA | 66.3 | 6309305 | NC_022806.1 |
| NA | NA | NA | NA | NA | 66.4 | 6588339 | NC_009656.1 |
| NA | NA | NA | NA | NA | 66.5 | 6342034 | NC_021577.1 |
| NA | NA | NA | Homo sapiens | CF isolate | 66.3 | 6725183 | NC_023149.1 |
| 2013 | China | NA | Homo sapiens | medium | 66.2 | 6621378 | NZ_CP024477.1 |
| 10/mar/14 | Poland | NA | Homo sapiens | eye | 65.8 | 6902135 | NZ_CP035739.1 |
| 13/11/2018 | China | NA | Homo sapiens | sputum | 65.8 | 7344079 | NZ_CP046061.1 |
| 18/11/2018 | China | NA | Homo sapiens | sputum | 65.8 | 7343000 | NZ_CP046060.1 |
| 05/03/2015 | Colombia | NA | Homo sapiens | blood | 66.0 | 7097241 | NZ_CP029605.1 |
| 2016 | China: Beijing | NA | Homo sapiens | sputum specimen | 66.1 | 6809062 | NZ_CP041774.1 |
| 2015 | Ghana: Kumasi | NA | Homo sapiens | urine | 65.9 | 6925889 | NZ_CP031449.2 |
| 2014 | China: Guangdong | NA | Homo sapiens | sputum | 66.2 | 6616247 | NZ_CP041771.1 |
| 2010 | Costa Rica | NA | Homo sapiens | Sputum-lungs | 65.7 | 7190208 | NZ_CP045739.1 |
| NA | NA | NA | NA | NA | 66.0 | 6799785 | NZ_CP029745.1 |
| NA | NA | NA | NA | NA | 66.0 | 6946231 | NZ_CP032257.1 |
| NA | NA | NA | NA | NA | 65.9 | 7012922 | NZ_CP027174.1 |
| NA | NA | NA | NA | NA | 65.7 | 7240677 | NZ_CP027172.1 |
| NA | NA | NA | NA | NA | 66.1 | 6747010 | NZ_CP027171.1 |
| NA | NA | NA | NA | NA | 65.8 | 7162784 | NZ_CP027166.1 |
| NA | NA | NA | NA | NA | 66.4 | 6463575 | NZ_CP027165.1 |
| NA | NA | NA | NA | NA | 65.9 | 6540996 | NZ_CP030328.1 |
| NA | NA | NA | NA | NA | 65.9 | 7139518 | NZ_CP029097.1 |
| NA | NA | NA | NA | NA | 65.8 | 7267567 | NZ_CP029090.1 |
| NA | NA | NA | NA | NA | 66.1 | 6853499 | NZ_CP029089.1 |
| NA | NA | NA | NA | NA | 65.8 | 7125975 | NZ_CP029088.1 |
| NA | Hong Kong | ATCC:27853 | Homo sapiens | sputum | 66.1 | 6833187 | NZ_CP011857.1 |
| 01-May-2014 | Netherlands | ATCC:27853 | Homo sapiens | NA | 66.1 | 6827737 | NZ_CP015117.1 |
| 2012 | Germany: Heidelberg | NA | NA | urinary tract infection | 66.5 | 6527298 | NZ_CP041354.1 |
| 04-May-2014 | India | NA | Homo sapiens | blood | 65.9 | 6759594 | NZ_CP034435.1 |
| 16-May-2014 | India | NA | Homo sapiens | blood | 65.9 | 6744658 | NZ_CP034436.1 |
| 16/mar/17 | India | NA | Homo sapiens | blood | 65.8 | 6876113 | NZ_CP032569.1 |
| NA | NA | NA | Homo sapiens | combat injury wound | 66.0 | 7021552 | NZ_CP015377.1 |
| 22/09/2017 | China | NA | Homo sapiens | NA | 66.1 | 6756154 | NZ_CP040684.1 |
| 16/04/2012 | Netherlands:Rotterdam | NA | NA | Microbial feature | 65.6 | 7497593 | NZ_CP011317.1 |
| 2001-11 | Sweden:Solna | CCUG:51971 | Homo sapiens | Urine sample | 66.1 | 7012798 | NZ_CP043328.1 |
| 08/01/2013 | Sweden: Gothenburg | CCUG:70744 | Homo sapiens | sputum | 66.0 | 6859232 | NZ_CP023255.1 |
| 2013 | China | NA | NA | soil | 66.3 | 6641902 | NZ_CP017099.1 |
| 2010 | USA | NA | Homo sapiens | oropharynx, sputum, or bronchoalveolar lavage | 66.2 | 6813135 | NZ_CP044006.1 |
| 2012 | USA: New York, NY | NA | Homo sapiens | NA | 66.2 | 6517340 | NZ_CP008856.2 |
| 2012 | USA: New York, NY | NA | Homo sapiens | NA | 65.8 | 7273258 | NZ_CP008857.1 |
| 2012 | USA: New York | NA | Homo sapiens | urine | 65.9 | 6645227 | NZ_CP026680.1 |
| 2012 | USA: New York, NY | NA | Homo sapiens | NA | 66.3 | 6618768 | NZ_CP008858.2 |
| 2012 | USA: New York, NY | NA | Homo sapiens | NA | 66.1 | 6794354 | NZ_CP008873.1 |
| 01/01/2015 | China | NA | NA | wastewater | 66.2 | 6866790 | NZ_CP017353.1 |
| 18-Dec-2015 | NA | FDA:FDAARGOS_505 | Homo sapiens | Endotracheal aspirate | 65.9 | 7029824 | NZ_CP033832.1 |
| 06-Apr-2016 | NA | FDA:FDAARGOS_532 | Homo sapiens | Throat (pharynx) | 66.1 | 6928914 | NZ_CP033771.1 |
| NA | NA | FDA:FDAARGOS_570 | Homo sapiens | UCC isolate | 65.9 | 7119105 | NZ_CP033835.1 |
| NA | NA | FDA:FDAARGOS_610 | ZymoBIOMICS Microbial Community Standard Strain | Water bottle, Animal room | 66.2 | 6792215 | NZ_CP041013.1 |
| 1979 | USA: Oregon | NA | Homo sapiens | the sputum of a cystic fibrosis patient | 66.1 | 6712339 | NZ_CP010555.1 |
| 06/04/2010 | Switzerland | NA | Homo sapiens | skin | 66.1 | 7079598 | NZ_CP033684.1 |
| 2012 | USA: New York, NY | NA | Homo sapiens | NA | 66.3 | 6568228 | NZ_CP008860.2 |
| 2012 | USA: New York, NY | NA | Homo sapiens | NA | 66.1 | 6836415 | NZ_CP008861.1 |
| NA | China: Shanghai | NA | NA | NA | 66.2 | 6876988 | NZ_CP030861.1 |
| 2011 | Belgium | NA | Homo sapiens | urine | 65.8 | 7047704 | NZ_CP034354.1 |
| 20-Apr-2012 | China:Daqing | NA | NA | crude oil | 66.4 | 6486336 | NZ_CP028959.1 |
| 20-Apr-2012 | China:Daqing | NA | NA | crude oil | 66.4 | 6501414 | NZ_CP028848.1 |
| 20-Apr-2012 | China:Daqing | NA | NA | crude oil | 66.4 | 6481697 | NZ_CP028849.1 |
| 18/05/1998 | Mexico | NA | Homo sapiens | cystic fibrosis pediatric isolate | 66.4 | 6335031 | NZ_CP047592.1 |
| 05-Feb-2018 | USA: Madison, WI | NA | NA | soil | 66.0 | 6867314 | NZ_CP028917.1 |
| NA | NA | NA | NA | NA | 65.9 | 7038012 | NZ_CP029707.1 |
| NA | Belgium: Primeur, Waregem | LMG:23160 | NA | methanogenic sludge from a potato processing plant | 66.3 | 6737396 | NZ_CP046069.1 |
| 01/10/2012 | China: Beijing | NA | Homo sapiens | sputum | 66.0 | 6824837 | NZ_CP022478.1 |
| 2012 | USA: New York, NY | NA | Homo sapiens | NA | 66.0 | 6460023 | NZ_CP008862.2 |
| 2012 | USA: New York, NY | NA | Homo sapiens | NA | 66.0 | 6897231 | NZ_CP008863.1 |
| 13/10/2012 | USA:Texas | NA | Homo sapiens | Sacrum | 66.0 | 7050928 | NZ_CP028162.1 |
| 15/jun/13 | China: Haidian, Beijing | CGMCC:8511 | NA | soil | 66.4 | 6370730 | NZ_CP014948.1 |
| 1957 | NA | NCTC:11445 | NA | not available: to be reported later | 66.1 | 6766292 | NZ_LR134308.1 |
| 1969 | NA | NCTC:12903 | NA | Blood culture | 66.1 | 6839985 | NZ_LR134309.1 |
| NA | NA | NA | NA | Water bottle | 66.2 | 6791490 | NZ_LR590473.1 |
| 1900/2015 | NA | NCTC:13618 | Homo sapiens | not available: not collected | 66.2 | 6788588 | NZ_LR590474.1 |
| 1900/2015 | NA | NCTC:13620 | not available: not collected | not available: not collected | 66.2 | 6779267 | NZ_LR590472.1 |
| 01/jun/03 | Pacific Ocean | NA | NA | Open Ocean | 66.0 | 6952237 | NZ_CP022526.1 |
| 01/jun/03 | Pacific Ocean | NA | NA | Open Ocean | 66.0 | 6943220 | NZ_CP022525.1 |
| NA | Hong Kong | NA | Homo sapiens | NA | 66.4 | 6445239 | NZ_CP014866.1 |
| 1997 | Brazil: Sao Paulo, SP | NA | Homo sapiens | urine | 66.1 | 6721480 | NZ_CP015001.1 |
| 27/jun/12 | Mexico: Mexico city | NA | Homo sapiens | Blood | 65.7 | 7411863 | NZ_CP022001.1 |
| 02-May-2014 | Mexico: Mexico city | NA | Homo sapiens | Blood | 65.8 | 7050510 | NZ_CP022002.1 |
| 15-May-2014 | China: Chongqing | NA | NA | hospital sewage | 66.3 | 6500439 | NZ_CP012679.1 |
| 23/05/2018 | China | NA | Homo sapiens | feces | 66.2 | 6539894 | NZ_CP040127.1 |
| 1997 | India | NA | Homo sapiens | eye | 66.1 | 6810079 | NZ_CP032552.1 |
| 25-Oct-2005 | Mexico: Mexico city | NA | Homo sapiens | bronchial washing | 65.8 | 7241575 | NZ_CP021775.1 |
| 2010 | China: WeiHai,Shandong; Mink raising farm | NA | Mink | NA | 66.1 | 6926363 | NZ_CP024630.1 |
| 2006 | Brazil: Sao Paulo, SP | NA | Homo sapiens | tracheal aspirate | 66.0 | 7018690 | NZ_CP014999.1 |
| 2007 | Brazil: Sao Paulo, SP | NA | Homo sapiens | tracheal aspirate | 66.0 | 6928736 | NZ_CP015002.1 |
| Dec-2013 | Germany | NA | Homo sapiens | NA | 66.1 | 6816227 | NZ_CP017293.1 |
| 20/jul/05 | Mexico: Mexico city | NA | Homo sapiens | bronchial washing | 66.2 | 6566724 | NZ_CP021999.1 |
| Oct-1999 | USA: Chicago, Illinois | NA | Homo sapiens | blood | 66.3 | 6546467 | NZ_CP031659.1 |
| jan/00 | USA: Chicago, Illinois | NA | Homo sapiens | blood | 66.3 | 6503460 | NZ_CP031660.1 |
| May-2001 | USA: Chicago, Illinois | NA | Homo sapiens | blood | 66.0 | 6879622 | NZ_CP039293.1 |
| 03/11/2017 | Poland:Warsaw | NA | NA | wound | 66.4 | 6383803 | NZ_CP032126.1 |
| 02/05/2015 | Singapore | NA | Homo sapiens | sputum | 66.1 | 6893164 | NZ_CP020703.1 |
| 11/04/2015 | Singapore | NA | Homo sapiens | sputum | 66.0 | 6985102 | NZ_CP020704.1 |
| NA | Canada: Montreal | NA | NA | Dental unit waterline | 65.9 | 6930893 | NZ_CP023316.1 |
| NA | Netherlands: Bilthoven | NA | Homo sapiens | NA | 65.7 | 7380063 | NZ_CP016955.1 |
| 2012 | USA: New York, NY | NA | Homo sapiens | NA | 66.0 | 6934277 | NZ_CP008865.2 |
| 18-Sep-2016 | India | NA | Homo sapiens | sputum | 65.8 | 6937609 | NZ_CP034369.1 |
| 27-Sep-2016 | India | NA | Homo sapiens | sputum | 65.8 | 7005215 | NZ_CP034409.1 |
| 2016 | India | NA | Homo sapiens | sputum | 65.8 | 6877287 | NZ_CP033439.1 |
| 2013 | Thailand: Bangkok | NA | Homo sapiens | adult male sputum | 66.2 | 6573638 | NZ_CP039990.1 |
| 2013 | Thailand: Bangkok | NA | Homo sapiens | adult male sputum | 66.1 | 6782092 | NZ_CP039988.1 |
| 2012 | USA: New York, NY | NA | Homo sapiens | NA | 66.1 | 6795741 | NZ_CP008866.2 |
| NA | NA | NA | Homo sapiens | Skin wound of burn human patient | 66.4 | 6400418 | NZ_CP013245.1 |
| 2012 | USA: New York, NY | NA | Homo sapiens | NA | 65.9 | 6808844 | NZ_CP008869.2 |
| 2012 | USA: New York, NY | NA | Homo sapiens | NA | 66.2 | 6777566 | NZ_CP008871.2 |
| 2012 | USA: New York, NY | NA | Homo sapiens | NA | 66.2 | 6896700 | NZ_CP008864.2 |
| 2017-07 | China: Chengdu, Sichuan | NA | Homo sapiens | NA | 66.0 | 6886080 | NZ_CP028584.2 |
| 2009 | South Korea: Seoul | NA | Homo sapiens | sputum | 66.2 | 6831076 | NZ_CP030910.1 |
| 2009 | South Korea: Seoul | NA | Homo sapiens | sputum | 66.0 | 6940949 | NZ_CP030911.1 |
| 2009 | South Korea: Seoul | NA | Homo sapiens | sputum | 65.8 | 7106857 | NZ_CP030912.1 |
| 2009 | South Korea: Seoul | NA | Homo sapiens | sputum | 66.1 | 6868832 | NZ_CP030913.1 |
| 2009 | South Korea: Seoul | NA | Homo sapiens | sputum | 60.1 | 85842 | NZ_CP030914.1 |
| NA | NA | NA | NA | NA | 66.3 | 6537648 | NC_008463.1 |
| NA | Malaysia | NA | NA | compost | 66.4 | 6433441 | NZ_CP007147.1 |
| 1900/1971 | NA | NCTC:10783 | Homo sapiens | Respiratory Tract | 65.9 | 6866429 | NZ_LR134300.1 |
| 07/11/2012 | Kuwait: Fahaheel | NA | NA | Oil contaminated soil | 65.8 | 6909530 | NZ_CP025229.1 |

NA stands for no information available.
