# Supplementary material for: ICEs Are the Main Reservoirs of the Ciprofloxacin-Modifying crpP Gene in Pseudomonas aeruginosa
Source: Genes (Basel). 2020 Aug 4;11(8):889. doi: 10.3390/genes11080889 (PMC7463715; doi:10.3390/genes11080889)
Supplement: Supplementary file 1 [file genes-11-00889-s001.zip › Table_S1.docx]

**Table S1**. Complete Pseudomonas genomes analyzed in this study.

| **Name** | **Description** | **Sequence Length** | **%GC** |
| --- | --- | --- | --- |
| NC_002516.2 | Pseudomonas aeruginosa PAO1, complete genome | 6264404 | 66.6 |
| NC_002947.4 | Pseudomonas putida KT2440 chromosome, complete genome | 6181873 | 61.5 |
| NC_004129.6 | Pseudomonas protegens Pf-5, complete genome | 7074893 | 63.3 |
| NC_004578.1 | [Pseudomonas syringae] pv. tomato str. DC3000 chromosome, complete genome | 6397126 | 58.4 |
| NC_004632.1 | [Pseudomonas syringae] pv. tomato str. DC3000 plasmid pDC3000B, complete sequence | 67473 | 56.2 |
| NC_004633.1 | [Pseudomonas syringae] pv. tomato str. DC3000 plasmid pDC3000A, complete sequence | 73661 | 55.1 |
| NC_005773.3 | Pseudomonas savastanoi pv. phaseolicola 1448A, complete genome | 5928787 | 58.0 |
| NC_007005.1 | Pseudomonas syringae pv. syringae B728a chromosome, complete genome | 6093698 | 59.2 |
| NC_007274.1 | Pseudomonas savastanoi pv. phaseolicola 1448A large plasmid, complete sequence | 131950 | 54.1 |
| NC_007275.1 | Pseudomonas savastanoi pv. phaseolicola 1448A small plasmid, complete sequence | 51711 | 56.0 |
| NC_007492.2 | Pseudomonas fluorescens Pf0-1, complete genome | 6438405 | 60.5 |
| NC_008027.1 | Pseudomonas entomophila L48, complete sequence | 5888780 | 64.2 |
| NC_008463.1 | Pseudomonas aeruginosa UCBPP-PA14, complete genome | 6537648 | 66.3 |
| NC_009434.1 | Pseudomonas stutzeri A1501, complete genome | 4567418 | 63.9 |
| NC_009439.1 | Pseudomonas mendocina ymp, complete genome | 5072807 | 64.7 |
| NC_009512.1 | Pseudomonas putida F1, complete genome | 5959964 | 61.9 |
| NC_009656.1 | Pseudomonas aeruginosa PA7, complete genome | 6588339 | 66.4 |
| NC_010322.1 | Pseudomonas putida GB-1, complete sequence | 6078430 | 61.9 |
| NC_010501.1 | Pseudomonas putida W619, complete sequence | 5774330 | 61.4 |
| NC_011770.1 | Pseudomonas aeruginosa LESB58 complete genome sequence | 6601757 | 66.3 |
| NC_012660.1 | Pseudomonas fluorescens SBW25 complete genome | 6722539 | 60.5 |
| NC_015379.1 | Pseudomonas brassicacearum subsp. brassicacearum NFM421, complete genome | 6843248 | 60.8 |
| NC_015410.1 | Pseudomonas mendocina NK-01, complete genome | 5434353 | 62.5 |
| NC_015556.1 | Pseudomonas fulva 12-X, complete genome | 4920769 | 63.5 |
| NC_015733.1 | Pseudomonas putida S16, complete genome | 5984790 | 62.3 |
| NC_015740.1 | Pseudomonas stutzeri strain CGMCC 1.1803, complete genome | 4547930 | 63.9 |
| NC_016830.1 | Pseudomonas fluorescens F113, complete genome | 6845832 | 60.8 |
| NC_017530.1 | Pseudomonas putida BIRD-1, complete genome | 5731541 | 61.7 |
| NC_017532.1 | Pseudomonas stutzeri DSM 4166, complete genome | 4689946 | 64.0 |
| NC_017548.1 | Pseudomonas aeruginosa M18, complete genome | 6327754 | 66.5 |
| NC_017549.1 | Pseudomonas aeruginosa NCGM2.S1 DNA, complete genome | 6764661 | 66.1 |
| NC_017911.1 | Pseudomonas fluorescens A506, complete genome | 5962570 | 60.0 |
| NC_017986.1 | Pseudomonas putida ND6, complete genome | 6085449 | 61.8 |
| NC_018028.1 | Pseudomonas stutzeri CCUG 29243, complete genome | 4709064 | 62.7 |
| NC_018080.1 | Pseudomonas aeruginosa DK2, complete genome | 6402658 | 66.3 |
| NC_018177.1 | Pseudomonas stutzeri DSM 10701, complete genome | 4174118 | 63.2 |
| NC_018220.1 | Pseudomonas putida DOT-T1E, complete genome | 6260702 | 61.4 |
| NC_018746.1 | Pseudomonas putida ND6 plasmid pND6-2, complete sequence | 117003 | 57.8 |
| NC_019670.1 | Pseudomonas sp. UW4, complete genome | 6183388 | 60.1 |
| NC_019905.1 | Pseudomonas putida HB3267, complete genome | 5875750 | 62.6 |
| NC_019906.1 | Pseudomonas putida HB3267 plasmid pPC9, complete sequence | 80360 | 60.0 |
| NC_019936.1 | Pseudomonas stutzeri RCH2, complete genome | 4575057 | 62.5 |
| NC_019937.1 | Pseudomonas stutzeri RCH2 plasmid pPSEST01, complete sequence | 12763 | 53.9 |
| NC_019938.1 | Pseudomonas stutzeri RCH2 plasmid pPSEST02, complete sequence | 9865 | 60.3 |
| NC_019939.1 | Pseudomonas stutzeri RCH2 plasmid pPSEST03, complete sequence | 2804 | 61.8 |
| NC_020209.1 | Pseudomonas poae RE*1-1-14, complete genome | 5512241 | 60.8 |
| NC_020829.1 | Pseudomonas sp. ATCC 13867, complete sequence | 5696307 | 65.2 |
| NC_020912.1 | Pseudomonas aeruginosa B136-33, complete genome | 6421010 | 66.4 |
| NC_021237.1 | Pseudomonas protegens CHA0, complete genome | 6867980 | 63.4 |
| NC_021361.1 | Pseudomonas fluorescens A506 plasmid pA506, complete sequence | 56977 | 51.9 |
| NC_021491.1 | Pseudomonas putida H8234, complete genome | 6870827 | 61.6 |
| NC_021499.1 | Pseudomonas resinovorans NBRC 106553 DNA, complete geonome | 6285863 | 65.6 |
| NC_021505.1 | Pseudomonas putida NBRC 14164 DNA, complete genome | 6156701 | 62.3 |
| NC_021506.1 | Pseudomonas resinovorans NBRC 106553 plasmid pCAR1.3 DNA, complete genome | 198965 | 56.4 |
| NC_021577.1 | Pseudomonas aeruginosa RP73, complete genome | 6342034 | 66.5 |
| NC_022738.1 | Pseudomonas sp. VLB120, complete genome | 5644569 | 61.8 |
| NC_022739.1 | Pseudomonas sp. VLB120 plasmid pSTY, complete sequence | 321653 | 56.8 |
| NC_022806.1 | Pseudomonas aeruginosa PA1R, complete genome | 6309305 | 66.3 |
| NC_022808.2 | Pseudomonas aeruginosa PA1, complete genome | 6498072 | 66.4 |
| NC_023019.1 | Pseudomonas aeruginosa MTB-1, complete genome | 6580038 | 66.2 |
| NC_023064.1 | Pseudomonas sp. TKP, complete genome | 7012672 | 60.5 |
| NC_023066.1 | Pseudomonas aeruginosa LES431, complete genome | 6550070 | 66.3 |
| NC_023075.1 | Pseudomonas monteilii SB3078, complete genome | 6000087 | 62.5 |
| NC_023076.1 | Pseudomonas monteilii SB3101, complete genome | 5945120 | 62.5 |
| NC_023149.1 | Pseudomonas aeruginosa SCV20265, complete genome | 6725183 | 66.3 |
| NZ_AP014522.1 | Pseudomonas protegens Cab57 DNA, complete genome | 6827892 | 63.3 |
| NZ_AP014622.1 | Pseudomonas aeruginosa DNA, complete genome, strain: NCGM 1900 | 6814936 | 66.0 |
| NZ_AP014623.1 | Pseudomonas chlororaphis subsp. aurantiaca DNA, complete genome, strain: StFRB508 | 6997933 | 62.8 |
| NZ_AP014627.1 | Pseudomonas sp. Os17 DNA, complete genome | 6885464 | 63.5 |
| NZ_AP014628.1 | Pseudomonas sp. St29 DNA, complete genome | 6833117 | 63.3 |
| NZ_AP014637.1 | Pseudomonas sp. StFLB209 DNA, complete genome | 6332373 | 60.7 |
| NZ_AP014646.1 | Pseudomonas aeruginosa DNA, complete genome, strain: NCGM 1984 | 6850954 | 66.0 |
| NZ_AP014651.1 | Pseudomonas aeruginosa DNA, complete genome, strain: NCGM257 | 7090694 | 65.9 |
| NZ_AP014839.1 | Pseudomonas aeruginosa DNA, complete genome, strain: 8380 | 6613260 | 66.2 |
| NZ_AP014862.1 | Pseudomonas pseudoalcaligenes KF707 = NBRC 110670 DNA, complete genome | 6183134 | 65.5 |
| NZ_AP014863.1 | Pseudomonas pseudoalcaligenes KF707 = NBRC 110670 plasmid pKF707 DNA, complete genome | 59815 | 60.5 |
| NZ_AP015029.1 | Pseudomonas putida DNA, complete genome, strain: KF715 | 6583377 | 61.9 |
| NZ_AP015030.1 | Pseudomonas putida plasmid pKF715A DNA, complete genome, strain: KF715 | 483376 | 57.4 |
| NZ_AP015031.1 | Pseudomonas putida plasmid pKF715B DNA, complete genome, strain: KF715 | 276165 | 56.2 |
| NZ_AP015032.1 | Pseudomonas putida plasmid pKF715C DNA, complete genome, strain: KF715 | 94696 | 62.4 |
| NZ_AP015033.1 | Pseudomonas putida plasmid pKF715D DNA, complete genome, strain: KF715 | 30071 | 54.0 |
| NZ_AP017302.1 | Pseudomonas aeruginosa DNA, complete genome, strain: IOMTU 133 | 6897018 | 66.0 |
| NZ_AP020337.1 | Pseudomonas sp. KUIN-1 DNA, complete genome | 6028589 | 59.2 |
| NZ_AY208917.1 | Pseudomonas putida ND6 plasmid pND6-1, complete sequence | 101858 | 57.0 |
| NZ_CP005960.1 | Pseudomonas mandelii JR-1, complete genome | 6778052 | 59.2 |
| NZ_CP005961.1 | Pseudomonas mandelii JR-1 plasmid, complete sequence | 410512 | 55.1 |
| NZ_CP005969.1 | Pseudomonas syringae pv. syringae B301D chromosome, complete genome | 6094819 | 59.2 |
| NZ_CP005970.1 | Pseudomonas syringae UMAF0158, complete genome | 5787986 | 59.3 |
| NZ_CP005971.1 | Pseudomonas syringae UMAF0158 plasmid, complete sequence | 63004 | 54.6 |
| NZ_CP005975.1 | Pseudomonas simiae strain PICF7, complete genome | 6136735 | 60.4 |
| NZ_CP006256.1 | Pseudomonas syringae pv. syringae HS191, complete genome | 5950211 | 59.0 |
| NZ_CP006257.1 | Pseudomonas syringae pv. syringae HS191 plasmid, complete sequence | 52548 | 54.4 |
| NZ_CP007012.1 | Pseudomonas sp. FGI182, complete genome | 5891312 | 63.3 |
| NZ_CP007014.1 | Pseudomonas syringae CC1557, complete sequence | 5758024 | 58.6 |
| NZ_CP007015.1 | Pseudomonas syringae CC1557 plasmid pCC1557, complete sequence | 53629 | 54.1 |
| NZ_CP007039.1 | Pseudomonas cichorii JBC1, complete genome | 5986012 | 58.1 |
| NZ_CP007147.1 | Pseudomonas aeruginosa YL84, complete genome | 6433441 | 66.4 |
| NZ_CP007399.1 | Pseudomonas aeruginosa strain F22031, complete genome | 6603099 | 66.2 |
| NZ_CP007410.1 | Pseudomonas brassicacearum strain DF41, complete genome | 6652396 | 60.5 |
| NZ_CP007441.1 | Pseudomonas stutzeri strain 28a24, complete genome | 4731359 | 60.6 |
| NZ_CP007509.1 | Pseudomonas stutzeri strain 19SMN4, complete genome | 4725662 | 62.3 |
| NZ_CP007510.1 | Pseudomonas stutzeri strain 19SMN4 plasmid pLIB119, complete plasmid | 107733 | 58.1 |
| NZ_CP007511.1 | Pseudomonas balearica DSM 6083 strain DSM6083 (=SP1402) chromosome, complete genome | 4383480 | 64.7 |
| NZ_CP007620.1 | Pseudomonas putida strain DLL-E4, complete genome | 6484062 | 62.5 |
| NZ_CP008696.1 | Pseudomonas chlororaphis strain PA23, complete genome | 7122173 | 62.6 |
| NZ_CP008739.1 | Pseudomonas aeruginosa VRFPA04, complete genome | 6818030 | 66.5 |
| NZ_CP008742.1 | Pseudomonas savastanoi pv. savastanoi NCPPB 3335, complete genome | 6016828 | 58.1 |
| NZ_CP008856.2 | Pseudomonas aeruginosa strain F23197, complete genome | 6517340 | 66.2 |
| NZ_CP008857.1 | Pseudomonas aeruginosa strain F30658, complete genome | 7273258 | 65.8 |
| NZ_CP008858.2 | Pseudomonas aeruginosa strain F63912, complete genome | 6618768 | 66.3 |
| NZ_CP008859.2 | Pseudomonas aeruginosa strain H5708, complete genome | 6334378 | 66.5 |
| NZ_CP008860.2 | Pseudomonas aeruginosa strain H27930, complete genome | 6568228 | 66.3 |
| NZ_CP008861.1 | Pseudomonas aeruginosa strain H47921, complete genome | 6836415 | 66.1 |
| NZ_CP008862.2 | Pseudomonas aeruginosa strain M1608, complete genome | 6460023 | 66.0 |
| NZ_CP008863.1 | Pseudomonas aeruginosa strain M37351, complete genome | 6897231 | 66.0 |
| NZ_CP008864.2 | Pseudomonas aeruginosa strain W60856, complete genome | 6896700 | 66.2 |
| NZ_CP008865.2 | Pseudomonas aeruginosa strain S86968, complete genome | 6934277 | 66.0 |
| NZ_CP008866.2 | Pseudomonas aeruginosa strain T38079, complete genome | 6795741 | 66.1 |
| NZ_CP008867.1 | Pseudomonas aeruginosa strain T52373, complete genome | 6322459 | 66.5 |
| NZ_CP008868.1 | Pseudomonas aeruginosa strain T63266, complete genome | 6456866 | 66.3 |
| NZ_CP008869.2 | Pseudomonas aeruginosa strain W16407, complete genome | 6808844 | 65.9 |
| NZ_CP008870.2 | Pseudomonas aeruginosa strain W36662, complete genome | 6791731 | 66.2 |
| NZ_CP008871.2 | Pseudomonas aeruginosa strain W45909, complete genome | 6777566 | 66.2 |
| NZ_CP008872.2 | Pseudomonas aeruginosa strain X78812, complete genome | 6348761 | 66.4 |
| NZ_CP008873.1 | Pseudomonas aeruginosa strain F9670, complete genome | 6794354 | 66.1 |
| NZ_CP008896.1 | Pseudomonas fluorescens strain UK4, complete genome | 6064456 | 60.1 |
| NZ_CP009048.1 | Pseudomonas alkylphenolica strain KL28 chromosome, complete genome | 5764622 | 60.6 |
| NZ_CP009290.1 | Pseudomonas chlororaphis subsp. aurantiaca strain JD37, complete genome | 6702062 | 62.8 |
| NZ_CP009365.1 | Pseudomonas soli strain SJ10, complete genome | 6247860 | 63.4 |
| NZ_CP009455.1 | Pseudomonas cremoricolorata strain ND07, complete genome | 4780403 | 63.2 |
| NZ_CP009533.1 | Pseudomonas rhizosphaerae strain DSM 16299, complete genome | 4688635 | 62.0 |
| NZ_CP009747.1 | Pseudomonas parafulva strain CRS01-1, complete genome | 5087619 | 63.5 |
| NZ_CP009974.1 | Pseudomonas putida S12, complete genome | 5798534 | 61.8 |
| NZ_CP009975.1 | Pseudomonas putida S12 plasmid pTTS12, complete sequence | 583900 | 57.9 |
| NZ_CP010359.1 | Pseudomonas plecoglossicida strain NyZ12, complete genome | 6233254 | 62.4 |
| NZ_CP010555.1 | Pseudomonas aeruginosa strain FRD1, complete genome | 6712339 | 66.1 |
| NZ_CP010892.1 | Pseudomonas sp. MRSN12121, complete genome | 6929263 | 63.8 |
| NZ_CP010893.1 | Pseudomonas sp. MRSN12121 plasmid pMRVIM0812, complete sequence | 36379 | 54.7 |
| NZ_CP010894.1 | Pseudomonas sp. MRSN12121 plasmid, complete sequence | 21121 | 62.8 |
| NZ_CP010896.1 | Pseudomonas simiae strain PCL1751, complete genome | 6143950 | 60.4 |
| NZ_CP010945.1 | Pseudomonas fluorescens NCIMB 11764, complete genome | 6998154 | 59.0 |
| NZ_CP010979.1 | Pseudomonas putida S13.1.2, complete genome | 6621848 | 62.3 |
| NZ_CP011020.1 | Pseudomonas chlororaphis strain UFB2, complete genome | 6360256 | 62.0 |
| NZ_CP011110.1 | Pseudomonas chlororaphis strain PCL1606, complete genome | 6646309 | 64.0 |
| NZ_CP011111.1 | Pseudomonas chlororaphis strain PCL1606 plasmid, complete sequence | 16587 | 56.3 |
| NZ_CP011117.2 | Pseudomonas synxantha strain LBUM223 chromosome, complete genome | 6690033 | 59.4 |
| NZ_CP011317.1 | Pseudomonas aeruginosa strain Carb01 63, complete genome | 7497593 | 65.6 |
| NZ_CP011507.1 | Pseudomonas trivialis strain IHBB745, complete genome | 6452803 | 59.9 |
| NZ_CP011508.1 | Pseudomonas mendocina strain NSYSU, complete genome | 5238364 | 64.6 |
| NZ_CP011525.1 | Pseudomonas sp. JY-Q, complete genome | 6178825 | 61.3 |
| NZ_CP011566.1 | Pseudomonas sp. DR 5-09, complete genome | 6427864 | 60.5 |
| NZ_CP011567.1 | Pseudomonas sp. GR 6-02, complete genome | 6500345 | 59.4 |
| NZ_CP011789.1 | Pseudomonas putida strain PC2, complete genome | 5808624 | 63.2 |
| NZ_CP011854.1 | Pseudomonas stutzeri strain SLG510A3-8, complete genome | 4650155 | 64.0 |
| NZ_CP011857.1 | Pseudomonas aeruginosa strain ATCC 27853, complete genome | 6833187 | 66.1 |
| NZ_CP011972.2 | Pseudomonas syringae pv. actinidiae ICMP 18884, complete genome | 6555569 | 58.4 |
| NZ_CP011973.1 | Pseudomonas syringae pv. actinidiae ICMP 18884 plasmid, complete sequence | 74423 | 56.0 |
| NZ_CP012001.1 | Pseudomonas aeruginosa DSM 50071 = NBRC 12689 strain DSM 50071 chromosome, complete genome | 6317050 | 66.5 |
| NZ_CP012066.1 | Pseudomonas aeruginosa strain F9676, complete genome | 6368008 | 66.5 |
| NZ_CP012179.1 | Pseudomonas syringae pv. actinidiae ICMP 18708, complete genome | 6555571 | 58.4 |
| NZ_CP012180.1 | Pseudomonas syringae pv. actinidiae ICMP 18708 plasmid, complete sequence | 74432 | 56.0 |
| NZ_CP012400.2 | Pseudomonas yamanorum strain LBUM636 chromosome, complete genome | 6856835 | 60.6 |
| NZ_CP012578.1 | Pseudomonas aeruginosa strain PA_D2, complete genome | 6642996 | 66.2 |
| NZ_CP012579.1 | Pseudomonas aeruginosa strain PA_D5, complete genome | 6681992 | 66.2 |
| NZ_CP012580.1 | Pseudomonas aeruginosa strain PA_D9, complete genome | 6645477 | 66.2 |
| NZ_CP012581.1 | Pseudomonas aeruginosa strain PA_D16, complete genome | 6681975 | 66.2 |
| NZ_CP012582.1 | Pseudomonas aeruginosa strain PA_D21, complete genome | 6639108 | 66.2 |
| NZ_CP012583.1 | Pseudomonas aeruginosa strain PA_D22, complete genome | 6681981 | 66.2 |
| NZ_CP012584.1 | Pseudomonas aeruginosa strain PA_D25, complete genome | 6683204 | 66.2 |
| NZ_CP012585.1 | Pseudomonas aeruginosa strain PA_D1, complete genome | 6643823 | 66.2 |
| NZ_CP012676.1 | Pseudomonas versuta strain L10.10 chromosome, complete genome | 5149788 | 58.2 |
| NZ_CP012679.1 | Pseudomonas aeruginosa strain PA1RG, complete genome | 6500439 | 66.3 |
| NZ_CP012680.1 | Pseudomonas brassicacearum strain LBUM300, complete genome | 6976764 | 60.8 |
| NZ_CP012830.1 | Pseudomonas fluorescens strain FW300-N2E3, complete genome | 6391871 | 58.8 |
| NZ_CP012831.1 | Pseudomonas fluorescens strain FW300-N2C3, complete genome | 7119102 | 61.3 |
| NZ_CP012901.1 | Pseudomonas aeruginosa strain N15-01092, complete sequence | 6972899 | 65.9 |
| NZ_CP013113.1 | Pseudomonas aeruginosa strain PAER4_119, complete genome | 6504659 | 66.4 |
| NZ_CP013124.1 | Pseudomonas mendocina S5.2, complete genome | 5120146 | 62.7 |
| NZ_CP013125.1 | Pseudomonas mendocina S5.2 plasmid pPME5, complete sequence | 252328 | 55.6 |
| NZ_CP013183.1 | Pseudomonas syringae pv. lapsa strain ATCC 10859, complete genome | 5918899 | 59.1 |
| NZ_CP013184.1 | Pseudomonas protegens strain H78, complete genome | 7032394 | 63.4 |
| NZ_CP013245.1 | Pseudomonas aeruginosa strain VA-134, complete genome | 6400418 | 66.4 |
| NZ_CP013477.1 | Pseudomonas aeruginosa strain SCVFeb, complete genome | 6213026 | 66.6 |
| NZ_CP013478.1 | Pseudomonas aeruginosa strain SCVJan, complete genome | 6213029 | 66.6 |
| NZ_CP013479.1 | Pseudomonas aeruginosa strain NHmuc, complete genome | 6213276 | 66.6 |
| NZ_CP013696.1 | Pseudomonas aeruginosa strain 12-4-4(59), complete genome | 6431911 | 66.3 |
| NZ_CP013861.1 | Pseudomonas fragi strain P121, complete genome | 5101809 | 58.6 |
| NZ_CP013987.1 | Pseudomonas oryzihabitans strain USDA-ARS-USMARC-56511, complete genome | 4834356 | 65.1 |
| NZ_CP013989.1 | Pseudomonas aeruginosa strain USDA-ARS-USMARC-41639, complete genome | 6364583 | 66.4 |
| NZ_CP013993.1 | Pseudomonas aeruginosa DHS01, complete genome | 7055752 | 65.8 |
| NZ_CP013997.1 | Pseudomonas monteilii strain USDA-ARS-USMARC-56711, complete genome | 4714359 | 64.4 |
| NZ_CP014025.1 | Pseudomonas fulva strain FDAARGOS_167 chromosome, complete genome | 4865091 | 61.6 |
| NZ_CP014061.1 | Pseudomonas monteilii strain FDAARGOS_171 plasmid unnamed, complete sequence | 60588 | 59.7 |
| NZ_CP014062.1 | Pseudomonas monteilii strain FDAARGOS_171 chromosome, complete genome | 5836684 | 61.9 |
| NZ_CP014158.1 | Pseudomonas citronellolis strain P3B5, complete genome | 6951444 | 67.1 |
| NZ_CP014262.1 | Pseudomonas corrugata strain RM1-1-4, complete genome | 6124363 | 60.7 |
| NZ_CP014343.1 | Pseudomonas putida strain 1A00316, complete genome | 5715815 | 64.4 |
| NZ_CP014546.1 | Pseudomonas azotoformans strain S4, complete genome | 6859618 | 60.3 |
| NZ_CP014693.1 | Pseudomonas brassicacearum strain L13-6-12, complete genome | 6715909 | 60.9 |
| NZ_CP014784.1 | Pseudomonas alcaligenes strain NEB 585, complete genome | 4406305 | 65.5 |
| NZ_CP014866.1 | Pseudomonas aeruginosa strain PA_154197, complete genome | 6445239 | 66.4 |
| NZ_CP014867.1 | Pseudomonas chlororaphis isolate 189 chromosome, complete genome | 6837781 | 62.7 |
| NZ_CP014870.1 | Pseudomonas silesiensis strain A3, complete genome | 6823539 | 59.6 |
| NZ_CP014947.1 | Pseudomonas koreensis strain D26, complete genome | 6301761 | 59.6 |
| NZ_CP014948.1 | Pseudomonas aeruginosa strain N17-1 chromosome, complete genome | 6370730 | 66.4 |
| NZ_CP014999.1 | Pseudomonas aeruginosa strain PA7790, complete genome | 7018690 | 66.0 |
| NZ_CP015000.1 | Pseudomonas aeruginosa strain PA7790 plasmid pPA7790, complete sequence | 49021 | 58.9 |
| NZ_CP015001.1 | Pseudomonas aeruginosa strain PA1088, complete genome | 6721480 | 66.1 |
| NZ_CP015002.1 | Pseudomonas aeruginosa strain PA8281, complete genome | 6928736 | 66.0 |
| NZ_CP015003.1 | Pseudomonas aeruginosa strain PA11803, complete genome | 7006578 | 66.0 |
| NZ_CP015117.1 | Pseudomonas aeruginosa strain ATCC 27853, complete genome | 6827737 | 66.1 |
| NZ_CP015202.1 | Pseudomonas putida B6-2 chromosome, complete genome | 6377271 | 61.6 |
| NZ_CP015377.1 | Pseudomonas aeruginosa strain BAMCPA07-48, complete genome | 7021552 | 66.0 |
| NZ_CP015600.1 | Pseudomonas antarctica strain PAMC 27494 chromosome, complete genome | 6441449 | 59.8 |
| NZ_CP015601.1 | Pseudomonas antarctica strain PAMC 27494 plasmid pP27494_1, complete sequence | 135475 | 54.7 |
| NZ_CP015602.1 | Pseudomonas antarctica strain PAMC 27494 plasmid pP27494_2, complete sequence | 30116 | 49.4 |
| NZ_CP015637.1 | Pseudomonas fluorescens strain L321, complete genome | 6641144 | 60.8 |
| NZ_CP015638.1 | Pseudomonas fluorescens strain L111, complete genome | 6606606 | 60.8 |
| NZ_CP015639.1 | Pseudomonas lurida strain L228 chromosome, complete genome | 6175426 | 60.8 |
| NZ_CP015640.1 | Pseudomonas lurida strain L228 plasmid, complete sequence | 77900 | 52.5 |
| NZ_CP015641.1 | Pseudomonas stutzeri strain 273, complete genome | 5030940 | 60.3 |
| NZ_CP015852.1 | Pseudomonas koreensis strain CRS05-R5 chromosome, complete genome | 5991225 | 60.6 |
| NZ_CP015876.1 | Pseudomonas putida SJTE-1, complete genome | 6077201 | 61.8 |
| NZ_CP015877.1 | Pseudomonas aeruginosa SJTD-1 chromosome, complete genome | 6243825 | 66.5 |
| NZ_CP015878.1 | Pseudomonas citronellolis strain SJTE-3 chromosome, complete genome | 7309421 | 67.0 |
| NZ_CP015879.1 | Pseudomonas citronellolis strain SJTE-3 plasmid pRBL16, complete sequence | 370338 | 56.6 |
| NZ_CP015992.1 | Pseudomonas sp. TCU-HL1 chromosome, complete genome | 6244007 | 63.2 |
| NZ_CP015993.1 | Pseudomonas sp. TCU-HL1 plasmid pTCUH, complete sequence | 12690 | 58.2 |
| NZ_CP016162.1 | Pseudomonas alcaliphila JAB1 chromosome, complete genome | 5340293 | 62.5 |
| NZ_CP016212.1 | Pseudomonas putida JB, complete genome | 5846746 | 61.8 |
| NZ_CP016214.1 | Pseudomonas aeruginosa strain PA121617 chromosome, complete genome | 6430493 | 66.4 |
| NZ_CP016215.1 | Pseudomonas aeruginosa strain PA121617 plasmid pBM413, complete sequence | 423017 | 56.4 |
| NZ_CP016446.1 | Pseudomonas putida strain IEC33019 plasmid pIEC33019, complete sequence | 52710 | 59.2 |
| NZ_CP016634.1 | Pseudomonas putida strain IEC33019 chromosome, complete genome | 5794410 | 62.3 |
| NZ_CP016849.1 | Pseudomonas sp. TMW 2.1634 chromosome, complete genome | 5510371 | 59.1 |
| NZ_CP016850.1 | Pseudomonas sp. TMW 2.1634 plasmid pL21564-1, complete sequence | 99718 | 55.9 |
| NZ_CP016851.1 | Pseudomonas sp. TMW 2.1634 plasmid pL21634-2, complete sequence | 56859 | 51.4 |
| NZ_CP016955.1 | Pseudomonas aeruginosa strain RIVM-EMC2982 chromosome, complete genome | 7380063 | 65.7 |
| NZ_CP017007.1 | Pseudomonas syringae pv. actinidiae strain NZ-45 chromosome, complete genome | 6665031 | 58.4 |
| NZ_CP017008.1 | Pseudomonas syringae pv. actinidiae strain NZ-45 plasmid pPsa20586, complete sequence | 76088 | 56.0 |
| NZ_CP017009.1 | Pseudomonas syringae pv. actinidiae strain NZ-47 chromosome, complete genome | 6545910 | 58.4 |
| NZ_CP017010.1 | Pseudomonas syringae pv. actinidiae strain NZ-47 plasmid pPsa22180a, complete sequence | 74424 | 56.0 |
| NZ_CP017011.1 | Pseudomonas syringae pv. actinidiae strain NZ-47 plasmid pPsa22180b, complete sequence | 55944 | 53.2 |
| NZ_CP017073.1 | Pseudomonas putida strain PP112420, complete genome | 6031212 | 62.0 |
| NZ_CP017099.1 | Pseudomonas aeruginosa strain DN1 chromosome, complete genome | 6641902 | 66.3 |
| NZ_CP017149.1 | Pseudomonas aeruginosa strain ATCC 15692 chromosome, complete genome | 6276434 | 66.5 |
| NZ_CP017290.1 | Pseudomonas sp. LPH1 chromosome, complete genome | 5229495 | 62.7 |
| NZ_CP017293.1 | Pseudomonas aeruginosa strain PA83 chromosome, complete genome | 6816227 | 66.1 |
| NZ_CP017294.1 | Pseudomonas aeruginosa strain PA83 plasmid unnamed1, complete sequence | 398087 | 59.4 |
| NZ_CP017296.1 | Pseudomonas fluorescens strain Pt14 chromosome, complete genome | 5841722 | 60.3 |
| NZ_CP017306.1 | Pseudomonas aeruginosa strain PA_150577 chromosome, complete genome | 6334472 | 66.5 |
| NZ_CP017353.1 | Pseudomonas aeruginosa strain FA-HZ1, complete genome | 6866790 | 66.2 |
| NZ_CP017432.1 | Pseudomonas sp. Lz4W chromosome, complete genome | 5018820 | 58.7 |
| NZ_CP017687.1 | Pseudomonas lundensis strain AU1044 chromosome, complete genome | 4814265 | 58.7 |
| NZ_CP017886.1 | Pseudomonas frederiksbergensis strain ERDD5:01 chromosome, complete genome | 5747498 | 58.7 |
| NZ_CP017887.1 | Pseudomonas frederiksbergensis strain ERDD5:01 plasmid unnamed1, complete sequence | 371069 | 55.0 |
| NZ_CP017969.1 | Pseudomonas aeruginosa isolate B10W, complete genome | 6723378 | 66.2 |
| NZ_CP018048.1 | Pseudomonas aeruginosa strain DN1 plasmid unnamed1, complete sequence | 317349 | 56.9 |
| NZ_CP018049.1 | Pseudomonas orientalis strain F9 chromosome, complete genome | 5986236 | 60.4 |
| NZ_CP018202.1 | Pseudomonas syringae pv. actinidiae ICMP 9853, complete genome | 6439609 | 58.7 |
| NZ_CP018203.1 | Pseudomonas syringae pv. actinidiae ICMP 9853 plasmid p9853_A, complete sequence | 34963 | 54.6 |
| NZ_CP018204.1 | Pseudomonas syringae pv. actinidiae ICMP 9853 plasmid p9853_B, complete sequence | 32947 | 55.3 |
| NZ_CP018319.1 | Pseudomonas frederiksbergensis strain AS1, complete genome | 6126864 | 58.9 |
| NZ_CP018320.1 | Pseudomonas frederiksbergensis strain AS1 plasmid, complete sequence | 81841 | 56.2 |
| NZ_CP018420.1 | Pseudomonas veronii strain R02 chromosome, complete genome | 6852809 | 61.1 |
| NZ_CP018758.1 | Pseudomonas psychrotolerans strain PRS08-11306, complete genome | 5271920 | 64.8 |
| NZ_CP018759.1 | Pseudomonas psychrotolerans strain PRS08-11306 plasmid pPRS08-11306, complete sequence | 114250 | 65.1 |
| NZ_CP018846.1 | Pseudomonas putida strain AA7 chromosome, complete genome | 6137024 | 62.5 |
| NZ_CP019338.1 | Pseudomonas aeruginosa strain L10 chromosome, complete genome | 6661962 | 66.1 |
| NZ_CP019396.1 | Pseudomonas sp. R32 chromosome, complete genome | 5646108 | 62.4 |
| NZ_CP019397.1 | Pseudomonas sp. S19 chromosome, complete genome | 6098674 | 60.9 |
| NZ_CP019398.1 | Pseudomonas sp. S34 chromosome, complete genome | 6269896 | 59.8 |
| NZ_CP019399.1 | Pseudomonas chlororaphis strain R47 chromosome, complete genome | 7197297 | 62.6 |
| NZ_CP019426.1 | Pseudomonas sp. R84 chromosome, complete genome | 6601805 | 59.1 |
| NZ_CP019427.1 | Pseudomonas sp. S04 chromosome, complete genome | 6098669 | 60.9 |
| NZ_CP019428.1 | Pseudomonas sp. R76 chromosome, complete genome | 6821900 | 60.0 |
| NZ_CP019429.1 | Pseudomonas sp. R76 plasmid p76, complete sequence | 7195 | 47.1 |
| NZ_CP019431.1 | Pseudomonas sp. S35 chromosome, complete genome | 6611402 | 60.1 |
| NZ_CP019432.1 | Pseudomonas sp. S49 chromosome, complete genome | 6659551 | 59.2 |
| NZ_CP019730.1 | Pseudomonas syringae pv. actinidiae strain CRAFRU 12.29 chromosome, complete genome | 6545549 | 58.4 |
| NZ_CP019731.1 | Pseudomonas syringae pv. actinidiae strain CRAFRU 12.29 plasmid unnamed, complete sequence | 74460 | 56.0 |
| NZ_CP019732.1 | Pseudomonas syringae pv. actinidiae strain CRAFRU 14.08 chromosome, complete genome | 6546028 | 58.4 |
| NZ_CP019733.1 | Pseudomonas syringae pv. actinidiae strain CRAFRU 14.08 plasmid unnamed, complete sequence | 74460 | 56.0 |
| NZ_CP019856.1 | Pseudomonas azotoformans strain F77 chromosome, complete genome | 6581295 | 60.6 |
| NZ_CP019871.1 | Pseudomonas syringae pv. tomato strain B13-200 chromosome, complete genome | 6220728 | 58.6 |
| NZ_CP019872.1 | Pseudomonas syringae pv. tomato strain B13-200 plasmid pB13-200A, complete sequence | 125801 | 60.1 |
| NZ_CP019873.1 | Pseudomonas syringae pv. tomato strain B13-200 plasmid pB13-200B, complete sequence | 93873 | 55.2 |
| NZ_CP019874.1 | Pseudomonas syringae pv. tomato strain B13-200 plasmid pB13-200C, complete sequence | 82965 | 55.4 |
| NZ_CP019947.1 | Pseudomonas sp. CC6-YY-74 chromosome, complete genome | 5040792 | 61.7 |
| NZ_CP019952.1 | Pseudomonas parafulva strain PRS09-11288 chromosome, complete genome | 4690783 | 61.7 |
| NZ_CP020100.1 | Pseudomonas sp. S-6-2 chromosome, complete genome | 4035153 | 60.1 |
| NZ_CP020351.1 | Pseudomonas amygdali pv. lachrymans strain NM002 chromosome, complete genome | 6009235 | 58.1 |
| NZ_CP020560.1 | Pseudomonas aeruginosa strain CR1 chromosome, complete genome | 6118054 | 66.9 |
| NZ_CP020561.2 | Pseudomonas aeruginosa strain CR1 plasmid pCR1, complete sequence | 46804 | 59.2 |
| NZ_CP020602.1 | Pseudomonas aeruginosa strain E6130952 plasmid pJHX613, complete sequence | 36454 | 61.3 |
| NZ_CP020603.1 | Pseudomonas aeruginosa strain E6130952 chromosome, complete genome | 7040952 | 65.9 |
| NZ_CP020659.1 | Pseudomonas aeruginosa PAK chromosome, complete genome | 6283316 | 66.3 |
| NZ_CP020703.1 | Pseudomonas aeruginosa strain PASGNDM345 chromosome, complete genome | 6893164 | 66.1 |
| NZ_CP020704.1 | Pseudomonas aeruginosa strain PASGNDM699 chromosome, complete genome | 6985102 | 66.0 |
| NZ_CP020892.1 | Pseudomonas sp. M30-35 chromosome, complete genome | 4926954 | 54.3 |
| NZ_CP021132.1 | Pseudomonas fragi strain NMC25 chromosome, complete genome | 5001447 | 59.3 |
| NZ_CP021133.1 | Pseudomonas fragi strain NMC25 plasmid unnamed1, complete sequence | 79891 | 56.1 |
| NZ_CP021134.1 | Pseudomonas fragi strain NMC25 plasmid unnamed2, complete sequence | 54359 | 56.4 |
| NZ_CP021135.1 | Pseudomonas fragi strain NMC25 plasmid unnamed3, complete sequence | 16437 | 56.7 |
| NZ_CP021645.1 | Pseudomonas psychrotolerans strain CS51 chromosome, complete genome | 5364174 | 64.7 |
| NZ_CP021774.1 | Pseudomonas aeruginosa strain Pa124 chromosome, complete genome | 7008516 | 65.8 |
| NZ_CP021775.1 | Pseudomonas aeruginosa strain Pa58 chromosome, complete genome | 7241575 | 65.8 |
| NZ_CP021999.1 | Pseudomonas aeruginosa strain Pa84 chromosome, complete genome | 6566724 | 66.2 |
| NZ_CP022000.1 | Pseudomonas aeruginosa strain Pa127 chromosome, complete genome | 7148302 | 65.7 |
| NZ_CP022001.1 | Pseudomonas aeruginosa strain Pa1207 chromosome, complete genome | 7411863 | 65.7 |
| NZ_CP022002.1 | Pseudomonas aeruginosa strain Pa1242 chromosome, complete genome | 7050510 | 65.8 |
| NZ_CP022097.2 | Pseudomonas protegens strain FDAARGOS_307 chromosome, complete genome | 7075815 | 63.3 |
| NZ_CP022411.1 | Pseudomonas sp. RU47 chromosome, complete genome | 6663463 | 59.2 |
| NZ_CP022478.1 | Pseudomonas aeruginosa strain LW chromosome, complete genome | 6824837 | 66.0 |
| NZ_CP022525.1 | Pseudomonas aeruginosa strain Ocean-1175 chromosome, complete genome | 6943220 | 66.0 |
| NZ_CP022526.1 | Pseudomonas aeruginosa strain Ocean-1155 chromosome, complete genome | 6952237 | 66.0 |
| NZ_CP022560.1 | Pseudomonas putida strain B1 chromosome, complete genome | 6368486 | 62.0 |
| NZ_CP022561.1 | Pseudomonas putida strain B4 chromosome, complete genome | 6011680 | 61.8 |
| NZ_CP022562.1 | Pseudomonas monteilii strain B5 chromosome, complete genome | 5948598 | 61.8 |
| NZ_CP022563.1 | Pseudomonas monteilii strain B5 plasmid pSH5-1, complete sequence | 130536 | 56.7 |
| NZ_CP023048.1 | Pseudomonas fulva strain SB1 chromosome, complete genome | 5011694 | 61.3 |
| NZ_CP023255.1 | Pseudomonas aeruginosa strain CCUG 70744 chromosome, complete genome | 6859232 | 66.0 |
| NZ_CP023269.1 | Pseudomonas sp. MYb193 chromosome, complete genome | 6211636 | 59.9 |
| NZ_CP023272.1 | Pseudomonas lurida strain MYb11 chromosome, complete genome | 6100532 | 60.8 |
| NZ_CP023299.1 | Pseudomonas mosselii strain BS011 chromosome, complete genome | 5751088 | 64.3 |
| NZ_CP023316.1 | Pseudomonas aeruginosa strain PPF-1 chromosome, complete genome | 6930893 | 65.9 |
| NZ_CP023466.1 | Pseudomonas frederiksbergensis strain KNU-15 chromosome, complete genome | 6595804 | 59.4 |
| NZ_CP023641.1 | Pseudomonas mendocina strain MAE1-K chromosome, complete genome | 5157724 | 64.5 |
| NZ_CP023969.1 | Pseudomonas sp. FDAARGOS_380 chromosome, complete genome | 6499364 | 59.7 |
| NZ_CP024085.1 | Pseudomonas putida strain E41 chromosome, complete genome | 6093023 | 62.2 |
| NZ_CP024086.1 | Pseudomonas putida strain E46 chromosome, complete genome | 6180967 | 62.3 |
| NZ_CP024159.1 | Pseudomonas mosselii strain PtA1 chromosome, complete genome | 5742165 | 64.4 |
| NZ_CP024477.1 | Pseudomonas aeruginosa strain 12939 chromosome, complete genome | 6621378 | 66.2 |
| NZ_CP024478.1 | Pseudomonas sp. HLS-6 chromosome, complete genome | 5276694 | 59.9 |
| NZ_CP024630.1 | Pseudomonas aeruginosa strain PA59 chromosome, complete genome | 6926363 | 66.1 |
| NZ_CP024631.1 | Pseudomonas aeruginosa strain PA59 plasmid unnamed1, complete sequence | 46627 | 59.1 |
| NZ_CP024646.1 | Pseudomonas syringae isolate inb918 chromosome, complete genome | 6381237 | 60.7 |
| NZ_CP024712.1 | Pseudomonas syringae pv. actinidiae strain MAFF212063 chromosome, complete genome | 6556999 | 58.4 |
| NZ_CP024713.1 | Pseudomonas syringae pv. actinidiae strain MAFF212063 plasmid pMAFF212063-A, complete sequence | 68316 | 55.7 |
| NZ_CP024714.1 | Pseudomonas syringae pv. actinidiae strain MAFF212063 plasmid pMAFF212063-B, complete sequence | 68156 | 56.1 |
| NZ_CP025035.2 | Pseudomonas sp. SGAir0191 chromosome, complete genome | 5071227 | 61.4 |
| NZ_CP025049.1 | Pseudomonas aeruginosa strain PB369 chromosome, complete genome | 6526814 | 66.0 |
| NZ_CP025050.1 | Pseudomonas aeruginosa strain PB368 chromosome, complete genome | 6638559 | 66.0 |
| NZ_CP025051.1 | Pseudomonas aeruginosa strain PB353 chromosome, complete genome | 6437515 | 66.4 |
| NZ_CP025052.1 | Pseudomonas aeruginosa strain PB353 plasmid pPB353_1, complete sequence | 59923 | 57.3 |
| NZ_CP025053.1 | Pseudomonas aeruginosa strain PB354 chromosome, complete genome | 6434352 | 66.4 |
| NZ_CP025054.1 | Pseudomonas aeruginosa strain PB354 plasmid pPB354_1, complete sequence | 59923 | 57.3 |
| NZ_CP025055.1 | Pseudomonas aeruginosa strain PB350 chromosome, complete genome | 6752870 | 66.2 |
| NZ_CP025056.1 | Pseudomonas aeruginosa strain PB367 chromosome, complete genome | 6752906 | 66.2 |
| NZ_CP025149.2 | Pseudomonas stutzeri strain SGAir0442 chromosome, complete genome | 4524655 | 64.0 |
| NZ_CP025229.1 | Pseudomonas sp. AK6U chromosome, complete genome | 6909530 | 65.8 |
| NZ_CP025309.1 | Pseudomonas chlororaphis strain Lzh-T5 chromosome, complete genome | 6826693 | 63.1 |
| NZ_CP025542.1 | Pseudomonas fluorescens strain 2P24 chromosome, complete genome | 6610571 | 60.8 |
| NZ_CP025624.1 | Pseudomonas sp. NC02 chromosome, complete genome | 6890566 | 61.1 |
| NZ_CP026115.1 | Pseudomonas putida strain W5 chromosome, complete genome | 6166183 | 62.7 |
| NZ_CP026332.1 | Pseudomonas sp. XWY-1 chromosome, complete genome | 5902716 | 61.9 |
| NZ_CP026333.1 | Pseudomonas sp. XWY-1 plasmid, complete genome | 394537 | 56.3 |
| NZ_CP026386.1 | Pseudomonas sp. PONIH3 chromosome, complete genome | 6313552 | 63.4 |
| NZ_CP026557.1 | Pseudomonas amygdali pv. morsprunorum strain R15244 plasmid p1_tig4, complete sequence | 81536 | 56.5 |
| NZ_CP026558.1 | Pseudomonas amygdali pv. morsprunorum strain R15244 chromosome, complete genome | 6109228 | 58.2 |
| NZ_CP026559.1 | Pseudomonas amygdali pv. morsprunorum strain R15244 plasmid p2_tig3, complete sequence | 168854 | 56.1 |
| NZ_CP026560.1 | Pseudomonas amygdali pv. morsprunorum strain R15244 plasmid p3_tig5, complete sequence | 45535 | 56.4 |
| NZ_CP026561.1 | Pseudomonas amygdali pv. morsprunorum strain R15244 plasmid p4_tig6, complete sequence | 40810 | 55.1 |
| NZ_CP026562.1 | Pseudomonas avellanae strain R2leaf chromosome, complete genome | 6242845 | 58.6 |
| NZ_CP026563.1 | Pseudomonas avellanae strain R2leaf plasmid p1_tig4, complete sequence | 97840 | 55.5 |
| NZ_CP026564.1 | Pseudomonas avellanae strain R2leaf plasmid p2_tig5, complete sequence | 102862 | 54.2 |
| NZ_CP026565.1 | Pseudomonas avellanae strain R2leaf plasmid p3_tig6, complete sequence | 69519 | 56.7 |
| NZ_CP026566.1 | Pseudomonas avellanae strain R2leaf plasmid p4_tig8, complete sequence | 42783 | 54.4 |
| NZ_CP026567.1 | Pseudomonas avellanae strain R2leaf plasmid p5_tig9, complete sequence | 20491 | 52.8 |
| NZ_CP026568.1 | Pseudomonas syringae pv. syringae strain Pss9097 chromosome, complete genome | 5929959 | 59.3 |
| NZ_CP026674.1 | Pseudomonas sp. SWI44 chromosome, complete genome | 5919083 | 61.8 |
| NZ_CP026675.1 | Pseudomonas sp. SWI36 chromosome, complete genome | 6172256 | 61.8 |
| NZ_CP026676.1 | Pseudomonas sp. SWI6 chromosome, complete genome | 5652054 | 61.8 |
| NZ_CP026680.1 | Pseudomonas aeruginosa strain F5677 chromosome, complete genome | 6645227 | 65.9 |
| NZ_CP026880.1 | Pseudomonas sp. LH1G9 chromosome, complete genome | 6548975 | 61.0 |
| NZ_CP026881.1 | Pseudomonas sp. LG1D9 chromosome, complete genome | 6289439 | 60.5 |
| NZ_CP027165.1 | Pseudomonas aeruginosa strain AR_0360 chromosome, complete genome | 6463575 | 66.4 |
| NZ_CP027166.1 | Pseudomonas aeruginosa strain AR_0357 chromosome, complete genome | 7162784 | 65.8 |
| NZ_CP027167.1 | Pseudomonas aeruginosa strain AR_0356 plasmid unnamed3, complete sequence | 165365 | 55.8 |
| NZ_CP027168.1 | Pseudomonas aeruginosa strain AR_0356 plasmid unnamed1, complete sequence | 57053 | 60.9 |
| NZ_CP027169.1 | Pseudomonas aeruginosa strain AR_0356 chromosome, complete genome | 6586916 | 66.4 |
| NZ_CP027170.1 | Pseudomonas aeruginosa strain AR_0356 plasmid unnamed2, complete sequence | 438531 | 57.1 |
| NZ_CP027171.1 | Pseudomonas aeruginosa strain AR_0354 chromosome, complete genome | 6747010 | 66.1 |
| NZ_CP027172.1 | Pseudomonas aeruginosa strain AR_0353 chromosome, complete genome | 7240677 | 65.7 |
| NZ_CP027173.1 | Pseudomonas aeruginosa strain AR_0353 plasmid unnamed1, complete sequence | 41559 | 60.8 |
| NZ_CP027174.1 | Pseudomonas aeruginosa strain AR_0230 chromosome, complete genome | 7012922 | 65.9 |
| NZ_CP027175.1 | Pseudomonas aeruginosa strain AR_0230 plasmid unnamed1 | 71782 | 59.7 |
| NZ_CP027176.1 | Pseudomonas aeruginosa strain AR_0230 plasmid unnamed2 | 1350 | 48.0 |
| NZ_CP027218.1 | Pseudomonas sp. DTU12.3 chromosome, complete genome | 6268469 | 59.5 |
| NZ_CP027538.1 | Pseudomonas aeruginosa strain AR_0095 chromosome, complete genome | 6822666 | 66.1 |
| NZ_CP027543.1 | Pseudomonas stutzeri strain DW2-1 chromosome, complete genome | 4400660 | 63.0 |
| NZ_CP027656.1 | Pseudomonas chlororaphis subsp. piscium strain ZJU60 chromosome, complete genome | 6818002 | 62.8 |
| NZ_CP027657.1 | Pseudomonas mendocina strain NEB698 chromosome, complete genome | 5773548 | 62.4 |
| NZ_CP027664.1 | Pseudomonas stutzeri strain 1W1-1A chromosome, complete genome | 4454378 | 64.2 |
| NZ_CP027705.1 | Pseudomonas sp. CMR5c chromosome, complete genome | 6796817 | 63.5 |
| NZ_CP027706.1 | Pseudomonas sp. CMR12a chromosome, complete genome | 6896611 | 62.8 |
| NZ_CP027707.1 | Pseudomonas chlororaphis subsp. piscium strain DSM 21509 chromosome, complete genome | 7065155 | 62.6 |
| NZ_CP027708.1 | Pseudomonas chlororaphis subsp. piscium strain ATCC 17411 chromosome, complete genome | 7212397 | 62.5 |
| NZ_CP027709.1 | Pseudomonas chlororaphis subsp. piscium strain ATCC 17809 chromosome, complete genome | 7218893 | 62.4 |
| NZ_CP027710.1 | Pseudomonas chlororaphis subsp. piscium strain SLPH10 chromosome, complete genome | 7227643 | 62.5 |
| NZ_CP027711.1 | Pseudomonas chlororaphis subsp. piscium strain ChPhzTR44 chromosome, complete genome | 6878216 | 62.8 |
| NZ_CP027712.1 | Pseudomonas chlororaphis subsp. chlororaphis strain DSM 50083 chromosome, complete genome | 6808187 | 63.0 |
| NZ_CP027713.1 | Pseudomonas chlororaphis strain TAMOak81 chromosome, complete genome | 6711360 | 63.0 |
| NZ_CP027714.1 | Pseudomonas chlororaphis strain ATCC 17415 chromosome, complete genome | 6670884 | 63.0 |
| NZ_CP027715.1 | Pseudomonas chlororaphis subsp. aurantiaca strain M12 chromosome, complete genome | 6730980 | 63.0 |
| NZ_CP027716.1 | Pseudomonas chlororaphis strain Pb-St2 chromosome, complete genome | 6613798 | 63.2 |
| NZ_CP027717.1 | Pseudomonas chlororaphis subsp. aurantiaca strain PCM 2210 chromosome, complete genome | 6854483 | 63.0 |
| NZ_CP027718.1 | Pseudomonas chlororaphis subsp. aurantiaca strain Q16 chromosome, complete genome | 6928549 | 62.8 |
| NZ_CP027719.1 | Pseudomonas chlororaphis subsp. aureofaciens strain P2 chromosome, complete genome | 7203062 | 62.8 |
| NZ_CP027720.1 | Pseudomonas chlororaphis subsp. aureofaciens strain DSM 6698 chromosome, complete genome | 7037850 | 62.7 |
| NZ_CP027721.1 | Pseudomonas chlororaphis subsp. aureofaciens strain ChPhzTR36 chromosome, complete genome | 6934077 | 62.7 |
| NZ_CP027722.1 | Pseudomonas chlororaphis subsp. aureofaciens strain C50 chromosome, complete genome | 6741275 | 63.0 |
| NZ_CP027723.1 | Pseudomonas orientalis strain 8B chromosome, complete genome | 5919814 | 60.6 |
| NZ_CP027724.1 | Pseudomonas orientalis strain L1-3-08 chromosome, complete genome | 5962679 | 60.7 |
| NZ_CP027725.1 | Pseudomonas orientalis strain R2-66-08W chromosome, complete genome | 5929469 | 60.7 |
| NZ_CP027726.1 | Pseudomonas orientalis strain R4-35-08 chromosome, complete genome | 5912532 | 60.7 |
| NZ_CP027727.1 | Pseudomonas sp. R5-89-07 chromosome, complete genome | 5997145 | 60.6 |
| NZ_CP027728.1 | Pseudomonas sp. R2-37-08W chromosome, complete genome | 5833149 | 60.4 |
| NZ_CP027729.1 | Pseudomonas sp. R3-18-08 chromosome, complete genome | 5810083 | 60.4 |
| NZ_CP027730.1 | Pseudomonas sp. R3-52-08 chromosome, complete genome | 5926806 | 60.3 |
| NZ_CP027731.1 | Pseudomonas sp. R2-60-08W chromosome, complete genome | 5838856 | 60.4 |
| NZ_CP027732.1 | Pseudomonas sp. R4-35-07 chromosome, complete genome | 5836505 | 60.3 |
| NZ_CP027733.1 | Pseudomonas sp. R4-39-08 chromosome, complete genome | 5882212 | 60.4 |
| NZ_CP027734.1 | Pseudomonas sp. R1-43-08 chromosome, complete genome | 5645326 | 60.6 |
| NZ_CP027735.1 | Pseudomonas chlororaphis subsp. piscium strain DTR133 chromosome, complete genome | 7064618 | 62.7 |
| NZ_CP027736.1 | Pseudomonas chlororaphis subsp. piscium strain PCL1391 chromosome, complete genome | 6870622 | 62.8 |
| NZ_CP027737.1 | Pseudomonas chlororaphis subsp. piscium strain PCL1607 chromosome, complete genome | 6913645 | 62.8 |
| NZ_CP027738.1 | Pseudomonas chlororaphis subsp. piscium strain ChPhzS135 chromosome, complete genome | 6940016 | 62.8 |
| NZ_CP027739.1 | Pseudomonas chlororaphis subsp. piscium strain ToZa7 chromosome, complete genome | 7015602 | 62.6 |
| NZ_CP027740.1 | Pseudomonas chlororaphis subsp. piscium strain ChPhzS140 chromosome, complete genome | 7074317 | 62.6 |
| NZ_CP027741.1 | Pseudomonas chlororaphis subsp. aurantiaca strain 449 chromosome, complete genome | 6962068 | 62.9 |
| NZ_CP027742.1 | Pseudomonas chlororaphis subsp. aurantiaca strain 464 chromosome, complete genome | 6964452 | 62.9 |
| NZ_CP027743.1 | Pseudomonas chlororaphis subsp. aurantiaca strain CW2 chromosome, complete genome | 6925198 | 62.8 |
| NZ_CP027744.1 | Pseudomonas chlororaphis subsp. aurantiaca strain M71 chromosome, complete genome | 6807002 | 62.9 |
| NZ_CP027745.1 | Pseudomonas chlororaphis subsp. aurantiaca strain K27 chromosome, complete genome | 6867699 | 62.8 |
| NZ_CP027746.1 | Pseudomonas chlororaphis subsp. aurantiaca strain DSM 19603 chromosome, complete genome | 7109779 | 62.9 |
| NZ_CP027747.1 | Pseudomonas chlororaphis subsp. aureofaciens strain 66 chromosome, complete genome | 6797278 | 63.0 |
| NZ_CP027748.1 | Pseudomonas chlororaphis subsp. aureofaciens strain ChPhzS23 chromosome, complete genome | 6756093 | 63.0 |
| NZ_CP027749.1 | Pseudomonas chlororaphis subsp. aureofaciens strain ChPhzTR39 chromosome, complete genome | 7046099 | 62.7 |
| NZ_CP027750.1 | Pseudomonas chlororaphis subsp. aureofaciens strain ChPhzS24 chromosome, complete genome | 6889553 | 62.9 |
| NZ_CP027751.1 | Pseudomonas chlororaphis subsp. aureofaciens strain ChPhzTR18 chromosome, complete genome | 6873200 | 62.9 |
| NZ_CP027752.1 | Pseudomonas chlororaphis subsp. aureofaciens strain ChPhzTR38 chromosome, complete genome | 6947716 | 62.8 |
| NZ_CP027753.1 | Pseudomonas chlororaphis strain B25 chromosome, complete genome | 7016593 | 62.0 |
| NZ_CP027754.1 | Pseudomonas synxantha strain 30B chromosome, complete genome | 6776271 | 59.7 |
| NZ_CP027755.1 | Pseudomonas synxantha strain 2-79 chromosome, complete genome | 6460613 | 59.8 |
| NZ_CP027756.1 | Pseudomonas synxantha strain R6-28-08 chromosome, complete genome | 6562330 | 59.6 |
| NZ_CP027757.1 | Pseudomonas synxantha strain R2-4-08W chromosome, complete genome | 6193282 | 60.0 |
| NZ_CP027758.1 | Pseudomonas synxantha strain R2-54-08W chromosome, complete genome | 6277070 | 59.7 |
| NZ_CP027759.1 | Pseudomonas sp. R2-7-07 chromosome, complete genome | 5799132 | 60.4 |
| NZ_CP027760.1 | Pseudomonas sp. R4-34-07 chromosome, complete genome | 5924968 | 60.4 |
| NZ_CP027761.1 | Pseudomonas sp. R11-23-07 chromosome, complete genome | 5689077 | 60.6 |
| NZ_CP027762.1 | Pseudomonas sp. LBUM920 chromosome, complete genome | 6533866 | 60.7 |
| NZ_CP028132.1 | Pseudomonas aeruginosa strain YB01 chromosome, complete genome | 6299465 | 66.6 |
| NZ_CP028162.1 | Pseudomonas aeruginosa strain MRSN12280 chromosome, complete genome | 7050928 | 66.0 |
| NZ_CP028490.1 | Pseudomonas syringae pv. atrofaciens strain LMG5095 chromosome, complete genome | 6080544 | 59.0 |
| NZ_CP028584.2 | Pseudomonas aeruginosa strain WCHPA075019 chromosome, complete genome | 6886080 | 66.0 |
| NZ_CP028826.1 | Pseudomonas fluorescens strain MS82 chromosome, complete genome | 6207556 | 60.6 |
| NZ_CP028848.1 | Pseudomonas aeruginosa strain IMP67 chromosome, complete genome | 6501414 | 66.4 |
| NZ_CP028849.1 | Pseudomonas aeruginosa strain IMP68 chromosome, complete genome | 6481697 | 66.4 |
| NZ_CP028917.1 | Pseudomonas aeruginosa strain JB2 chromosome, complete genome | 6867314 | 66.0 |
| NZ_CP028959.1 | Pseudomonas aeruginosa strain IMP66 chromosome, complete genome | 6486336 | 66.4 |
| NZ_CP029088.1 | Pseudomonas aeruginosa strain AR445 chromosome, complete genome | 7125975 | 65.8 |
| NZ_CP029089.1 | Pseudomonas aeruginosa strain AR444 chromosome, complete genome | 6853499 | 66.1 |
| NZ_CP029090.1 | Pseudomonas aeruginosa strain AR442 chromosome, complete genome | 7267567 | 65.8 |
| NZ_CP029091.1 | Pseudomonas aeruginosa strain AR441 plasmid unnamed1, complete sequence | 165365 | 55.8 |
| NZ_CP029092.1 | Pseudomonas aeruginosa strain AR441 plasmid unnamed2, complete sequence | 57052 | 60.9 |
| NZ_CP029093.1 | Pseudomonas aeruginosa strain AR441 chromosome, complete genome | 6584825 | 66.4 |
| NZ_CP029094.1 | Pseudomonas aeruginosa strain AR441 plasmid unnamed3, complete sequence | 438529 | 57.1 |
| NZ_CP029095.1 | Pseudomonas aeruginosa strain AR439 plasmid unnamed1 | 1129 | 54.4 |
| NZ_CP029096.1 | Pseudomonas aeruginosa strain AR439 plasmid unnamed2, complete sequence | 437392 | 56.9 |
| NZ_CP029097.1 | Pseudomonas aeruginosa strain AR439 chromosome, complete genome | 7139518 | 65.9 |
| NZ_CP029482.1 | Pseudomonas sp. 31-12 chromosome, complete genome | 6730253 | 59.1 |
| NZ_CP029605.1 | Pseudomonas aeruginosa strain 24Pae112 chromosome, complete genome | 7097241 | 66.0 |
| NZ_CP029608.1 | Pseudomonas kribbensis strain 46-2 chromosome, complete genome | 6324282 | 60.5 |
| NZ_CP029660.1 | Pseudomonas aeruginosa strain AR_0446 chromosome, complete genome | 6475581 | 66.3 |
| NZ_CP029693.1 | Pseudomonas putida strain JBC17 chromosome, complete genome | 6845198 | 60.3 |
| NZ_CP029707.1 | Pseudomonas aeruginosa strain K34-7 chromosome, complete genome | 7038012 | 65.9 |
| NZ_CP029708.1 | Pseudomonas aeruginosa strain K34-7 plasmid pK34-7-1, complete sequence | 4440 | 30.1 |
| NZ_CP029745.1 | Pseudomonas aeruginosa strain AR_0110 chromosome, complete genome | 6799785 | 66.0 |
| NZ_CP029772.1 | Pseudomonas sp. R2A2 chromosome, complete genome | 4559447 | 63.7 |
| NZ_CP029822.1 | Pseudomonas sp. QZS01 chromosome, complete genome | 3337477 | 38.1 |
| NZ_CP029983.1 | Pseudomonas sp. LG1E9 chromosome, complete genome | 6243355 | 60.2 |
| NZ_CP030327.1 | Pseudomonas aeruginosa strain AR_458 chromosome, complete genome | 6685102 | 66.2 |
| NZ_CP030328.1 | Pseudomonas aeruginosa strain AR_455 chromosome, complete genome | 6540996 | 65.9 |
| NZ_CP030351.1 | Pseudomonas aeruginosa strain AR_460 chromosome, complete genome | 6303875 | 66.6 |
| NZ_CP030750.1 | Pseudomonas putida strain NX-1 chromosome, complete genome | 5848357 | 62.9 |
| NZ_CP030861.1 | Pseudomonas aeruginosa strain HS9 chromosome, complete genome | 6876988 | 66.2 |
| NZ_CP030910.1 | Pseudomonas aeruginosa strain Y31 chromosome, complete genome | 6831076 | 66.2 |
| NZ_CP030911.1 | Pseudomonas aeruginosa strain Y71 chromosome, complete genome | 6940949 | 66.0 |
| NZ_CP030912.1 | Pseudomonas aeruginosa strain Y82 chromosome, complete genome | 7106857 | 65.8 |
| NZ_CP030913.1 | Pseudomonas aeruginosa strain Y89 chromosome, complete genome | 6868832 | 66.1 |
| NZ_CP030914.1 | Pseudomonas aeruginosa strain Y89 plasmid pY89, complete sequence | 85842 | 60.1 |
| NZ_CP031146.1 | Pseudomonas plecoglossicida strain XSDHY-P chromosome, complete genome | 5525520 | 62.7 |
| NZ_CP031396.1 | Pseudomonas protegens strain FD6 chromosome, complete genome | 6667995 | 62.5 |
| NZ_CP031449.2 | Pseudomonas aeruginosa strain 97 chromosome, complete genome | 6925889 | 65.9 |
| NZ_CP031450.1 | Pseudomonas fluorescens strain SIK_W1 chromosome, complete genome | 6791087 | 61.7 |
| NZ_CP031606.1 | Pseudomonas sp. phDV1 chromosome, complete genome | 4727682 | 62.3 |
| NZ_CP031641.1 | Pseudomonas parafulva strain JBCS1880 chromosome, complete genome | 5208480 | 63.4 |
| NZ_CP031648.1 | Pseudomonas fluorescens strain Pf275 chromosome, complete genome | 6610362 | 60.9 |
| NZ_CP031659.1 | Pseudomonas aeruginosa strain PABL012 chromosome, complete genome | 6546467 | 66.3 |
| NZ_CP031660.1 | Pseudomonas aeruginosa strain PABL017 chromosome, complete genome | 6503460 | 66.3 |
| NZ_CP031677.1 | Pseudomonas aeruginosa strain E80 chromosome, complete genome | 6819140 | 66.1 |
| NZ_CP032126.1 | Pseudomonas aeruginosa strain PAO1161 chromosome, complete genome | 6383803 | 66.4 |
| NZ_CP032256.1 | Pseudomonas aeruginosa strain AR_0111 plasmid unnamed, complete sequence | 129422 | 57.5 |
| NZ_CP032257.1 | Pseudomonas aeruginosa strain AR_0111 chromosome, complete genome | 6946231 | 66.0 |
| NZ_CP032311.1 | Pseudomonas sp. DG56-2 chromosome, complete genome | 5820432 | 58.7 |
| NZ_CP032352.1 | Pseudomonas protegens strain pf5-k3 chromosome, complete genome | 7085242 | 63.3 |
| NZ_CP032353.1 | Pseudomonas protegens strain pf5-k2 chromosome, complete genome | 7080416 | 63.3 |
| NZ_CP032358.1 | Pseudomonas protegens strain pf5 chromosome, complete genome | 7087572 | 63.3 |
| NZ_CP032419.1 | Pseudomonas sp. K2W31S-8 chromosome, complete genome | 4949615 | 64.5 |
| NZ_CP032552.1 | Pseudomonas aeruginosa strain PA34 chromosome, complete genome | 6810079 | 66.1 |
| NZ_CP032569.1 | Pseudomonas aeruginosa strain BA7823 chromosome, complete genome | 6876113 | 65.8 |
| NZ_CP032615.1 | Pseudomonas sp. DY-1 plasmid p.1, complete sequence | 26350 | 56.8 |
| NZ_CP032616.1 | Pseudomonas sp. DY-1 chromosome, complete genome | 5886398 | 63.0 |
| NZ_CP032618.1 | Pseudomonas fluorescens strain PF08 chromosome, complete genome | 6030573 | 60.7 |
| NZ_CP032631.1 | Pseudomonas syringae pv. actinidiae str. Shaanxi_M228 chromosome, complete genome | 6674594 | 58.3 |
| NZ_CP032632.1 | Pseudomonas syringae pv. actinidiae str. Shaanxi_M228 plasmid pM228, complete sequence | 72748 | 56.2 |
| NZ_CP032677.1 | Pseudomonas sp. Leaf58 chromosome, complete genome | 5431558 | 62.3 |
| NZ_CP032678.1 | Pseudomonas sp. Leaf58 plasmid pBASL58, complete sequence | 904163 | 55.4 |
| NZ_CP032761.1 | Pseudomonas aeruginosa strain 268 chromosome, complete genome | 7030474 | 65.9 |
| NZ_CP032870.1 | Pseudomonas syringae pv. actinidiae strain P155 plasmid pLKQG722, complete sequence | 77771 | 56.0 |
| NZ_CP032871.1 | Pseudomonas syringae pv. actinidiae strain P155 chromosome, complete genome | 6529859 | 58.4 |
| NZ_CP033104.1 | Pseudomonas sp. LTGT-11-2Z chromosome, complete genome | 6073276 | 61.7 |
| NZ_CP033105.1 | Pseudomonas sp. LTJR-52 chromosome, complete genome | 5539211 | 55.2 |
| NZ_CP033116.1 | Pseudomonas pelagia strain Kongs-67 chromosome, complete genome | 4550643 | 57.5 |
| NZ_CP033439.1 | Pseudomonas aeruginosa strain SP4528 chromosome, complete genome | 6877287 | 65.8 |
| NZ_CP033684.1 | Pseudomonas aeruginosa strain H26027 chromosome, complete genome | 7079598 | 66.1 |
| NZ_CP033685.1 | Pseudomonas aeruginosa strain H26023 chromosome, complete genome | 6729216 | 66.2 |
| NZ_CP033686.1 | Pseudomonas aeruginosa strain H25883 chromosome, complete genome | 6706800 | 66.2 |
| NZ_CP033771.1 | Pseudomonas aeruginosa strain FDAARGOS_532 chromosome, complete genome | 6928914 | 66.1 |
| NZ_CP033772.1 | Pseudomonas aeruginosa strain FDAARGOS_532 plasmid unnamed1 | 1249 | 51.7 |
| NZ_CP033773.1 | Pseudomonas aeruginosa strain FDAARGOS_532 plasmid unnamed2 | 1089 | 46.4 |
| NZ_CP033832.1 | Pseudomonas aeruginosa strain FDAARGOS_505 chromosome, complete genome | 7029824 | 65.9 |
| NZ_CP033833.1 | Pseudomonas aeruginosa strain FDAARGOS_571 chromosome, complete genome | 6999770 | 65.9 |
| NZ_CP033834.1 | Pseudomonas aeruginosa strain FDAARGOS_570 plasmid unnamed, complete sequence | 36032 | 61.3 |
| NZ_CP033835.1 | Pseudomonas aeruginosa strain FDAARGOS_570 chromosome, complete genome | 7119105 | 65.9 |
| NZ_CP033843.1 | Pseudomonas aeruginosa strain FDAARGOS_501 chromosome, complete genome | 6865838 | 66.0 |
| NZ_CP034078.1 | Pseudomonas syringae pv. pisi str. PP1 chromosome, complete genome | 5883416 | 58.9 |
| NZ_CP034079.1 | Pseudomonas syringae pv. pisi str. PP1 plasmid pPP1-1, complete sequence | 62150 | 56.3 |
| NZ_CP034080.1 | Pseudomonas syringae pv. pisi str. PP1 plasmid pPP1-2, complete sequence | 54993 | 55.7 |
| NZ_CP034081.1 | Pseudomonas syringae pv. pisi str. PP1 plasmid pPP1-3, complete sequence | 39003 | 51.7 |
| NZ_CP034337.1 | Pseudomonas entomophila strain 2014 chromosome, complete genome | 5686346 | 64.0 |
| NZ_CP034338.1 | Pseudomonas entomophila strain 1257 chromosome, complete genome | 6049604 | 63.8 |
| NZ_CP034354.1 | Pseudomonas aeruginosa strain IMP-13 chromosome, complete genome | 7047704 | 65.8 |
| NZ_CP034355.1 | Pseudomonas aeruginosa strain IMP-13 plasmid pPYO_TB, complete sequence | 130306 | 57.7 |
| NZ_CP034369.1 | Pseudomonas aeruginosa strain SP4371 chromosome, complete genome | 6937609 | 65.8 |
| NZ_CP034409.1 | Pseudomonas aeruginosa strain SP4527 chromosome, complete genome | 7005215 | 65.8 |
| NZ_CP034425.1 | Pseudomonas libanensis strain DMSP-1 chromosome, complete genome | 6282445 | 60.0 |
| NZ_CP034429.1 | Pseudomonas aeruginosa strain GIMC5015:PAKB6, complete sequence | 6258491 | 66.5 |
| NZ_CP034434.1 | Pseudomonas aeruginosa strain SP2230 chromosome, complete genome | 6976603 | 65.7 |
| NZ_CP034435.1 | Pseudomonas aeruginosa strain B14130 chromosome, complete genome | 6759594 | 65.9 |
| NZ_CP034436.1 | Pseudomonas aeruginosa strain B17932 chromosome, complete genome | 6744658 | 65.9 |
| NZ_CP034725.1 | Pseudomonas brassicacearum strain 3Re2-7 chromosome, complete genome | 6738544 | 60.8 |
| NZ_CP034780.1 | Pseudomonas sp. MPC6 plasmid pMPC6-4K, complete sequence | 4268 | 53.9 |
| NZ_CP034781.1 | Pseudomonas sp. MPC6 plasmid pMPC6-47K, complete sequence | 47564 | 54.7 |
| NZ_CP034782.1 | Pseudomonas sp. MPC6 plasmid pMPC6-328K, complete sequence | 328214 | 55.4 |
| NZ_CP034783.1 | Pseudomonas sp. MPC6 chromosome, complete genome | 6841168 | 60.0 |
| NZ_CP035088.1 | Pseudomonas sp. 11K1 chromosome, complete genome | 6682832 | 60.4 |
| NZ_CP035089.1 | Pseudomonas sp. 11K1 plasmid p11K1, complete sequence | 22045 | 53.2 |
| NZ_CP035739.1 | Pseudomonas aeruginosa strain 1334/14 chromosome, complete genome | 6902135 | 65.8 |
| NZ_CP035952.1 | Pseudomonas sp. SNU WT1 chromosome, complete genome | 5685196 | 61.8 |
| NZ_CP037925.1 | Pseudomonas aeruginosa strain AES1M chromosome, complete genome | 6373139 | 66.5 |
| NZ_CP037926.1 | Pseudomonas aeruginosa strain AES1R chromosome, complete genome | 6373893 | 66.5 |
| NZ_CP038001.1 | Pseudomonas sp. SXM-1 chromosome, complete genome | 7226716 | 60.8 |
| NZ_CP038207.1 | Pseudomonas sp. S150 chromosome, complete genome | 6304843 | 60.2 |
| NZ_CP038438.1 | Pseudomonas fluorescens strain LBUM677 chromosome, complete genome | 6140320 | 60.3 |
| NZ_CP039293.1 | Pseudomonas aeruginosa strain PABL048 chromosome, complete genome | 6879622 | 66.0 |
| NZ_CP039294.1 | Pseudomonas aeruginosa strain PABL048 plasmid pPABL048, complete sequence | 414954 | 56.6 |
| NZ_CP039371.1 | Pseudomonas putida strain 1290 chromosome, complete genome | 6495886 | 63.1 |
| NZ_CP039372.1 | Pseudomonas putida strain 1290 plasmid pPp1290, complete sequence | 114265 | 54.6 |
| NZ_CP039631.2 | Pseudomonas veronii strain Pvy chromosome, complete genome | 7110470 | 60.7 |
| NZ_CP039632.2 | Pseudomonas veronii strain Pvy plasmid unnamed, complete sequence | 194526 | 59.7 |
| NZ_CP039988.1 | Pseudomonas aeruginosa strain T2436 chromosome, complete genome | 6782092 | 66.1 |
| NZ_CP039989.1 | Pseudomonas aeruginosa strain T2436 plasmid pBT2436, complete sequence | 422811 | 56.9 |
| NZ_CP039990.1 | Pseudomonas aeruginosa strain T2101 chromosome, complete genome | 6573638 | 66.2 |
| NZ_CP039991.1 | Pseudomonas aeruginosa strain T2101 plasmid pBT2101, complete sequence | 439744 | 57.0 |
| NZ_CP040126.1 | Pseudomonas aeruginosa strain PA298 plasmid pBM908, complete sequence | 395774 | 56.9 |
| NZ_CP040127.1 | Pseudomonas aeruginosa strain PA298 chromosome, complete genome | 6539894 | 66.2 |
| NZ_CP040324.1 | Pseudomonas monteilii strain TCU-CK1 chromosome, complete genome | 5901796 | 61.9 |
| NZ_CP040459.1 | Pseudomonas stutzeri strain PheN2 plasmid unnamed, complete sequence | 122879 | 57.4 |
| NZ_CP040460.1 | Pseudomonas stutzeri strain PheN2 chromosome, complete genome | 3936412 | 64.4 |
| NZ_CP040684.1 | Pseudomonas aeruginosa strain C79 chromosome, complete genome | 6756154 | 66.1 |
| NZ_CP040685.1 | Pseudomonas aeruginosa strain C79 plasmid p1, complete sequence | 40180 | 58.1 |
| NZ_CP040930.1 | Pseudomonas sp. SWI7 chromosome, complete genome | 4931431 | 61.6 |
| NZ_CP041008.1 | Pseudomonas aeruginosa strain FDAARGOS_767 chromosome, complete genome | 6263676 | 66.5 |
| NZ_CP041013.1 | Pseudomonas aeruginosa strain FDAARGOS_610 chromosome, complete genome | 6792215 | 66.2 |
| NZ_CP041189.1 | Pseudomonas sp. NIBRBAC000502773 chromosome, complete genome | 6712511 | 61.3 |
| NZ_CP041236.1 | Pseudomonas azotoformans strain P45A chromosome, complete genome | 6884339 | 61.1 |
| NZ_CP041354.1 | Pseudomonas aeruginosa strain AZPAE15042 chromosome, complete genome | 6527298 | 66.5 |
| NZ_CP041355.1 | Pseudomonas aeruginosa strain AZPAE15042 plasmid pIHMA87, complete sequence | 185168 | 59.3 |
| NZ_CP041753.1 | Pseudomonas sp. ATCC 43928 chromosome, complete genome | 6466929 | 59.4 |
| NZ_CP041754.1 | Pseudomonas sp. KBS0707 chromosome, complete genome | 5936802 | 58.0 |
| NZ_CP041755.1 | Pseudomonas sp. KBS0707 plasmid unnamed1, complete sequence | 131974 | 54.1 |
| NZ_CP041756.1 | Pseudomonas sp. KBS0707 plasmid unnamed2, complete sequence | 41171 | 54.8 |
| NZ_CP041771.1 | Pseudomonas aeruginosa strain A681 chromosome, complete genome | 6616247 | 66.2 |
| NZ_CP041772.1 | Pseudomonas aeruginosa strain 243931 chromosome, complete genome | 6514208 | 66.3 |
| NZ_CP041773.1 | Pseudomonas aeruginosa strain 1705-19119 chromosome, complete genome | 6714255 | 66.0 |
| NZ_CP041774.1 | Pseudomonas aeruginosa strain 60503 chromosome, complete genome | 6809062 | 66.1 |
| NZ_CP041933.1 | Pseudomonas sp. BJP69 chromosome, complete genome | 5597068 | 62.2 |
| NZ_CP041945.1 | Pseudomonas aeruginosa strain ST773 chromosome, complete genome | 6835731 | 66.1 |
| NZ_CP042180.1 | Pseudomonas sp. KBS0802 chromosome, complete genome | 6200328 | 61.5 |
| NZ_CP042181.1 | Pseudomonas sp. KBS0802 plasmid unnamed, complete sequence | 116559 | 58.6 |
| NZ_CP042268.1 | Pseudomonas aeruginosa strain HOU1 plasmid pHOU1-1, complete sequence | 167069 | 64.9 |
| NZ_CP042269.1 | Pseudomonas aeruginosa strain HOU1 chromosome, complete genome | 6123373 | 66.4 |
| NZ_CP042804.1 | Pseudomonas amygdali pv. tabaci str. ATCC 11528 chromosome, complete genome | 6133558 | 58.0 |
| NZ_CP042805.1 | Pseudomonas amygdali pv. tabaci str. ATCC 11528 plasmid pTab1, complete sequence | 68162 | 55.5 |
| NZ_CP043060.1 | Pseudomonas sp. J380 chromosome, complete genome | 6261650 | 59.7 |
| NZ_CP043179.1 | Pseudomonas protegens strain SN15-2 chromosome, complete genome | 7075587 | 63.3 |
| NZ_CP043311.1 | Pseudomonas sp. PE08 chromosome, complete genome | 6056953 | 64.2 |
| NZ_CP043320.1 | Pseudomonas sp. C27(2019) chromosome, complete genome | 3217366 | 48.6 |
| NZ_CP043328.1 | Pseudomonas aeruginosa strain CCUG 51971 chromosome, complete genome | 7012798 | 66.1 |
| NZ_CP043626.1 | Pseudomonas denitrificans (nomen rejiciendum) strain BG1 chromosome, complete genome | 6880508 | 65.3 |
| NZ_CP043835.1 | Pseudomonas putida strain JYR-1 chromosome, complete genome | 5413503 | 62.5 |
| NZ_CP044006.1 | Pseudomonas aeruginosa strain E90 chromosome, complete genome | 6813135 | 66.2 |
| NZ_CP044072.1 | Pseudomonas oryzihabitans strain FDAARGOS_657 plasmid unnamed1, complete sequence | 132067 | 58.7 |
| NZ_CP044073.1 | Pseudomonas oryzihabitans strain FDAARGOS_657 plasmid unnamed2, complete sequence | 183433 | 60.9 |
| NZ_CP044074.1 | Pseudomonas oryzihabitans strain FDAARGOS_657 chromosome, complete genome | 4949413 | 66.2 |
| NZ_CP044084.1 | Pseudomonas luteola strain FDAARGOS_637 plasmid unnamed1, complete sequence | 585976 | 54.9 |
| NZ_CP044085.1 | Pseudomonas luteola strain FDAARGOS_637 chromosome 2, complete sequence | 919958 | 54.0 |
| NZ_CP044086.1 | Pseudomonas luteola strain FDAARGOS_637 chromosome 1, complete sequence | 4273365 | 55.8 |
| NZ_CP044087.1 | Pseudomonas luteola strain FDAARGOS_637 plasmid unnamed2 | 120674 | 56.5 |
| NZ_CP044088.1 | Pseudomonas luteola strain FDAARGOS_637 plasmid unnamed3 | 1410 | 47.1 |
| NZ_CP045118.1 | Pseudomonas sp. SCB32 chromosome, complete genome | 6311241 | 64.6 |
| NZ_CP045221.1 | Pseudomonas chlororaphis subsp. aurantiaca strain ARS 38 chromosome, complete genome | 6615046 | 63.2 |
| NZ_CP045254.1 | Pseudomonas sp. DTU12.1 chromosome, complete genome | 5943629 | 60.7 |
| NZ_CP045349.1 | Pseudomonas sp. THAF187a chromosome, complete genome | 5298761 | 64.8 |
| NZ_CP045359.1 | Pseudomonas sp. THAF42 chromosome, complete genome | 5298227 | 64.8 |
| NZ_CP045416.1 | Pseudomonas sp. THAF7b chromosome, complete genome | 4522538 | 63.3 |
| NZ_CP045701.1 | Pseudomonas brassicacearum strain S-1 chromosome, complete genome | 6575053 | 60.8 |
| NZ_CP045739.1 | Pseudomonas aeruginosa strain AG1 chromosome, complete genome | 7190208 | 65.7 |
| NZ_CP045767.1 | Pseudomonas sp. CFSAN084952 chromosome, complete genome | 6326501 | 59.9 |
| NZ_CP045768.1 | Pseudomonas aeruginosa strain CFSAN084950 chromosome, complete genome | 6441924 | 66.3 |
| NZ_CP045799.1 | Pseudomonas syringae USA011 chromosome, complete genome | 6024796 | 59.2 |
| NZ_CP045800.1 | Pseudomonas syringae USA011 plasmid pUSA011-1, complete sequence | 44089 | 54.1 |
| NZ_CP045801.1 | Pseudomonas syringae USA011 plasmid pUSA011-2, complete sequence | 43372 | 54.0 |
| NZ_CP046035.1 | Pseudomonas coronafaciens pv. oryzae str. 1_6 chromosome, complete genome | 5662937 | 57.9 |
| NZ_CP046036.1 | Pseudomonas coronafaciens pv. oryzae str. 1_6 plasmid pPor1_6, complete sequence | 51521 | 54.6 |
| NZ_CP046060.1 | Pseudomonas aeruginosa strain 1811-18R001 chromosome, complete genome | 7343000 | 65.8 |
| NZ_CP046061.1 | Pseudomonas aeruginosa strain 1811-13R031 chromosome, complete genome | 7344079 | 65.8 |
| NZ_CP046069.1 | Pseudomonas aeruginosa strain KRP1 chromosome, complete genome | 6737396 | 66.3 |
| NZ_CP046538.1 | Pseudomonas stutzeri strain XL272 chromosome, complete genome | 3925175 | 64.7 |
| NZ_CP046621.1 | Pseudomonas alkylphenolica strain Neo chromosome, complete genome | 5612010 | 61.2 |
| NZ_CP046874.1 | Pseudomonas sp. S58 chromosome, complete genome | 6486667 | 61.1 |
| NZ_CP046902.1 | Pseudomonas stutzeri strain PM101005 chromosome, complete genome | 4682747 | 62.1 |
| NZ_CP046903.1 | Pseudomonas stutzeri strain PM101005 plasmid p1_PM101005, complete sequence | 265581 | 57.5 |
| NZ_CP047073.1 | Pseudomonas syringae pv. tomato strain delta X chromosome, complete genome | 6397114 | 58.4 |
| NZ_CP047260.1 | Pseudomonas syringae pv. maculicola str. ES4326 chromosome, complete genome | 6115755 | 58.7 |
| NZ_CP047261.1 | Pseudomonas syringae pv. maculicola str. ES4326 plasmid pPma4326F, complete sequence | 387260 | 52.7 |
| NZ_CP047262.1 | Pseudomonas syringae pv. maculicola str. ES4326 plasmid pPma4326A, complete sequence | 46546 | 55.1 |
| NZ_CP047263.1 | Pseudomonas syringae pv. maculicola str. ES4326 plasmid pPma4326B, complete sequence | 39848 | 55.4 |
| NZ_CP047264.1 | Pseudomonas syringae pv. maculicola str. ES4326 plasmid pPma4326E, complete sequence | 4217 | 56.1 |
| NZ_CP047265.1 | Pseudomonas asturiensis strain CC1524 chromosome, complete genome | 5803532 | 59.1 |
| NZ_CP047266.1 | Pseudomonas asturiensis strain CC1524 plasmid pCC1524, complete sequence | 108727 | 59.5 |
| NZ_CP047267.1 | Pseudomonas syringae UB303 chromosome, complete genome | 6141482 | 59.2 |
| NZ_CP047592.1 | Pseudomonas aeruginosa strain INP-43 chromosome, complete genome | 6335031 | 66.4 |
| NZ_CP047697.1 | Pseudomonas aeruginosa strain RD1-3 chromosome, complete genome | 6397159 | 66.4 |
| NZ_CP047698.1 | Pseudomonas knackmussii strain N1-2 chromosome, complete genome | 6095635 | 65.6 |
| NZ_HG322950.1 | Pseudomonas knackmussii B13 complete genome | 6162905 | 65.6 |
| NZ_HG916826.1 | Pseudomonas pseudoalcaligenes CECT 5344 complete genome | 4686340 | 62.3 |
| NZ_LK391695.1 | Pseudomonas pseudoalcaligenes genome assembly Ppseudo_Pac, chromosome : I | 4696984 | 62.3 |
| NZ_LN831024.1 | Pseudomonas aeruginosa genome assembly NCTC10332, chromosome : 1 | 6316979 | 66.5 |
| NZ_LN847264.1 | Pseudomonas sp. CCOS 191 genome assembly Pseudomonas sp. strain CCOS 191, chromosome : I | 6012947 | 64.2 |
| NZ_LN854573.1 | Pseudomonas sp. URMO17WK12:I11 genome assembly Shine, chromosome : 1 | 6374437 | 59.2 |
| NZ_LN865164.1 | Pseudomonas sp. URMO17WK12:I11 isolate Yellow genome assembly, chromosome: 1 | 4835345 | 61.7 |
| NZ_LN870292.1 | Pseudomonas aeruginosa DK1 genome assembly Pseudomonas aeruginosa DK1 substr. NH57388A, chromosome : I | 6212531 | 66.6 |
| NZ_LN871187.1 | Pseudomonas aeruginosa genome assembly PAO1OR, chromosome : I | 6276469 | 66.5 |
| NZ_LR130527.1 | Pseudomonas aeruginosa isolate paerg002 genome assembly, chromosome: 0 | 6451470 | 66.4 |
| NZ_LR130528.1 | Pseudomonas aeruginosa isolate paerg000 genome assembly, chromosome: 0 | 6493562 | 66.4 |
| NZ_LR130530.1 | Pseudomonas aeruginosa isolate paerg003 genome assembly, chromosome: 0 | 6433962 | 66.4 |
| NZ_LR130531.1 | Pseudomonas aeruginosa isolate paerg004 genome assembly, chromosome: 0 | 6452809 | 66.4 |
| NZ_LR130533.1 | Pseudomonas aeruginosa isolate paerg009 genome assembly, chromosome: 0 | 6941287 | 66.0 |
| NZ_LR130534.1 | Pseudomonas aeruginosa isolate paerg005 genome assembly, chromosome: 0 | 6931425 | 66.0 |
| NZ_LR130535.1 | Pseudomonas aeruginosa isolate paerg011 genome assembly, chromosome: 0 | 6434133 | 66.4 |
| NZ_LR130536.1 | Pseudomonas aeruginosa isolate paerg010 genome assembly, chromosome: 0 | 6433960 | 66.4 |
| NZ_LR130537.1 | Pseudomonas aeruginosa isolate paerg012 genome assembly, chromosome: 0 | 6434020 | 66.4 |
| NZ_LR130779.1 | Pseudomonas oleovorans strain T9AD genome assembly, chromosome: POT9AD | 5623977 | 64.5 |
| NZ_LR130780.1 | Pseudomonas oleovorans strain T9AD genome assembly, plasmid: POT9AD_p | 2777 | 61.8 |
| NZ_LR134290.1 | Pseudomonas mendocina strain NCTC10897 genome assembly, chromosome: 1 | 5192837 | 62.8 |
| NZ_LR134299.1 | Pseudomonas putida strain NCTC13186 genome assembly, chromosome: 1 | 6134366 | 61.5 |
| NZ_LR134300.1 | Pseudomonas fluorescens strain NCTC10783 genome assembly, chromosome: 1 | 6866429 | 65.9 |
| NZ_LR134308.1 | Pseudomonas aeruginosa strain NCTC11445 genome assembly, chromosome: 1 | 6766292 | 66.1 |
| NZ_LR134309.1 | Pseudomonas aeruginosa strain NCTC12903 genome assembly, chromosome: 1 | 6839985 | 66.1 |
| NZ_LR134318.1 | Pseudomonas fluorescens strain NCTC9428 genome assembly, chromosome: 1 | 6033966 | 59.0 |
| NZ_LR134319.1 | Pseudomonas stutzeri strain NCTC10450 genome assembly, chromosome: 1 | 4438731 | 64.1 |
| NZ_LR134330.1 | Pseudomonas aeruginosa strain NCTC13715 genome assembly, chromosome: 1 | 6765311 | 66.1 |
| NZ_LR134334.1 | Pseudomonas chlororaphis strain NCTC7357 genome assembly, chromosome: 1 | 7209930 | 62.5 |
| NZ_LR134342.1 | Pseudomonas aeruginosa strain NCTC10728 genome assembly, chromosome: 1 | 6363395 | 66.4 |
| NZ_LR134393.1 | Pseudomonas taetrolens strain NCTC8067 genome assembly, chromosome: 1 | 4967572 | 58.2 |
| NZ_LR134482.1 | Pseudomonas stutzeri strain NCTC10475 genome assembly, chromosome: 1 | 4426240 | 63.1 |
| NZ_LR215729.1 | Pseudomonas marincola strain YSy11 genome assembly, chromosome: PMYSY11 | 4814646 | 57.3 |
| NZ_LR590472.1 | Pseudomonas aeruginosa strain NCTC13620 genome assembly, chromosome: 1 | 6779267 | 66.2 |
| NZ_LR590473.1 | Pseudomonas aeruginosa strain NCTC13359 genome assembly, chromosome: 1 | 6791490 | 66.2 |
| NZ_LR590474.1 | Pseudomonas aeruginosa strain NCTC13618 genome assembly, chromosome: 1 | 6788588 | 66.2 |
| NZ_LR590482.1 | Pseudomonas synxantha strain NCTC10696 genome assembly, chromosome: 1 | 6841780 | 59.6 |
| NZ_LR657304.1 | Pseudomonas aeruginosa PAK chromosome 1 | 6395872 | 66.4 |
| NZ_LS483370.1 | Pseudomonas taetrolens strain NCTC10697 genome assembly, chromosome: 1 | 4843871 | 58.3 |
| NZ_LS483372.1 | Pseudomonas fluorescens strain NCTC10038 genome assembly, chromosome: 1 | 6515171 | 60.0 |
| NZ_LS483433.1 | Pseudomonas mucidolens strain NCTC8068 genome assembly, chromosome: 1 | 5853060 | 59.1 |
| NZ_LS998783.1 | Pseudomonas aeruginosa isolate 1 genome assembly, chromosome: 1 | 7207057 | 65.6 |
| NZ_LS998784.1 | Pseudomonas aeruginosa isolate 1 genome assembly, plasmid: 2 | 24853 | 57.1 |
| NZ_LS999205.1 | Pseudomonas protegens CHA0 genome assembly, chromosome: 1 | 6868303 | 63.4 |
| NZ_LT222313.1 | Pseudomonas cerasi isolate Sour cherry (Prunus cerasus) symptomatic leaf plasmid p58T1, complete sequence | 101345 | 56.0 |
| NZ_LT222314.1 | Pseudomonas cerasi isolate Sour cherry (Prunus cerasus) symptomatic leaf plasmid p58T2, complete sequence | 70584 | 55.2 |
| NZ_LT222315.1 | Pseudomonas cerasi isolate Sour cherry (Prunus cerasus) symptomatic leaf plasmid p58T3 | 143614 | 55.6 |
| NZ_LT222316.1 | Pseudomonas cerasi isolate Sour cherry (Prunus cerasus) symptomatic leaf plasmid p58T4 | 64870 | 56.2 |
| NZ_LT222317.1 | Pseudomonas cerasi isolate Sour cherry (Prunus cerasus) symptomatic leaf plasmid p58T5, complete sequence | 61794 | 53.8 |
| NZ_LT222318.1 | Pseudomonas cerasi isolate Sour cherry (Prunus cerasus) symptomatic leaf plasmid p58T6 | 11314 | 55.1 |
| NZ_LT222319.1 | Pseudomonas cerasi isolate Sour cherry (Prunus cerasus) symptomatic leaf chromosome 1, complete sequence | 5891735 | 59.0 |
| NZ_LT608330.1 | Pseudomonas aeruginosa isolate PA14Or_reads genome assembly, chromosome: PA14OR | 6541482 | 66.3 |
| NZ_LT673656.1 | Pseudomonas aeruginosa isolate PcyII-10 genome assembly, chromosome: PcyII-10 | 6288645 | 66.5 |
| NZ_LT855380.1 | Pseudomonas viridiflava strain CFBP 1590 genome assembly, chromosome: I | 6093513 | 59.2 |
| NZ_LT883143.1 | Pseudomonas aeruginosa C-NN2 isolate early isolate NN2 (clone C) genome assembly, chromosome: I | 6902967 | 66.1 |
| NZ_LT962480.1 | Pseudomonas syringae pv. syringae isolate CFBP4215 genome assembly, chromosome: 1 | 6035297 | 59.3 |
| NZ_LT962481.1 | Pseudomonas syringae pv. syringae isolate CFBP2118 genome assembly, chromosome: 1 | 6031285 | 59.3 |
| NZ_LT963391.1 | Pseudomonas syringae pv. cerasicola isolate CFBP6109 genome assembly, chromosome: 1 | 6015874 | 58.2 |
| NZ_LT963392.1 | Pseudomonas syringae pv. cerasicola isolate CFBP6109 genome assembly, plasmid: PP1 | 118774 | 55.5 |
| NZ_LT963393.1 | Pseudomonas syringae pv. cerasicola isolate CFBP6109 genome assembly, plasmid: PP2 | 86328 | 56.3 |
| NZ_LT963394.1 | Pseudomonas syringae pv. cerasicola isolate CFBP6109 genome assembly, plasmid: PP3 | 111777 | 56.6 |
| NZ_LT963395.1 | Pseudomonas cerasi isolate PL963 genome assembly, chromosome: 1 | 5889419 | 59.0 |
| NZ_LT963396.1 | Pseudomonas cerasi isolate PL963 genome assembly, plasmid: PP1 | 127474 | 54.8 |
| NZ_LT963397.1 | Pseudomonas cerasi isolate PL963 genome assembly, plasmid: PP2 | 144075 | 55.6 |
| NZ_LT963398.1 | Pseudomonas cerasi isolate PL963 genome assembly, plasmid: PP3 | 81323 | 55.1 |
| NZ_LT963399.1 | Pseudomonas cerasi isolate PL963 genome assembly, plasmid: PP4 | 70589 | 55.2 |
| NZ_LT963400.1 | Pseudomonas cerasi isolate PL963 genome assembly, plasmid: PP5 | 48121 | 55.0 |
| NZ_LT963401.1 | Pseudomonas cerasi isolate PL963 genome assembly, plasmid: PP6 | 18360 | 55.3 |
| NZ_LT963402.1 | Pseudomonas syringae pv. avii isolate CFBP3846 genome assembly, chromosome: 1 | 6120158 | 58.7 |
| NZ_LT963403.1 | Pseudomonas syringae pv. avii isolate CFBP3846 genome assembly, plasmid: PP1 | 43975 | 56.0 |
| NZ_LT963404.1 | Pseudomonas syringae pv. avii isolate CFBP3846 genome assembly, plasmid: PP2 | 109843 | 55.7 |
| NZ_LT963405.1 | Pseudomonas syringae pv. avii isolate CFBP3846 genome assembly, plasmid: PP3 | 108842 | 55.8 |
| NZ_LT963406.1 | Pseudomonas syringae pv. avii isolate CFBP3846 genome assembly, plasmid: PP4 | 77492 | 55.9 |
| NZ_LT963407.1 | Pseudomonas syringae pv. avii isolate CFBP3846 genome assembly, plasmid: PP5 | 41285 | 54.5 |
| NZ_LT963408.1 | Pseudomonas syringae group genomosp. 3 isolate CFBP6411 genome assembly, chromosome: I | 6363674 | 58.6 |
| NZ_LT963409.1 | Pseudomonas syringae isolate CFBP3840 genome assembly, chromosome: 1 | 6013125 | 58.2 |
| NZ_LT963410.1 | Pseudomonas syringae isolate CFBP3840 genome assembly, plasmid: PP1 | 110420 | 54.7 |
| NZ_LT963411.1 | Pseudomonas syringae isolate CFBP3840 genome assembly, plasmid: PP2 | 90464 | 55.0 |
| NZ_LT963412.1 | Pseudomonas syringae isolate CFBP3840 genome assembly, plasmid: PP3 | 86369 | 58.3 |
| NZ_LT963413.1 | Pseudomonas syringae isolate CFBP3840 genome assembly, plasmid: PP4 | 78348 | 55.2 |
| NZ_LT969519.1 | Pseudomonas aeruginosa isolate RW109 genome assembly, plasmid: RW109 plasmid 1 | 555265 | 58.1 |
| NZ_LT969520.1 | Pseudomonas aeruginosa isolate RW109 genome assembly, chromosome: Main_chromosome | 7049347 | 65.8 |
| NZ_LT969521.1 | Pseudomonas aeruginosa isolate RW109 genome assembly, plasmid: RW109 plasmid 2 | 151612 | 57.3 |
