# Supplementary material for: ICEs Are the Main Reservoirs of the Ciprofloxacin-Modifying crpP Gene in Pseudomonas aeruginosa
Source: Genes (Basel). 2020 Aug 4;11(8):889. doi: 10.3390/genes11080889 (PMC7463715; doi:10.3390/genes11080889)
Supplement: Supplementary file 1 [file genes-11-00889-s001.zip › Table_S4.docx]

**Table S4**. General features of the *crpP*-carrying ICEs identified in this study.

| **Name** | **Species** | **Strain** | **MLST** | **Other ARGs** | **Bacteriocins** | **CRISPR-Cas** | **ICE_location** | **ICE Family** | **%GC of ICE** | **Sequence Length** |
| --- | --- | --- | --- | --- | --- | --- | --- | --- | --- | --- |
| NC_008463.1 | Pseudomonas aeruginosa | UCBPP-PA14 | 253 | NA | Pyocin S5 | NA | 5251438..5359437 | MPFG | 59.7 | 108000 |
| NC_009656.1 | Pseudomonas aeruginosa | PA7 | 1195 | NA | NA | NA | 4573758..4659356 | MPFG | 60.5 | 85599 |
| NC_018080.1 | Pseudomonas aeruginosa | DK2 | 386 | NA | NA | NA | 5097748..5188381 | MPFG | 59.3 | 90634 |
| NC_020912.1 | Pseudomonas aeruginosa | B136-33 | 1024 | NA | NA | NA | 5129453..5234036 | MPFG | 61.5 | 104584 |
| NC_021577.1 | Pseudomonas aeruginosa | RP73 | 198 | NA | NA | NA | 1023533..1126247 | MPFG | 60.9 | 102715 |
| NC_022806.1 | Pseudomonas aeruginosa | PA1R | NA | NA | NA | NA | 5021959..5124759 | MPFG | 60.4 | 102801 |
| NC_022808.2 | Pseudomonas aeruginosa | PA1 | 782 | NA | NA | NA | 4518241..4621030 | MPFG | 60.4 | 102790 |
| NC_023019.1 | Pseudomonas aeruginosa | MTB-1 | 2689 | NA | NA | NA | 5252992..5341025 | MPFG | 58.7 | 88034 |
| NC_023149.1 | Pseudomonas aeruginosa | SCV20265 | 299 | NA | NA | NA | 4723738..4816893 | MPFG | 60.4 | 93156 |
| NZ_AP014651.1 | Pseudomonas aeruginosa | NCGM257 | 357 | NA | NA | NA | 5699825..5782875 | MPFG | 60.7 | 83051 |
| NZ_AP014839.1 | Pseudomonas aeruginosa | 8380 | 2619 | NA | NA | NA | 5301494..5407295 | MPFG | 60.9 | 105802 |
| NZ_AP017302.1 | Pseudomonas aeruginosa | IOMTU 133 | 1047 | NA | NA | NA | 5541198..5641750 | MPFG | 56.6 | 100553 |
| NZ_CP007147.1 | Pseudomonas aeruginosa | YL84 | NA | NA | NA | NA | 2684203..2769071 | MPFG | 59.7 | 84869 |
| NZ_CP008856.2 | Pseudomonas aeruginosa | F23197 | 1295 | NA | NA | NA | 5221858..5338071 | MPFG | 59.5 | 116214 |
| NZ_CP008857.1 | Pseudomonas aeruginosa | F30658 | 111 | NA | NA | NA | 3269423..3355117 | MPFG | 60.7 | 85695 |
| NZ_CP008858.2 | Pseudomonas aeruginosa | F63912 | 198 | NA | NA | NA | 5314589..5418165 | MPFG | 60.9 | 103577 |
| NZ_CP008860.2 | Pseudomonas aeruginosa | H27930 | 389 | NA | NA | NA | 5258436..5361673 | MPFG | 60.9 | 103238 |
| NZ_CP008861.1 | Pseudomonas aeruginosa | H47921 | 1105 | NA | NA | NA | 5739208..5820841 | MPFG | 60.3 | 81634 |
| NZ_CP008862.2 | Pseudomonas aeruginosa | M1608 | 253 | NA | Pyocin S5 | NA | 1207510..1325095 | MPFG | 59.0 | 117586 |
| NZ_CP008863.1 | Pseudomonas aeruginosa | M37351 | 253 | NA | Pyocin S5 | NA | 4759452..4877038 | MPFG | 59.0 | 117587 |
| NZ_CP008865.2 | Pseudomonas aeruginosa | S86968 | 155 | NA | NA | NA | 5573693..5659002 | NA | 61.2 | 85310 |
| NZ_CP008865.2 | Pseudomonas aeruginosa | S86968 | 155 | NA | NA | NA | 4715709..4827366 | MPFG | 59.4 | 111658 |
| NZ_CP008866.2 | Pseudomonas aeruginosa | T38079 | 155 | NA | NA | NA | 5456109..5543956 | MPFG | 60.3 | 87848 |
| NZ_CP008869.2 | Pseudomonas aeruginosa | W16407 | 244 | NA | NA | NA | 5440677..5533836 | MPFG | 60.2 | 93160 |
| NZ_CP008871.2 | Pseudomonas aeruginosa | W45909 | 27 | NA | NA | NA | 4687981..4791112 | MPFG | 59.4 | 103132 |
| NZ_CP008873.1 | Pseudomonas aeruginosa | F9670 | 155 | NA | NA | NA | 2635482..2723321 | MPFG | 60.3 | 87840 |
| NZ_CP010555.1 | Pseudomonas aeruginosa | FRD1 | 111 | NA | NA | NA | 5486878..5572127 | MPFG | 60.1 | 85250 |
| NZ_CP011317.1 | Pseudomonas aeruginosa | Carb01 63 | 111 | NA | NA | NA | 6087900..6172252 | MPFG | 60.4 | 84353 |
| NZ_CP011857.1 | Pseudomonas aeruginosa | ATCC 27853 | 155 | NA | NA | NA | 5504320..5607155 | MPFG | 60.6 | 102836 |
| NZ_CP012679.1 | Pseudomonas aeruginosa | PA1RG | 782 | NA | NA | NA | 4518241..4621031 | MPFG | 60.4 | 102791 |
| NZ_CP013245.1 | Pseudomonas aeruginosa | VA-134 | 3090 | NA | NA | CAS-TypeIC | 3408411..3521196 | MPFG | 60.9 | 112786 |
| NZ_CP013993.1 | Pseudomonas aeruginosa | DHS01 | 395 | NA | NA | NA | 5007879..5102356 | MPFG | 60.3 | 94478 |
| NZ_CP014866.1 | Pseudomonas aeruginosa | PA_154197 | 550 | NA | NA | NA | 4527232..4622184 | MPFG | 59.1 | 94953 |
| NZ_CP014948.1 | Pseudomonas aeruginosa | N17-1 | 2362 | NA | NA | CAS-TypeIC | 4463713..4568937 | MPFG | 61.1 | 105225 |
| NZ_CP014999.1 | Pseudomonas aeruginosa | PA7790 | 277 | NA | Pyocin S5 | CAS-TypeIC | 5635771..5781235 | MPFG | 60.6 | 145465 |
| NZ_CP015001.1 | Pseudomonas aeruginosa | PA1088 | 277 | NA | Pyocin S5 | CAS-TypeIC | 5382752..5525578 | MPFG | 60.2 | 142827 |
| NZ_CP015002.1 | Pseudomonas aeruginosa | PA8281 | 277 | NA | Pyocin S5 | CAS-TypeIC | 5572504..5717969 | MPFG | 60.6 | 145466 |
| NZ_CP015117.1 | Pseudomonas aeruginosa | ATCC 27853 | 155 | NA | NA | NA | 2266451..2368432 | MPFG | 60.6 | 101982 |
| NZ_CP015377.1 | Pseudomonas aeruginosa | BAMCPA07-48 | 313 | NA | NA | NA | 6978181..66418 | NA | 60.9 | 109790 |
| NZ_CP016955.1 | Pseudomonas aeruginosa | RIVM-EMC2982 | 111 | NA | NA | NA | 1326863..1412944 | MPFG | 60.6 | 86082 |
| NZ_CP017099.1 | Pseudomonas aeruginosa | DN1 | 316 | NA | NA | NA | 5362518..5455159 | MPFG | 59.7 | 92642 |
| NZ_CP017293.1 | Pseudomonas aeruginosa | PA83 | 233 | NA | NA | NA | 4783902..4873008 | MPFG | 60.7 | 89107 |
| NZ_CP017353.1 | Pseudomonas aeruginosa | FA-HZ1 | 27 | NA | NA | NA | 599732..698016 | MPFG | 59.6 | 98285 |
| NZ_CP017969.1 | Pseudomonas aeruginosa | B10W | 308 | NA | NA | NA | 4492177..4610785 | MPFG | 58.4 | 118609 |
| NZ_CP020703.1 | Pseudomonas aeruginosa | PASGNDM345 | 308 | NA | NA | NA | 4867806..4986414 | MPFG | 58.4 | 118609 |
| NZ_CP020704.1 | Pseudomonas aeruginosa | PASGNDM699 | 308 | NA | NA | NA | 4986378..5078349 | MPFG | 61.0 | 91972 |
| NZ_CP021775.1 | Pseudomonas aeruginosa | Pa58 | 308 | NA | NA | NA | 5906126..5994299 | MPFG | 60.6 | 88174 |
| NZ_CP021999.1 | Pseudomonas aeruginosa | Pa84 | NA | NA | NA | NA | 5296771..5387683 | MPFG | 60.0 | 90913 |
| NZ_CP022001.1 | Pseudomonas aeruginosa | Pa1207 | 155 | NA | NA | NA | 1735211..1838035 | MPFG | 60.6 | 102825 |
| NZ_CP022002.1 | Pseudomonas aeruginosa | Pa1242 | 277 | NA | NA | NA | 1701475..1808580 | MPFG | 59.3 | 107106 |
| NZ_CP022478.1 | Pseudomonas aeruginosa | LW | 1182 | NA | NA | NA | 4249539..4333185 | MPFG | 61.2 | 83647 |
| NZ_CP022525.1 | Pseudomonas aeruginosa | Ocean-1175 | 316 | NA | NA | NA | 3108007..3190184 | MPFG | 60.5 | 82178 |
| NZ_CP022526.1 | Pseudomonas aeruginosa | Ocean-1155 | 316 | NA | NA | NA | 2424411..2506587 | MPFG | 60.5 | 82177 |
| NZ_CP023255.1 | Pseudomonas aeruginosa | CCUG 70744 | 395 | NA | NA | NA | 2762227..2856704 | MPFG | 60.3 | 94478 |
| NZ_CP023316.1 | Pseudomonas aeruginosa | PPF-1 | NA | NA | NA | NA | 5557899..5656637 | MPFG | 60.7 | 98739 |
| NZ_CP024477.1 | Pseudomonas aeruginosa | 12939 | 1420 | NA | NA | NA | 5256358..5348471 | MPFG | 60.2 | 92114 |
| NZ_CP024630.1 | Pseudomonas aeruginosa | PA59 | 260 | aac(6')-IIa, floR2, tet(G), sul1 | NA | NA | 5489910..5616157 | MPFG | 60.4 | 126248 |
| NZ_CP025229.1 | Pseudomonas sp. | AK6U | NA | NA | NA | NA | 6234155..6327996 | MPFG | 60.5 | 93842 |
| NZ_CP026680.1 | Pseudomonas aeruginosa | F5677 | 111 | NA | NA | NA | 5281383..5371346 | MPFG | 60.1 | 89964 |
| NZ_CP027165.1 | Pseudomonas aeruginosa | AR_0360 | 1712 | NA | NA | NA | 3947007..4046995 | MPFG | 61.2 | 99989 |
| NZ_CP027166.1 | Pseudomonas aeruginosa | AR_0357 | 235 | NA | NA | NA | 3795236..3886516 | MPFG | 59.4 | 91281 |
| NZ_CP027171.1 | Pseudomonas aeruginosa | AR_0354 | 235 | NA | NA | NA | 3284186..3383318 | MPFG | 60.2 | 99133 |
| NZ_CP027172.1 | Pseudomonas aeruginosa | AR_0353 | 308 | NA | NA | NA | 1359551..1445795 | MPFG | 60.5 | 86245 |
| NZ_CP027174.1 | Pseudomonas aeruginosa | AR_0230 | 233 | NA | NA | NA | 4178092..4265187 | NA | 60.5 | 87096 |
| NZ_CP027174.1 | Pseudomonas aeruginosa | AR_0230 | 233 | NA | NA | NA | 3429923..3519029 | MPFG | 60.7 | 89107 |
| NZ_CP028162.1 | Pseudomonas aeruginosa | MRSN12280 | NA | NA | NA | NA | 4865466..4953317 | MPFG | 60.3 | 87852 |
| NZ_CP028584.2 | Pseudomonas aeruginosa | WCHPA075019 | 277 | NA | NA | NA | 5567129..5674073 | MPFG | 59.3 | 106945 |
| NZ_CP028848.1 | Pseudomonas aeruginosa | IMP67 | 132 | NA | NA | NA | 5164794..5276868 | MPFG | 60.8 | 112075 |
| NZ_CP028849.1 | Pseudomonas aeruginosa | IMP68 | 132 | NA | NA | NA | 5164090..5276164 | MPFG | 60.8 | 112075 |
| NZ_CP028917.1 | Pseudomonas aeruginosa | JB2 | 296 | NA | NA | NA | 1699886..1790283 | MPFG | 60.1 | 90398 |
| NZ_CP028959.1 | Pseudomonas aeruginosa | IMP66 | 132 | NA | NA | NA | 5179005..5291079 | MPFG | 60.8 | 112075 |
| NZ_CP029088.1 | Pseudomonas aeruginosa | AR445 | 111 | NA | NA | NA | 5757738..5841631 | MPFG | 60.7 | 83894 |
| NZ_CP029089.1 | Pseudomonas aeruginosa | AR444 | 233 | NA | NA | NA | 6693279..6782711 | MPFG | 60.6 | 89433 |
| NZ_CP029090.1 | Pseudomonas aeruginosa | AR442 | 395 | NA | NA | NA | 4891592..4986069 | MPFG | 60.3 | 94478 |
| NZ_CP029097.1 | Pseudomonas aeruginosa | AR439 | 179 | NA | NA | NA | 1479588..1567435 | MPFG | 60.3 | 87848 |
| NZ_CP029605.1 | Pseudomonas aeruginosa | 24Pae112 | 235 | aac(6')-Il, blaOXA-2-like | NA | NA | 5752794..5854462 | MPFG | 60.5 | 101669 |
| NZ_CP029707.1 | Pseudomonas aeruginosa | K34-7 | 233 | NA | NA | NA | 4153870..4238811 | MPFG | 60.8 | 84942 |
| NZ_CP029745.1 | Pseudomonas aeruginosa | AR_0110 | 233 | NA | NA | NA | 4637689..4726795 | MPFG | 60.7 | 89107 |
| NZ_CP030328.1 | Pseudomonas aeruginosa | AR_455 | 298 | NA | NA | NA | 6407..111863 | MPFG | 60.5 | 105457 |
| NZ_CP030861.1 | Pseudomonas aeruginosa | HS9 | 27 | NA | NA | NA | 4536891..4635175 | MPFG | 59.6 | 98285 |
| NZ_CP030910.1 | Pseudomonas aeruginosa | Y31 | NA | NA | NA | NA | 1166409..1272911 | MPFG | 60.4 | 106503 |
| NZ_CP030911.1 | Pseudomonas aeruginosa | Y71 | 245 | NA | NA | NA | 5555242..5641369 | MPFG | 60.6 | 86128 |
| NZ_CP030912.1 | Pseudomonas aeruginosa | Y82 | 111 | NA | NA | NA | 4834779..4921886 | MPFG | 60.9 | 87108 |
| NZ_CP030912.1 | Pseudomonas aeruginosa | Y82 | 245 | NA | NA | NA | 5610710..5696287 | NA | 60.4 | 85578 |
| NZ_CP030913.1 | Pseudomonas aeruginosa | Y89 | NA | NA | NA | NA | 5492151..5581179 | MPFG | 60.5 | 89029 |
| NZ_CP031449.2 | Pseudomonas aeruginosa | 97 | 234 | NA | NA | NA | 5597669..5708907 | MPFG | 58.2 | 111239 |
| NZ_CP031659.1 | Pseudomonas aeruginosa | PABL012 | NA | NA | NA | NA | 5260858..5365171 | MPFG | 60.8 | 104314 |
| NZ_CP031660.1 | Pseudomonas aeruginosa | PABL017 | 2167 | NA | NA | NA | 5228262..5320455 | MPFG | 59.9 | 92194 |
| NZ_CP032126.1 | Pseudomonas aeruginosa | PAO1161 | 549 | NA | NA | NA | 5086423..5194320 | MPFG | 59.6 | 107898 |
| NZ_CP032257.1 | Pseudomonas aeruginosa | AR_0111 | 233 | NA | NA | NA | 1227760..1316873 | MPFG | 60.7 | 89114 |
| NZ_CP032552.1 | Pseudomonas aeruginosa | PA34 | 1284 | NA | NA | NA | 5459604..5545649 | MPFG | 60.3 | 86046 |
| NZ_CP032569.1 | Pseudomonas aeruginosa | BA7823 | 357 | NA | NA | NA | 5058369..5152012 | MPFG | 60.6 | 93644 |
| NZ_CP033439.1 | Pseudomonas aeruginosa | SP4528 | 357 | NA | NA | NA | 1065389..1155743 | MPFG | 60.5 | 90355 |
| NZ_CP033684.1 | Pseudomonas aeruginosa | H26027 | 17 | NA | NA | NA | 5743498..5837210 | MPFG | 60.4 | 93713 |
| NZ_CP033771.1 | Pseudomonas aeruginosa | FDAARGOS_532 | 179 | NA | NA | NA | 3332235..3423228 | MPFG | 60.0 | 90994 |
| NZ_CP033832.1 | Pseudomonas aeruginosa | FDAARGOS_505 | 179 | NA | NA | NA | 37920..127485 | MPFG | 60.2 | 89566 |
| NZ_CP033835.1 | Pseudomonas aeruginosa | FDAARGOS_570 | 244 | NA | NA | NA | 2186141..2271377 | NA | 60.6 | 85237 |
| NZ_CP033835.1 | Pseudomonas aeruginosa | FDAARGOS_570 | 244 | NA | NA | NA | 1337211..1429410 | MPFG | 60.4 | 92200 |
| NZ_CP034354.1 | Pseudomonas aeruginosa | IMP-13 | 621 | NA | NA | NA | 910412..1013390 | MPFG | 59.6 | 102979 |
| NZ_CP034369.1 | Pseudomonas aeruginosa | SP4371 | 357 | NA | NA | NA | 4706492..4795934 | MPFG | 60.4 | 89443 |
| NZ_CP034409.1 | Pseudomonas aeruginosa | SP4527 | 357 | NA | NA | NA | 3802866..3893221 | MPFG | 60.5 | 90356 |
| NZ_CP034435.1 | Pseudomonas aeruginosa | B14130 | 357 | NA | NA | NA | 1001650..1092003 | MPFG | 60.5 | 90354 |
| NZ_CP035739.1 | Pseudomonas aeruginosa | 1334/14 | 234 | NA | NA | NA | 5368921..5488563 | MPFG | 58.4 | 119643 |
| NZ_CP039293.1 | Pseudomonas aeruginosa | PABL048 | 298 | NA | NA | NA | 5578776..5666623 | MPFG | 60.3 | 87848 |
| NZ_CP039988.1 | Pseudomonas aeruginosa | T2436 | NA | NA | NA | NA | 5454570..5547141 | MPFG | 58.6 | 92572 |
| NZ_CP039990.1 | Pseudomonas aeruginosa | T2101 | 708 | NA | NA | NA | 5251404..5335058 | MPFG | 60.6 | 83655 |
| NZ_CP040127.1 | Pseudomonas aeruginosa | PA298 | 277 | NA | Pyocin S5 | CAS-TypeIC | 5251106..5357222 | MPFG | 59.6 | 106117 |
| NZ_CP040684.1 | Pseudomonas aeruginosa | C79 | 316 | NA | NA | NA | 5922289..6005409 | MPFG | 61.0 | 83121 |
| NZ_CP041013.1 | Pseudomonas aeruginosa | FDAARGOS_610 | 252 | NA | NA | NA | 1254133..1357505 | MPFG | 60.5 | 103373 |
| NZ_CP041354.1 | Pseudomonas aeruginosa | AZPAE15042 | 2211 | NA | NA | NA | 5208858..5312808 | MPFG | 59.3 | 103951 |
| NZ_CP041771.1 | Pseudomonas aeruginosa | A681 | 274 | NA | NA | NA | 4614086..4707996 | MPFG | 59.2 | 93911 |
| NZ_CP041774.1 | Pseudomonas aeruginosa | 60503 | 773 | NA | NA | NA | 5519535..5613671 | MPFG | 60.0 | 94137 |
| NZ_CP043328.1 | Pseudomonas aeruginosa | CCUG 51971 | 235 | NA | Pyocin S5 | CAS-TypeIC | 5638612..5757809 | MPFG | 59.6 | 119198 |
| NZ_CP044006.1 | Pseudomonas aeruginosa | E90 | 282 | NA | NA | NA | 4742387..4839256 | MPFG | 60.3 | 96870 |
| NZ_CP045739.1 | Pseudomonas aeruginosa | AG1 | 111 | NA | NA | NA | 5788614..5873476 | MPFG | 60.8 | 84863 |
| NZ_CP046060.1 | Pseudomonas aeruginosa | 1811-18R001 | 395 | NA | NA | NA | 5988162..6087613 | MPFG | 60.5 | 99452 |
| NZ_CP046061.1 | Pseudomonas aeruginosa | 1811-13R031 | 395 | NA | NA | NA | 5987958..6087409 | MPFG | 60.5 | 99452 |
| NZ_CP046069.1 | Pseudomonas aeruginosa | KRP1 | 27 | NA | NA | NA | 5366781..5465070 | MPFG | 59.6 | 98290 |
| NZ_CP047592.1 | Pseudomonas aeruginosa | INP-43 | NA | NA | Pyocin S5 | NA | 5607949..5701975 | MPFG | 59.1 | 94027 |
| NZ_LR130527.1 | Pseudomonas aeruginosa | paerg002 | 262 | NA | NA | NA | 1796189..1903319 | MPFG | 60.4 | 107131 |
| NZ_LR130530.1 | Pseudomonas aeruginosa | paerg003 | 262 | NA | NA | NA | 5115586..5222717 | MPFG | 60.4 | 107132 |
| NZ_LR130531.1 | Pseudomonas aeruginosa | paerg004 | 262 | NA | NA | NA | 2433304..2540433 | MPFG | 60.4 | 107130 |
| NZ_LR130535.1 | Pseudomonas aeruginosa | paerg011 | 262 | NA | NA | NA | 5115719..5222850 | MPFG | 60.4 | 107132 |
| NZ_LR130536.1 | Pseudomonas aeruginosa | paerg010 | 262 | NA | NA | NA | 5115575..5222706 | MPFG | 60.4 | 107132 |
| NZ_LR130537.1 | Pseudomonas aeruginosa | paerg012 | 262 | NA | NA | NA | 5115596..5222727 | MPFG | 60.4 | 107132 |
| NZ_LR134300.1 | Pseudomonas fluorescens | NCTC10783 | 800 | NA | NA | NA | 3471634..3610133 | MPFG | 61.2 | 138500 |
| NZ_LR134308.1 | Pseudomonas aeruginosa | NCTC11445 | NA | NA | NA | NA | 718488..812871 | MPFG | 60.3 | 94384 |
| NZ_LR134309.1 | Pseudomonas aeruginosa | NCTC12903 | 155 | NA | NA | NA | 5511116..5613952 | MPFG | 60.6 | 102837 |
| NZ_LR590472.1 | Pseudomonas aeruginosa | NCTC13620 | NA | NA | NA | NA | 5476242..5579821 | NA | 60.9 | 103580 |
| NZ_LR590473.1 | Pseudomonas aeruginosa | NCTC13359 | 252 | NA | NA | NA | 5871185..5974561 | MPFG | 60.5 | 103377 |
| NZ_LR590474.1 | Pseudomonas aeruginosa | NCTC13618 | NA | NA | NA | NA | 4689973..4788263 | MPFG | 59.6 | 98291 |
| NZ_LS998783.1 | Pseudomonas aeruginosa | isolate 1 | 111 | NA | NA | NA | 5820251..5904145 | MPFG | 60.7 | 83895 |
| NZ_LT608330.1 | Pseudomonas aeruginosa | PA14Or | 253 | NA | Pyocin S5 | NA | 5254562..5362559 | MPFG | 59.7 | 107998 |
| NZ_LT883143.1 | Pseudomonas aeruginosa | C-NN2 | 17 | sul1, qacEdelta1, ant(2'')-Ia | NA | NA | 5577611..5704249 | NA | 61.3 | 126639 |
| NZ_LT969520.1 | Pseudomonas aeruginosa | RW109 | 111 | NA | NA | NA | 5629923..5724354 | MPFG | 60.3 | 94432 |
| NZ_LT969520.1 | Pseudomonas aeruginosa | RW109 | 111 | NA | NA | NA | 5724373..5820312 | MPFG | 60.1 | 95928 |

NA stands for no information available.
