# Supplementary material for: ICEs Are the Main Reservoirs of the Ciprofloxacin-Modifying crpP Gene in Pseudomonas aeruginosa
Source: Genes (Basel). 2020 Aug 4;11(8):889. doi: 10.3390/genes11080889 (PMC7463715; doi:10.3390/genes11080889)
Supplement: Supplementary file 1 [file genes-11-00889-s001.zip › Table_S6.docx]

**Table S6**. Functional annotation of the ICE proteomes identified in this study.

| **Query_name** | **Seed_eggNOG_ortholog** | **Seed_ortholog_evalue** | **Seed_ortholog_score** | **Best_tax_level** | **COG Functional Category** |
| --- | --- | --- | --- | --- | --- |
| NC_008463.1_00001 | 287.DR97_1723 | 2.8e-154 | 551.2 | Pseudomonas aeruginosa group | D |
| NC_008463.1_00002 | 287.DR97_1724 | 2.5e-119 | 434.9 | Pseudomonas aeruginosa group | - |
| NC_008463.1_00004 | 287.DR97_1726 | 5.6e-10 | 69.3 | Pseudomonas aeruginosa group | - |
| NC_008463.1_00005 | 287.DR97_1727 | 2.4e-95 | 355.1 | Gammaproteobacteria | - |
| NC_008463.1_00006 | 1395571.TMS3_0120310 | 4.6e-70 | 271.2 | Gammaproteobacteria | S |
| NC_008463.1_00007 | 287.DR97_1729 | 1.6e-131 | 475.3 | Pseudomonas aeruginosa group | S |
| NC_008463.1_00008 | 287.DR97_1730 | 2,00E-27 | 127.9 | Pseudomonas aeruginosa group | - |
| NC_008463.1_00009 | 287.DR97_1731 | 1.9e-237 | 828.2 | Pseudomonas aeruginosa group | L |
| NC_008463.1_00011 | 1388763.O165_001830 | 1.1e-36 | 159.8 | Pseudomonas putida group | - |
| NC_008463.1_00014 | 1388763.O165_001825 | 1.5e-14 | 85.1 | Pseudomonas putida group | - |
| NC_008463.1_00015 | 287.DR97_4017 | 5.3e-184 | 650.2 | Pseudomonas aeruginosa group | S |
| NC_008463.1_00017 | 287.DR97_1741 | 1,00E-275 | 955.7 | Gammaproteobacteria | K |
| NC_008463.1_00018 | 287.DR97_1742 | 1.3e-113 | 416.0 | Pseudomonas aeruginosa group | S |
| NC_008463.1_00019 | 287.DR97_1743 | 2,00E-231 | 808.1 | Gammaproteobacteria | - |
| NC_008463.1_00022 | 287.DR97_1746 | 1.7e-132 | 478.8 | Gammaproteobacteria | S |
| NC_008463.1_00023 | 287.DR97_1747 | 7.3e-76 | 290.0 | Gammaproteobacteria | S |
| NC_008463.1_00024 | 287.DR97_1749 | 1.9e-75 | 288.5 | Pseudomonas aeruginosa group | L |
| NC_008463.1_00027 | 287.DR97_1750 | 0.0 | 1219.9 | Pseudomonas aeruginosa group | G |
| NC_008463.1_00028 | 390235.PputW619_3356 | 1.8e-41 | 175.6 | Proteobacteria | - |
| NC_008463.1_00029 | 1415630.U771_05000 | 7.2e-134 | 484.6 | Proteobacteria | - |
| NC_008463.1_00030 | 287.DR97_1753 | 0.0 | 1261.1 | Pseudomonas aeruginosa group | KL |
| NC_008463.1_00031 | 287.DR97_1756 | 6.2e-19 | 99.4 | Pseudomonas aeruginosa group | S |
| NC_008463.1_00032 | 634499.EpC_11210 | 2.3e-26 | 127.1 | Erwinia | S |
| NC_008463.1_00033 | 287.DR97_1756 | 3.6e-157 | 561.2 | Pseudomonas aeruginosa group | S |
| NC_008463.1_00034 | 287.DR97_1757 | 2.8e-294 | 1017.3 | Pseudomonas aeruginosa group | NU |
| NC_008463.1_00035 | 287.DR97_1758 | 8.8e-248 | 862.4 | Gammaproteobacteria | S |
| NC_008463.1_00036 | 287.DR97_1759 | 3.4e-81 | 307.8 | Gammaproteobacteria | - |
| NC_008463.1_00037 | 287.DR97_1760 | 8.8e-303 | 1045.4 | Pseudomonas aeruginosa group | NU |
| NC_008463.1_00038 | 287.DR97_1761 | 8.4e-196 | 689.5 | Pseudomonas aeruginosa group | NU |
| NC_008463.1_00039 | 287.DR97_1762 | 5.8e-57 | 227.3 | Pseudomonas aeruginosa group | S |
| NC_008463.1_00040 | 287.DR97_1763 | 5.7e-140 | 503.8 | Pseudomonas aeruginosa group | NU |
| NC_008463.1_00041 | 287.DR97_1764 | 3.2e-149 | 535.0 | Pseudomonas aeruginosa group | NU |
| NC_008463.1_00042 | 1395571.TMS3_0120490 | 2,00E-31 | 142.1 | Proteobacteria | S |
| NC_008463.1_00043 | 629265.PMA4326_19278 | 4.5e-07 | 61.6 | Pseudomonas syringae group | - |
| NC_008463.1_00046 | 287.DR97_1769 | 4.5e-80 | 303.9 | Gammaproteobacteria | S |
| NC_008463.1_00048 | 287.DR97_1771 | 8.6e-22 | 109.0 | Gammaproteobacteria | - |
| NC_008463.1_00050 | 287.DR97_1772 | 1.2e-107 | 396.0 | Gammaproteobacteria | S |
| NC_008463.1_00051 | 1388763.O165_001385 | 7.5e-21 | 106.7 | Pseudomonas putida group | - |
| NC_008463.1_00052 | 287.DR97_1774 | 3,00E-92 | 344.7 | Gammaproteobacteria | S |
| NC_008463.1_00054 | 1442599.JAAN01000014_gene3439 | 1.3e-23 | 115.9 | Xanthomonadales | - |
| NC_008463.1_00055 | 287.DR97_1777 | 8.3e-38 | 162.5 | Pseudomonas aeruginosa group | - |
| NC_008463.1_00056 | 287.DR97_1778 | 2.9e-113 | 414.5 | Gammaproteobacteria | - |
| NC_008463.1_00057 | 287.DR97_1779 | 1.1e-259 | 902.1 | Gammaproteobacteria | L |
| NC_008463.1_00058 | 1388763.O165_001355 | 0.0 | 1387.1 | Gammaproteobacteria | L |
| NC_008463.1_00059 | 629265.PMA4326_28970 | 3.6e-62 | 245.7 | Proteobacteria | - |
| NC_008463.1_00060 | 160488.PP_3499 | 2.1e-36 | 157.9 | Gammaproteobacteria | L |
| NC_008463.1_00061 | 1042876.PPS_4069 | 6.6e-59 | 233.0 | Gammaproteobacteria | L |
| NC_008463.1_00062 | 160488.PP_3501 | 3.5e-272 | 943.7 | Gammaproteobacteria | S |
| NC_008463.1_00063 | 157783.LK03_17885 | 4.5e-21 | 107.5 | Bacteria | K |
| NC_008463.1_00064 | 1205753.A989_13794 | 2,00E-07 | 63.2 | Xanthomonadales | - |
| NC_008463.1_00067 | 1395571.TMS3_0120570 | 3.8e-69 | 268.1 | Gammaproteobacteria | - |
| NC_008463.1_00068 | 287.DR97_1789 | 1.1e-128 | 466.1 | Gammaproteobacteria | O |
| NC_008463.1_00069 | 287.DR97_1790 | 6.4e-102 | 376.7 | Pseudomonas aeruginosa group | M |
| NC_008463.1_00070 | 287.DR97_1791 | 9.8e-83 | 312.8 | Gammaproteobacteria | KT |
| NC_008463.1_00072 | 287.DR97_1793 | 0.0 | 1459.1 | Gammaproteobacteria | U |
| NC_008463.1_00073 | 287.DR97_1794 | 7.7e-132 | 476.5 | Gammaproteobacteria | S |
| NC_008463.1_00074 | 1163398.AJJP01000227_gene2388 | 8.2e-24 | 115.5 | Gammaproteobacteria | L |
| NC_008463.1_00075 | 1437882.AZRU01000211_gene2483 | 4,00E-69 | 267.7 | Pseudomonas aeruginosa group | NU |
| NC_008463.1_00076 | 1294143.H681_06075 | 2.9e-94 | 351.7 | Gammaproteobacteria | NU |
| NC_008463.1_00077 | 1294143.H681_06070 | 2,00E-36 | 158.3 | Gammaproteobacteria | NU |
| NC_008463.1_00078 | 1226994.AMZB01000117_gene2582 | 0.0 | 1270.0 | Pseudomonas aeruginosa group | NU |
| NC_008463.1_00079 | 1437882.AZRU01000211_gene2480 | 1.3e-209 | 735.7 | Pseudomonas aeruginosa group | NU |
| NC_008463.1_00080 | 1294143.H681_06060 | 3.1e-82 | 311.6 | Gammaproteobacteria | NU |
| NC_008463.1_00081 | 95619.PM1_0206900 | 3.4e-89 | 334.7 | Proteobacteria | K |
| NC_008463.1_00082 | 95619.PM1_0206895 | 0.0 | 1437.2 | Gammaproteobacteria | T |
| NC_008463.1_00083 | 95619.PM1_0206890 | 4.5e-166 | 590.9 | Gammaproteobacteria | T |
| NC_008463.1_00084 | 95619.PM1_0206885 | 0.0 | 1171.0 | Gammaproteobacteria | T |
| NC_008463.1_00085 | 287.DR97_1794 | 2.6e-115 | 421.4 | Gammaproteobacteria | S |
| NC_008463.1_00086 | 287.DR97_1795 | 7.5e-264 | 916.0 | Pseudomonas aeruginosa group | L |
| NC_008463.1_00087 | 287.DR97_1796 | 2.9e-175 | 621.3 | Pseudomonas aeruginosa group | - |
| NC_008463.1_00088 | 399739.Pmen_3824 | 1.4e-15 | 89.0 | Pseudomonas aeruginosa group | S |
| NC_008463.1_00089 | 1005395.CSV86_18312 | 4.2e-20 | 104.0 | Pseudomonas putida group | - |
| NC_008463.1_00090 | 287.DR97_1817 | 3.2e-53 | 214.2 | Proteobacteria | S |
| NC_008463.1_00091 | 1395571.TMS3_0124565 | 5.8e-17 | 93.2 | Gammaproteobacteria | S |
| NC_008463.1_00092 | 287.DR97_1819 | 1.1e-51 | 209.1 | Gammaproteobacteria | S |
| NC_008463.1_00093 | 220664.PFL_6232 | 1.9e-33 | 148.7 | Pseudomonas fluorescens group | S |
| NC_008463.1_00094 | 287.DR97_1821 | 5.4e-129 | 466.8 | Gammaproteobacteria | S |
| NC_008463.1_00095 | 287.DR97_1822 | 9,00E-156 | 556.2 | Gammaproteobacteria | NU |
| NC_008463.1_00096 | 287.DR97_1823 | 5.7e-251 | 873.2 | Gammaproteobacteria | M |
| NC_008463.1_00097 | 287.DR97_1824 | 2.9e-78 | 297.7 | Gammaproteobacteria | S |
| NC_008463.1_00098 | 287.DR97_1825 | 0.0 | 1944.9 | Gammaproteobacteria | U |
| NC_008463.1_00099 | 322710.Avin_36290 | 1.6e-26 | 125.2 | Gammaproteobacteria | - |
| NC_008463.1_00100 | 287.DR97_1828 | 1.2e-115 | 422.5 | Pseudomonas aeruginosa group | O |
| NC_008463.1_00102 | 287.DR97_1830 | 1.3e-181 | 642.1 | Gammaproteobacteria | S |
| NC_008463.1_00103 | 287.DR97_1831 | 5.6e-245 | 853.2 | Pseudomonas aeruginosa group | S |
| NC_008463.1_00104 | 1388763.O165_001190 | 2.7e-07 | 61.6 | Pseudomonas putida group | - |
| NC_008463.1_00105 | 287.DR97_1833 | 5.3e-281 | 973.0 | Gammaproteobacteria | S |
| NC_008463.1_00106 | 287.DR97_1834 | 1.4e-39 | 169.1 | Bacteria | K |
| NC_008463.1_00107 | 287.DR97_1835 | 3.2e-56 | 224.2 | Proteobacteria | S |
| NC_008463.1_00108 | 1196835.A458_20135 | 1.2e-13 | 81.6 | Pseudomonas stutzeri group | L |
| NC_008463.1_00109 | 95619.PM1_0220210 | 0.0 | 1451.8 | Gammaproteobacteria | L |
| NC_008463.1_00110 | 195105.CN97_16180 | 1,00E-167 | 597.0 | Alphaproteobacteria | L |
| NC_008463.1_00112 | 318167.Sfri_3393 | 1,00E-64 | 253.1 | Bacteria | F |
| NC_008463.1_00113 | 1001585.MDS_1571 | 1.5e-121 | 442.2 | Gammaproteobacteria | S |
| NC_008463.1_00114 | 318167.Sfri_3391 | 1.9e-55 | 222.2 | Bacteria | F |
| NC_008463.1_00115 | 287.DR97_1840 | 0.0 | 1210.7 | Gammaproteobacteria | S |
| NC_008463.1_00116 | 287.DR97_1841 | 1.3e-144 | 519.2 | Gammaproteobacteria | L |
| NC_009656.1_00002 | 287.DR97_1724 | 1.8e-117 | 428.7 | Pseudomonas aeruginosa group | - |
| NC_009656.1_00004 | 287.DR97_1726 | 3.9e-73 | 281.6 | Pseudomonas aeruginosa group | - |
| NC_009656.1_00018 | 287.DR97_1739 | 4.2e-169 | 600.5 | Bacteria | L |
| NC_009656.1_00020 | 287.DR97_1741 | 1.8e-269 | 934.9 | Gammaproteobacteria | K |
| NC_009656.1_00021 | 287.DR97_1742 | 2.2e-102 | 378.6 | Pseudomonas aeruginosa group | S |
| NC_009656.1_00022 | 287.DR97_1743 | 1.1e-234 | 818.9 | Gammaproteobacteria | - |
| NC_009656.1_00028 | 287.DR97_1748 | 6.7e-153 | 546.6 | Gammaproteobacteria | S |
| NC_009656.1_00030 | 1282356.H045_14305 | 3.6e-15 | 87.8 | Pseudomonas fluorescens group | K |
| NC_009656.1_00031 | 1005395.CSV86_12865 | 7.2e-13 | 79.3 | Pseudomonas putida group | - |
| NC_009656.1_00033 | 1380387.JADM01000034_gene1737 | 3.7e-65 | 255.0 | Gammaproteobacteria | - |
| NC_009656.1_00038 | 287.DR97_1754 | 6.1e-45 | 186.4 | Pseudomonas aeruginosa group | K |
| NC_009656.1_00039 | 287.DR97_1755 | 1.9e-49 | 201.4 | Pseudomonas aeruginosa group | S |
| NC_009656.1_00045 | 287.DR97_1761 | 1.7e-196 | 691.8 | Pseudomonas aeruginosa group | NU |
| NC_009656.1_00047 | 287.DR97_1763 | 4.1e-170 | 604.0 | Pseudomonas aeruginosa group | NU |
| NC_009656.1_00053 | 287.DR97_1769 | 4.6e-85 | 320.5 | Gammaproteobacteria | S |
| NC_009656.1_00055 | 287.DR97_1771 | 2.5e-29 | 134.0 | Gammaproteobacteria | - |
| NC_009656.1_00056 | 287.DR97_1772 | 1.3e-141 | 508.8 | Gammaproteobacteria | S |
| NC_009656.1_00058 | 287.DR97_1774 | 2.3e-113 | 414.8 | Gammaproteobacteria | S |
| NC_009656.1_00078 | 287.DR97_1795 | 2.8e-266 | 924.1 | Pseudomonas aeruginosa group | L |
| NC_009656.1_00093 | 62977.ACIAD1842 | 4.6e-08 | 65.5 | Moraxellaceae | - |
| NC_009656.1_00094 | 287.DR97_1828 | 5,00E-21 | 106.3 | Pseudomonas aeruginosa group | O |
| NC_009656.1_00096 | 1415630.U771_04520 | 1.1e-34 | 152.9 | Gammaproteobacteria | S |
| NC_009656.1_00097 | 287.DR97_1830 | 1.7e-181 | 641.7 | Gammaproteobacteria | S |
| NC_009656.1_00098 | 287.DR97_1831 | 4.7e-244 | 850.1 | Pseudomonas aeruginosa group | S |
| NC_009656.1_00104 | 287.DR97_1836 | 2.7e-163 | 581.3 | Gammaproteobacteria | - |
| NC_009656.1_00105 | 287.DR97_1837 | 1.1e-39 | 168.7 | Gammaproteobacteria | - |
| NC_009656.1_00106 | 292.DM42_543 | 2.7e-214 | 751.1 | Burkholderiaceae | L |
| NC_009656.1_00107 | 287.DR97_1840 | 0.0 | 1201.8 | Gammaproteobacteria | S |
| NC_009656.1_00108 | 287.DR97_1841 | 1.5e-138 | 499.6 | Gammaproteobacteria | L |
| NC_018080.1_00005 | 287.DR97_1730 | 6.8e-09 | 65.5 | Pseudomonas aeruginosa group | - |
| NC_018080.1_00016 | 287.DR97_1741 | 0.0 | 1096.3 | Gammaproteobacteria | K |
| NC_018080.1_00017 | 287.DR97_1742 | 9.6e-138 | 496.1 | Pseudomonas aeruginosa group | S |
| NC_018080.1_00020 | 287.DR97_1746 | 2.8e-131 | 474.6 | Gammaproteobacteria | S |
| NC_018080.1_00022 | 287.DR97_1748 | 5.4e-126 | 457.2 | Gammaproteobacteria | S |
| NC_018080.1_00026 | 287.DR97_1750 | 0.0 | 1234.2 | Pseudomonas aeruginosa group | G |
| NC_018080.1_00027 | 390235.PputW619_3356 | 5.7e-40 | 170.6 | Proteobacteria | - |
| NC_018080.1_00058 | 1216976.AX27061_1104 | 0.0 | 1157.1 | Alcaligenaceae | LV |
| NC_018080.1_00059 | 1395571.TMS3_0120555 | 5,00E-224 | 783.5 | Gammaproteobacteria | L |
| NC_018080.1_00060 | 1196835.A458_07825 | 1.9e-33 | 147.9 | Pseudomonas stutzeri group | L |
| NC_018080.1_00061 | 223283.PSPTO_0048 | 1.8e-175 | 622.1 | Gammaproteobacteria | S |
| NC_018080.1_00062 | 223283.PSPTO_0049 | 5.1e-144 | 516.9 | Gammaproteobacteria | L |
| NC_018080.1_00063 | 223283.PSPTO_0050 | 6.4e-222 | 776.5 | Gammaproteobacteria | - |
| NC_018080.1_00084 | 287.DR97_1825 | 0.0 | 1984.9 | Gammaproteobacteria | U |
| NC_018080.1_00088 | 220664.PFL_3006 | 1.2e-21 | 110.2 | Proteobacteria | - |
| NC_018080.1_00090 | 1415630.U771_04520 | 4.7e-33 | 147.5 | Gammaproteobacteria | S |
| NC_018080.1_00100 | 740709.A10D4_13118 | 1.5e-68 | 267.7 | Gammaproteobacteria | D |
| NC_018080.1_00102 | 643562.Daes_2533 | 2.5e-130 | 472.2 | Deltaproteobacteria | L |
| NC_020912.1_00006 | 287.DR97_1730 | 4.2e-30 | 136.7 | Pseudomonas aeruginosa group | - |
| NC_020912.1_00015 | 287.DR97_1741 | 2.8e-278 | 964.1 | Gammaproteobacteria | K |
| NC_020912.1_00016 | 287.DR97_1742 | 3.4e-127 | 461.1 | Pseudomonas aeruginosa group | S |
| NC_020912.1_00051 | 287.DR97_1779 | 6,00E-41 | 172.9 | Gammaproteobacteria | L |
| NC_020912.1_00054 | 1226994.AMZB01000079_gene80 | 3.6e-222 | 777.3 | Pseudomonas aeruginosa group | L |
| NC_020912.1_00055 | 1301098.PKB_1302 | 6.7e-23 | 112.5 | Bacteria | K |
| NC_020912.1_00057 | 287.DR97_1781 | 5,00E-130 | 470.3 | Pseudomonas aeruginosa group | S |
| NC_020912.1_00058 | 287.DR97_5422 | 2.5e-21 | 107.8 | Pseudomonas aeruginosa group | - |
| NC_020912.1_00065 | 287.DR97_1794 | 1.7e-131 | 475.3 | Gammaproteobacteria | S |
| NC_020912.1_00066 | 159087.Daro_2205 | 4.5e-83 | 314.7 | Betaproteobacteria | - |
| NC_020912.1_00070 | 287.DR97_1819 | 2.9e-52 | 211.1 | Gammaproteobacteria | S |
| NC_020912.1_00080 | 1216976.AX27061_3953 | 2.2e-197 | 694.9 | Alcaligenaceae | S |
| NC_020912.1_00081 | 1144325.PMI22_04214 | 7.1e-18 | 97.1 | Bacteria | S |
| NC_020912.1_00082 | 1124983.PFLCHA0_c13620 | 4.2e-105 | 387.9 | Pseudomonas fluorescens group | S |
| NC_020912.1_00083 | 1415630.U771_04520 | 7.3e-34 | 150.2 | Gammaproteobacteria | S |
| NC_020912.1_00091 | 1120999.JONM01000038_gene1893 | 2,00E-24 | 118.2 | Neisseriales | L |
| NC_020912.1_00092 | 1437882.AZRU01000053_gene3683 | 1.3e-42 | 178.7 | Pseudomonas aeruginosa group | L |
| NC_020912.1_00093 | 1437882.AZRU01000099_gene1125 | 7.2e-113 | 413.3 | Pseudomonas aeruginosa group | S |
| NC_020912.1_00094 | 1437882.AZRU01000099_gene1124 | 1.4e-249 | 868.6 | Pseudomonas aeruginosa group | I |
| NC_020912.1_00095 | 1226994.AMZB01000048_gene3885 | 1.1e-116 | 426.0 | Pseudomonas aeruginosa group | K |
| NC_020912.1_00096 | 1437882.AZRU01000099_gene1122 | 4.4e-133 | 480.7 | Pseudomonas aeruginosa group | S |
| NC_020912.1_00097 | 1437882.AZRU01000099_gene1121 | 1.4e-102 | 379.0 | Pseudomonas aeruginosa group | NU |
| NC_020912.1_00098 | 1437882.AZRU01000099_gene1120 | 7.8e-112 | 409.8 | Pseudomonas aeruginosa group | T |
| NC_020912.1_00099 | 1226994.AMZB01000048_gene3889 | 2.4e-224 | 784.6 | Pseudomonas aeruginosa group | T |
| NC_020912.1_00100 | 1439940.BAY1663_04844 | 1.9e-51 | 209.5 | Gammaproteobacteria | C |
| NC_020912.1_00101 | 1452718.JBOY01000050_gene1156 | 5.7e-44 | 183.7 | Gammaproteobacteria | P |
| NC_020912.1_00102 | 1226994.AMZB01000108_gene1860 | 1.1e-177 | 629.8 | Pseudomonas aeruginosa group | C |
| NC_020912.1_00103 | 287.DR97_1130 | 4.9e-41 | 173.3 | Pseudomonas aeruginosa group | L |
| NC_020912.1_00104 | 287.DR97_1131 | 3.1e-142 | 511.1 | Pseudomonas aeruginosa group | L |
| NC_020912.1_00105 | 1437882.AZRU01000085_gene6787 | 8.2e-67 | 260.0 | Pseudomonas aeruginosa group | H |
| NC_020912.1_00106 | 1437882.AZRU01000085_gene6786 | 1.7e-83 | 315.5 | Pseudomonas aeruginosa group | S |
| NC_020912.1_00107 | 1226994.AMZB01000115_gene2166 | 1.2e-27 | 129.0 | Gammaproteobacteria | S |
| NC_020912.1_00108 | 1437882.AZRU01000085_gene6784 | 1.7e-271 | 941.4 | Pseudomonas aeruginosa group | S |
| NC_020912.1_00109 | 1226994.AMZB01000115_gene2164 | 4.7e-37 | 161.0 | Pseudomonas aeruginosa group | S |
| NC_020912.1_00110 | 1437882.AZRU01000011_gene3553 | 1.1e-207 | 729.2 | Pseudomonas aeruginosa group | M |
| NC_020912.1_00111 | 1437882.AZRU01000011_gene3554 | 4.2e-123 | 447.6 | Pseudomonas aeruginosa group | S |
| NC_020912.1_00112 | 1226994.AMZB01000115_gene2161 | 1.6e-214 | 751.9 | Pseudomonas aeruginosa group | S |
| NC_020912.1_00113 | 1437882.AZRU01000011_gene3556 | 9.3e-101 | 373.2 | Pseudomonas aeruginosa group | S |
| NC_020912.1_00114 | 1226994.AMZB01000115_gene2159 | 6.2e-62 | 243.4 | Pseudomonas aeruginosa group | S |
| NC_020912.1_00115 | 1437882.AZRU01000011_gene3558 | 2.4e-62 | 245.0 | Pseudomonas aeruginosa group | T |
| NC_020912.1_00116 | 1437882.AZRU01000011_gene3559 | 1.5e-110 | 406.0 | Pseudomonas aeruginosa group | K |
| NC_020912.1_00117 | 290315.Clim_2475 | 0.0 | 1414.1 | Bacteria | - |
| NC_020912.1_00118 | 523791.Kkor_0811 | 6.6e-46 | 190.7 | Gammaproteobacteria | - |
| NC_021577.1_00003 | 1439940.BAY1663_04194 | 8.2e-144 | 516.9 | Gammaproteobacteria | I |
| NC_021577.1_00004 | 1301098.PKB_5470 | 5,00E-186 | 657.9 | Gammaproteobacteria | M |
| NC_021577.1_00005 | 748247.AZKH_4089 | 6.6e-15 | 87.4 | Rhodocyclales | - |
| NC_021577.1_00007 | 1301098.PKB_5465 | 3.9e-112 | 411.0 | Gammaproteobacteria | K |
| NC_021577.1_00010 | 266265.Bxe_C1346 | 0.0 | 3630.9 | Burkholderiaceae | G |
| NC_021577.1_00011 | 287.DR97_1835 | 1.5e-50 | 205.3 | Proteobacteria | S |
| NC_021577.1_00015 | 287.DR97_1831 | 2.3e-246 | 857.8 | Pseudomonas aeruginosa group | S |
| NC_021577.1_00043 | 1301098.PKB_1306 | 1.1e-144 | 519.2 | Gammaproteobacteria | L |
| NC_021577.1_00044 | 287.DR97_5228 | 4.5e-157 | 560.5 | Pseudomonas aeruginosa group | L |
| NC_021577.1_00045 | 287.DR97_5229 | 3,00E-34 | 151.0 | Pseudomonas aeruginosa group | L |
| NC_021577.1_00046 | 1301098.PKB_1307 | 1.1e-47 | 196.1 | Gammaproteobacteria | - |
| NC_021577.1_00047 | 1301098.PKB_1308 | 1.8e-139 | 501.9 | Gammaproteobacteria | IQ |
| NC_021577.1_00048 | 1301098.PKB_1309 | 3.5e-97 | 360.9 | Gammaproteobacteria | K |
| NC_021577.1_00049 | 1301098.PKB_1310 | 5.5e-56 | 223.4 | Gammaproteobacteria | S |
| NC_021577.1_00050 | 1301098.PKB_1311 | 1.8e-119 | 435.3 | Bacteria | Q |
| NC_021577.1_00051 | 1301098.PKB_1312 | 3.5e-97 | 360.9 | Proteobacteria | O |
| NC_021577.1_00052 | 1301098.PKB_1313 | 1.4e-68 | 265.8 | Gammaproteobacteria | C |
| NC_021577.1_00076 | 1144307.PMI04_00913 | 6.2e-150 | 537.3 | Sphingomonadales | - |
| NC_021577.1_00077 | 1038922.PflQ2_3285 | 7.3e-102 | 376.7 | Gammaproteobacteria | L |
| NC_021577.1_00080 | 287.DR97_1751 | 7.3e-32 | 142.5 | Pseudomonas aeruginosa group | K |
| NC_021577.1_00083 | 1005395.CSV86_29057 | 1,00E-31 | 142.9 | Bacteria | - |
| NC_021577.1_00084 | 1112217.PPL19_07856 | 4.8e-59 | 233.8 | Gammaproteobacteria | S |
| NC_021577.1_00088 | 287.DR97_1748 | 5.1e-132 | 477.2 | Gammaproteobacteria | S |
| NC_021577.1_00090 | 287.DR97_1746 | 2,00E-132 | 478.4 | Gammaproteobacteria | S |
| NC_021577.1_00095 | 287.DR97_1741 | 1.2e-284 | 985.3 | Gammaproteobacteria | K |
| NC_021577.1_00109 | 287.DR97_1727 | 3.8e-93 | 347.8 | Gammaproteobacteria | - |
| NC_022806.1_00001 | 287.DR97_1841 | 6,00E-233 | 813.1 | Gammaproteobacteria | L |
| NC_022806.1_00002 | 287.DR97_1840 | 0.0 | 1231.5 | Gammaproteobacteria | S |
| NC_022806.1_00003 | 1282356.H045_00265 | 0.0 | 1147.9 | Pseudomonas fluorescens group | L |
| NC_022806.1_00004 | 1282356.H045_00270 | 2.4e-298 | 1030.8 | Proteobacteria | L |
| NC_022806.1_00019 | 287.DR97_1821 | 5.4e-129 | 466.8 | Gammaproteobacteria | S |
| NC_022806.1_00024 | 1005395.CSV86_18312 | 9.2e-15 | 86.3 | Pseudomonas putida group | - |
| NC_022806.1_00025 | 1123519.PSJM300_15380 | 1.4e-120 | 439.5 | Pseudomonas stutzeri group | P |
| NC_022806.1_00026 | 1123519.PSJM300_15385 | 4.9e-102 | 377.5 | Pseudomonas stutzeri group | P |
| NC_022806.1_00027 | 1439940.BAY1663_01516 | 9.7e-109 | 399.8 | Proteobacteria | P |
| NC_022806.1_00028 | 1123519.PSJM300_15395 | 9.6e-143 | 513.5 | Pseudomonas stutzeri group | P |
| NC_022806.1_00029 | 1123519.PSJM300_15400 | 3.6e-116 | 424.5 | Gammaproteobacteria | K |
| NC_022806.1_00030 | 1123519.PSJM300_15405 | 5.9e-200 | 703.7 | Gammaproteobacteria | O |
| NC_022806.1_00031 | 1439940.BAY1663_01512 | 5.8e-46 | 191.4 | Proteobacteria | - |
| NC_022806.1_00032 | 1123519.PSJM300_15415 | 1.4e-297 | 1029.2 | Gammaproteobacteria | P |
| NC_022806.1_00042 | 1218075.BAYA01000010_gene3322 | 3.5e-48 | 198.4 | Burkholderiaceae | K |
| NC_022806.1_00044 | 693444.D782_3750 | 4.6e-81 | 307.8 | Gammaproteobacteria | IQ |
| NC_022806.1_00045 | 1278971.AOGF01000025_gene1932 | 5.7e-12 | 77.8 | Pasteurellales | M |
| NC_022806.1_00046 | 983328.AFGH01000025_gene233 | 5.7e-54 | 218.0 | Epsilonproteobacteria | S |
| NC_022806.1_00047 | 1114970.PSF113_2143 | 9,00E-38 | 162.5 | Gammaproteobacteria | S |
| NC_022806.1_00048 | 1178482.BJB45_16675 | 1.3e-36 | 158.7 | Gammaproteobacteria | K |
| NC_022806.1_00049 | 571.MC52_30140 | 2.4e-90 | 338.2 | Gammaproteobacteria | L |
| NC_022806.1_00050 | 925775.XVE_2997 | 5.7e-07 | 61.6 | Xanthomonadales | - |
| NC_022806.1_00053 | 1042876.PPS_5228 | 0.0 | 1907.1 | Pseudomonas putida group | L |
| NC_022806.1_00082 | 287.DR97_784 | 1.2e-50 | 208.4 | Pseudomonas aeruginosa group | S |
| NC_022806.1_00084 | 40571.JOEA01000003_gene3840 | 8.5e-08 | 63.9 | Pseudonocardiales | - |
| NC_022806.1_00097 | 287.DR97_1741 | 2.3e-296 | 1024.2 | Gammaproteobacteria | K |
| NC_023019.1_00002 | 287.DR97_1724 | 4.2e-114 | 417.5 | Pseudomonas aeruginosa group | - |
| NC_023019.1_00004 | 287.DR97_1726 | 6,00E-85 | 320.9 | Pseudomonas aeruginosa group | - |
| NC_023019.1_00028 | 1268068.PG5_27780 | 9.1e-96 | 357.5 | Proteobacteria | - |
| NC_023019.1_00057 | 1247726.MIM_c31730 | 7.1e-64 | 250.4 | Alcaligenaceae | Q |
| NC_023019.1_00058 | 158822.LH89_18700 | 1.7e-77 | 296.2 | Gammaproteobacteria | K |
| NC_023019.1_00059 | 1419583.V466_11050 | 2.1e-85 | 322.8 | Pseudomonas fluorescens group | S |
| NC_023019.1_00060 | 412597.AEPN01000026_gene390 | 3.9e-52 | 211.5 | Paracoccus | O |
| NC_023019.1_00061 | 1429916.X566_12125 | 3.5e-131 | 475.3 | Bradyrhizobiaceae | EGP |
| NC_023019.1_00062 | 1429916.X566_12120 | 1.3e-76 | 293.5 | Bradyrhizobiaceae | V |
| NC_023019.1_00063 | 1441629.PCH70_15200 | 1.4e-70 | 273.1 | Pseudomonas syringae group | M |
| NC_023019.1_00065 | 1212548.B381_05156 | 1.2e-60 | 239.2 | Pseudomonas stutzeri group | L |
| NC_023019.1_00067 | 316273.XCV4227 | 7.5e-07 | 61.2 | Xanthomonadales | - |
| NC_023019.1_00069 | 287.DR97_6328 | 7,00E-56 | 224.6 | Bacteria | CH |
| NC_023019.1_00078 | 287.DR97_1795 | 5.3e-39 | 167.2 | Pseudomonas aeruginosa group | L |
| NC_023019.1_00101 | 159087.Daro_2205 | 8.6e-82 | 310.5 | Betaproteobacteria | - |
| NC_023149.1_00027 | 287.DR97_1749 | 3.3e-85 | 320.9 | Pseudomonas aeruginosa group | L |
| NC_023149.1_00067 | 287.DR97_5422 | 9.6e-21 | 105.9 | Pseudomonas aeruginosa group | - |
| NC_023149.1_00068 | 287.DR97_1782 | 7.6e-64 | 249.6 | Gammaproteobacteria | - |
| NC_023149.1_00069 | 287.DR97_1783 | 5.5e-101 | 373.6 | Gammaproteobacteria | - |
| NC_023149.1_00070 | 287.DR97_1784 | 1.2e-103 | 382.5 | Gammaproteobacteria | S |
| NC_023149.1_00101 | 287.DR97_1815 | 7.7e-131 | 473.0 | Pseudomonas aeruginosa group | T |
| NC_023149.1_00102 | 287.DR97_1814 | 2.8e-105 | 387.9 | Gammaproteobacteria | K |
| NC_023149.1_00103 | 287.DR97_1813 | 2.7e-140 | 504.6 | Pseudomonas aeruginosa group | IQ |
| NC_023149.1_00104 | 287.DR97_1812 | 3.2e-82 | 311.2 | Pseudomonas aeruginosa group | O |
| NC_023149.1_00107 | 287.DR97_1797 | 0.0 | 1963.3 | Pseudomonas aeruginosa group | L |
| NZ_AP014651.1_00030 | 1415630.U771_05000 | 1.1e-131 | 477.2 | Proteobacteria | - |
| NZ_AP014651.1_00059 | 1226994.AMZB01000079_gene80 | 1.1e-223 | 782.3 | Pseudomonas aeruginosa group | L |
| NZ_AP014651.1_00083 | 287.DR97_1819 | 9.8e-53 | 212.6 | Gammaproteobacteria | S |
| NZ_AP014839.1_00063 | 1323663.AROI01000055_gene1436 | 7.5e-178 | 629.8 | Gammaproteobacteria | L |
| NZ_AP014839.1_00079 | 1323663.AROI01000055_gene1436 | 7.5e-178 | 629.8 | Gammaproteobacteria | L |
| NZ_AP014839.1_00100 | 287.DR97_1831 | 2.3e-246 | 857.8 | Pseudomonas aeruginosa group | S |
| NZ_AP017302.1_ICE_00023 | 287.DR97_1748 | 3.3e-152 | 544.3 | Gammaproteobacteria | S |
| NZ_AP017302.1_ICE_00027 | 1415630.U771_05000 | 1.3e-132 | 480.3 | Proteobacteria | - |
| NZ_AP017302.1_ICE_00030 | 443144.GM21_3259 | 5.8e-08 | 65.9 | Desulfuromonadales | M |
| NZ_AP017302.1_ICE_00031 | 1122992.CBQQ010000043_gene513 | 3.5e-07 | 62.0 | Bacteroidia | V |
| NZ_AP017302.1_ICE_00034 | 287.DR97_1756 | 3.5e-23 | 114.0 | Pseudomonas aeruginosa group | S |
| NZ_AP017302.1_ICE_00035 | 1333856.L686_06380 | 2.7e-277 | 960.7 | Pseudomonas stutzeri group | S |
| NZ_AP017302.1_ICE_00036 | 1123020.AUIE01000026_gene4841 | 3.1e-56 | 224.2 | Pseudomonas aeruginosa group | L |
| NZ_AP017302.1_ICE_00037 | 1123020.AUIE01000026_gene4840 | 7.2e-53 | 213.0 | Pseudomonas aeruginosa group | L |
| NZ_AP017302.1_ICE_00039 | 1215092.PA6_105_00010 | 1.4e-122 | 445.7 | Pseudomonas aeruginosa group | L |
| NZ_AP017302.1_ICE_00040 | 1026882.MAMP_02736 | 2,00E-57 | 233.0 | Thiotrichales | Q |
| NZ_AP017302.1_ICE_00041 | 706587.Desti_4001 | 1.3e-10 | 73.6 | Syntrophobacterales | - |
| NZ_AP017302.1_ICE_00043 | 706587.Desti_4001 | 4.3e-11 | 75.1 | Syntrophobacterales | - |
| NZ_AP017302.1_ICE_00044 | 1031711.RSPO_c00800 | 1.1e-165 | 589.7 | Betaproteobacteria | M |
| NZ_AP017302.1_ICE_00045 | 1294143.H681_09335 | 1,00E-193 | 682.9 | Gammaproteobacteria | M |
| NZ_AP017302.1_ICE_00046 | 1215092.PA6_052_00140 | 0.0 | 1157.5 | Pseudomonas aeruginosa group | V |
| NZ_AP017302.1_ICE_00064 | 287.DR97_1822 | 2.1e-152 | 545.0 | Gammaproteobacteria | NU |
| NZ_AP017302.1_ICE_00069 | 1245471.PCA10_39000 | 1.9e-129 | 468.8 | Gammaproteobacteria | S |
| NZ_AP017302.1_ICE_00070 | 1245471.PCA10_39010 | 2.2e-133 | 481.9 | Pseudomonas aeruginosa group | K |
| NZ_AP017302.1_ICE_00071 | 32042.PstZobell_08057 | 1.1e-24 | 118.6 | Pseudomonas stutzeri group | L |
| NZ_AP017302.1_ICE_00072 | 1419583.V466_28870 | 7.2e-147 | 526.9 | Pseudomonas fluorescens group | C |
| NZ_AP017302.1_ICE_00074 | 1437882.AZRU01000172_gene1183 | 4.8e-20 | 102.8 | Gammaproteobacteria | L |
| NZ_AP017302.1_ICE_00075 | 1437882.AZRU01000172_gene1183 | 5,00E-98 | 364.0 | Gammaproteobacteria | L |
| NZ_AP017302.1_ICE_00076 | 1437882.AZRU01000172_gene1182 | 2.5e-37 | 161.0 | Gammaproteobacteria | L |
| NZ_AP017302.1_ICE_00077 | 1470593.BW43_01790 | 6.1e-80 | 304.3 | Gammaproteobacteria | K |
| NZ_AP017302.1_ICE_00079 | 1419583.V466_11030 | 4.9e-88 | 331.6 | Pseudomonas fluorescens group | EGP |
| NZ_AP017302.1_ICE_00080 | 598467.BrE312_0537 | 4.2e-119 | 434.9 | Gammaproteobacteria | V |
| NZ_AP017302.1_ICE_00081 | 311403.Arad_7251 | 1.2e-79 | 302.8 | Alphaproteobacteria | Q |
| NZ_AP017302.1_ICE_00082 | 267608.RSp0444 | 2.2e-78 | 298.9 | Burkholderiaceae | S |
| NZ_AP017302.1_ICE_00083 | 1123504.JQKD01000108_gene5212 | 6,00E-66 | 257.3 | Comamonadaceae | O |
| NZ_AP017302.1_ICE_00084 | 598467.BrE312_0530 | 4.9e-114 | 417.5 | Gammaproteobacteria | S |
| NZ_AP017302.1_ICE_00085 | 1042209.HK44_004480 | 1.1e-07 | 62.0 | Proteobacteria | - |
| NZ_AP017302.1_ICE_00086 | 287.DR97_3675 | 9.9e-277 | 958.7 | Pseudomonas aeruginosa group | P |
| NZ_AP017302.1_ICE_00095 | 381666.H16_A3231 | 1.2e-200 | 706.8 | Bacteria | - |
| NZ_AP017302.1_ICE_00096 | 85643.Tmz1t_0482 | 0.0 | 1082.4 | Rhodocyclales | S |
| NZ_AP017302.1_ICE_00097 | 1504981.KO116_0757 | 2.3e-163 | 581.6 | Gammaproteobacteria | S |
| NZ_AP017302.1_ICE_00099 | 287.DR97_1841 | 1.5e-239 | 835.1 | Gammaproteobacteria | L |
| NZ_CP007147.1_00003 | 629265.PMA4326_23696 | 2.3e-80 | 306.2 | Proteobacteria | S |
| NZ_CP007147.1_00013 | 1196835.A458_15110 | 1.9e-121 | 442.2 | Pseudomonas stutzeri group | L |
| NZ_CP007147.1_00014 | 555778.Hneap_1630 | 6.6e-172 | 610.5 | Chromatiales | K |
| NZ_CP007147.1_00027 | 1151116.Q7S_07185 | 6.4e-117 | 427.9 | Gammaproteobacteria | L |
| NZ_CP007147.1_00028 | 1345023.M467_13045 | 1,00E-19 | 103.2 | Bacilli | - |
| NZ_CP007147.1_00029 | 1121889.AUDM01000018_gene1257 | 1.2e-50 | 206.8 | Flavobacterium | - |
| NZ_CP007147.1_00030 | 1388763.O165_001285 | 2.6e-22 | 111.3 | Pseudomonas putida group | - |
| NZ_CP007147.1_00043 | 287.DR97_1782 | 3.4e-56 | 224.2 | Gammaproteobacteria | - |
| NZ_CP007147.1_00056 | 287.DR97_5228 | 1.1e-73 | 282.7 | Pseudomonas aeruginosa group | L |
| NZ_CP007147.1_00057 | 287.DR97_5229 | 1.2e-35 | 155.6 | Pseudomonas aeruginosa group | L |
| NZ_CP008856.2_00057 | 1005395.CSV86_02202 | 1.6e-26 | 125.2 | Gammaproteobacteria | L |
| NZ_CP008856.2_00067 | 379731.PST_3445 | 1.8e-92 | 347.1 | Gammaproteobacteria | - |
| NZ_CP008856.2_00068 | 379731.PST_3446 | 3.4e-108 | 399.4 | Gammaproteobacteria | S |
| NZ_CP008856.2_00069 | 1437882.AZRU01000053_gene3683 | 6.8e-34 | 149.4 | Pseudomonas aeruginosa group | L |
| NZ_CP008856.2_00070 | 477228.YO5_01706 | 2.3e-132 | 478.4 | Pseudomonas stutzeri group | L |
| NZ_CP008856.2_00071 | 1144319.PMI16_04806 | 9.5e-120 | 436.4 | Oxalobacteraceae | L |
| NZ_CP008856.2_00072 | 1301098.PKB_1314 | 1.3e-31 | 141.7 | Gammaproteobacteria | S |
| NZ_CP008856.2_00073 | 1301098.PKB_1313 | 4.1e-206 | 723.8 | Gammaproteobacteria | C |
| NZ_CP008856.2_00075 | 1301098.PKB_1311 | 1.2e-99 | 369.4 | Bacteria | Q |
| NZ_CP008856.2_00079 | 1301098.PKB_1307 | 6.9e-42 | 176.8 | Gammaproteobacteria | - |
| NZ_CP008856.2_00102 | 287.DR97_1825 | 0.0 | 1981.1 | Gammaproteobacteria | U |
| NZ_CP008856.2_00114 | 287.DR97_1835 | 4,00E-53 | 214.2 | Proteobacteria | S |
| NZ_CP008856.2_00115 | 1144342.PMI40_00513 | 1.4e-128 | 466.8 | Proteobacteria | L |
| NZ_CP008856.2_00117 | 1144342.PMI40_00511 | 0.0 | 1647.1 | Proteobacteria | L |
| NZ_CP008856.2_00118 | 1031711.RSPO_c00010 | 6.2e-286 | 989.9 | Proteobacteria | - |
| NZ_CP008856.2_00119 | 999541.bgla_1g36730 | 1.2e-62 | 247.3 | Betaproteobacteria | S |
| NZ_CP008856.2_00121 | 287.DR97_1838 | 8.2e-45 | 186.0 | Pseudomonas aeruginosa group | L |
| NZ_CP008856.2_00123 | 1207055.C100_12290 | 1.3e-07 | 62.8 | Alphaproteobacteria | - |
| NZ_CP008856.2_00124 | 357276.EL88_18845 | 5.3e-41 | 174.1 | Bacteroidaceae | - |
| NZ_CP008856.2_00125 | 1046627.BZARG_2836 | 5.8e-25 | 120.9 | Bacteria | - |
| NZ_CP008857.1_00103 | 287.DR97_1841 | 1.1e-146 | 526.6 | Gammaproteobacteria | L |
| NZ_CP008861.1_00047 | 287.DR97_1783 | 2.3e-102 | 378.3 | Gammaproteobacteria | - |
| NZ_CP008861.1_00083 | 1182590.BN5_00834 | 1.6e-143 | 515.4 | Pseudomonas aeruginosa group | L |
| NZ_CP008861.1_00084 | 1182590.BN5_04409 | 2.8e-290 | 1003.8 | Pseudomonas aeruginosa group | L |
| NZ_CP008861.1_00085 | 287.DR97_1750 | 1.1e-104 | 386.0 | Pseudomonas aeruginosa group | G |
| NZ_CP008861.1_00087 | 1182590.BN5_04409 | 2.8e-290 | 1003.8 | Pseudomonas aeruginosa group | L |
| NZ_CP008861.1_00088 | 1182590.BN5_00834 | 1.6e-143 | 515.4 | Pseudomonas aeruginosa group | L |
| NZ_CP008861.1_00089 | 287.DR97_1727 | 6.6e-36 | 156.0 | Gammaproteobacteria | - |
| NZ_CP008861.1_00090 | 287.DR97_1726 | 5.8e-123 | 446.8 | Pseudomonas aeruginosa group | - |
| NZ_CP008861.1_00093 | 287.DR97_1723 | 2,00E-155 | 555.1 | Pseudomonas aeruginosa group | D |
| NZ_CP008862.2_00072 | 246196.MSMEI_1366 | 2.5e-09 | 68.2 | Mycobacteriaceae | - |
| NZ_CP008862.2_00073 | 399739.Pmen_0427 | 1.6e-114 | 418.7 | Pseudomonas aeruginosa group | L |
| NZ_CP008862.2_00074 | 663932.KB902575_gene2417 | 5.3e-129 | 468.0 | Alphaproteobacteria | E |
| NZ_CP008862.2_00075 | 1323663.AROI01000008_gene2466 | 3.2e-100 | 371.7 | Proteobacteria | Q |
| NZ_CP008862.2_00076 | 1144664.F973_02845 | 2.6e-22 | 112.5 | Moraxellaceae | K |
| NZ_CP008862.2_00078 | 246196.MSMEI_1366 | 2.5e-09 | 68.2 | Mycobacteriaceae | - |
| NZ_CP008862.2_00079 | 399739.Pmen_0427 | 1.6e-114 | 418.7 | Pseudomonas aeruginosa group | L |
| NZ_CP008862.2_00096 | 246196.MSMEI_1366 | 2.5e-09 | 68.2 | Mycobacteriaceae | - |
| NZ_CP008862.2_00097 | 399739.Pmen_0427 | 1.6e-114 | 418.7 | Pseudomonas aeruginosa group | L |
| NZ_CP008863.1_00002 | 287.DR97_1841 | 4.9e-132 | 477.2 | Gammaproteobacteria | L |
| NZ_CP008865.2_4Mb_00059 | 666684.AfiDRAFT_1384 | 3.9e-62 | 245.0 | Alphaproteobacteria | - |
| NZ_CP008865.2_4Mb_00060 | 1114970.PSF113_4943 | 2.7e-48 | 199.1 | Gammaproteobacteria | - |
| NZ_CP008865.2_4Mb_00062 | 748247.AZKH_1346 | 1.3e-282 | 979.2 | Proteobacteria | L |
| NZ_CP008865.2_4Mb_00063 | 748247.AZKH_1345 | 0.0 | 1450.6 | Rhodocyclales | L |
| NZ_CP008865.2_4Mb_00064 | 397945.Aave_0577 | 9.7e-76 | 290.8 | Betaproteobacteria | L |
| NZ_CP008865.2_4Mb_00079 | 1301098.PKB_1306 | 7.3e-14 | 82.0 | Gammaproteobacteria | L |
| NZ_CP008865.2_4Mb_00106 | 379731.PST_0255 | 8.2e-193 | 679.5 | Pseudomonas stutzeri group | L |
| NZ_CP008865.2_4Mb_00107 | 379731.PST_0255 | 8.2e-193 | 679.5 | Pseudomonas stutzeri group | L |
| NZ_CP008865.2_4Mb_00109 | 379731.PST_0255 | 8.2e-193 | 679.5 | Pseudomonas stutzeri group | L |
| NZ_CP008865.2_5Mb_00094 | 287.DR97_1833 | 5,00E-279 | 966.5 | Gammaproteobacteria | S |
| NZ_CP008866.2_00110 | 287.DR97_1836 | 4.2e-172 | 610.5 | Gammaproteobacteria | - |
| NZ_CP008869.2_00050 | 1114970.PSF113_0022 | 1.6e-44 | 185.3 | Pseudomonas fluorescens group | L |
| NZ_CP008869.2_00051 | 1182590.BN5_02987 | 1.3e-54 | 218.8 | Pseudomonas aeruginosa group | L |
| NZ_CP008869.2_00052 | 321846.PS417_24960 | 1.9e-257 | 894.8 | Gammaproteobacteria | S |
| NZ_CP008869.2_00053 | 1415630.U771_00025 | 2.7e-182 | 644.4 | Gammaproteobacteria | L |
| NZ_CP008869.2_00054 | 1268068.PG5_01080 | 5.6e-239 | 833.2 | Gammaproteobacteria | I |
| NZ_CP008869.2_00069 | 1182590.BN5_01212 | 5,00E-240 | 836.6 | Pseudomonas aeruginosa group | L |
| NZ_CP008869.2_00072 | 287.DR97_6328 | 8.3e-57 | 227.6 | Bacteria | CH |
| NZ_CP008869.2_00105 | 265072.Mfla_1495 | 6.5e-97 | 360.1 | Nitrosomonadales | L |
| NZ_CP008871.2_00004 | 287.DR97_1726 | 7.8e-81 | 307.0 | Pseudomonas aeruginosa group | - |
| NZ_CP008871.2_00057 | 379731.PST_3351 | 3.5e-63 | 247.3 | Gammaproteobacteria | S |
| NZ_CP008871.2_00058 | 379731.PST_3352 | 0.0 | 1461.8 | Gammaproteobacteria | L |
| NZ_CP008871.2_00059 | 379731.PST_3353 | 1.2e-196 | 692.2 | Gammaproteobacteria | L |
| NZ_CP008871.2_00060 | 440512.C211_02391 | 5.3e-74 | 283.5 | Gammaproteobacteria | - |
| NZ_CP008871.2_00061 | 1226994.AMZB01000062_gene3824 | 4.7e-311 | 1072.8 | Pseudomonas aeruginosa group | C |
| NZ_CP008871.2_00062 | 440512.C211_02401 | 2.4e-77 | 294.7 | Gammaproteobacteria | S |
| NZ_CP008871.2_00063 | 1226994.AMZB01000062_gene3826 | 5.3e-38 | 163.3 | Pseudomonas aeruginosa group | P |
| NZ_CP008871.2_00064 | 440512.C211_02411 | 4.2e-56 | 223.8 | Gammaproteobacteria | P |
| NZ_CP008871.2_00065 | 1226994.AMZB01000062_gene3828 | 1.1e-68 | 265.8 | Pseudomonas aeruginosa group | K |
| NZ_CP008871.2_00066 | 440512.C211_02426 | 8.7e-53 | 213.0 | Gammaproteobacteria | - |
| NZ_CP008871.2_00067 | 1301098.PKB_1306 | 2,00E-306 | 1057.7 | Gammaproteobacteria | L |
| NZ_CP008871.2_00068 | 1005395.CSV86_15400 | 2.1e-88 | 331.6 | Pseudomonas putida group | L |
| NZ_CP008871.2_00069 | 1005395.CSV86_15405 | 1.2e-213 | 748.8 | Pseudomonas putida group | L |
| NZ_CP008871.2_00076 | 287.DR97_1784 | 3.5e-103 | 380.9 | Gammaproteobacteria | S |
| NZ_CP008871.2_00108 | 287.DR97_1831 | 7.8e-247 | 859.4 | Pseudomonas aeruginosa group | S |
| NZ_CP008873.1_00085 | 205918.Psyr_1521 | 1.9e-33 | 149.4 | Pseudomonas syringae group | - |
| NZ_CP010555.1_00011 | 158500.BV97_05657 | 2.3e-96 | 360.1 | Sphingomonadales | L |
| NZ_CP010555.1_00012 | 1051632.TPY_2178 | 2.2e-59 | 236.5 | Bacteria | L |
| NZ_CP010555.1_00105 | 287.DR97_1727 | 2.3e-130 | 471.5 | Gammaproteobacteria | - |
| NZ_CP011317.1_00022 | 287.DR97_1741 | 1.6e-278 | 964.9 | Gammaproteobacteria | K |
| NZ_CP011317.1_00031 | 1380387.JADM01000034_gene1737 | 5.5e-69 | 267.7 | Gammaproteobacteria | - |
| NZ_CP011317.1_00045 | 228410.NE0492 | 2.1e-60 | 239.6 | Nitrosomonadales | S |
| NZ_CP011317.1_00061 | 930169.B5T_03182 | 4.1e-13 | 81.3 | Bacteria | M |
| NZ_CP011317.1_00062 | 930169.B5T_03182 | 2.4e-61 | 243.0 | Bacteria | M |
| NZ_CP011317.1_00089 | 1005395.CSV86_18402 | 2.4e-09 | 68.6 | Pseudomonas putida group | - |
| NZ_CP011317.1_00090 | 287.DR97_1833 | 2.3e-263 | 914.4 | Gammaproteobacteria | S |
| NZ_CP011317.1_00092 | 287.DR97_1836 | 2.1e-168 | 598.2 | Gammaproteobacteria | - |
| NZ_CP011317.1_00093 | 287.DR97_1837 | 7.8e-38 | 162.5 | Gammaproteobacteria | - |
| NZ_CP011317.1_00094 | 287.DR97_1840 | 0.0 | 1185.2 | Gammaproteobacteria | S |
| NZ_CP011857.1_00005 | 287.DR97_1727 | 2.3e-130 | 471.5 | Gammaproteobacteria | - |
| NZ_CP011857.1_00025 | 287.DR97_1746 | 2.1e-134 | 485.0 | Gammaproteobacteria | S |
| NZ_CP011857.1_00084 | 287.DR97_1797 | 0.0 | 1963.3 | Pseudomonas aeruginosa group | L |
| NZ_CP011857.1_00087 | 1182590.BN5_04396 | 8.5e-75 | 286.2 | Pseudomonas aeruginosa group | K |
| NZ_CP011857.1_00088 | 1182590.BN5_04397 | 9,00E-59 | 232.6 | Pseudomonas aeruginosa group | P |
| NZ_CP011857.1_00089 | 1182590.BN5_04398 | 1.1e-38 | 165.6 | Pseudomonas aeruginosa group | P |
| NZ_CP011857.1_00090 | 1182590.BN5_04399 | 9.8e-36 | 155.6 | Pseudomonas aeruginosa group | S |
| NZ_CP011857.1_00091 | 1276756.AUEX01000036_gene1212 | 2.2e-304 | 1050.8 | Comamonadaceae | C |
| NZ_CP011857.1_00092 | 1182590.BN5_04401 | 9.4e-59 | 232.6 | Pseudomonas aeruginosa group | K |
| NZ_CP011857.1_00093 | 1182590.BN5_04402 | 8.5e-37 | 159.1 | Pseudomonas aeruginosa group | S |
| NZ_CP011857.1_00094 | 1182590.BN5_04403 | 3.4e-109 | 401.0 | Pseudomonas aeruginosa group | L |
| NZ_CP011857.1_00095 | 1276756.AUEX01000032_gene1216 | 3.8e-245 | 853.6 | Comamonadaceae | S |
| NZ_CP011857.1_00096 | 1276756.AUEX01000032_gene1217 | 6.9e-167 | 593.2 | Comamonadaceae | O |
| NZ_CP011857.1_00097 | 1182590.BN5_04406 | 0.0 | 1114.8 | Pseudomonas aeruginosa group | L |
| NZ_CP011857.1_00099 | 287.DR97_1813 | 2.7e-140 | 504.6 | Pseudomonas aeruginosa group | IQ |
| NZ_CP013245.1_00020 | 95619.PM1_0225240 | 3.6e-76 | 291.2 | Gammaproteobacteria | NU |
| NZ_CP013245.1_00021 | 95619.PM1_0225235 | 5.4e-82 | 310.8 | Gammaproteobacteria | M |
| NZ_CP013245.1_00022 | 95619.PM1_0225230 | 0.0 | 1236.9 | Gammaproteobacteria | NU |
| NZ_CP013245.1_00023 | 95619.PM1_0225220 | 2.1e-47 | 196.4 | Bacteria | NU |
| NZ_CP013245.1_00024 | 1149133.ppKF707_1260 | 4,00E-58 | 230.7 | Pseudomonas aeruginosa group | L |
| NZ_CP013245.1_00027 | 95619.PM1_0225205 | 8,00E-61 | 240.0 | Proteobacteria | K |
| NZ_CP013245.1_00028 | 287.DR97_1828 | 1.5e-62 | 245.7 | Pseudomonas aeruginosa group | O |
| NZ_CP013245.1_00033 | 287.DR97_1822 | 1,00E-154 | 552.7 | Gammaproteobacteria | NU |
| NZ_CP013245.1_00039 | 351746.Pput_3754 | 7.7e-28 | 129.8 | Gammaproteobacteria | M |
| NZ_CP013245.1_00040 | 351746.Pput_3752 | 9.4e-102 | 376.3 | Gammaproteobacteria | K |
| NZ_CP013245.1_00041 | 351746.Pput_3751 | 0.0 | 1804.3 | Gammaproteobacteria | T |
| NZ_CP013245.1_00042 | 351746.Pput_3750 | 4,00E-215 | 753.8 | Gammaproteobacteria | T |
| NZ_CP013245.1_00043 | 351746.Pput_3749 | 0.0 | 1663.3 | Pseudomonas putida group | T |
| NZ_CP013245.1_00044 | 69328.PVLB_07460 | 1.3e-39 | 169.1 | Bacteria | NU |
| NZ_CP013245.1_00052 | 379731.PST_3745 | 0.0 | 1096.6 | Gammaproteobacteria | L |
| NZ_CP013245.1_00053 | 379731.PST_3746 | 8.8e-103 | 379.8 | Gammaproteobacteria | S |
| NZ_CP013245.1_00054 | 379731.PST_3747 | 4.9e-262 | 910.2 | Gammaproteobacteria | S |
| NZ_CP013245.1_00055 | 379731.PST_3748 | 2.1e-149 | 535.0 | Gammaproteobacteria | L |
| NZ_CP013245.1_00056 | 379731.PST_3749 | 2,00E-80 | 305.4 | Gammaproteobacteria | L |
| NZ_CP013245.1_00058 | 379731.PST_3750 | 3.7e-36 | 157.1 | Gammaproteobacteria | L |
| NZ_CP013245.1_00059 | 379731.PST_3751 | 1.9e-41 | 174.9 | Gammaproteobacteria | L |
| NZ_CP014866.1_ICE_00029 | 1221522.B723_18995 | 8.7e-25 | 120.2 | Pseudomonas fluorescens group | - |
| NZ_CP014866.1_ICE_00061 | 287.DR97_6328 | 6.4e-57 | 228.0 | Bacteria | CH |
| NZ_CP014866.1_ICE_00074 | 1395571.TMS3_0120570 | 4.4e-73 | 281.2 | Gammaproteobacteria | - |
| NZ_CP014866.1_ICE_00082 | 287.DR97_1795 | 4.5e-39 | 167.5 | Pseudomonas aeruginosa group | L |
| NZ_CP014866.1_ICE_00087 | 287.DR97_1819 | 7.1e-51 | 206.5 | Gammaproteobacteria | S |
| NZ_CP014866.1_ICE_00101 | 1388763.O165_001190 | 8.5e-09 | 66.6 | Pseudomonas putida group | - |
| NZ_CP014866.1_ICE_00106 | 1215092.PA6_005_02190 | 4.2e-264 | 917.1 | Gammaproteobacteria | L |
| NZ_CP014948.1_00027 | 930169.B5T_02144 | 1.7e-13 | 82.8 | Gammaproteobacteria | - |
| NZ_CP014948.1_00054 | 379731.PST_3747 | 8.3e-265 | 919.5 | Gammaproteobacteria | S |
| NZ_CP014948.1_00055 | 379731.PST_3746 | 6.1e-104 | 383.6 | Gammaproteobacteria | S |
| NZ_CP014948.1_00056 | 379731.PST_3745 | 0.0 | 1098.2 | Gammaproteobacteria | L |
| NZ_CP014999.1_00031 | 287.DR97_1756 | 9.3e-16 | 88.6 | Pseudomonas aeruginosa group | S |
| NZ_CP014999.1_00060 | 379731.PST_3747 | 2.4e-264 | 917.9 | Gammaproteobacteria | S |
| NZ_CP014999.1_00064 | 1437882.AZRU01000002_gene2315 | 2.7e-81 | 308.1 | Pseudomonas aeruginosa group | S |
| NZ_CP014999.1_00067 | 287.DR97_3708 | 3.2e-124 | 451.1 | Pseudomonas aeruginosa group | S |
| NZ_CP014999.1_00068 | 1301098.PKB_0744 | 1.9e-83 | 315.5 | Gammaproteobacteria | S |
| NZ_CP014999.1_00070 | 151599.Q5ZR08_9CAUD | 6.2e-66 | 256.5 | Caudovirales | - |
| NZ_CP014999.1_00071 | 287.DR97_3713 | 5.2e-33 | 146.7 | Pseudomonas aeruginosa group | - |
| NZ_CP014999.1_00072 | 287.DR97_3714 | 2.9e-46 | 191.0 | Pseudomonas aeruginosa group | - |
| NZ_CP014999.1_00073 | 151599.Q5ZR05_9CAUD | 1.4e-183 | 649.0 | Caudovirales | L |
| NZ_CP014999.1_00074 | 1225792.J9STP5_9CAUD | 2.7e-215 | 755.0 | Caudovirales | S |
| NZ_CP014999.1_00075 | 151599.Q5ZR03_9CAUD | 8.6e-149 | 533.1 | Caudovirales | - |
| NZ_CP014999.1_00076 | 1437882.AZRU01000002_gene2327 | 5.6e-28 | 130.2 | Gammaproteobacteria | K |
| NZ_CP014999.1_00077 | 151599.Q5ZR01_9CAUD | 5.6e-91 | 340.1 | Caudovirales | - |
| NZ_CP014999.1_00079 | 287.DR97_3721 | 1.3e-29 | 135.6 | Pseudomonas aeruginosa group | - |
| NZ_CP014999.1_00080 | 287.DR97_3722 | 1.2e-85 | 322.4 | Gammaproteobacteria | - |
| NZ_CP014999.1_00082 | 1225792.J9STQ9_9CAUD | 1.7e-45 | 188.3 | Caudovirales | S |
| NZ_CP014999.1_00084 | 1225792.J9SH25_9CAUD | 1.1e-118 | 432.6 | Caudovirales | S |
| NZ_CP014999.1_00086 | 1225791.J9SVN7_9CAUD | 3.3e-96 | 357.8 | Caudovirales | - |
| NZ_CP014999.1_00087 | 151599.Q5ZQY8_9CAUD | 5.7e-62 | 243.4 | Caudovirales | - |
| NZ_CP014999.1_00088 | 1225791.J9SN59_9CAUD | 1.2e-51 | 208.8 | Caudovirales | - |
| NZ_CP014999.1_00089 | 151599.Q5ZQY6_9CAUD | 2.3e-91 | 341.7 | Caudovirales | S |
| NZ_CP014999.1_00090 | 1301098.PKB_0769 | 2,00E-248 | 864.8 | Gammaproteobacteria | S |
| NZ_CP014999.1_00091 | 151599.Q5ZQY4_9CAUD | 2.7e-282 | 977.2 | Siphoviridae | - |
| NZ_CP014999.1_00092 | 1301098.PKB_0771 | 1.1e-146 | 526.6 | Gammaproteobacteria | L |
| NZ_CP014999.1_00093 | 151599.Q5ZQY1_9CAUD | 3.6e-105 | 387.5 | Siphoviridae | S |
| NZ_CP014999.1_00094 | 151599.Q5ZQY0_9CAUD | 4.7e-182 | 644.0 | Caudovirales | S |
| NZ_CP014999.1_00095 | 151599.Q5ZQX8_9CAUD | 1,00E-55 | 222.6 | Caudovirales | - |
| NZ_CP014999.1_00096 | 151599.Q5ZQX7_9CAUD | 3.1e-175 | 620.9 | Caudovirales | - |
| NZ_CP014999.1_00098 | 1397284.AYMN01000002_gene2281 | 1.9e-22 | 111.7 | Gammaproteobacteria | - |
| NZ_CP014999.1_00099 | 1301098.PKB_0778 | 2.4e-60 | 238.4 | Gammaproteobacteria | S |
| NZ_CP014999.1_00100 | 1301098.PKB_0779 | 1.1e-54 | 219.5 | Gammaproteobacteria | - |
| NZ_CP014999.1_00102 | 287.DR97_3740 | 1.7e-91 | 342.4 | Pseudomonas aeruginosa group | - |
| NZ_CP014999.1_00103 | 1437882.AZRU01000002_gene2352 | 1.5e-41 | 176.0 | Pseudomonas aeruginosa group | - |
| NZ_CP014999.1_00105 | 151599.Q5ZQW7_9CAUD | 2.9e-153 | 547.7 | Siphoviridae | S |
| NZ_CP014999.1_00106 | 151599.Q5ZQW6_9CAUD | 0.0 | 1684.5 | Siphoviridae | S |
| NZ_CP014999.1_00107 | 389469.A0SMQ2_9CAUD | 1.7e-179 | 635.2 | Siphoviridae | - |
| NZ_CP014999.1_00108 | 287.DR97_3744 | 3.7e-176 | 624.0 | Gammaproteobacteria | - |
| NZ_CP014999.1_00109 | 1225791.J9STL4_9CAUD | 0.0 | 1102.8 | Siphoviridae | - |
| NZ_CP014999.1_00110 | 287.DR97_3746 | 7.1e-163 | 579.7 | Pseudomonas aeruginosa group | S |
| NZ_CP014999.1_00111 | 287.DR97_3747 | 9.2e-36 | 155.6 | Pseudomonas aeruginosa group | - |
| NZ_CP014999.1_00112 | 151599.Q5ZQV9_9CAUD | 0.0 | 1441.0 | Siphoviridae | S |
| NZ_CP014999.1_00113 | 1461694.ATO9_22105 | 8.7e-15 | 87.8 | Oceanicola | S |
| NZ_CP014999.1_00114 | 1500301.JQMF01000003_gene4252 | 2.4e-20 | 104.8 | Rhizobiaceae | - |
| NZ_CP014999.1_00117 | 379731.PST_3745 | 3,00E-182 | 644.8 | Gammaproteobacteria | L |
| NZ_CP014999.1_00119 | 1301098.PKB_1315 | 5.8e-192 | 676.8 | Gammaproteobacteria | L |
| NZ_CP014999.1_00158 | 1388763.O165_001190 | 3.3e-07 | 61.2 | Pseudomonas putida group | - |
| NZ_CP014999.1_00159 | 287.DR97_1833 | 3.5e-272 | 943.7 | Gammaproteobacteria | S |
| NZ_CP014999.1_00166 | 69328.PVLB_13910 | 2.3e-228 | 798.1 | Gammaproteobacteria | C |
| NZ_CP014999.1_00167 | 1136138.JH604622_gene1834 | 4.6e-182 | 644.0 | Gammaproteobacteria | S |
| NZ_CP014999.1_00168 | 216595.PFLU_3251 | 3.4e-32 | 145.2 | Bacteria | M |
| NZ_CP014999.1_00169 | 1177181.T9A_02552 | 1.1e-26 | 126.3 | Gammaproteobacteria | O |
| NZ_CP014999.1_00170 | 1298593.TOL_0865 | 6.6e-10 | 72.4 | Gammaproteobacteria | - |
| NZ_CP014999.1_00171 | 379731.PST_3407 | 8.1e-111 | 406.4 | Pseudomonas stutzeri group | L |
| NZ_CP014999.1_00174 | 68570.DC74_940 | 1.2e-39 | 169.5 | Bacteria | S |
| NZ_CP014999.1_00175 | 339670.Bamb_6451 | 7.5e-97 | 360.5 | Burkholderiaceae | S |
| NZ_CP014999.1_00176 | 318424.EU78_00430 | 2.6e-43 | 182.2 | Mycobacteriaceae | K |
| NZ_CP015001.1_00125 | 1437882.AZRU01000002_gene2363 | 1.1e-139 | 502.7 | Pseudomonas aeruginosa group | L |
| NZ_CP015001.1_00126 | 35343.Q9ZXK5_9CAUD | 2.1e-31 | 142.1 | Myoviridae | S |
| NZ_CP015001.1_00127 | 287.DR97_1360 | 4.2e-124 | 452.2 | Pseudomonas aeruginosa group | S |
| NZ_CP015001.1_00128 | 930166.CD58_18225 | 1.9e-58 | 232.3 | Gammaproteobacteria | S |
| NZ_CP015001.1_00129 | 930166.CD58_18230 | 8.5e-135 | 486.9 | Gammaproteobacteria | S |
| NZ_CP015001.1_00130 | 930166.CD58_18235 | 1.7e-47 | 195.3 | Gammaproteobacteria | S |
| NZ_CP015001.1_00131 | 930166.CD58_18245 | 4.3e-60 | 237.7 | Gammaproteobacteria | S |
| NZ_CP015001.1_00132 | 1005395.CSV86_24814 | 1.3e-103 | 383.3 | Gammaproteobacteria | S |
| NZ_CP015001.1_00133 | 1005395.CSV86_24809 | 2.5e-19 | 100.9 | Gammaproteobacteria | S |
| NZ_CP015001.1_00134 | 587753.EY04_15280 | 9.8e-46 | 190.7 | Gammaproteobacteria | S |
| NZ_CP015001.1_00135 | 587753.EY04_15275 | 4.3e-193 | 681.8 | Proteobacteria | G |
| NZ_CP015001.1_00136 | 1005395.CSV86_24784 | 2.3e-21 | 108.2 | Gammaproteobacteria | S |
| NZ_CP015001.1_00138 | 243265.plu3450 | 5.7e-18 | 97.4 | Gammaproteobacteria | - |
| NZ_CP015001.1_00139 | 930166.CD58_18315 | 9.3e-76 | 289.7 | Gammaproteobacteria | S |
| NZ_CP015001.1_00140 | 1005395.CSV86_24769 | 5.1e-209 | 733.8 | Gammaproteobacteria | S |
| NZ_CP015001.1_00141 | 1005395.CSV86_24764 | 5.8e-09 | 66.6 | Gammaproteobacteria | - |
| NZ_CP015001.1_00142 | 1005395.CSV86_24759 | 4.1e-62 | 244.2 | Gammaproteobacteria | S |
| NZ_CP015001.1_00143 | 1005395.CSV86_24754 | 9.7e-63 | 246.1 | Gammaproteobacteria | S |
| NZ_CP015001.1_00144 | 1005395.CSV86_24749 | 1.9e-37 | 161.8 | Gammaproteobacteria | S |
| NZ_CP015001.1_00146 | 1005395.CSV86_24739 | 2.3e-154 | 551.6 | Gammaproteobacteria | - |
| NZ_CP015001.1_00147 | 587753.EY04_15210 | 3.7e-146 | 524.6 | Gammaproteobacteria | - |
| NZ_CP015001.1_00148 | 1005395.CSV86_24729 | 2.9e-63 | 248.1 | Gammaproteobacteria | S |
| NZ_CP015001.1_00149 | 1005395.CSV86_24724 | 9.3e-123 | 446.4 | Gammaproteobacteria | L |
| NZ_CP015001.1_00150 | 1005395.CSV86_24719 | 2.9e-255 | 887.5 | Gammaproteobacteria | S |
| NZ_CP015001.1_00151 | 1005395.CSV86_24714 | 1.5e-281 | 974.9 | Gammaproteobacteria | S |
| NZ_CP015001.1_00152 | 257310.BB3633 | 5.3e-45 | 187.6 | Proteobacteria | S |
| NZ_CP015001.1_00153 | 930166.CD58_18400 | 5.6e-38 | 163.3 | Gammaproteobacteria | K |
| NZ_CP015001.1_00154 | 1005395.CSV86_24699 | 1.4e-33 | 148.7 | Gammaproteobacteria | - |
| NZ_CP015001.1_00155 | 1051985.l11_04410 | 3.6e-08 | 63.9 | Neisseriales | T |
| NZ_CP015001.1_00156 | 1225791.J9SVN7_9CAUD | 3.4e-45 | 188.3 | Caudovirales | - |
| NZ_CP015001.1_00159 | 587753.EY04_15155 | 1.5e-72 | 278.9 | Gammaproteobacteria | - |
| NZ_CP015001.1_00160 | 930166.CD58_18430 | 3.7e-20 | 105.1 | Proteobacteria | - |
| NZ_CP015001.1_00161 | 587753.EY04_15140 | 1.8e-42 | 178.7 | Gammaproteobacteria | K |
| NZ_CP015001.1_00162 | 930166.CD58_18445 | 6.2e-29 | 132.9 | Proteobacteria | - |
| NZ_CP015001.1_00163 | 243365.CV_2153 | 3,00E-44 | 184.9 | Neisseriales | - |
| NZ_CP015001.1_00164 | 287.DR97_3719 | 9.7e-34 | 149.1 | Pseudomonas aeruginosa group | - |
| NZ_CP015001.1_00165 | 287.DR97_3718 | 1.6e-38 | 165.2 | Bacteria | K |
| NZ_CP015001.1_00166 | 287.DR97_3717 | 4.5e-79 | 301.6 | Pseudomonas aeruginosa group | - |
| NZ_CP015001.1_00167 | 399795.CtesDRAFT_PD2880 | 5.6e-205 | 720.7 | Comamonadaceae | L |
| NZ_CP015001.1_00168 | 1196095.GAPWK_1647 | 2.3e-112 | 412.5 | Gammaproteobacteria | U |
| NZ_CP015001.1_00169 | 1437882.AZRU01000002_gene2322 | 8,00E-12 | 76.6 | Pseudomonas aeruginosa group | - |
| NZ_CP015001.1_00170 | 287.DR97_3713 | 6.5e-21 | 106.7 | Pseudomonas aeruginosa group | - |
| NZ_CP015001.1_00171 | 1437882.AZRU01000002_gene2320 | 2.8e-21 | 109.0 | Gammaproteobacteria | - |
| NZ_CP015001.1_00172 | 151599.Q5ZR10_9CAUD | 1,00E-105 | 389.4 | Caudovirales | S |
| NZ_CP015001.1_00175 | 587753.EY04_15085 | 1.2e-44 | 186.0 | Gammaproteobacteria | S |
| NZ_CP015001.1_00176 | 1005395.CSV86_24609 | 3.4e-43 | 181.0 | Bacteria | - |
| NZ_CP015001.1_00177 | 287.DR97_1841 | 5,00E-157 | 560.5 | Gammaproteobacteria | L |
| NZ_CP015377.1_ICE_00005 | 287.DR97_1729 | 4.6e-28 | 130.6 | Pseudomonas aeruginosa group | S |
| NZ_CP015377.1_ICE_00035 | 287.DR97_1757 | 2.8e-294 | 1017.3 | Pseudomonas aeruginosa group | NU |
| NZ_CP015377.1_ICE_00036 | 287.DR97_1758 | 2,00E-247 | 861.3 | Gammaproteobacteria | S |
| NZ_CP015377.1_ICE_00037 | 287.DR97_1759 | 2.3e-82 | 311.6 | Gammaproteobacteria | - |
| NZ_CP015377.1_ICE_00038 | 287.DR97_1760 | 9.7e-63 | 246.1 | Pseudomonas aeruginosa group | NU |
| NZ_CP015377.1_ICE_00039 | 287.DR97_1760 | 7.9e-45 | 186.0 | Pseudomonas aeruginosa group | NU |
| NZ_CP015377.1_ICE_00040 | 287.DR97_1760 | 7.6e-88 | 329.7 | Pseudomonas aeruginosa group | NU |
| NZ_CP015377.1_ICE_00041 | 287.DR97_1760 | 1.1e-36 | 158.7 | Pseudomonas aeruginosa group | NU |
| NZ_CP015377.1_ICE_00043 | 287.DR97_1761 | 1.7e-39 | 168.3 | Pseudomonas aeruginosa group | NU |
| NZ_CP015377.1_ICE_00044 | 287.DR97_1761 | 3,00E-23 | 114.8 | Pseudomonas aeruginosa group | NU |
| NZ_CP015377.1_ICE_00047 | 287.DR97_1763 | 4.2e-30 | 137.1 | Pseudomonas aeruginosa group | NU |
| NZ_CP015377.1_ICE_00048 | 287.DR97_1764 | 2.3e-65 | 255.0 | Pseudomonas aeruginosa group | NU |
| NZ_CP015377.1_ICE_00049 | 287.DR97_1753 | 5.1e-215 | 753.4 | Pseudomonas aeruginosa group | KL |
| NZ_CP015377.1_ICE_00051 | 287.DR97_1754 | 6.1e-45 | 186.4 | Pseudomonas aeruginosa group | K |
| NZ_CP015377.1_ICE_00052 | 287.DR97_1755 | 1.9e-49 | 201.4 | Pseudomonas aeruginosa group | S |
| NZ_CP015377.1_ICE_00053 | 287.DR97_1756 | 1.3e-159 | 569.3 | Pseudomonas aeruginosa group | S |
| NZ_CP015377.1_ICE_00057 | 287.DR97_1760 | 4.5e-109 | 400.6 | Pseudomonas aeruginosa group | NU |
| NZ_CP015377.1_ICE_00059 | 287.DR97_1761 | 5.6e-195 | 686.8 | Pseudomonas aeruginosa group | NU |
| NZ_CP015377.1_ICE_00067 | 287.DR97_5858 | 3.7e-226 | 790.4 | Pseudomonas aeruginosa group | H |
| NZ_CP015377.1_ICE_00068 | 287.DR97_5859 | 2,00E-45 | 188.0 | Pseudomonas aeruginosa group | S |
| NZ_CP015377.1_ICE_00069 | 287.DR97_5860 | 2,00E-143 | 515.0 | Pseudomonas aeruginosa group | H |
| NZ_CP015377.1_ICE_00070 | 287.DR97_5861 | 7.6e-182 | 642.9 | Pseudomonas aeruginosa group | S |
| NZ_CP015377.1_ICE_00071 | 1226994.AMZB01000103_gene1571 | 0.0 | 1126.7 | Pseudomonas aeruginosa group | KQ |
| NZ_CP015377.1_ICE_00072 | 1226994.AMZB01000103_gene1570 | 2.7e-258 | 897.5 | Pseudomonas aeruginosa group | C |
| NZ_CP015377.1_ICE_00073 | 1226994.AMZB01000103_gene1569 | 0.0 | 1434.1 | Pseudomonas aeruginosa group | CG |
| NZ_CP015377.1_ICE_00110 | 390235.PputW619_5175 | 1.7e-51 | 208.4 | Gammaproteobacteria | L |
| NZ_CP015377.1_ICE_00111 | 1388763.O165_024100 | 5.2e-56 | 223.4 | Pseudomonas putida group | L |
| NZ_CP015377.1_ICE_00112 | 1388763.O165_024120 | 8.6e-145 | 520.0 | Pseudomonas putida group | P |
| NZ_CP015377.1_ICE_00113 | 1221522.B723_06990 | 8.9e-60 | 236.1 | Pseudomonas fluorescens group | D |
| NZ_CP015377.1_ICE_00114 | 1221522.B723_06995 | 2,00E-58 | 231.5 | Gammaproteobacteria | S |
| NZ_CP015377.1_ICE_00115 | 118797.XP_007456469.1 | 5,00E-96 | 357.1 | Bilateria | C |
| NZ_CP015377.1_ICE_00116 | 287.DR97_1828 | 5.1e-60 | 236.9 | Pseudomonas aeruginosa group | O |
| NZ_CP015377.1_ICE_00125 | 287.DR97_1835 | 1.1e-32 | 146.0 | Proteobacteria | S |
| NZ_CP015377.1_ICE_00126 | 1331060.RLDS_23330 | 7.5e-40 | 171.4 | Sphingomonadales | - |
| NZ_CP015377.1_ICE_00127 | 549.BW31_01541 | 0.0 | 1513.0 | Gammaproteobacteria | - |
| NZ_CP016955.1_00004 | 287.DR97_1837 | 6,00E-38 | 162.9 | Gammaproteobacteria | - |
| NZ_CP017099.1_00068 | 223283.PSPTO_4328 | 1.1e-72 | 279.3 | Gammaproteobacteria | L |
| NZ_CP017099.1_00069 | 342113.DM82_922 | 3.4e-144 | 518.1 | Burkholderiaceae | E |
| NZ_CP017099.1_00070 | 1207075.PputUW4_02326 | 3.6e-174 | 617.8 | Gammaproteobacteria | EGP |
| NZ_CP017099.1_00071 | 342113.DM82_924 | 4.8e-177 | 628.2 | Burkholderiaceae | G |
| NZ_CP017099.1_00072 | 46234.ANA_C10160 | 2.4e-78 | 299.3 | Nostocales | GM |
| NZ_CP017099.1_00073 | 272630.MexAM1_META1p0381 | 1.4e-07 | 63.5 | Methylobacteriaceae | S |
| NZ_CP017099.1_00074 | 319003.Bra1253DRAFT_07925 | 4.2e-30 | 137.9 | Bradyrhizobiaceae | IM |
| NZ_CP017099.1_00076 | 1449342.JQMR01000001_gene585 | 2.9e-81 | 309.3 | Bacilli | H |
| NZ_CP017099.1_00077 | 1042876.PPS_3160 | 1.6e-91 | 342.0 | Pseudomonas putida group | L |
| NZ_CP017353.1_00066 | 440512.C211_02431 | 5.8e-28 | 129.4 | Bacteria | L |
| NZ_CP017353.1_00067 | 440512.C211_02431 | 1.6e-17 | 95.1 | Bacteria | L |
| NZ_CP017969.1_00005 | 287.DR97_1727 | 5.3e-95 | 354.0 | Gammaproteobacteria | - |
| NZ_CP017969.1_00030 | 1294143.H681_06280 | 7.9e-95 | 353.2 | Gammaproteobacteria | E |
| NZ_CP017969.1_00031 | 1294143.H681_06285 | 1.5e-147 | 528.9 | Gammaproteobacteria | K |
| NZ_CP017969.1_00044 | 629265.PMA4326_08665 | 6.2e-98 | 364.0 | Gammaproteobacteria | K |
| NZ_CP017969.1_00045 | 571.MC52_00640 | 6.6e-146 | 523.9 | Gammaproteobacteria | S |
| NZ_CP017969.1_00046 | 571.MC52_00635 | 1.1e-213 | 750.0 | Gammaproteobacteria | H |
| NZ_CP017969.1_00047 | 266265.Bxe_A0217 | 1.4e-94 | 352.8 | Burkholderiaceae | S |
| NZ_CP017969.1_00048 | 266265.Bxe_A0218 | 1.1e-57 | 230.3 | Burkholderiaceae | - |
| NZ_CP017969.1_00065 | 287.DR97_1407 | 1.9e-14 | 84.7 | Pseudomonas aeruginosa group | E |
| NZ_CP017969.1_00066 | 1121127.JAFA01000014_gene6546 | 1.1e-101 | 377.1 | Burkholderiaceae | KT |
| NZ_CP017969.1_00067 | 1215114.BBIU01000004_gene436 | 5.7e-13 | 80.1 | Gammaproteobacteria | S |
| NZ_CP017969.1_00068 | 1215114.BBIU01000004_gene435 | 2.6e-128 | 465.3 | Gammaproteobacteria | V |
| NZ_CP017969.1_00069 | 1215114.BBIU01000004_gene434 | 5.7e-161 | 574.7 | Gammaproteobacteria | S |
| NZ_CP017969.1_00071 | 287.DR97_5436 | 5.6e-93 | 347.1 | Pseudomonas aeruginosa group | K |
| NZ_CP017969.1_00072 | 287.DR97_5435 | 3.5e-63 | 247.7 | Gammaproteobacteria | C |
| NZ_CP017969.1_00073 | 359.CN09_31800 | 9,00E-79 | 300.4 | Rhizobiaceae | K |
| NZ_CP017969.1_00074 | 1419583.V466_11030 | 1.5e-92 | 346.7 | Pseudomonas fluorescens group | EGP |
| NZ_CP017969.1_00075 | 598467.BrE312_0537 | 1.9e-124 | 452.6 | Gammaproteobacteria | V |
| NZ_CP017969.1_00076 | 598467.BrE312_0538 | 7.2e-118 | 431.0 | Gammaproteobacteria | MU |
| NZ_CP017969.1_00077 | 311403.Arad_7251 | 3.1e-80 | 304.7 | Alphaproteobacteria | Q |
| NZ_CP017969.1_00078 | 1366050.N234_12125 | 1,00E-80 | 306.6 | Burkholderiaceae | S |
| NZ_CP017969.1_00079 | 1123504.JQKD01000108_gene5212 | 3.8e-68 | 264.6 | Comamonadaceae | O |
| NZ_CP017969.1_00080 | 311403.Arad_7247 | 3.7e-114 | 417.9 | Rhizobiaceae | S |
| NZ_CP017969.1_00082 | 32042.PstZobell_10539 | 4.5e-76 | 291.6 | Pseudomonas stutzeri group | L |
| NZ_CP017969.1_00107 | 1042876.PPS_5229 | 2.3e-32 | 144.4 | Pseudomonas putida group | L |
| NZ_CP017969.1_00112 | 1452718.JBOY01000065_gene2449 | 0.0 | 1901.7 | Gammaproteobacteria | L |
| NZ_CP017969.1_00118 | 287.DR97_1833 | 4.2e-273 | 946.8 | Gammaproteobacteria | S |
| NZ_CP017969.1_00122 | 658612.MD26_13360 | 9.8e-140 | 503.1 | Gammaproteobacteria | S |
| NZ_CP017969.1_00124 | 1388763.O165_023335 | 5.4e-100 | 370.5 | Gammaproteobacteria | L |
| NZ_CP017969.1_00125 | 1217703.F904_00999 | 2.3e-92 | 345.5 | Gammaproteobacteria | L |
| NZ_CP017969.1_00126 | 857087.Metme_4483 | 2,00E-49 | 202.6 | Proteobacteria | C |
| NZ_CP017969.1_00127 | 1217703.F904_00997 | 3.7e-76 | 291.2 | Moraxellaceae | F |
| NZ_CP017969.1_00128 | 857087.Metme_4485 | 3.5e-126 | 458.4 | Gammaproteobacteria | G |
| NZ_CP017969.1_00129 | 1217703.F904_00995 | 3.4e-12 | 77.8 | Moraxellaceae | - |
| NZ_CP020704.1_00037 | 930169.B5T_02144 | 1.4e-36 | 160.2 | Gammaproteobacteria | - |
| NZ_CP020704.1_00066 | 287.DR97_5915 | 2.1e-23 | 114.8 | Pseudomonas aeruginosa group | NT |
| NZ_CP020704.1_00067 | 399739.Pmen_0427 | 5.5e-183 | 646.7 | Pseudomonas aeruginosa group | L |
| NZ_CP020704.1_00068 | 1323663.AROI01000029_gene1962 | 1.5e-174 | 619.4 | Gammaproteobacteria | J |
| NZ_CP020704.1_00069 | 1282356.H045_09500 | 1.3e-25 | 122.5 | Pseudomonas fluorescens group | S |
| NZ_CP020704.1_00070 | 1207076.ALAT01000198_gene1167 | 9.1e-154 | 550.1 | Pseudomonas stutzeri group | I |
| NZ_CP020704.1_00071 | 1395571.TMS3_0121435 | 1.5e-80 | 305.8 | Gammaproteobacteria | K |
| NZ_CP020704.1_00072 | 1453503.AU05_17105 | 2.4e-15 | 87.8 | Pseudomonas aeruginosa group | T |
| NZ_CP020704.1_00073 | 1136138.JH604622_gene416 | 3.3e-40 | 170.6 | Gammaproteobacteria | S |
| NZ_CP020704.1_00074 | 1136138.JH604622_gene417 | 3.6e-39 | 167.2 | Gammaproteobacteria | K |
| NZ_CP020704.1_00075 | 1245471.PCA10_p0510 | 3.3e-87 | 328.2 | Pseudomonas aeruginosa group | S |
| NZ_CP020704.1_00076 | 1211112.ALJC01000134_gene3399 | 1.6e-103 | 382.1 | Gammaproteobacteria | L |
| NZ_CP020704.1_00077 | 1215092.PA6_067_00030 | 0.0 | 1962.2 | Pseudomonas aeruginosa group | L |
| NZ_CP020704.1_00112 | 287.DR97_1841 | 8.5e-147 | 526.9 | Gammaproteobacteria | L |
| NZ_CP021999.1_00090 | 1415630.U771_04520 | 3.6e-33 | 147.9 | Gammaproteobacteria | S |
| NZ_CP022002.1_00107 | 287.DR97_1741 | 3.5e-86 | 324.3 | Gammaproteobacteria | K |
| NZ_CP022478.1_00095 | 1005395.CSV86_18402 | 6,00E-10 | 70.5 | Pseudomonas putida group | - |
| NZ_CP022478.1_00096 | 287.DR97_1833 | 1.1e-262 | 912.1 | Gammaproteobacteria | S |
| NZ_CP024477.1_00038 | 287.DR97_5228 | 3.2e-99 | 367.9 | Pseudomonas aeruginosa group | L |
| NZ_CP024477.1_00039 | 1007105.PT7_2838 | 2.9e-241 | 840.9 | Alcaligenaceae | L |
| NZ_CP024630.1_00062 | 1452718.JBOY01000089_gene1007 | 1.5e-245 | 855.1 | Gammaproteobacteria | M |
| NZ_CP024630.1_00063 | 1452718.JBOY01000089_gene1006 | 0.0 | 1935.6 | Gammaproteobacteria | V |
| NZ_CP024630.1_00064 | 1452718.JBOY01000089_gene1005 | 9.6e-158 | 563.1 | Gammaproteobacteria | M |
| NZ_CP024630.1_00065 | 1441629.PCH70_21620 | 1,00E-72 | 279.6 | Pseudomonas syringae group | K |
| NZ_CP024630.1_00066 | 1123020.AUIE01000010_gene1237 | 8.5e-298 | 1029.6 | Gammaproteobacteria | L |
| NZ_CP024630.1_00067 | 1207076.ALAT01000007_gene1388 | 6,00E-50 | 203.8 | Proteobacteria | - |
| NZ_CP024630.1_00068 | 1123053.AUDG01000066_gene2610 | 1.3e-290 | 1005.4 | Proteobacteria | L |
| NZ_CP024630.1_00069 | 1123053.AUDG01000066_gene2609 | 8.8e-256 | 889.4 | Gammaproteobacteria | L |
| NZ_CP024630.1_00070 | 1144319.PMI16_04806 | 9.3e-202 | 709.5 | Oxalobacteraceae | L |
| NZ_CP024630.1_00071 | 1218352.B597_022960 | 6.3e-45 | 186.4 | Pseudomonas stutzeri group | L |
| NZ_CP024630.1_00072 | 1212548.B381_05156 | 5,00E-122 | 443.7 | Pseudomonas stutzeri group | L |
| NZ_CP024630.1_00073 | 1117958.PE143B_0100110 | 0.0 | 2359.3 | Gammaproteobacteria | L |
| NZ_CP024630.1_00074 | 1117958.PE143B_0100115 | 3.8e-164 | 584.7 | Gammaproteobacteria | - |
| NZ_CP024630.1_00077 | 1484157.PSNIH2_21645 | 1.7e-195 | 688.3 | Pantoea | L |
| NZ_CP024630.1_00078 | 1173020.Cha6605_6175 | 1.1e-93 | 349.4 | Cyanobacteria | J |
| NZ_CP024630.1_00079 | 1211112.ALJC01000116_gene2892 | 7.2e-77 | 293.1 | Proteobacteria | U |
| NZ_CP024630.1_00080 | 1211112.ALJC01000116_gene2891 | 1.5e-201 | 708.8 | Gammaproteobacteria | EGP |
| NZ_CP024630.1_00081 | 1211112.ALJC01000116_gene2890 | 1.3e-113 | 415.6 | Gammaproteobacteria | K |
| NZ_CP024630.1_00082 | 1211112.ALJC01000116_gene2889 | 2.7e-208 | 731.1 | Gammaproteobacteria | EGP |
| NZ_CP024630.1_00083 | 90371.CY43_20095 | 1.6e-47 | 195.7 | Proteobacteria | - |
| NZ_CP024630.1_00084 | 1211112.ALJC01000116_gene2887 | 3.1e-297 | 1026.9 | Gammaproteobacteria | M |
| NZ_CP024630.1_00085 | 90371.CY43_20105 | 5.2e-108 | 397.1 | Salmonella | O |
| NZ_CP024630.1_00086 | 1484157.PSNIH2_20735 | 1.1e-155 | 555.8 | Gammaproteobacteria | H |
| NZ_CP024630.1_00087 | 1176165.CAJD01000003_gene33 | 3.3e-94 | 350.9 | Bacteria | K |
| NZ_CP024630.1_00088 | 1097668.BYI23_E002520 | 1.7e-150 | 538.5 | Burkholderiaceae | L |
| NZ_CP025229.1_00002 | 287.DR97_1840 | 1.6e-61 | 241.9 | Gammaproteobacteria | S |
| NZ_CP025229.1_00065 | 287.DR97_1764 | 1.5e-150 | 538.9 | Pseudomonas aeruginosa group | NU |
| NZ_CP026680.1_00029 | 390235.PputW619_3356 | 1,00E-41 | 176.4 | Proteobacteria | - |
| NZ_CP026680.1_00083 | 670307.HYPDE_40248 | 3.6e-108 | 398.7 | Alphaproteobacteria | - |
| NZ_CP026680.1_00084 | 1294143.H681_04385 | 3,00E-168 | 598.2 | Gammaproteobacteria | S |
| NZ_CP026680.1_00085 | 1149133.ppKF707_0545 | 3.3e-37 | 160.6 | Pseudomonas aeruginosa group | L |
| NZ_CP026680.1_00086 | 1196835.A458_08070 | 3.5e-17 | 93.2 | Pseudomonas stutzeri group | L |
| NZ_CP026680.1_00091 | 287.DR97_1831 | 6.1e-86 | 323.6 | Pseudomonas aeruginosa group | S |
| NZ_CP026680.1_00097 | 1005395.CSV86_09088 | 1.3e-12 | 81.3 | Gammaproteobacteria | S |
| NZ_CP027165.1_00065 | 1112217.PPL19_20961 | 5.3e-13 | 80.1 | Gammaproteobacteria | L |
| NZ_CP027165.1_00112 | 1452718.JBOY01000120_gene2768 | 1.1e-22 | 112.8 | Gammaproteobacteria | - |
| NZ_CP028848.1_00090 | 1163398.AJJP01000227_gene2388 | 1.3e-38 | 165.6 | Gammaproteobacteria | L |
| NZ_CP028848.1_00091 | 342113.DM82_4483 | 1.5e-70 | 272.7 | Burkholderiaceae | L |
| NZ_CP028848.1_00092 | 1163398.AJJP01000227_gene2388 | 6,00E-18 | 96.3 | Gammaproteobacteria | L |
| NZ_CP028848.1_00093 | 1001585.MDS_0249 | 2.7e-19 | 102.4 | Pseudomonas aeruginosa group | T |
| NZ_CP028848.1_00094 | 999541.bgla_2g16460 | 5.5e-56 | 224.2 | Burkholderiaceae | L |
| NZ_CP028848.1_00095 | 287.DR97_1957 | 6.4e-67 | 260.4 | Pseudomonas aeruginosa group | S |
| NZ_CP028848.1_00096 | 287.DR97_1958 | 1.4e-71 | 275.8 | Pseudomonas aeruginosa group | S |
| NZ_CP028848.1_00097 | 287.DR97_1959 | 5.4e-74 | 283.9 | Pseudomonas aeruginosa group | S |
| NZ_CP028848.1_00098 | 287.DR97_1960 | 1.6e-140 | 505.4 | Pseudomonas aeruginosa group | M |
| NZ_CP028848.1_00099 | 287.DR97_1961 | 0.0 | 1530.0 | Pseudomonas aeruginosa group | NU |
| NZ_CP028848.1_00100 | 287.DR97_1962 | 7.7e-137 | 493.4 | Gammaproteobacteria | S |
| NZ_CP028848.1_00101 | 223283.PSPTO_5629 | 5.6e-102 | 377.9 | Gammaproteobacteria | L |
| NZ_CP028848.1_00103 | 1214065.BAGV01000015_gene2365 | 1.3e-49 | 202.2 | Gammaproteobacteria | S |
| NZ_CP028848.1_00108 | 287.DR97_1833 | 8.4e-274 | 949.1 | Gammaproteobacteria | S |
| NZ_CP028917.1_00105 | 76869.PputGB1_4823 | 1.1e-181 | 642.5 | Gammaproteobacteria | L |
| NZ_CP029605.1_00089 | 283942.IL1808 | 2.3e-102 | 379.4 | Gammaproteobacteria | V |
| NZ_CP029605.1_00090 | 1042209.HK44_023770 | 1.2e-104 | 386.0 | Gammaproteobacteria | L |
| NZ_CP029605.1_00091 | 1007105.PT7_0588 | 2.3e-225 | 787.7 | Betaproteobacteria | S |
| NZ_CP029605.1_00092 | 266265.Bxe_C1174 | 1.7e-165 | 588.6 | Betaproteobacteria | O |
| NZ_CP029605.1_00093 | 1134474.O59_002514 | 0.0 | 1087.0 | Cellvibrio | L |
| NZ_CP029707.1_00102 | 1097668.BYI23_E000580 | 1.6e-46 | 193.4 | Betaproteobacteria | S |
| NZ_CP030328.1_00081 | 1097668.BYI23_E002870 | 4.1e-69 | 267.3 | Burkholderiaceae | K |
| NZ_CP030328.1_00082 | 375286.mma_1752 | 8.5e-57 | 226.1 | Oxalobacteraceae | P |
| NZ_CP030328.1_00084 | 571.MC52_30165 | 6.3e-63 | 246.5 | Gammaproteobacteria | S |
| NZ_CP030328.1_00085 | 571.MC52_30160 | 1.6e-289 | 1001.5 | Gammaproteobacteria | C |
| NZ_CP030328.1_00086 | 1218076.BAYB01000072_gene6430 | 1.8e-57 | 228.4 | Burkholderiaceae | K |
| NZ_CP030328.1_00087 | 1218076.BAYB01000072_gene6429 | 8.5e-37 | 159.1 | Burkholderiaceae | S |
| NZ_CP030328.1_00088 | 1218076.BAYB01000072_gene6428 | 1.5e-178 | 632.1 | Burkholderiaceae | T |
| NZ_CP030328.1_00090 | 237609.PSAKL28_31230 | 2.6e-238 | 830.9 | Gammaproteobacteria | S |
| NZ_CP030328.1_00092 | 1097668.BYI23_E002790 | 0.0 | 1102.4 | Burkholderiaceae | L |
| NZ_CP030910.1_00019 | 1443113.LC20_03104 | 1.2e-68 | 267.3 | Gammaproteobacteria | K |
| NZ_CP030910.1_00020 | 1443113.LC20_03105 | 2.2e-75 | 288.9 | Gammaproteobacteria | L |
| NZ_CP030911.1_00095 | 287.DR97_1836 | 2.3e-162 | 578.2 | Gammaproteobacteria | - |
| NZ_CP030911.1_00097 | 1333856.L686_06390 | 6.4e-54 | 216.5 | Pseudomonas stutzeri group | L |
| NZ_CP031449.2_00028 | 390235.PputW619_3356 | 3.9e-41 | 174.5 | Proteobacteria | - |
| NZ_CP031659.1_00055 | 754035.Mesau_00730 | 7,00E-25 | 119.8 | Alphaproteobacteria | - |
| NZ_CP031659.1_00056 | 469595.CSAG_02793 | 1.6e-123 | 449.1 | Citrobacter | K |
| NZ_CP031659.1_00067 | 1437882.AZRU01000211_gene2478 | 1.4e-65 | 256.1 | Pseudomonas aeruginosa group | T |
| NZ_CP031659.1_00068 | 1437882.AZRU01000211_gene2477 | 1.8e-278 | 965.7 | Gammaproteobacteria | T |
| NZ_CP031659.1_00069 | 1294143.H681_05960 | 1.1e-100 | 373.6 | Gammaproteobacteria | T |
| NZ_CP031659.1_00070 | 1294143.H681_05955 | 3.2e-142 | 512.7 | Gammaproteobacteria | T |
| NZ_CP031659.1_00071 | 243365.CV_1294 | 6.9e-21 | 107.5 | Neisseriales | NU |
| NZ_CP031659.1_00072 | 1294143.H681_03335 | 8.7e-56 | 223.8 | Gammaproteobacteria | M |
| NZ_CP031659.1_00073 | 1294143.H681_03340 | 2.2e-239 | 835.5 | Gammaproteobacteria | NU |
| NZ_CP031659.1_00074 | 1042209.HK44_023730 | 3.5e-20 | 105.1 | Bacteria | NU |
| NZ_CP032126.1_00066 | 379731.PST_3352 | 0.0 | 1308.1 | Gammaproteobacteria | L |
| NZ_CP032126.1_00067 | 379731.PST_3353 | 7.7e-175 | 619.8 | Gammaproteobacteria | L |
| NZ_CP032126.1_00080 | 287.DR97_1780 | 2.7e-38 | 164.1 | Bacteria | L |
| NZ_CP032552.1_00004 | 287.DR97_1726 | 1.1e-73 | 283.5 | Pseudomonas aeruginosa group | - |
| NZ_CP032569.1_00082 | 1415630.U771_31755 | 7.8e-86 | 323.2 | Gammaproteobacteria | L |
| NZ_CP032569.1_00083 | 1117958.PE143B_0100860 | 3.9e-253 | 880.2 | Gammaproteobacteria | L |
| NZ_CP032569.1_00084 | 1196835.A458_08060 | 2.4e-95 | 354.8 | Pseudomonas stutzeri group | L |
| NZ_CP032569.1_00110 | 1123020.AUIE01000007_gene3278 | 6.9e-272 | 942.6 | Pseudomonas aeruginosa group | L |
| NZ_CP033771.1_00052 | 1323663.AROI01000055_gene1436 | 1.1e-157 | 562.8 | Gammaproteobacteria | L |
| NZ_CP033771.1_00075 | 1323663.AROI01000055_gene1436 | 1.1e-157 | 562.8 | Gammaproteobacteria | L |
| NZ_CP033771.1_00077 | 1395516.PMO01_23420 | 1.6e-126 | 458.8 | Bacteria | T |
| NZ_CP033771.1_00079 | 1395516.PMO01_23430 | 1.4e-239 | 835.1 | Gammaproteobacteria | I |
| NZ_CP033771.1_00080 | 1395516.PMO01_23435 | 1.8e-119 | 435.3 | Gammaproteobacteria | IQ |
| NZ_CP033771.1_00081 | 1395516.PMO01_23445 | 6.4e-187 | 659.8 | Gammaproteobacteria | - |
| NZ_CP033771.1_00082 | 1323663.AROI01000055_gene1436 | 1.1e-157 | 562.8 | Gammaproteobacteria | L |
| NZ_CP033771.1_00083 | 1395516.PMO01_23450 | 3.8e-100 | 370.9 | Gammaproteobacteria | M |
| NZ_CP033771.1_00084 | 1323663.AROI01000055_gene1436 | 1.1e-157 | 562.8 | Gammaproteobacteria | L |
| NZ_CP033771.1_00099 | 1323663.AROI01000055_gene1436 | 1.1e-157 | 562.8 | Gammaproteobacteria | L |
| NZ_CP033771.1_00111 | 1323663.AROI01000055_gene1436 | 1.1e-157 | 562.8 | Gammaproteobacteria | L |
| NZ_CP033835.1_2Mb_00006 | 287.DR97_1833 | 2.7e-272 | 944.1 | Gammaproteobacteria | S |
| NZ_CP033835.1_2Mb_00101 | 287.DR97_1724 | 1.5e-119 | 435.6 | Pseudomonas aeruginosa group | - |
| NZ_CP034354.1_00059 | 1357275.AVEL02000134_gene276 | 6.1e-14 | 82.4 | Gammaproteobacteria | L |
| NZ_CP034354.1_00096 | 1288963.ADIS_4035 | 3.3e-155 | 555.1 | Bacteroidetes | L |
| NZ_CP034354.1_00097 | 177437.HRM2_p00700 | 1.8e-37 | 162.2 | Deltaproteobacteria | - |
| NZ_CP034354.1_00098 | 1207076.ALAT01000172_gene2246 | 2.1e-108 | 398.7 | Bacteria | - |
| NZ_CP034354.1_00099 | 257310.BB0484 | 3.7e-74 | 284.3 | Betaproteobacteria | S |
| NZ_CP034354.1_00100 | 317025.Tcr_0374 | 1.2e-47 | 196.1 | Gammaproteobacteria | S |
| NZ_CP034354.1_00101 | 257310.BB0482 | 1.7e-54 | 219.2 | Betaproteobacteria | S |
| NZ_CP034354.1_00102 | 1454004.AW11_02265 | 5.2e-103 | 381.3 | Betaproteobacteria | S |
| NZ_CP034354.1_00103 | 1454004.AW11_02266 | 2.5e-53 | 215.3 | Betaproteobacteria | - |
| NZ_CP035739.1_ICE_00006 | 95619.PM1_0220260 | 2.3e-72 | 278.5 | Bacteria | K |
| NZ_CP035739.1_ICE_00007 | 1226994.AMZB01000077_gene512 | 9,00E-165 | 586.3 | Bacteria | PT |
| NZ_CP035739.1_ICE_00008 | 1211112.ALJC01000087_gene925 | 0.0 | 1182.2 | Gammaproteobacteria | P |
| NZ_CP035739.1_ICE_00009 | 1218352.B597_006740 | 9.8e-258 | 895.6 | Pseudomonas stutzeri group | L |
| NZ_CP039988.1_00102 | 287.DR97_1835 | 7.4e-53 | 213.0 | Proteobacteria | S |
| NZ_CP039988.1_00103 | 32057.KB217472_gene7933 | 1.9e-104 | 386.7 | Nostocales | S |
| NZ_CP039988.1_00104 | 231434.JQJH01000005_gene2294 | 1.5e-55 | 223.4 | Proteobacteria | - |
| NZ_CP039988.1_00105 | 1419583.V466_23080 | 2.1e-132 | 478.8 | Proteobacteria | S |
| NZ_CP039990.1_00090 | 287.DR97_1835 | 1.2e-53 | 215.7 | Proteobacteria | S |
| NZ_CP039990.1_00091 | 1215092.PA6_009_00430 | 1.3e-27 | 129.0 | Pseudomonas aeruginosa group | L |
| NZ_CP039990.1_00092 | 1215092.PA6_009_00430 | 3.4e-08 | 63.5 | Pseudomonas aeruginosa group | L |
| NZ_CP040684.1_00001 | 469595.CSAG_04354 | 3.6e-185 | 654.1 | Citrobacter | L |
| NZ_CP041013.1_00062 | 287.DR97_1783 | 2.3e-102 | 378.3 | Gammaproteobacteria | - |
| NZ_CP041354.1_00014 | 1218352.B597_008095 | 6.9e-289 | 999.2 | Pseudomonas stutzeri group | L |
| NZ_CP041354.1_00015 | 1182590.BN5_04361 | 7.3e-138 | 496.5 | Pseudomonas aeruginosa group | L |
| NZ_CP041354.1_00071 | 1182590.BN5_04361 | 7.3e-138 | 496.5 | Pseudomonas aeruginosa group | L |
| NZ_CP041354.1_00072 | 1218352.B597_008095 | 6.9e-289 | 999.2 | Pseudomonas stutzeri group | L |
| NZ_CP041771.1_00031 | 287.DR97_784 | 2.5e-49 | 204.1 | Pseudomonas aeruginosa group | S |
| NZ_CP041774.1_00103 | 380358.XALC_1244 | 1.2e-62 | 245.7 | Proteobacteria | L |
| NZ_CP041774.1_00104 | 596154.Alide2_3660 | 8.3e-34 | 150.2 | Comamonadaceae | - |
| NZ_CP041774.1_00105 | 670292.JH26_07345 | 6.3e-47 | 194.5 | Bacteria | I |
| NZ_CP041774.1_00106 | 477184.KYC_14907 | 3.2e-139 | 501.1 | Betaproteobacteria | L |
| NZ_CP041774.1_00107 | 477184.KYC_14912 | 1.4e-35 | 155.2 | Alcaligenaceae | L |
| NZ_CP041774.1_00109 | 511.JT27_13725 | 2.1e-157 | 561.6 | Betaproteobacteria | Q |
| NZ_CP041774.1_00110 | 511.JT27_13720 | 7.8e-180 | 636.3 | Alcaligenaceae | K |
| NZ_CP041774.1_00112 | 391008.Smal_3775 | 1.3e-14 | 85.5 | Xanthomonadales | S |
| NZ_CP043328.1_00130 | 522306.CAP2UW1_0649 | 2.2e-160 | 571.6 | Betaproteobacteria | V |
| NZ_CP044006.1_00076 | 640081.Dsui_1508 | 3.4e-132 | 477.6 | Rhodocyclales | L |
| NZ_CP044006.1_00077 | 640081.Dsui_1507 | 6.4e-287 | 992.6 | Rhodocyclales | L |
| NZ_CP046060.1_00005 | 287.DR97_1727 | 2.3e-130 | 471.5 | Gammaproteobacteria | - |
| NZ_CP046060.1_00010 | 1388763.O165_009820 | 2.4e-200 | 704.5 | Gammaproteobacteria | L |
| NZ_CP046060.1_00011 | 1388763.O165_009815 | 3.8e-73 | 280.8 | Gammaproteobacteria | L |
| NZ_CP046060.1_00029 | 1388763.O165_009820 | 2.4e-200 | 704.5 | Gammaproteobacteria | L |
| NZ_CP046060.1_00030 | 1388763.O165_009815 | 3.8e-73 | 280.8 | Gammaproteobacteria | L |
| NZ_CP046060.1_00098 | 1388763.O165_009820 | 2.4e-200 | 704.5 | Gammaproteobacteria | L |
| NZ_CP046060.1_00099 | 1388763.O165_009815 | 3.3e-73 | 281.2 | Gammaproteobacteria | L |
| NZ_CP047592.1_00105 | 68170.KL590521_gene9175 | 9.3e-72 | 276.9 | Actinobacteria | S |
| NZ_LR130527.1_00004 | 287.DR97_1726 | 4.4e-75 | 288.5 | Pseudomonas aeruginosa group | - |
| NZ_LR130527.1_00118 | 1500306.JQLA01000058_gene176 | 2.2e-51 | 209.9 | Rhizobiaceae | F |
| NZ_LR130527.1_00119 | 1268239.PALB_17210 | 2.6e-24 | 118.6 | Gammaproteobacteria | KT |
| NZ_LR130527.1_00120 | 1500306.JQLA01000058_gene174 | 5.8e-39 | 169.9 | Rhizobiaceae | T |
| NZ_LR134300.1_00039 | 1144307.PMI04_00913 | 6.2e-150 | 537.3 | Sphingomonadales | - |
| NZ_LR134300.1_00040 | 1149133.ppKF707_1404 | 6.3e-156 | 557.4 | Pseudomonas aeruginosa group | L |
| NZ_LR134300.1_00068 | 1424334.W822_09445 | 4.3e-232 | 810.4 | Alcaligenaceae | F |
| NZ_LR134300.1_00069 | 1424334.W822_09450 | 8.2e-286 | 989.2 | Alcaligenaceae | L |
| NZ_LR134300.1_00070 | 1215092.PA6_056_00150 | 6.6e-265 | 919.5 | Pseudomonas aeruginosa group | L |
| NZ_LR134300.1_00071 | 1301098.PKB_4380 | 5.6e-104 | 383.6 | Gammaproteobacteria | L |
| NZ_LR134300.1_00072 | 1226994.AMZB01000066_gene1887 | 2.5e-163 | 581.3 | Pseudomonas aeruginosa group | S |
| NZ_LR134300.1_00073 | 1437882.AZRU01000058_gene4176 | 8.6e-223 | 779.2 | Pseudomonas aeruginosa group | I |
| NZ_LR134300.1_00074 | 1226994.AMZB01000067_gene1890 | 9.6e-136 | 489.6 | Pseudomonas aeruginosa group | G |
| NZ_LR134300.1_00075 | 1226994.AMZB01000067_gene1891 | 8.3e-240 | 835.9 | Pseudomonas aeruginosa group | S |
| NZ_LR134300.1_00076 | 1226994.AMZB01000067_gene1892 | 1.7e-221 | 775.0 | Pseudomonas aeruginosa group | I |
| NZ_LR134300.1_00077 | 1226994.AMZB01000067_gene1893 | 3,00E-159 | 567.8 | Proteobacteria | I |
| NZ_LR134300.1_00078 | 1226994.AMZB01000067_gene1894 | 3.7e-181 | 640.6 | Gammaproteobacteria | S |
| NZ_LR134300.1_00079 | 1226994.AMZB01000067_gene1895 | 3.2e-164 | 584.3 | Pseudomonas aeruginosa group | K |
| NZ_LR134300.1_00080 | 1226994.AMZB01000067_gene1896 | 1.5e-210 | 738.4 | Gammaproteobacteria | S |
| NZ_LR134300.1_00081 | 1226994.AMZB01000067_gene1897 | 3.1e-127 | 461.1 | Gammaproteobacteria | IQ |
| NZ_LR134300.1_00082 | 1226994.AMZB01000067_gene1898 | 8.1e-128 | 463.0 | Pseudomonas aeruginosa group | I |
| NZ_LR134300.1_00083 | 1226994.AMZB01000067_gene1899 | 1,00E-130 | 472.6 | Pseudomonas aeruginosa group | I |
| NZ_LR134300.1_00084 | 1226994.AMZB01000067_gene1900 | 4,00E-172 | 610.5 | Gammaproteobacteria | I |
| NZ_LR134300.1_00085 | 1226994.AMZB01000067_gene1901 | 2.5e-239 | 834.3 | Pseudomonas aeruginosa group | EGP |
| NZ_LR134300.1_00086 | 1301098.PKB_4396 | 7.7e-45 | 186.0 | Gammaproteobacteria | Q |
| NZ_LR134300.1_00087 | 1226994.AMZB01000067_gene1903 | 1.5e-228 | 798.5 | Proteobacteria | C |
| NZ_LR134300.1_00088 | 477228.YO5_14495 | 1.4e-267 | 928.3 | Pseudomonas stutzeri group | L |
| NZ_LR134300.1_00089 | 666684.AfiDRAFT_1370 | 6.4e-59 | 234.2 | Alphaproteobacteria | - |
| NZ_LR134300.1_00092 | 379731.PST_3352 | 0.0 | 1348.2 | Gammaproteobacteria | L |
| NZ_LR134300.1_00093 | 379731.PST_3353 | 4.2e-181 | 640.6 | Gammaproteobacteria | L |
| NZ_LR134300.1_00136 | 1301098.PKB_5472 | 1.1e-306 | 1058.9 | Gammaproteobacteria | G |
| NZ_LR134308.1_00079 | 287.DR97_1763 | 1.7e-24 | 117.9 | Pseudomonas aeruginosa group | NU |
| NZ_LR134308.1_00081 | 287.DR97_1763 | 2.3e-60 | 238.4 | Pseudomonas aeruginosa group | NU |
| NZ_LR590473.1_00079 | 287.DR97_1793 | 1.1e-29 | 135.2 | Gammaproteobacteria | U |
| NZ_LT883143.1_00041 | 1211112.ALJC01000059_gene1643 | 5,00E-101 | 373.6 | Bacteria | S |
| NZ_LT883143.1_00043 | 1121116.KB894794_gene1267 | 3.2e-95 | 354.4 | Comamonadaceae | - |
| NZ_LT883143.1_00044 | 1121116.KB894794_gene1266 | 2.6e-62 | 244.6 | Comamonadaceae | S |
| NZ_LT883143.1_00045 | 1094184.KWO_0105095 | 4.8e-38 | 163.3 | Proteobacteria | K |
| NZ_LT883143.1_00046 | 1121116.KB894794_gene1265 | 1.8e-96 | 358.6 | Comamonadaceae | L |
| NZ_LT883143.1_00047 | 1458427.BAWN01000094_gene2220 | 7.3e-86 | 323.9 | Comamonadaceae | L |
| NZ_LT883143.1_00048 | 325777.GW15_0222530 | 2.4e-102 | 378.3 | Xanthomonadales | D |
| NZ_LT883143.1_00049 | 325777.GW15_0222525 | 5.2e-20 | 103.2 | Gammaproteobacteria | - |
| NZ_LT883143.1_00050 | 325777.GW15_0222520 | 1.6e-143 | 515.8 | Gammaproteobacteria | S |
| NZ_LT883143.1_00051 | 85643.Tmz1t_2361 | 1.1e-28 | 134.0 | Betaproteobacteria | S |
| NZ_LT883143.1_00053 | 1131451.O1K_04406 | 1.2e-187 | 663.7 | Xanthomonadales | U |
| NZ_LT883143.1_00054 | 1500257.JQNM01000007_gene1692 | 2,00E-07 | 61.6 | Rhizobiaceae | S |
| NZ_LT883143.1_00055 | 202952.BBLI01000084_gene3695 | 3.8e-263 | 914.8 | Gammaproteobacteria | L |
| NZ_LT883143.1_00058 | 243233.MCA1619 | 1.4e-34 | 153.7 | Bacteria | O |
| NZ_LT883143.1_00060 | 232721.Ajs_4254 | 3.8e-25 | 120.2 | Proteobacteria | - |
| NZ_LT883143.1_00062 | 232721.Ajs_4253 | 3.5e-115 | 421.0 | Proteobacteria | L |
| NZ_LT883143.1_00063 | 1097668.BYI23_E002520 | 1.7e-150 | 538.5 | Burkholderiaceae | L |
| NZ_LT969520.1_ICE_2_00053 | 1117958.PE143B_0128655 | 6.4e-88 | 330.1 | Gammaproteobacteria | L |
| NZ_LT969520.1_ICE_2_00054 | 1117958.PE143B_0128650 | 1.5e-152 | 545.4 | Gammaproteobacteria | L |
