# Supplementary material for: ICEs Are the Main Reservoirs of the Ciprofloxacin-Modifying crpP Gene in Pseudomonas aeruginosa
Source: Genes (Basel). 2020 Aug 4;11(8):889. doi: 10.3390/genes11080889 (PMC7463715; doi:10.3390/genes11080889)
Supplement: Supplementary file 1 [file genes-11-00889-s001.zip › Table_S5.docx]

**Table S5**. Macsyfinder reports for the ICE proteomes analysed in this study.

| **Hit_Id** | **Sequence length** | **Gene** | **Predicted system** | **Gene status** | **i-evalue** | **Score** | **Profile coverage** | **Sequence coverage** |
| --- | --- | --- | --- | --- | --- | --- | --- | --- |
| NC_008463.1_5251438..5359437_00068 | 251 | G_tfc3 | typeG | accessory | 4.700e-95 | 310.000 | 0.967 | 0.976 |
| NC_008463.1_5251438..5359437_00070 | 166 | G_tfc5 | typeG | accessory | 7.000e-56 | 181.500 | 0.977 | 1.000 |
| NC_008463.1_5251438..5359437_00072 | 744 | t4cp2 | typeG | mandatory | 5.400e-31 | 100.400 | 0.955 | 0.319 |
| NC_008463.1_5251438..5359437_00073 | 248 | G_tfc7 | typeG | accessory | 1.100e-103 | 339.000 | 1.000 | 1.000 |
| NC_008463.1_5251438..5359437_00085 | 216 | G_tfc7 | typeG | accessory | 9.300e-96 | 313.000 | 0.871 | 1.000 |
| NC_008463.1_5251438..5359437_00090 | 112 | G_tfc8 | typeG | accessory | 3.900e-41 | 132.500 | 0.922 | 0.929 |
| NC_008463.1_5251438..5359437_00091 | 79 | G_tfc9 | typeG | accessory | 8.900e-31 | 99.000 | 1.000 | 1.000 |
| NC_008463.1_5251438..5359437_00092 | 118 | G_tfc10 | typeG | accessory | 1.200e-44 | 143.600 | 0.883 | 0.890 |
| NC_008463.1_5251438..5359437_00093 | 128 | G_tfc11 | typeG | accessory | 4.700e-50 | 161.400 | 0.923 | 0.938 |
| NC_008463.1_5251438..5359437_00094 | 219 | G_tfc12 | typeG | accessory | 1.400e-91 | 298.400 | 0.950 | 0.968 |
| NC_008463.1_5251438..5359437_00095 | 294 | G_tfc13 | typeG | accessory | 1.100e-118 | 388.500 | 0.955 | 0.956 |
| NC_008463.1_5251438..5359437_00096 | 501 | G_tfc14 | typeG | accessory | 9.300e-185 | 608.000 | 0.996 | 0.992 |
| NC_008463.1_5251438..5359437_00097 | 147 | G_tfc15 | typeG | accessory | 2.000e-59 | 192.500 | 0.957 | 0.925 |
| NC_008463.1_5251438..5359437_00098 | 980 | virb4 | typeG | mandatory | 1.600e-89 | 294.200 | 0.807 | 0.899 |
| NC_008463.1_5251438..5359437_00102 | 312 | G_tfc23 | typeG | accessory | 8.700e-134 | 438.600 | 0.973 | 0.987 |
| NC_008463.1_5251438..5359437_00103 | 460 | G_tfc22 | typeG | accessory | 8.900e-180 | 591.000 | 0.987 | 0.974 |
| NC_008463.1_5251438..5359437_00104 | 113 | G_tfc18 | typeG | accessory | 5.100e-28 | 90.100 | 0.912 | 0.885 |
| NC_008463.1_5251438..5359437_00105 | 503 | G_tfc19 | typeG | accessory | 1.300e-222 | 732.600 | 0.986 | 0.994 |
| NC_008463.1_5251438..5359437_00115 | 639 | MOBH | typeG | mandatory | 2.000e-44 | 144.500 | 0.887 | 0.275 |
| NC_009656.1_4573758..4659356_00040 | 374 | G_tfc2 | typeG | accessory | 6.000e-73 | 237.400 | 0.911 | 0.701 |
| NC_009656.1_4573758..4659356_00071 | 250 | G_tfc3 | typeG | accessory | 5.500e-96 | 313.000 | 0.967 | 0.980 |
| NC_009656.1_4573758..4659356_00073 | 166 | G_tfc5 | typeG | accessory | 3.200e-57 | 185.700 | 0.971 | 0.994 |
| NC_009656.1_4573758..4659356_00075 | 743 | t4cp2 | typeG | mandatory | 4.100e-31 | 100.700 | 0.955 | 0.318 |
| NC_009656.1_4573758..4659356_00076 | 248 | G_tfc7 | typeG | accessory | 1.000e-103 | 339.000 | 1.000 | 1.000 |
| NC_009656.1_4573758..4659356_00082 | 112 | G_tfc8 | typeG | accessory | 5.900e-41 | 131.800 | 0.931 | 0.938 |
| NC_009656.1_4573758..4659356_00083 | 79 | G_tfc9 | typeG | accessory | 2.000e-30 | 97.800 | 1.000 | 1.000 |
| NC_009656.1_4573758..4659356_00084 | 118 | G_tfc10 | typeG | accessory | 5.600e-44 | 141.300 | 0.925 | 0.924 |
| NC_009656.1_4573758..4659356_00085 | 128 | G_tfc11 | typeG | accessory | 1.000e-49 | 160.200 | 0.923 | 0.938 |
| NC_009656.1_4573758..4659356_00086 | 219 | G_tfc12 | typeG | accessory | 1.300e-91 | 298.400 | 0.950 | 0.968 |
| NC_009656.1_4573758..4659356_00087 | 294 | G_tfc13 | typeG | accessory | 1.000e-118 | 388.400 | 0.955 | 0.956 |
| NC_009656.1_4573758..4659356_00088 | 501 | G_tfc14 | typeG | accessory | 2.300e-184 | 606.600 | 0.996 | 0.992 |
| NC_009656.1_4573758..4659356_00089 | 147 | G_tfc15 | typeG | accessory | 1.700e-59 | 192.600 | 0.957 | 0.925 |
| NC_009656.1_4573758..4659356_00090 | 983 | virb4 | typeG | mandatory | 1.200e-89 | 294.500 | 0.810 | 0.899 |
| NC_009656.1_4573758..4659356_00096 | 143 | G_tfc24 | typeG | accessory | 3.400e-51 | 165.500 | 0.914 | 0.895 |
| NC_009656.1_4573758..4659356_00097 | 312 | G_tfc23 | typeG | accessory | 2.800e-134 | 440.100 | 0.973 | 0.987 |
| NC_009656.1_4573758..4659356_00098 | 460 | G_tfc22 | typeG | accessory | 6.000e-180 | 591.400 | 0.989 | 0.976 |
| NC_009656.1_4573758..4659356_00099 | 115 | G_tfc18 | typeG | accessory | 3.800e-29 | 93.600 | 0.912 | 0.887 |
| NC_009656.1_4573758..4659356_00100 | 503 | G_tfc19 | typeG | accessory | 1.300e-222 | 732.500 | 0.986 | 0.994 |
| NC_009656.1_4573758..4659356_00107 | 639 | MOBH | typeG | mandatory | 2.800e-44 | 143.900 | 0.868 | 0.268 |
| NC_018080.1_5097748..5188381_00065 | 251 | G_tfc3 | typeG | accessory | 3.300e-96 | 313.600 | 0.988 | 0.996 |
| NC_018080.1_5097748..5188381_00067 | 166 | G_tfc5 | typeG | accessory | 2.400e-57 | 186.100 | 0.971 | 0.994 |
| NC_018080.1_5097748..5188381_00069 | 743 | t4cp2 | typeG | mandatory | 4.000e-31 | 100.700 | 0.955 | 0.318 |
| NC_018080.1_5097748..5188381_00070 | 249 | G_tfc7 | typeG | accessory | 9.200e-104 | 339.100 | 1.000 | 0.996 |
| NC_018080.1_5097748..5188381_00076 | 112 | G_tfc8 | typeG | accessory | 1.300e-40 | 130.600 | 0.914 | 0.920 |
| NC_018080.1_5097748..5188381_00077 | 79 | G_tfc9 | typeG | accessory | 1.000e-30 | 98.700 | 1.000 | 1.000 |
| NC_018080.1_5097748..5188381_00078 | 118 | G_tfc10 | typeG | accessory | 6.500e-44 | 141.000 | 0.875 | 0.890 |
| NC_018080.1_5097748..5188381_00079 | 128 | G_tfc11 | typeG | accessory | 4.600e-50 | 161.300 | 0.923 | 0.938 |
| NC_018080.1_5097748..5188381_00080 | 219 | G_tfc12 | typeG | accessory | 7.200e-92 | 299.200 | 0.950 | 0.968 |
| NC_018080.1_5097748..5188381_00081 | 294 | G_tfc13 | typeG | accessory | 3.500e-118 | 386.700 | 0.955 | 0.956 |
| NC_018080.1_5097748..5188381_00082 | 501 | G_tfc14 | typeG | accessory | 9.100e-184 | 604.600 | 0.996 | 0.992 |
| NC_018080.1_5097748..5188381_00083 | 147 | G_tfc15 | typeG | accessory | 3.700e-60 | 194.700 | 0.957 | 0.925 |
| NC_018080.1_5097748..5188381_00084 | 980 | virb4 | typeG | mandatory | 1.100e-90 | 297.900 | 0.806 | 0.898 |
| NC_018080.1_5097748..5188381_00090 | 143 | G_tfc24 | typeG | accessory | 7.800e-51 | 164.400 | 0.907 | 0.888 |
| NC_018080.1_5097748..5188381_00091 | 312 | G_tfc23 | typeG | accessory | 7.800e-134 | 438.600 | 0.973 | 0.987 |
| NC_018080.1_5097748..5188381_00092 | 462 | G_tfc22 | typeG | accessory | 4.600e-180 | 591.800 | 0.987 | 0.970 |
| NC_018080.1_5097748..5188381_00093 | 115 | G_tfc18 | typeG | accessory | 3.300e-29 | 93.800 | 0.912 | 0.887 |
| NC_018080.1_5097748..5188381_00094 | 511 | G_tfc19 | typeG | accessory | 5.900e-223 | 733.600 | 0.990 | 0.982 |
| NC_018080.1_5097748..5188381_00104 | 639 | MOBH | typeG | mandatory | 4.000e-44 | 143.400 | 0.868 | 0.268 |
| NC_020912.1_5129453..5234036_00060 | 251 | G_tfc3 | typeG | accessory | 8.900e-96 | 312.400 | 0.988 | 0.996 |
| NC_020912.1_5129453..5234036_00062 | 166 | G_tfc5 | typeG | accessory | 6.100e-57 | 185.000 | 0.971 | 0.988 |
| NC_020912.1_5129453..5234036_00064 | 743 | t4cp2 | typeG | mandatory | 4.600e-31 | 100.700 | 0.955 | 0.318 |
| NC_020912.1_5129453..5234036_00065 | 248 | G_tfc7 | typeG | accessory | 4.200e-103 | 337.100 | 1.000 | 1.000 |
| NC_020912.1_5129453..5234036_00068 | 112 | G_tfc8 | typeG | accessory | 1.500e-40 | 130.600 | 0.931 | 0.938 |
| NC_020912.1_5129453..5234036_00069 | 79 | G_tfc9 | typeG | accessory | 9.100e-31 | 99.000 | 1.000 | 1.000 |
| NC_020912.1_5129453..5234036_00070 | 118 | G_tfc10 | typeG | accessory | 6.300e-42 | 134.800 | 0.858 | 0.873 |
| NC_020912.1_5129453..5234036_00071 | 128 | G_tfc11 | typeG | accessory | 4.700e-50 | 161.400 | 0.923 | 0.938 |
| NC_020912.1_5129453..5234036_00072 | 219 | G_tfc12 | typeG | accessory | 6.500e-92 | 299.500 | 0.950 | 0.968 |
| NC_020912.1_5129453..5234036_00073 | 294 | G_tfc13 | typeG | accessory | 2.400e-118 | 387.400 | 0.955 | 0.956 |
| NC_020912.1_5129453..5234036_00074 | 501 | G_tfc14 | typeG | accessory | 1.100e-184 | 607.800 | 0.996 | 0.992 |
| NC_020912.1_5129453..5234036_00075 | 147 | G_tfc15 | typeG | accessory | 3.900e-60 | 194.800 | 0.957 | 0.925 |
| NC_020912.1_5129453..5234036_00076 | 980 | virb4 | typeG | mandatory | 1.800e-90 | 297.400 | 0.807 | 0.899 |
| NC_020912.1_5129453..5234036_00083 | 143 | G_tfc24 | typeG | accessory | 2.900e-51 | 165.900 | 0.914 | 0.895 |
| NC_020912.1_5129453..5234036_00084 | 312 | G_tfc23 | typeG | accessory | 1.600e-133 | 437.800 | 0.976 | 0.990 |
| NC_020912.1_5129453..5234036_00085 | 460 | G_tfc22 | typeG | accessory | 4.000e-180 | 592.200 | 0.987 | 0.974 |
| NC_020912.1_5129453..5234036_00086 | 105 | G_tfc18 | typeG | accessory | 5.700e-20 | 64.200 | 0.912 | 0.876 |
| NC_020912.1_5129453..5234036_00087 | 511 | G_tfc19 | typeG | accessory | 1.700e-222 | 732.300 | 0.990 | 0.982 |
| NC_020912.1_5129453..5234036_00119 | 639 | MOBH | typeG | mandatory | 4.700e-44 | 143.300 | 0.868 | 0.268 |
| NC_021577.1_1023533..1126247_00002 | 639 | MOBH | typeG | mandatory | 3.000e-44 | 143.900 | 0.873 | 0.271 |
| NC_021577.1_1023533..1126247_00013 | 511 | G_tfc19 | typeG | accessory | 1.200e-222 | 732.700 | 0.990 | 0.982 |
| NC_021577.1_1023533..1126247_00014 | 115 | G_tfc18 | typeG | accessory | 9.500e-29 | 92.400 | 0.912 | 0.887 |
| NC_021577.1_1023533..1126247_00015 | 460 | G_tfc22 | typeG | accessory | 8.600e-180 | 591.000 | 0.987 | 0.974 |
| NC_021577.1_1023533..1126247_00016 | 312 | G_tfc23 | typeG | accessory | 3.400e-134 | 439.900 | 0.973 | 0.987 |
| NC_021577.1_1023533..1126247_00023 | 980 | virb4 | typeG | mandatory | 2.200e-89 | 293.700 | 0.807 | 0.899 |
| NC_021577.1_1023533..1126247_00024 | 147 | G_tfc15 | typeG | accessory | 7.100e-60 | 193.900 | 0.957 | 0.925 |
| NC_021577.1_1023533..1126247_00025 | 501 | G_tfc14 | typeG | accessory | 1.400e-184 | 607.400 | 0.996 | 0.992 |
| NC_021577.1_1023533..1126247_00026 | 294 | G_tfc13 | typeG | accessory | 7.400e-119 | 389.000 | 0.944 | 0.959 |
| NC_021577.1_1023533..1126247_00027 | 219 | G_tfc12 | typeG | accessory | 6.300e-91 | 296.200 | 0.941 | 0.959 |
| NC_021577.1_1023533..1126247_00028 | 128 | G_tfc11 | typeG | accessory | 8.500e-50 | 160.500 | 0.923 | 0.938 |
| NC_021577.1_1023533..1126247_00029 | 118 | G_tfc10 | typeG | accessory | 3.000e-44 | 142.200 | 0.875 | 0.890 |
| NC_021577.1_1023533..1126247_00030 | 79 | G_tfc9 | typeG | accessory | 8.600e-31 | 99.000 | 1.000 | 1.000 |
| NC_021577.1_1023533..1126247_00031 | 112 | G_tfc8 | typeG | accessory | 6.400e-41 | 131.700 | 0.922 | 0.929 |
| NC_021577.1_1023533..1126247_00035 | 248 | G_tfc7 | typeG | accessory | 7.900e-104 | 339.400 | 1.000 | 1.000 |
| NC_021577.1_1023533..1126247_00036 | 743 | t4cp2 | typeG | mandatory | 4.300e-31 | 100.700 | 0.955 | 0.318 |
| NC_021577.1_1023533..1126247_00038 | 166 | G_tfc5 | typeG | accessory | 2.600e-57 | 186.100 | 0.971 | 0.994 |
| NC_021577.1_1023533..1126247_00040 | 251 | G_tfc3 | typeG | accessory | 7.200e-97 | 315.900 | 0.988 | 0.996 |
| NC_022806.1_5021959..5124759_00002 | 639 | MOBH | typeG | mandatory | 2.800e-44 | 143.900 | 0.868 | 0.268 |
| NC_022806.1_5021959..5124759_00007 | 504 | G_tfc19 | typeG | accessory | 1.500e-222 | 732.400 | 0.984 | 0.990 |
| NC_022806.1_5021959..5124759_00008 | 115 | G_tfc18 | typeG | accessory | 3.400e-29 | 93.800 | 0.912 | 0.887 |
| NC_022806.1_5021959..5124759_00009 | 462 | G_tfc22 | typeG | accessory | 1.700e-179 | 590.000 | 0.987 | 0.970 |
| NC_022806.1_5021959..5124759_00010 | 312 | G_tfc23 | typeG | accessory | 7.600e-133 | 435.500 | 0.973 | 0.987 |
| NC_022806.1_5021959..5124759_00011 | 143 | G_tfc24 | typeG | accessory | 8.700e-51 | 164.300 | 0.914 | 0.895 |
| NC_022806.1_5021959..5124759_00015 | 980 | virb4 | typeG | mandatory | 1.900e-89 | 293.900 | 0.805 | 0.897 |
| NC_022806.1_5021959..5124759_00016 | 147 | G_tfc15 | typeG | accessory | 7.400e-60 | 193.800 | 0.957 | 0.925 |
| NC_022806.1_5021959..5124759_00017 | 501 | G_tfc14 | typeG | accessory | 7.300e-185 | 608.300 | 0.996 | 0.992 |
| NC_022806.1_5021959..5124759_00018 | 294 | G_tfc13 | typeG | accessory | 5.400e-118 | 386.100 | 0.955 | 0.956 |
| NC_022806.1_5021959..5124759_00019 | 219 | G_tfc12 | typeG | accessory | 3.100e-91 | 297.200 | 0.941 | 0.959 |
| NC_022806.1_5021959..5124759_00020 | 128 | G_tfc11 | typeG | accessory | 3.500e-49 | 158.500 | 0.923 | 0.938 |
| NC_022806.1_5021959..5124759_00021 | 118 | G_tfc10 | typeG | accessory | 4.100e-44 | 141.700 | 0.925 | 0.924 |
| NC_022806.1_5021959..5124759_00022 | 79 | G_tfc9 | typeG | accessory | 3.200e-31 | 100.400 | 1.000 | 1.000 |
| NC_022806.1_5021959..5124759_00023 | 112 | G_tfc8 | typeG | accessory | 1.200e-40 | 130.800 | 0.922 | 0.929 |
| NC_022806.1_5021959..5124759_00034 | 248 | G_tfc7 | typeG | accessory | 8.900e-104 | 339.200 | 1.000 | 1.000 |
| NC_022806.1_5021959..5124759_00035 | 743 | t4cp2 | typeG | mandatory | 2.000e-31 | 101.800 | 0.955 | 0.318 |
| NC_022806.1_5021959..5124759_00037 | 166 | G_tfc5 | typeG | accessory | 2.500e-57 | 186.100 | 0.971 | 0.994 |
| NC_022806.1_5021959..5124759_00039 | 251 | G_tfc3 | typeG | accessory | 1.700e-94 | 308.100 | 0.988 | 0.996 |
| NC_022808.2_4518241..4621030_00071 | 251 | G_tfc3 | typeG | accessory | 1.700e-94 | 308.100 | 0.988 | 0.996 |
| NC_022808.2_4518241..4621030_00073 | 166 | G_tfc5 | typeG | accessory | 2.500e-57 | 186.100 | 0.971 | 0.994 |
| NC_022808.2_4518241..4621030_00075 | 743 | t4cp2 | typeG | mandatory | 1.900e-31 | 101.800 | 0.955 | 0.318 |
| NC_022808.2_4518241..4621030_00076 | 248 | G_tfc7 | typeG | accessory | 8.800e-104 | 339.200 | 1.000 | 1.000 |
| NC_022808.2_4518241..4621030_00087 | 112 | G_tfc8 | typeG | accessory | 1.200e-40 | 130.800 | 0.922 | 0.929 |
| NC_022808.2_4518241..4621030_00088 | 79 | G_tfc9 | typeG | accessory | 3.200e-31 | 100.400 | 1.000 | 1.000 |
| NC_022808.2_4518241..4621030_00089 | 118 | G_tfc10 | typeG | accessory | 4.000e-44 | 141.700 | 0.925 | 0.924 |
| NC_022808.2_4518241..4621030_00090 | 128 | G_tfc11 | typeG | accessory | 3.500e-49 | 158.500 | 0.923 | 0.938 |
| NC_022808.2_4518241..4621030_00091 | 219 | G_tfc12 | typeG | accessory | 3.000e-91 | 297.200 | 0.941 | 0.959 |
| NC_022808.2_4518241..4621030_00092 | 294 | G_tfc13 | typeG | accessory | 5.400e-118 | 386.100 | 0.955 | 0.956 |
| NC_022808.2_4518241..4621030_00093 | 501 | G_tfc14 | typeG | accessory | 7.200e-185 | 608.300 | 0.996 | 0.992 |
| NC_022808.2_4518241..4621030_00094 | 147 | G_tfc15 | typeG | accessory | 7.300e-60 | 193.800 | 0.957 | 0.925 |
| NC_022808.2_4518241..4621030_00095 | 980 | virb4 | typeG | mandatory | 1.900e-89 | 293.900 | 0.805 | 0.897 |
| NC_022808.2_4518241..4621030_00099 | 143 | G_tfc24 | typeG | accessory | 8.600e-51 | 164.300 | 0.914 | 0.895 |
| NC_022808.2_4518241..4621030_00100 | 312 | G_tfc23 | typeG | accessory | 7.500e-133 | 435.500 | 0.973 | 0.987 |
| NC_022808.2_4518241..4621030_00101 | 462 | G_tfc22 | typeG | accessory | 1.700e-179 | 590.000 | 0.987 | 0.970 |
| NC_022808.2_4518241..4621030_00102 | 115 | G_tfc18 | typeG | accessory | 3.400e-29 | 93.800 | 0.912 | 0.887 |
| NC_022808.2_4518241..4621030_00103 | 504 | G_tfc19 | typeG | accessory | 1.500e-222 | 732.400 | 0.984 | 0.990 |
| NC_022808.2_4518241..4621030_00108 | 639 | MOBH | typeG | mandatory | 2.800e-44 | 143.900 | 0.868 | 0.268 |
| NC_023019.1_5252992..5341025_00072 | 251 | G_tfc3 | typeG | accessory | 1.100e-95 | 312.000 | 0.967 | 0.976 |
| NC_023019.1_5252992..5341025_00074 | 166 | G_tfc5 | typeG | accessory | 2.400e-57 | 186.100 | 0.977 | 1.000 |
| NC_023019.1_5252992..5341025_00076 | 743 | t4cp2 | typeG | mandatory | 4.000e-31 | 100.700 | 0.955 | 0.318 |
| NC_023019.1_5252992..5341025_00077 | 248 | G_tfc7 | typeG | accessory | 2.000e-103 | 337.900 | 1.000 | 1.000 |
| NC_023019.1_5252992..5341025_00081 | 112 | G_tfc8 | typeG | accessory | 5.900e-41 | 131.700 | 0.922 | 0.929 |
| NC_023019.1_5252992..5341025_00082 | 79 | G_tfc9 | typeG | accessory | 3.800e-31 | 100.100 | 1.000 | 1.000 |
| NC_023019.1_5252992..5341025_00083 | 118 | G_tfc10 | typeG | accessory | 1.000e-44 | 143.600 | 0.883 | 0.890 |
| NC_023019.1_5252992..5341025_00084 | 128 | G_tfc11 | typeG | accessory | 5.800e-50 | 160.900 | 0.923 | 0.938 |
| NC_023019.1_5252992..5341025_00085 | 219 | G_tfc12 | typeG | accessory | 1.300e-91 | 298.400 | 0.950 | 0.968 |
| NC_023019.1_5252992..5341025_00086 | 294 | G_tfc13 | typeG | accessory | 4.900e-119 | 389.500 | 0.955 | 0.956 |
| NC_023019.1_5252992..5341025_00087 | 501 | G_tfc14 | typeG | accessory | 9.900e-185 | 607.800 | 0.996 | 0.992 |
| NC_023019.1_5252992..5341025_00088 | 147 | G_tfc15 | typeG | accessory | 2.000e-59 | 192.400 | 0.957 | 0.925 |
| NC_023019.1_5252992..5341025_00089 | 980 | virb4 | typeG | mandatory | 1.100e-89 | 294.600 | 0.807 | 0.899 |
| NC_023019.1_5252992..5341025_00093 | 143 | G_tfc24 | typeG | accessory | 5.200e-51 | 164.900 | 0.914 | 0.895 |
| NC_023019.1_5252992..5341025_00094 | 312 | G_tfc23 | typeG | accessory | 7.700e-134 | 438.600 | 0.973 | 0.987 |
| NC_023019.1_5252992..5341025_00095 | 460 | G_tfc22 | typeG | accessory | 2.100e-180 | 592.900 | 0.987 | 0.974 |
| NC_023019.1_5252992..5341025_00096 | 117 | G_tfc18 | typeG | accessory | 7.400e-28 | 89.400 | 0.895 | 0.880 |
| NC_023019.1_5252992..5341025_00097 | 504 | G_tfc19 | typeG | accessory | 1.500e-222 | 732.200 | 0.984 | 0.990 |
| NC_023019.1_5252992..5341025_00102 | 639 | MOBH | typeG | mandatory | 3.400e-44 | 143.600 | 0.868 | 0.268 |
| NC_023149.1_4723738..4816893_00077 | 251 | G_tfc3 | typeG | accessory | 3.800e-96 | 313.600 | 0.988 | 0.996 |
| NC_023149.1_4723738..4816893_00079 | 166 | G_tfc5 | typeG | accessory | 2.700e-57 | 186.100 | 0.971 | 0.994 |
| NC_023149.1_4723738..4816893_00081 | 743 | t4cp2 | typeG | mandatory | 4.600e-31 | 100.700 | 0.955 | 0.318 |
| NC_023149.1_4723738..4816893_00082 | 249 | G_tfc7 | typeG | accessory | 1.100e-103 | 339.100 | 1.000 | 0.996 |
| NC_023149.1_4723738..4816893_00088 | 112 | G_tfc8 | typeG | accessory | 1.500e-40 | 130.600 | 0.914 | 0.920 |
| NC_023149.1_4723738..4816893_00089 | 79 | G_tfc9 | typeG | accessory | 1.200e-30 | 98.700 | 1.000 | 1.000 |
| NC_023149.1_4723738..4816893_00090 | 118 | G_tfc10 | typeG | accessory | 7.400e-44 | 141.000 | 0.875 | 0.890 |
| NC_023149.1_4723738..4816893_00091 | 128 | G_tfc11 | typeG | accessory | 5.300e-50 | 161.300 | 0.923 | 0.938 |
| NC_023149.1_4723738..4816893_00092 | 219 | G_tfc12 | typeG | accessory | 8.300e-92 | 299.200 | 0.950 | 0.968 |
| NC_023149.1_4723738..4816893_00093 | 294 | G_tfc13 | typeG | accessory | 1.800e-118 | 387.800 | 0.955 | 0.956 |
| NC_023149.1_4723738..4816893_00094 | 501 | G_tfc14 | typeG | accessory | 1.100e-184 | 607.800 | 0.996 | 0.992 |
| NC_023149.1_4723738..4816893_00095 | 147 | G_tfc15 | typeG | accessory | 4.200e-60 | 194.700 | 0.957 | 0.925 |
| NC_023149.1_4723738..4816893_00096 | 980 | virb4 | typeG | mandatory | 1.300e-90 | 297.900 | 0.806 | 0.898 |
| NC_023149.1_4723738..4816893_00108 | 143 | G_tfc24 | typeG | accessory | 8.900e-51 | 164.400 | 0.907 | 0.888 |
| NC_023149.1_4723738..4816893_00109 | 312 | G_tfc23 | typeG | accessory | 3.500e-134 | 440.000 | 0.973 | 0.987 |
| NC_023149.1_4723738..4816893_00110 | 460 | G_tfc22 | typeG | accessory | 5.100e-180 | 591.800 | 0.987 | 0.974 |
| NC_023149.1_4723738..4816893_00111 | 115 | G_tfc18 | typeG | accessory | 4.000e-29 | 93.700 | 0.912 | 0.887 |
| NC_023149.1_4723738..4816893_00112 | 504 | G_tfc19 | typeG | accessory | 1.500e-222 | 732.500 | 0.984 | 0.990 |
| NC_023149.1_4723738..4816893_00116 | 510 | MOBH | typeG | mandatory | 2.800e-44 | 144.000 | 0.868 | 0.335 |
| NZ_AP014651.1_5699825..5782875_00073 | 251 | G_tfc3 | typeG | accessory | 6.800e-96 | 312.600 | 0.988 | 0.996 |
| NZ_AP014651.1_5699825..5782875_00075 | 166 | G_tfc5 | typeG | accessory | 2.400e-57 | 186.100 | 0.971 | 0.994 |
| NZ_AP014651.1_5699825..5782875_00077 | 743 | t4cp2 | typeG | mandatory | 4.000e-31 | 100.700 | 0.955 | 0.318 |
| NZ_AP014651.1_5699825..5782875_00078 | 248 | G_tfc7 | typeG | accessory | 8.400e-104 | 339.200 | 1.000 | 1.000 |
| NZ_AP014651.1_5699825..5782875_00081 | 112 | G_tfc8 | typeG | accessory | 1.200e-40 | 130.800 | 0.922 | 0.929 |
| NZ_AP014651.1_5699825..5782875_00082 | 79 | G_tfc9 | typeG | accessory | 3.800e-31 | 100.100 | 1.000 | 1.000 |
| NZ_AP014651.1_5699825..5782875_00083 | 118 | G_tfc10 | typeG | accessory | 2.500e-43 | 139.100 | 0.867 | 0.881 |
| NZ_AP014651.1_5699825..5782875_00084 | 128 | G_tfc11 | typeG | accessory | 4.600e-50 | 161.300 | 0.923 | 0.938 |
| NZ_AP014651.1_5699825..5782875_00085 | 219 | G_tfc12 | typeG | accessory | 2.400e-91 | 297.500 | 0.946 | 0.963 |
| NZ_AP014651.1_5699825..5782875_00086 | 294 | G_tfc13 | typeG | accessory | 2.900e-118 | 387.000 | 0.951 | 0.956 |
| NZ_AP014651.1_5699825..5782875_00087 | 501 | G_tfc14 | typeG | accessory | 7.700e-185 | 608.200 | 0.996 | 0.992 |
| NZ_AP014651.1_5699825..5782875_00088 | 147 | G_tfc15 | typeG | accessory | 1.400e-59 | 192.900 | 0.957 | 0.925 |
| NZ_AP014651.1_5699825..5782875_00089 | 980 | virb4 | typeG | mandatory | 4.200e-89 | 292.600 | 0.806 | 0.898 |
| NZ_AP014651.1_5699825..5782875_00095 | 143 | G_tfc24 | typeG | accessory | 3.300e-51 | 165.500 | 0.914 | 0.895 |
| NZ_AP014651.1_5699825..5782875_00096 | 312 | G_tfc23 | typeG | accessory | 2.800e-134 | 440.100 | 0.973 | 0.987 |
| NZ_AP014651.1_5699825..5782875_00097 | 460 | G_tfc22 | typeG | accessory | 5.900e-180 | 591.400 | 0.989 | 0.976 |
| NZ_AP014651.1_5699825..5782875_00098 | 115 | G_tfc18 | typeG | accessory | 3.600e-29 | 93.600 | 0.912 | 0.887 |
| NZ_AP014651.1_5699825..5782875_00099 | 503 | G_tfc19 | typeG | accessory | 1.300e-222 | 732.500 | 0.986 | 0.994 |
| NZ_AP014651.1_5699825..5782875_00104 | 640 | MOBH | typeG | mandatory | 2.900e-44 | 143.800 | 0.868 | 0.267 |
| NZ_AP014839.1_5301494..5407295_00072 | 251 | G_tfc3 | typeG | accessory | 7.200e-97 | 315.900 | 0.988 | 0.996 |
| NZ_AP014839.1_5301494..5407295_00074 | 166 | G_tfc5 | typeG | accessory | 2.600e-57 | 186.100 | 0.971 | 0.994 |
| NZ_AP014839.1_5301494..5407295_00076 | 743 | t4cp2 | typeG | mandatory | 4.300e-31 | 100.700 | 0.955 | 0.318 |
| NZ_AP014839.1_5301494..5407295_00077 | 248 | G_tfc7 | typeG | accessory | 8.000e-104 | 339.400 | 1.000 | 1.000 |
| NZ_AP014839.1_5301494..5407295_00083 | 112 | G_tfc8 | typeG | accessory | 6.500e-41 | 131.700 | 0.922 | 0.929 |
| NZ_AP014839.1_5301494..5407295_00084 | 79 | G_tfc9 | typeG | accessory | 8.700e-31 | 99.000 | 1.000 | 1.000 |
| NZ_AP014839.1_5301494..5407295_00085 | 118 | G_tfc10 | typeG | accessory | 3.000e-44 | 142.200 | 0.875 | 0.890 |
| NZ_AP014839.1_5301494..5407295_00086 | 128 | G_tfc11 | typeG | accessory | 8.600e-50 | 160.500 | 0.923 | 0.938 |
| NZ_AP014839.1_5301494..5407295_00087 | 219 | G_tfc12 | typeG | accessory | 6.400e-91 | 296.200 | 0.941 | 0.959 |
| NZ_AP014839.1_5301494..5407295_00088 | 294 | G_tfc13 | typeG | accessory | 7.500e-119 | 389.000 | 0.944 | 0.959 |
| NZ_AP014839.1_5301494..5407295_00089 | 501 | G_tfc14 | typeG | accessory | 1.400e-184 | 607.400 | 0.996 | 0.992 |
| NZ_AP014839.1_5301494..5407295_00090 | 147 | G_tfc15 | typeG | accessory | 7.200e-60 | 193.900 | 0.957 | 0.925 |
| NZ_AP014839.1_5301494..5407295_00091 | 980 | virb4 | typeG | mandatory | 2.200e-89 | 293.700 | 0.807 | 0.899 |
| NZ_AP014839.1_5301494..5407295_00098 | 143 | G_tfc24 | typeG | accessory | 8.900e-51 | 164.300 | 0.914 | 0.895 |
| NZ_AP014839.1_5301494..5407295_00099 | 312 | G_tfc23 | typeG | accessory | 3.400e-134 | 439.900 | 0.973 | 0.987 |
| NZ_AP014839.1_5301494..5407295_00100 | 460 | G_tfc22 | typeG | accessory | 8.700e-180 | 591.000 | 0.987 | 0.974 |
| NZ_AP014839.1_5301494..5407295_00101 | 115 | G_tfc18 | typeG | accessory | 9.600e-29 | 92.400 | 0.912 | 0.887 |
| NZ_AP014839.1_5301494..5407295_00102 | 511 | G_tfc19 | typeG | accessory | 1.200e-222 | 732.700 | 0.990 | 0.982 |
| NZ_AP014839.1_5301494..5407295_00113 | 639 | MOBH | typeG | mandatory | 3.000e-44 | 143.900 | 0.873 | 0.271 |
| NZ_CP007147.1_2684203..2769071_00002 | 639 | MOBH | typeG | mandatory | 2.700e-44 | 143.900 | 0.868 | 0.268 |
| NZ_CP007147.1_2684203..2769071_00007 | 503 | G_tfc19 | typeG | accessory | 4.100e-222 | 730.800 | 0.984 | 0.992 |
| NZ_CP007147.1_2684203..2769071_00008 | 115 | G_tfc18 | typeG | accessory | 3.300e-29 | 93.800 | 0.912 | 0.887 |
| NZ_CP007147.1_2684203..2769071_00009 | 462 | G_tfc22 | typeG | accessory | 1.200e-179 | 590.400 | 0.987 | 0.970 |
| NZ_CP007147.1_2684203..2769071_00010 | 312 | G_tfc23 | typeG | accessory | 5.600e-134 | 439.100 | 0.973 | 0.987 |
| NZ_CP007147.1_2684203..2769071_00011 | 143 | G_tfc24 | typeG | accessory | 8.200e-51 | 164.300 | 0.914 | 0.895 |
| NZ_CP007147.1_2684203..2769071_00017 | 980 | virb4 | typeG | mandatory | 4.300e-90 | 295.900 | 0.807 | 0.899 |
| NZ_CP007147.1_2684203..2769071_00018 | 147 | G_tfc15 | typeG | accessory | 3.400e-60 | 194.800 | 0.957 | 0.925 |
| NZ_CP007147.1_2684203..2769071_00019 | 501 | G_tfc14 | typeG | accessory | 6.400e-185 | 608.400 | 0.996 | 0.992 |
| NZ_CP007147.1_2684203..2769071_00020 | 294 | G_tfc13 | typeG | accessory | 6.800e-118 | 385.700 | 0.955 | 0.956 |
| NZ_CP007147.1_2684203..2769071_00021 | 219 | G_tfc12 | typeG | accessory | 2.600e-91 | 297.400 | 0.950 | 0.968 |
| NZ_CP007147.1_2684203..2769071_00022 | 128 | G_tfc11 | typeG | accessory | 9.600e-50 | 160.200 | 0.923 | 0.938 |
| NZ_CP007147.1_2684203..2769071_00023 | 118 | G_tfc10 | typeG | accessory | 4.900e-44 | 141.400 | 0.883 | 0.890 |
| NZ_CP007147.1_2684203..2769071_00024 | 79 | G_tfc9 | typeG | accessory | 7.900e-31 | 99.000 | 1.000 | 1.000 |
| NZ_CP007147.1_2684203..2769071_00025 | 112 | G_tfc8 | typeG | accessory | 2.900e-41 | 132.700 | 0.922 | 0.929 |
| NZ_CP007147.1_2684203..2769071_00033 | 248 | G_tfc7 | typeG | accessory | 2.300e-103 | 337.800 | 1.000 | 1.000 |
| NZ_CP007147.1_2684203..2769071_00034 | 743 | t4cp2 | typeG | mandatory | 1.300e-30 | 99.000 | 0.955 | 0.318 |
| NZ_CP007147.1_2684203..2769071_00036 | 166 | G_tfc5 | typeG | accessory | 2.400e-57 | 186.100 | 0.971 | 0.994 |
| NZ_CP007147.1_2684203..2769071_00038 | 251 | G_tfc3 | typeG | accessory | 2.400e-95 | 310.800 | 0.967 | 0.976 |
| NZ_CP008856.2_5221858..5338071_00084 | 251 | G_tfc3 | typeG | accessory | 8.900e-96 | 312.500 | 0.967 | 0.976 |
| NZ_CP008856.2_5221858..5338071_00086 | 166 | G_tfc5 | typeG | accessory | 6.000e-55 | 178.600 | 0.971 | 0.994 |
| NZ_CP008856.2_5221858..5338071_00088 | 743 | t4cp2 | typeG | mandatory | 4.800e-31 | 100.700 | 0.955 | 0.318 |
| NZ_CP008856.2_5221858..5338071_00089 | 248 | G_tfc7 | typeG | accessory | 5.000e-103 | 337.000 | 1.000 | 1.000 |
| NZ_CP008856.2_5221858..5338071_00094 | 112 | G_tfc8 | typeG | accessory | 2.000e-41 | 133.500 | 0.931 | 0.938 |
| NZ_CP008856.2_5221858..5338071_00095 | 79 | G_tfc9 | typeG | accessory | 1.400e-30 | 98.500 | 1.000 | 1.000 |
| NZ_CP008856.2_5221858..5338071_00096 | 118 | G_tfc10 | typeG | accessory | 7.800e-44 | 141.000 | 0.875 | 0.890 |
| NZ_CP008856.2_5221858..5338071_00097 | 128 | G_tfc11 | typeG | accessory | 5.600e-50 | 161.300 | 0.923 | 0.938 |
| NZ_CP008856.2_5221858..5338071_00098 | 219 | G_tfc12 | typeG | accessory | 8.700e-92 | 299.200 | 0.950 | 0.968 |
| NZ_CP008856.2_5221858..5338071_00099 | 294 | G_tfc13 | typeG | accessory | 4.300e-118 | 386.700 | 0.955 | 0.956 |
| NZ_CP008856.2_5221858..5338071_00100 | 501 | G_tfc14 | typeG | accessory | 1.100e-183 | 604.600 | 0.996 | 0.992 |
| NZ_CP008856.2_5221858..5338071_00101 | 147 | G_tfc15 | typeG | accessory | 4.400e-60 | 194.700 | 0.957 | 0.925 |
| NZ_CP008856.2_5221858..5338071_00102 | 980 | virb4 | typeG | mandatory | 1.400e-90 | 297.800 | 0.806 | 0.898 |
| NZ_CP008856.2_5221858..5338071_00107 | 143 | G_tfc24 | typeG | accessory | 9.400e-51 | 164.400 | 0.907 | 0.888 |
| NZ_CP008856.2_5221858..5338071_00108 | 312 | G_tfc23 | typeG | accessory | 9.400e-134 | 438.600 | 0.973 | 0.987 |
| NZ_CP008856.2_5221858..5338071_00109 | 462 | G_tfc22 | typeG | accessory | 5.500e-180 | 591.800 | 0.987 | 0.970 |
| NZ_CP008856.2_5221858..5338071_00110 | 115 | G_tfc18 | typeG | accessory | 4.000e-29 | 93.800 | 0.912 | 0.887 |
| NZ_CP008856.2_5221858..5338071_00111 | 511 | G_tfc19 | typeG | accessory | 7.100e-223 | 733.600 | 0.990 | 0.982 |
| NZ_CP008856.2_5221858..5338071_00126 | 639 | MOBH | typeG | mandatory | 2.100e-43 | 141.300 | 0.868 | 0.268 |
| NZ_CP008857.1_3269423..3355117_00035 | 374 | G_tfc2 | typeG | accessory | 1.300e-71 | 232.900 | 0.782 | 0.631 |
| NZ_CP008857.1_3269423..3355117_00066 | 251 | G_tfc3 | typeG | accessory | 6.900e-96 | 312.600 | 0.967 | 0.976 |
| NZ_CP008857.1_3269423..3355117_00068 | 166 | G_tfc5 | typeG | accessory | 2.500e-57 | 186.000 | 0.971 | 0.994 |
| NZ_CP008857.1_3269423..3355117_00070 | 743 | t4cp2 | typeG | mandatory | 3.900e-31 | 100.700 | 0.955 | 0.318 |
| NZ_CP008857.1_3269423..3355117_00071 | 248 | G_tfc7 | typeG | accessory | 9.500e-104 | 339.000 | 1.000 | 1.000 |
| NZ_CP008857.1_3269423..3355117_00077 | 112 | G_tfc8 | typeG | accessory | 1.600e-41 | 133.500 | 0.931 | 0.938 |
| NZ_CP008857.1_3269423..3355117_00078 | 79 | G_tfc9 | typeG | accessory | 1.200e-30 | 98.500 | 1.000 | 1.000 |
| NZ_CP008857.1_3269423..3355117_00079 | 118 | G_tfc10 | typeG | accessory | 6.400e-44 | 141.000 | 0.875 | 0.890 |
| NZ_CP008857.1_3269423..3355117_00080 | 128 | G_tfc11 | typeG | accessory | 4.500e-50 | 161.300 | 0.923 | 0.938 |
| NZ_CP008857.1_3269423..3355117_00081 | 219 | G_tfc12 | typeG | accessory | 7.100e-92 | 299.200 | 0.950 | 0.968 |
| NZ_CP008857.1_3269423..3355117_00082 | 294 | G_tfc13 | typeG | accessory | 3.500e-118 | 386.700 | 0.955 | 0.956 |
| NZ_CP008857.1_3269423..3355117_00083 | 501 | G_tfc14 | typeG | accessory | 8.900e-184 | 604.600 | 0.996 | 0.992 |
| NZ_CP008857.1_3269423..3355117_00084 | 147 | G_tfc15 | typeG | accessory | 3.600e-60 | 194.700 | 0.957 | 0.925 |
| NZ_CP008857.1_3269423..3355117_00085 | 980 | virb4 | typeG | mandatory | 1.100e-90 | 297.900 | 0.806 | 0.898 |
| NZ_CP008857.1_3269423..3355117_00091 | 143 | G_tfc24 | typeG | accessory | 7.600e-51 | 164.400 | 0.907 | 0.888 |
| NZ_CP008857.1_3269423..3355117_00092 | 312 | G_tfc23 | typeG | accessory | 7.600e-134 | 438.600 | 0.973 | 0.987 |
| NZ_CP008857.1_3269423..3355117_00093 | 460 | G_tfc22 | typeG | accessory | 4.400e-180 | 591.800 | 0.987 | 0.974 |
| NZ_CP008857.1_3269423..3355117_00094 | 115 | G_tfc18 | typeG | accessory | 2.600e-29 | 94.100 | 0.912 | 0.887 |
| NZ_CP008857.1_3269423..3355117_00095 | 511 | G_tfc19 | typeG | accessory | 5.800e-223 | 733.600 | 0.990 | 0.982 |
| NZ_CP008857.1_3269423..3355117_00102 | 640 | MOBH | typeG | mandatory | 2.700e-44 | 143.900 | 0.868 | 0.267 |
| NZ_CP008858.2_5314589..5418165_00070 | 251 | G_tfc3 | typeG | accessory | 6.900e-97 | 315.900 | 0.988 | 0.996 |
| NZ_CP008858.2_5314589..5418165_00072 | 166 | G_tfc5 | typeG | accessory | 2.500e-57 | 186.100 | 0.971 | 0.994 |
| NZ_CP008858.2_5314589..5418165_00074 | 743 | t4cp2 | typeG | mandatory | 4.100e-31 | 100.700 | 0.955 | 0.318 |
| NZ_CP008858.2_5314589..5418165_00075 | 248 | G_tfc7 | typeG | accessory | 7.700e-104 | 339.400 | 1.000 | 1.000 |
| NZ_CP008858.2_5314589..5418165_00079 | 112 | G_tfc8 | typeG | accessory | 6.200e-41 | 131.700 | 0.922 | 0.929 |
| NZ_CP008858.2_5314589..5418165_00080 | 79 | G_tfc9 | typeG | accessory | 8.300e-31 | 99.000 | 1.000 | 1.000 |
| NZ_CP008858.2_5314589..5418165_00081 | 118 | G_tfc10 | typeG | accessory | 2.800e-44 | 142.200 | 0.875 | 0.890 |
| NZ_CP008858.2_5314589..5418165_00082 | 128 | G_tfc11 | typeG | accessory | 8.200e-50 | 160.500 | 0.923 | 0.938 |
| NZ_CP008858.2_5314589..5418165_00083 | 219 | G_tfc12 | typeG | accessory | 6.100e-91 | 296.200 | 0.941 | 0.959 |
| NZ_CP008858.2_5314589..5418165_00084 | 294 | G_tfc13 | typeG | accessory | 7.200e-119 | 389.000 | 0.944 | 0.959 |
| NZ_CP008858.2_5314589..5418165_00085 | 501 | G_tfc14 | typeG | accessory | 1.300e-184 | 607.400 | 0.996 | 0.992 |
| NZ_CP008858.2_5314589..5418165_00086 | 147 | G_tfc15 | typeG | accessory | 6.900e-60 | 193.900 | 0.957 | 0.925 |
| NZ_CP008858.2_5314589..5418165_00087 | 980 | virb4 | typeG | mandatory | 2.100e-89 | 293.700 | 0.807 | 0.899 |
| NZ_CP008858.2_5314589..5418165_00094 | 312 | G_tfc23 | typeG | accessory | 3.300e-134 | 439.900 | 0.973 | 0.987 |
| NZ_CP008858.2_5314589..5418165_00095 | 460 | G_tfc22 | typeG | accessory | 8.300e-180 | 591.000 | 0.987 | 0.974 |
| NZ_CP008858.2_5314589..5418165_00096 | 115 | G_tfc18 | typeG | accessory | 9.100e-29 | 92.400 | 0.912 | 0.887 |
| NZ_CP008858.2_5314589..5418165_00097 | 511 | G_tfc19 | typeG | accessory | 1.100e-222 | 732.700 | 0.990 | 0.982 |
| NZ_CP008858.2_5314589..5418165_00108 | 639 | MOBH | typeG | mandatory | 2.900e-44 | 143.900 | 0.873 | 0.271 |
| NZ_CP008860.2_5258436..5361673_00071 | 251 | G_tfc3 | typeG | accessory | 7.000e-97 | 315.900 | 0.988 | 0.996 |
| NZ_CP008860.2_5258436..5361673_00073 | 166 | G_tfc5 | typeG | accessory | 1.300e-56 | 183.800 | 0.971 | 0.994 |
| NZ_CP008860.2_5258436..5361673_00075 | 743 | t4cp2 | typeG | mandatory | 4.200e-31 | 100.700 | 0.955 | 0.318 |
| NZ_CP008860.2_5258436..5361673_00076 | 248 | G_tfc7 | typeG | accessory | 7.800e-104 | 339.400 | 1.000 | 1.000 |
| NZ_CP008860.2_5258436..5361673_00080 | 112 | G_tfc8 | typeG | accessory | 6.300e-41 | 131.700 | 0.922 | 0.929 |
| NZ_CP008860.2_5258436..5361673_00081 | 79 | G_tfc9 | typeG | accessory | 8.400e-31 | 99.000 | 1.000 | 1.000 |
| NZ_CP008860.2_5258436..5361673_00082 | 118 | G_tfc10 | typeG | accessory | 2.900e-44 | 142.200 | 0.875 | 0.890 |
| NZ_CP008860.2_5258436..5361673_00083 | 128 | G_tfc11 | typeG | accessory | 8.400e-50 | 160.500 | 0.923 | 0.938 |
| NZ_CP008860.2_5258436..5361673_00084 | 219 | G_tfc12 | typeG | accessory | 6.200e-91 | 296.200 | 0.941 | 0.959 |
| NZ_CP008860.2_5258436..5361673_00085 | 294 | G_tfc13 | typeG | accessory | 7.300e-119 | 389.000 | 0.944 | 0.959 |
| NZ_CP008860.2_5258436..5361673_00086 | 501 | G_tfc14 | typeG | accessory | 3.200e-184 | 606.200 | 0.996 | 0.992 |
| NZ_CP008860.2_5258436..5361673_00087 | 147 | G_tfc15 | typeG | accessory | 7.000e-60 | 193.900 | 0.957 | 0.925 |
| NZ_CP008860.2_5258436..5361673_00088 | 980 | virb4 | typeG | mandatory | 2.100e-89 | 293.700 | 0.807 | 0.899 |
| NZ_CP008860.2_5258436..5361673_00095 | 143 | G_tfc24 | typeG | accessory | 8.700e-51 | 164.300 | 0.914 | 0.895 |
| NZ_CP008860.2_5258436..5361673_00096 | 312 | G_tfc23 | typeG | accessory | 3.300e-134 | 439.900 | 0.973 | 0.987 |
| NZ_CP008860.2_5258436..5361673_00097 | 460 | G_tfc22 | typeG | accessory | 8.500e-180 | 591.000 | 0.987 | 0.974 |
| NZ_CP008860.2_5258436..5361673_00098 | 115 | G_tfc18 | typeG | accessory | 9.300e-29 | 92.400 | 0.912 | 0.887 |
| NZ_CP008860.2_5258436..5361673_00099 | 511 | G_tfc19 | typeG | accessory | 1.200e-222 | 732.700 | 0.990 | 0.982 |
| NZ_CP008860.2_5258436..5361673_00110 | 639 | MOBH | typeG | mandatory | 2.900e-44 | 143.900 | 0.873 | 0.271 |
| NZ_CP008861.1_5739208..5820841_00002 | 639 | MOBH | typeG | mandatory | 3.400e-44 | 143.400 | 0.868 | 0.268 |
| NZ_CP008861.1_5739208..5820841_00006 | 504 | G_tfc19 | typeG | accessory | 1.200e-222 | 732.500 | 0.984 | 0.990 |
| NZ_CP008861.1_5739208..5820841_00007 | 115 | G_tfc18 | typeG | accessory | 3.100e-29 | 93.700 | 0.912 | 0.887 |
| NZ_CP008861.1_5739208..5820841_00008 | 460 | G_tfc22 | typeG | accessory | 4.000e-180 | 591.800 | 0.987 | 0.974 |
| NZ_CP008861.1_5739208..5820841_00009 | 312 | G_tfc23 | typeG | accessory | 2.700e-134 | 440.000 | 0.973 | 0.987 |
| NZ_CP008861.1_5739208..5820841_00010 | 143 | G_tfc24 | typeG | accessory | 6.900e-51 | 164.400 | 0.907 | 0.888 |
| NZ_CP008861.1_5739208..5820841_00022 | 980 | virb4 | typeG | mandatory | 9.700e-91 | 297.900 | 0.806 | 0.898 |
| NZ_CP008861.1_5739208..5820841_00023 | 147 | G_tfc15 | typeG | accessory | 3.300e-60 | 194.700 | 0.957 | 0.925 |
| NZ_CP008861.1_5739208..5820841_00024 | 501 | G_tfc14 | typeG | accessory | 8.800e-185 | 607.800 | 0.996 | 0.992 |
| NZ_CP008861.1_5739208..5820841_00025 | 294 | G_tfc13 | typeG | accessory | 1.400e-118 | 387.800 | 0.955 | 0.956 |
| NZ_CP008861.1_5739208..5820841_00026 | 219 | G_tfc12 | typeG | accessory | 6.400e-92 | 299.200 | 0.950 | 0.968 |
| NZ_CP008861.1_5739208..5820841_00027 | 128 | G_tfc11 | typeG | accessory | 4.100e-50 | 161.300 | 0.923 | 0.938 |
| NZ_CP008861.1_5739208..5820841_00028 | 118 | G_tfc10 | typeG | accessory | 5.700e-44 | 141.000 | 0.875 | 0.890 |
| NZ_CP008861.1_5739208..5820841_00029 | 49 | G_tfc9 | typeG | accessory | 6.800e-18 | 57.400 | 0.620 | 1.000 |
| NZ_CP008861.1_5739208..5820841_00030 | 112 | G_tfc8 | typeG | accessory | 1.200e-40 | 130.600 | 0.914 | 0.920 |
| NZ_CP008861.1_5739208..5820841_00035 | 249 | G_tfc7 | typeG | accessory | 8.200e-104 | 339.100 | 1.000 | 0.996 |
| NZ_CP008861.1_5739208..5820841_00036 | 743 | t4cp2 | typeG | mandatory | 3.500e-31 | 100.700 | 0.955 | 0.318 |
| NZ_CP008861.1_5739208..5820841_00038 | 166 | G_tfc5 | typeG | accessory | 2.100e-57 | 186.100 | 0.971 | 0.994 |
| NZ_CP008861.1_5739208..5820841_00040 | 251 | G_tfc3 | typeG | accessory | 3.000e-96 | 313.600 | 0.988 | 0.996 |
| NZ_CP008862.2_1207510..1325095_00002 | 639 | MOBH | typeG | mandatory | 2.100e-44 | 144.500 | 0.887 | 0.275 |
| NZ_CP008862.2_1207510..1325095_00012 | 503 | G_tfc19 | typeG | accessory | 1.500e-222 | 732.600 | 0.986 | 0.994 |
| NZ_CP008862.2_1207510..1325095_00013 | 113 | G_tfc18 | typeG | accessory | 5.600e-28 | 90.100 | 0.912 | 0.885 |
| NZ_CP008862.2_1207510..1325095_00014 | 460 | G_tfc22 | typeG | accessory | 9.800e-180 | 591.000 | 0.987 | 0.974 |
| NZ_CP008862.2_1207510..1325095_00015 | 312 | G_tfc23 | typeG | accessory | 9.500e-134 | 438.600 | 0.973 | 0.987 |
| NZ_CP008862.2_1207510..1325095_00016 | 143 | G_tfc24 | typeG | accessory | 4.100e-51 | 165.500 | 0.907 | 0.888 |
| NZ_CP008862.2_1207510..1325095_00020 | 980 | virb4 | typeG | mandatory | 1.700e-89 | 294.200 | 0.807 | 0.899 |
| NZ_CP008862.2_1207510..1325095_00021 | 147 | G_tfc15 | typeG | accessory | 2.200e-59 | 192.500 | 0.957 | 0.925 |
| NZ_CP008862.2_1207510..1325095_00022 | 501 | G_tfc14 | typeG | accessory | 1.000e-184 | 608.000 | 0.996 | 0.992 |
| NZ_CP008862.2_1207510..1325095_00023 | 294 | G_tfc13 | typeG | accessory | 1.200e-118 | 388.500 | 0.955 | 0.956 |
| NZ_CP008862.2_1207510..1325095_00024 | 219 | G_tfc12 | typeG | accessory | 1.600e-91 | 298.400 | 0.950 | 0.968 |
| NZ_CP008862.2_1207510..1325095_00025 | 128 | G_tfc11 | typeG | accessory | 5.200e-50 | 161.400 | 0.923 | 0.938 |
| NZ_CP008862.2_1207510..1325095_00026 | 118 | G_tfc10 | typeG | accessory | 1.300e-44 | 143.600 | 0.883 | 0.890 |
| NZ_CP008862.2_1207510..1325095_00027 | 79 | G_tfc9 | typeG | accessory | 9.700e-31 | 99.000 | 1.000 | 1.000 |
| NZ_CP008862.2_1207510..1325095_00028 | 112 | G_tfc8 | typeG | accessory | 4.200e-41 | 132.500 | 0.922 | 0.929 |
| NZ_CP008862.2_1207510..1325095_00033 | 216 | G_tfc7 | typeG | accessory | 1.000e-95 | 313.000 | 0.871 | 1.000 |
| NZ_CP008862.2_1207510..1325095_00044 | 248 | G_tfc7 | typeG | accessory | 1.200e-103 | 339.000 | 1.000 | 1.000 |
| NZ_CP008862.2_1207510..1325095_00045 | 744 | t4cp2 | typeG | mandatory | 5.900e-31 | 100.400 | 0.955 | 0.319 |
| NZ_CP008862.2_1207510..1325095_00047 | 166 | G_tfc5 | typeG | accessory | 7.600e-56 | 181.500 | 0.977 | 1.000 |
| NZ_CP008862.2_1207510..1325095_00049 | 251 | G_tfc3 | typeG | accessory | 5.200e-95 | 310.000 | 0.967 | 0.976 |
| NZ_CP008863.1_4759452..4877038_00004 | 639 | MOBH | typeG | mandatory | 2.200e-44 | 144.500 | 0.887 | 0.275 |
| NZ_CP008863.1_4759452..4877038_00014 | 503 | G_tfc19 | typeG | accessory | 1.500e-222 | 732.600 | 0.986 | 0.994 |
| NZ_CP008863.1_4759452..4877038_00015 | 113 | G_tfc18 | typeG | accessory | 5.700e-28 | 90.100 | 0.912 | 0.885 |
| NZ_CP008863.1_4759452..4877038_00016 | 460 | G_tfc22 | typeG | accessory | 9.900e-180 | 591.000 | 0.987 | 0.974 |
| NZ_CP008863.1_4759452..4877038_00017 | 312 | G_tfc23 | typeG | accessory | 9.600e-134 | 438.600 | 0.973 | 0.987 |
| NZ_CP008863.1_4759452..4877038_00018 | 143 | G_tfc24 | typeG | accessory | 4.200e-51 | 165.500 | 0.907 | 0.888 |
| NZ_CP008863.1_4759452..4877038_00022 | 980 | virb4 | typeG | mandatory | 1.800e-89 | 294.200 | 0.807 | 0.899 |
| NZ_CP008863.1_4759452..4877038_00023 | 147 | G_tfc15 | typeG | accessory | 2.200e-59 | 192.500 | 0.957 | 0.925 |
| NZ_CP008863.1_4759452..4877038_00024 | 501 | G_tfc14 | typeG | accessory | 1.000e-184 | 608.000 | 0.996 | 0.992 |
| NZ_CP008863.1_4759452..4877038_00025 | 294 | G_tfc13 | typeG | accessory | 1.200e-118 | 388.500 | 0.955 | 0.956 |
| NZ_CP008863.1_4759452..4877038_00026 | 219 | G_tfc12 | typeG | accessory | 1.600e-91 | 298.400 | 0.950 | 0.968 |
| NZ_CP008863.1_4759452..4877038_00027 | 128 | G_tfc11 | typeG | accessory | 5.300e-50 | 161.400 | 0.923 | 0.938 |
| NZ_CP008863.1_4759452..4877038_00028 | 118 | G_tfc10 | typeG | accessory | 1.300e-44 | 143.600 | 0.883 | 0.890 |
| NZ_CP008863.1_4759452..4877038_00029 | 79 | G_tfc9 | typeG | accessory | 9.900e-31 | 99.000 | 1.000 | 1.000 |
| NZ_CP008863.1_4759452..4877038_00030 | 112 | G_tfc8 | typeG | accessory | 4.300e-41 | 132.500 | 0.922 | 0.929 |
| NZ_CP008863.1_4759452..4877038_00035 | 216 | G_tfc7 | typeG | accessory | 1.000e-95 | 313.000 | 0.871 | 1.000 |
| NZ_CP008863.1_4759452..4877038_00046 | 248 | G_tfc7 | typeG | accessory | 1.300e-103 | 339.000 | 1.000 | 1.000 |
| NZ_CP008863.1_4759452..4877038_00047 | 744 | t4cp2 | typeG | mandatory | 6.000e-31 | 100.400 | 0.955 | 0.319 |
| NZ_CP008863.1_4759452..4877038_00049 | 166 | G_tfc5 | typeG | accessory | 7.800e-56 | 181.500 | 0.977 | 1.000 |
| NZ_CP008863.1_4759452..4877038_00051 | 251 | G_tfc3 | typeG | accessory | 5.300e-95 | 310.000 | 0.967 | 0.976 |
| NZ_CP008865.2_4715709..4827366_00084 | 251 | G_tfc3 | typeG | accessory | 8.800e-96 | 312.500 | 0.967 | 0.976 |
| NZ_CP008865.2_4715709..4827366_00086 | 166 | G_tfc5 | typeG | accessory | 3.700e-57 | 185.700 | 0.971 | 0.994 |
| NZ_CP008865.2_4715709..4827366_00088 | 743 | t4cp2 | typeG | mandatory | 4.700e-31 | 100.700 | 0.955 | 0.318 |
| NZ_CP008865.2_4715709..4827366_00089 | 248 | G_tfc7 | typeG | accessory | 4.800e-103 | 337.000 | 1.000 | 1.000 |
| NZ_CP008865.2_4715709..4827366_00094 | 112 | G_tfc8 | typeG | accessory | 2.000e-41 | 133.500 | 0.931 | 0.938 |
| NZ_CP008865.2_4715709..4827366_00095 | 79 | G_tfc9 | typeG | accessory | 1.400e-30 | 98.500 | 1.000 | 1.000 |
| NZ_CP008865.2_4715709..4827366_00096 | 118 | G_tfc10 | typeG | accessory | 7.700e-44 | 141.000 | 0.875 | 0.890 |
| NZ_CP008865.2_4715709..4827366_00097 | 128 | G_tfc11 | typeG | accessory | 5.500e-50 | 161.300 | 0.923 | 0.938 |
| NZ_CP008865.2_4715709..4827366_00098 | 219 | G_tfc12 | typeG | accessory | 8.600e-92 | 299.200 | 0.950 | 0.968 |
| NZ_CP008865.2_4715709..4827366_00099 | 294 | G_tfc13 | typeG | accessory | 4.200e-118 | 386.700 | 0.955 | 0.956 |
| NZ_CP008865.2_4715709..4827366_00100 | 501 | G_tfc14 | typeG | accessory | 1.100e-183 | 604.600 | 0.996 | 0.992 |
| NZ_CP008865.2_4715709..4827366_00101 | 147 | G_tfc15 | typeG | accessory | 4.400e-60 | 194.700 | 0.957 | 0.925 |
| NZ_CP008865.2_4715709..4827366_00102 | 980 | virb4 | typeG | mandatory | 1.300e-90 | 297.900 | 0.806 | 0.898 |
| NZ_CP008865.2_4715709..4827366_00111 | 143 | G_tfc24 | typeG | accessory | 9.300e-51 | 164.400 | 0.907 | 0.888 |
| NZ_CP008865.2_4715709..4827366_00112 | 312 | G_tfc23 | typeG | accessory | 9.200e-134 | 438.600 | 0.973 | 0.987 |
| NZ_CP008865.2_4715709..4827366_00113 | 462 | G_tfc22 | typeG | accessory | 5.400e-180 | 591.800 | 0.987 | 0.970 |
| NZ_CP008865.2_4715709..4827366_00114 | 115 | G_tfc18 | typeG | accessory | 3.900e-29 | 93.800 | 0.912 | 0.887 |
| NZ_CP008865.2_4715709..4827366_00115 | 511 | G_tfc19 | typeG | accessory | 7.000e-223 | 733.600 | 0.990 | 0.982 |
| NZ_CP008865.2_4715709..4827366_00124 | 639 | MOBH | typeG | mandatory | 4.700e-44 | 143.400 | 0.868 | 0.268 |
| NZ_CP008866.2_5456109..5543956_00077 | 251 | G_tfc3 | typeG | accessory | 3.700e-96 | 313.600 | 0.988 | 0.996 |
| NZ_CP008866.2_5456109..5543956_00079 | 166 | G_tfc5 | typeG | accessory | 2.600e-57 | 186.100 | 0.971 | 0.994 |
| NZ_CP008866.2_5456109..5543956_00081 | 743 | t4cp2 | typeG | mandatory | 4.400e-31 | 100.700 | 0.955 | 0.318 |
| NZ_CP008866.2_5456109..5543956_00082 | 249 | G_tfc7 | typeG | accessory | 1.000e-103 | 339.100 | 1.000 | 0.996 |
| NZ_CP008866.2_5456109..5543956_00088 | 112 | G_tfc8 | typeG | accessory | 1.400e-40 | 130.600 | 0.914 | 0.920 |
| NZ_CP008866.2_5456109..5543956_00089 | 79 | G_tfc9 | typeG | accessory | 1.100e-30 | 98.700 | 1.000 | 1.000 |
| NZ_CP008866.2_5456109..5543956_00090 | 118 | G_tfc10 | typeG | accessory | 7.100e-44 | 141.000 | 0.875 | 0.890 |
| NZ_CP008866.2_5456109..5543956_00091 | 128 | G_tfc11 | typeG | accessory | 5.100e-50 | 161.300 | 0.923 | 0.938 |
| NZ_CP008866.2_5456109..5543956_00092 | 219 | G_tfc12 | typeG | accessory | 7.900e-92 | 299.200 | 0.950 | 0.968 |
| NZ_CP008866.2_5456109..5543956_00093 | 294 | G_tfc13 | typeG | accessory | 5.500e-119 | 389.400 | 0.955 | 0.956 |
| NZ_CP008866.2_5456109..5543956_00094 | 501 | G_tfc14 | typeG | accessory | 1.600e-184 | 607.300 | 0.990 | 0.984 |
| NZ_CP008866.2_5456109..5543956_00095 | 147 | G_tfc15 | typeG | accessory | 1.100e-60 | 196.600 | 0.957 | 0.925 |
| NZ_CP008866.2_5456109..5543956_00096 | 980 | virb4 | typeG | mandatory | 1.700e-90 | 297.400 | 0.807 | 0.899 |
| NZ_CP008866.2_5456109..5543956_00102 | 143 | G_tfc24 | typeG | accessory | 5.900e-51 | 164.900 | 0.914 | 0.895 |
| NZ_CP008866.2_5456109..5543956_00103 | 312 | G_tfc23 | typeG | accessory | 2.400e-134 | 440.400 | 0.973 | 0.987 |
| NZ_CP008866.2_5456109..5543956_00104 | 460 | G_tfc22 | typeG | accessory | 5.700e-180 | 591.600 | 0.987 | 0.974 |
| NZ_CP008866.2_5456109..5543956_00105 | 115 | G_tfc18 | typeG | accessory | 4.000e-29 | 93.600 | 0.912 | 0.887 |
| NZ_CP008866.2_5456109..5543956_00106 | 503 | G_tfc19 | typeG | accessory | 7.400e-222 | 730.100 | 0.984 | 0.992 |
| NZ_CP008866.2_5456109..5543956_00114 | 639 | MOBH | typeG | mandatory | 3.000e-44 | 143.900 | 0.868 | 0.268 |
| NZ_CP008869.2_5440677..5533836_00074 | 251 | G_tfc3 | typeG | accessory | 3.600e-95 | 310.300 | 0.967 | 0.976 |
| NZ_CP008869.2_5440677..5533836_00076 | 166 | G_tfc5 | typeG | accessory | 2.200e-57 | 186.200 | 0.971 | 0.994 |
| NZ_CP008869.2_5440677..5533836_00078 | 743 | t4cp2 | typeG | mandatory | 4.000e-31 | 100.700 | 0.955 | 0.318 |
| NZ_CP008869.2_5440677..5533836_00079 | 249 | G_tfc7 | typeG | accessory | 9.200e-104 | 339.100 | 1.000 | 0.996 |
| NZ_CP008869.2_5440677..5533836_00084 | 112 | G_tfc8 | typeG | accessory | 1.700e-41 | 133.500 | 0.931 | 0.938 |
| NZ_CP008869.2_5440677..5533836_00085 | 79 | G_tfc9 | typeG | accessory | 1.200e-30 | 98.500 | 1.000 | 1.000 |
| NZ_CP008869.2_5440677..5533836_00086 | 118 | G_tfc10 | typeG | accessory | 3.900e-44 | 141.700 | 0.925 | 0.924 |
| NZ_CP008869.2_5440677..5533836_00087 | 128 | G_tfc11 | typeG | accessory | 6.600e-50 | 160.800 | 0.923 | 0.938 |
| NZ_CP008869.2_5440677..5533836_00088 | 219 | G_tfc12 | typeG | accessory | 7.500e-91 | 295.900 | 0.941 | 0.959 |
| NZ_CP008869.2_5440677..5533836_00089 | 294 | G_tfc13 | typeG | accessory | 3.400e-118 | 386.700 | 0.944 | 0.959 |
| NZ_CP008869.2_5440677..5533836_00090 | 501 | G_tfc14 | typeG | accessory | 7.900e-184 | 604.800 | 0.996 | 0.992 |
| NZ_CP008869.2_5440677..5533836_00091 | 147 | G_tfc15 | typeG | accessory | 3.600e-60 | 194.700 | 0.957 | 0.925 |
| NZ_CP008869.2_5440677..5533836_00092 | 980 | virb4 | typeG | mandatory | 8.100e-90 | 295.000 | 0.807 | 0.899 |
| NZ_CP008869.2_5440677..5533836_00096 | 143 | G_tfc24 | typeG | accessory | 5.000e-51 | 165.000 | 0.914 | 0.895 |
| NZ_CP008869.2_5440677..5533836_00097 | 312 | G_tfc23 | typeG | accessory | 4.700e-133 | 436.100 | 0.973 | 0.987 |
| NZ_CP008869.2_5440677..5533836_00098 | 462 | G_tfc22 | typeG | accessory | 2.300e-179 | 589.400 | 0.987 | 0.970 |
| NZ_CP008869.2_5440677..5533836_00099 | 115 | G_tfc18 | typeG | accessory | 3.300e-29 | 93.800 | 0.912 | 0.887 |
| NZ_CP008869.2_5440677..5533836_00100 | 503 | G_tfc19 | typeG | accessory | 2.400e-222 | 731.600 | 0.984 | 0.992 |
| NZ_CP008869.2_5440677..5533836_00103 | 629 | MOBH | typeG | mandatory | 2.600e-44 | 143.900 | 0.868 | 0.272 |
| NZ_CP008871.2_4687981..4791112_00083 | 251 | G_tfc3 | typeG | accessory | 8.400e-96 | 312.500 | 0.967 | 0.976 |
| NZ_CP008871.2_4687981..4791112_00085 | 166 | G_tfc5 | typeG | accessory | 3.600e-57 | 185.700 | 0.971 | 0.994 |
| NZ_CP008871.2_4687981..4791112_00087 | 743 | t4cp2 | typeG | mandatory | 4.600e-31 | 100.700 | 0.955 | 0.318 |
| NZ_CP008871.2_4687981..4791112_00088 | 248 | G_tfc7 | typeG | accessory | 4.600e-103 | 337.000 | 1.000 | 1.000 |
| NZ_CP008871.2_4687981..4791112_00093 | 112 | G_tfc8 | typeG | accessory | 1.900e-41 | 133.500 | 0.931 | 0.938 |
| NZ_CP008871.2_4687981..4791112_00094 | 79 | G_tfc9 | typeG | accessory | 1.400e-30 | 98.500 | 1.000 | 1.000 |
| NZ_CP008871.2_4687981..4791112_00095 | 118 | G_tfc10 | typeG | accessory | 7.400e-44 | 141.000 | 0.875 | 0.890 |
| NZ_CP008871.2_4687981..4791112_00096 | 128 | G_tfc11 | typeG | accessory | 5.300e-50 | 161.300 | 0.923 | 0.938 |
| NZ_CP008871.2_4687981..4791112_00097 | 219 | G_tfc12 | typeG | accessory | 8.300e-92 | 299.200 | 0.950 | 0.968 |
| NZ_CP008871.2_4687981..4791112_00098 | 294 | G_tfc13 | typeG | accessory | 4.000e-118 | 386.700 | 0.955 | 0.956 |
| NZ_CP008871.2_4687981..4791112_00099 | 501 | G_tfc14 | typeG | accessory | 1.000e-183 | 604.600 | 0.996 | 0.992 |
| NZ_CP008871.2_4687981..4791112_00100 | 147 | G_tfc15 | typeG | accessory | 4.200e-60 | 194.700 | 0.957 | 0.925 |
| NZ_CP008871.2_4687981..4791112_00101 | 980 | virb4 | typeG | mandatory | 1.300e-90 | 297.900 | 0.806 | 0.898 |
| NZ_CP008871.2_4687981..4791112_00106 | 143 | G_tfc24 | typeG | accessory | 8.900e-51 | 164.400 | 0.907 | 0.888 |
| NZ_CP008871.2_4687981..4791112_00107 | 312 | G_tfc23 | typeG | accessory | 8.900e-134 | 438.600 | 0.973 | 0.987 |
| NZ_CP008871.2_4687981..4791112_00108 | 462 | G_tfc22 | typeG | accessory | 5.200e-180 | 591.800 | 0.987 | 0.970 |
| NZ_CP008871.2_4687981..4791112_00109 | 115 | G_tfc18 | typeG | accessory | 3.800e-29 | 93.800 | 0.912 | 0.887 |
| NZ_CP008871.2_4687981..4791112_00110 | 511 | G_tfc19 | typeG | accessory | 6.700e-223 | 733.600 | 0.990 | 0.982 |
| NZ_CP008871.2_4687981..4791112_00119 | 639 | MOBH | typeG | mandatory | 4.500e-44 | 143.400 | 0.868 | 0.268 |
| NZ_CP008873.1_2635482..2723321_00002 | 646 | MOBH | typeG | mandatory | 3.500e-44 | 143.700 | 0.868 | 0.265 |
| NZ_CP008873.1_2635482..2723321_00010 | 503 | G_tfc19 | typeG | accessory | 7.500e-222 | 730.100 | 0.984 | 0.992 |
| NZ_CP008873.1_2635482..2723321_00011 | 115 | G_tfc18 | typeG | accessory | 4.000e-29 | 93.600 | 0.912 | 0.887 |
| NZ_CP008873.1_2635482..2723321_00012 | 460 | G_tfc22 | typeG | accessory | 5.700e-180 | 591.600 | 0.987 | 0.974 |
| NZ_CP008873.1_2635482..2723321_00013 | 312 | G_tfc23 | typeG | accessory | 2.500e-134 | 440.400 | 0.973 | 0.987 |
| NZ_CP008873.1_2635482..2723321_00014 | 143 | G_tfc24 | typeG | accessory | 6.000e-51 | 164.900 | 0.914 | 0.895 |
| NZ_CP008873.1_2635482..2723321_00021 | 920 | virb4 | typeG | mandatory | 4.500e-87 | 286.100 | 0.784 | 0.934 |
| NZ_CP008873.1_2635482..2723321_00022 | 147 | G_tfc15 | typeG | accessory | 1.100e-60 | 196.600 | 0.957 | 0.925 |
| NZ_CP008873.1_2635482..2723321_00023 | 501 | G_tfc14 | typeG | accessory | 1.600e-184 | 607.300 | 0.990 | 0.984 |
| NZ_CP008873.1_2635482..2723321_00024 | 512 | G_tfc12 | typeG | accessory | 2.000e-90 | 294.600 | 0.946 | 0.412 |
| NZ_CP008873.1_2635482..2723321_00025 | 128 | G_tfc11 | typeG | accessory | 5.100e-50 | 161.300 | 0.923 | 0.938 |
| NZ_CP008873.1_2635482..2723321_00026 | 73 | G_tfc10 | typeG | accessory | 1.300e-31 | 101.400 | 0.608 | 1.000 |
| NZ_CP008873.1_2635482..2723321_00027 | 79 | G_tfc9 | typeG | accessory | 1.100e-30 | 98.700 | 1.000 | 1.000 |
| NZ_CP008873.1_2635482..2723321_00028 | 112 | G_tfc8 | typeG | accessory | 1.400e-40 | 130.600 | 0.914 | 0.920 |
| NZ_CP008873.1_2635482..2723321_00034 | 249 | G_tfc7 | typeG | accessory | 1.000e-103 | 339.100 | 1.000 | 0.996 |
| NZ_CP008873.1_2635482..2723321_00035 | 743 | t4cp2 | typeG | mandatory | 4.400e-31 | 100.700 | 0.955 | 0.318 |
| NZ_CP008873.1_2635482..2723321_00037 | 166 | G_tfc5 | typeG | accessory | 2.600e-57 | 186.100 | 0.971 | 0.994 |
| NZ_CP008873.1_2635482..2723321_00039 | 251 | G_tfc3 | typeG | accessory | 3.700e-96 | 313.600 | 0.988 | 0.996 |
| NZ_CP010555.1_5486878..5572127_00002 | 639 | MOBH | typeG | mandatory | 4.000e-44 | 143.400 | 0.868 | 0.268 |
| NZ_CP010555.1_5486878..5572127_00006 | 504 | G_tfc19 | typeG | accessory | 1.300e-222 | 732.500 | 0.984 | 0.990 |
| NZ_CP010555.1_5486878..5572127_00007 | 115 | G_tfc18 | typeG | accessory | 3.600e-29 | 93.700 | 0.912 | 0.887 |
| NZ_CP010555.1_5486878..5572127_00008 | 460 | G_tfc22 | typeG | accessory | 6.600e-180 | 591.300 | 0.987 | 0.974 |
| NZ_CP010555.1_5486878..5572127_00009 | 312 | G_tfc23 | typeG | accessory | 8.700e-134 | 438.500 | 0.976 | 0.990 |
| NZ_CP010555.1_5486878..5572127_00010 | 143 | G_tfc24 | typeG | accessory | 1.300e-51 | 166.900 | 0.914 | 0.895 |
| NZ_CP010555.1_5486878..5572127_00015 | 980 | virb4 | typeG | mandatory | 1.400e-90 | 297.600 | 0.810 | 0.902 |
| NZ_CP010555.1_5486878..5572127_00016 | 147 | G_tfc15 | typeG | accessory | 2.200e-60 | 195.500 | 0.957 | 0.925 |
| NZ_CP010555.1_5486878..5572127_00017 | 501 | G_tfc14 | typeG | accessory | 6.900e-185 | 608.400 | 0.996 | 0.992 |
| NZ_CP010555.1_5486878..5572127_00018 | 294 | G_tfc13 | typeG | accessory | 1.700e-117 | 384.500 | 0.955 | 0.956 |
| NZ_CP010555.1_5486878..5572127_00019 | 219 | G_tfc12 | typeG | accessory | 4.600e-91 | 296.600 | 0.941 | 0.959 |
| NZ_CP010555.1_5486878..5572127_00020 | 128 | G_tfc11 | typeG | accessory | 6.100e-50 | 160.900 | 0.923 | 0.938 |
| NZ_CP010555.1_5486878..5572127_00021 | 118 | G_tfc10 | typeG | accessory | 6.700e-44 | 141.000 | 0.875 | 0.890 |
| NZ_CP010555.1_5486878..5572127_00022 | 79 | G_tfc9 | typeG | accessory | 1.100e-30 | 98.700 | 1.000 | 1.000 |
| NZ_CP010555.1_5486878..5572127_00023 | 112 | G_tfc8 | typeG | accessory | 1.400e-40 | 130.600 | 0.914 | 0.920 |
| NZ_CP010555.1_5486878..5572127_00029 | 249 | G_tfc7 | typeG | accessory | 9.600e-104 | 339.100 | 1.000 | 0.996 |
| NZ_CP010555.1_5486878..5572127_00030 | 743 | t4cp2 | typeG | mandatory | 4.100e-31 | 100.700 | 0.955 | 0.318 |
| NZ_CP010555.1_5486878..5572127_00032 | 166 | G_tfc5 | typeG | accessory | 2.500e-57 | 186.100 | 0.971 | 0.994 |
| NZ_CP010555.1_5486878..5572127_00034 | 251 | G_tfc3 | typeG | accessory | 3.500e-96 | 313.600 | 0.988 | 0.996 |
| NZ_CP011317.1_6087900..6172252_00034 | 374 | G_tfc2 | typeG | accessory | 1.200e-71 | 232.900 | 0.782 | 0.631 |
| NZ_CP011317.1_6087900..6172252_00064 | 251 | G_tfc3 | typeG | accessory | 6.400e-96 | 312.600 | 0.967 | 0.976 |
| NZ_CP011317.1_6087900..6172252_00066 | 166 | G_tfc5 | typeG | accessory | 2.200e-57 | 186.100 | 0.971 | 0.994 |
| NZ_CP011317.1_6087900..6172252_00068 | 743 | t4cp2 | typeG | mandatory | 3.600e-31 | 100.700 | 0.955 | 0.318 |
| NZ_CP011317.1_6087900..6172252_00069 | 249 | G_tfc7 | typeG | accessory | 8.400e-104 | 339.100 | 1.000 | 0.996 |
| NZ_CP011317.1_6087900..6172252_00073 | 112 | G_tfc8 | typeG | accessory | 7.100e-41 | 131.300 | 0.922 | 0.929 |
| NZ_CP011317.1_6087900..6172252_00074 | 79 | G_tfc9 | typeG | accessory | 7.200e-31 | 99.000 | 1.000 | 1.000 |
| NZ_CP011317.1_6087900..6172252_00075 | 118 | G_tfc10 | typeG | accessory | 3.300e-45 | 145.100 | 0.925 | 0.941 |
| NZ_CP011317.1_6087900..6172252_00076 | 128 | G_tfc11 | typeG | accessory | 1.000e-49 | 160.000 | 0.923 | 0.938 |
| NZ_CP011317.1_6087900..6172252_00077 | 219 | G_tfc12 | typeG | accessory | 6.800e-91 | 295.900 | 0.941 | 0.959 |
| NZ_CP011317.1_6087900..6172252_00078 | 294 | G_tfc13 | typeG | accessory | 5.700e-119 | 389.100 | 0.944 | 0.959 |
| NZ_CP011317.1_6087900..6172252_00079 | 501 | G_tfc14 | typeG | accessory | 1.300e-184 | 607.200 | 0.996 | 0.992 |
| NZ_CP011317.1_6087900..6172252_00080 | 147 | G_tfc15 | typeG | accessory | 5.100e-60 | 194.100 | 0.957 | 0.925 |
| NZ_CP011317.1_6087900..6172252_00081 | 983 | virb4 | typeG | mandatory | 2.400e-90 | 296.600 | 0.810 | 0.899 |
| NZ_CP011317.1_6087900..6172252_00086 | 143 | G_tfc24 | typeG | accessory | 8.200e-51 | 164.100 | 0.907 | 0.888 |
| NZ_CP011317.1_6087900..6172252_00087 | 312 | G_tfc23 | typeG | accessory | 3.300e-134 | 439.700 | 0.973 | 0.987 |
| NZ_CP011317.1_6087900..6172252_00088 | 463 | G_tfc22 | typeG | accessory | 1.200e-180 | 593.500 | 0.989 | 0.970 |
| NZ_CP011317.1_6087900..6172252_00089 | 121 | G_tfc18 | typeG | accessory | 1.100e-27 | 88.700 | 0.930 | 0.860 |
| NZ_CP011317.1_6087900..6172252_00090 | 510 | G_tfc19 | typeG | accessory | 2.800e-221 | 727.900 | 0.994 | 0.988 |
| NZ_CP011317.1_6087900..6172252_00094 | 640 | MOBH | typeG | mandatory | 2.400e-44 | 143.900 | 0.868 | 0.267 |
| NZ_CP011857.1_5504320..5607155_00073 | 251 | G_tfc3 | typeG | accessory | 4.100e-96 | 313.600 | 0.988 | 0.996 |
| NZ_CP011857.1_5504320..5607155_00075 | 166 | G_tfc5 | typeG | accessory | 2.900e-57 | 186.100 | 0.971 | 0.994 |
| NZ_CP011857.1_5504320..5607155_00077 | 743 | t4cp2 | typeG | mandatory | 4.900e-31 | 100.700 | 0.955 | 0.318 |
| NZ_CP011857.1_5504320..5607155_00078 | 249 | G_tfc7 | typeG | accessory | 1.100e-103 | 339.100 | 1.000 | 0.996 |
| NZ_CP011857.1_5504320..5607155_00103 | 112 | G_tfc8 | typeG | accessory | 7.600e-41 | 131.700 | 0.914 | 0.920 |
| NZ_CP011857.1_5504320..5607155_00104 | 79 | G_tfc9 | typeG | accessory | 1.400e-30 | 98.500 | 1.000 | 1.000 |
| NZ_CP011857.1_5504320..5607155_00105 | 118 | G_tfc10 | typeG | accessory | 7.900e-44 | 141.000 | 0.875 | 0.890 |
| NZ_CP011857.1_5504320..5607155_00106 | 128 | G_tfc11 | typeG | accessory | 5.600e-50 | 161.300 | 0.923 | 0.938 |
| NZ_CP011857.1_5504320..5607155_00107 | 219 | G_tfc12 | typeG | accessory | 8.800e-92 | 299.200 | 0.950 | 0.968 |
| NZ_CP011857.1_5504320..5607155_00108 | 294 | G_tfc13 | typeG | accessory | 4.300e-118 | 386.700 | 0.955 | 0.956 |
| NZ_CP011857.1_5504320..5607155_00109 | 501 | G_tfc14 | typeG | accessory | 1.100e-183 | 604.600 | 0.996 | 0.992 |
| NZ_CP011857.1_5504320..5607155_00110 | 147 | G_tfc15 | typeG | accessory | 4.500e-60 | 194.700 | 0.957 | 0.925 |
| NZ_CP011857.1_5504320..5607155_00111 | 980 | virb4 | typeG | mandatory | 1.300e-90 | 297.900 | 0.806 | 0.898 |
| NZ_CP011857.1_5504320..5607155_00116 | 143 | G_tfc24 | typeG | accessory | 1.400e-49 | 160.600 | 0.907 | 0.888 |
| NZ_CP011857.1_5504320..5607155_00117 | 312 | G_tfc23 | typeG | accessory | 3.700e-134 | 440.000 | 0.973 | 0.987 |
| NZ_CP011857.1_5504320..5607155_00118 | 460 | G_tfc22 | typeG | accessory | 5.500e-180 | 591.800 | 0.987 | 0.974 |
| NZ_CP011857.1_5504320..5607155_00119 | 115 | G_tfc18 | typeG | accessory | 4.300e-29 | 93.700 | 0.912 | 0.887 |
| NZ_CP011857.1_5504320..5607155_00120 | 504 | G_tfc19 | typeG | accessory | 1.600e-222 | 732.500 | 0.984 | 0.990 |
| NZ_CP011857.1_5504320..5607155_00127 | 639 | MOBH | typeG | mandatory | 4.600e-44 | 143.400 | 0.868 | 0.268 |
| NZ_CP012679.1_4518241..4621031_00071 | 251 | G_tfc3 | typeG | accessory | 1.700e-94 | 308.100 | 0.988 | 0.996 |
| NZ_CP012679.1_4518241..4621031_00073 | 166 | G_tfc5 | typeG | accessory | 2.500e-57 | 186.100 | 0.971 | 0.994 |
| NZ_CP012679.1_4518241..4621031_00075 | 743 | t4cp2 | typeG | mandatory | 1.900e-31 | 101.800 | 0.955 | 0.318 |
| NZ_CP012679.1_4518241..4621031_00076 | 248 | G_tfc7 | typeG | accessory | 8.800e-104 | 339.200 | 1.000 | 1.000 |
| NZ_CP012679.1_4518241..4621031_00087 | 112 | G_tfc8 | typeG | accessory | 1.200e-40 | 130.800 | 0.922 | 0.929 |
| NZ_CP012679.1_4518241..4621031_00088 | 79 | G_tfc9 | typeG | accessory | 3.200e-31 | 100.400 | 1.000 | 1.000 |
| NZ_CP012679.1_4518241..4621031_00089 | 118 | G_tfc10 | typeG | accessory | 4.000e-44 | 141.700 | 0.925 | 0.924 |
| NZ_CP012679.1_4518241..4621031_00090 | 128 | G_tfc11 | typeG | accessory | 3.500e-49 | 158.500 | 0.923 | 0.938 |
| NZ_CP012679.1_4518241..4621031_00091 | 219 | G_tfc12 | typeG | accessory | 3.000e-91 | 297.200 | 0.941 | 0.959 |
| NZ_CP012679.1_4518241..4621031_00092 | 294 | G_tfc13 | typeG | accessory | 5.400e-118 | 386.100 | 0.955 | 0.956 |
| NZ_CP012679.1_4518241..4621031_00093 | 501 | G_tfc14 | typeG | accessory | 7.200e-185 | 608.300 | 0.996 | 0.992 |
| NZ_CP012679.1_4518241..4621031_00094 | 147 | G_tfc15 | typeG | accessory | 7.300e-60 | 193.800 | 0.957 | 0.925 |
| NZ_CP012679.1_4518241..4621031_00095 | 980 | virb4 | typeG | mandatory | 1.900e-89 | 293.900 | 0.805 | 0.897 |
| NZ_CP012679.1_4518241..4621031_00099 | 143 | G_tfc24 | typeG | accessory | 8.600e-51 | 164.300 | 0.914 | 0.895 |
| NZ_CP012679.1_4518241..4621031_00100 | 312 | G_tfc23 | typeG | accessory | 7.500e-133 | 435.500 | 0.973 | 0.987 |
| NZ_CP012679.1_4518241..4621031_00101 | 462 | G_tfc22 | typeG | accessory | 1.700e-179 | 590.000 | 0.987 | 0.970 |
| NZ_CP012679.1_4518241..4621031_00102 | 115 | G_tfc18 | typeG | accessory | 3.400e-29 | 93.800 | 0.912 | 0.887 |
| NZ_CP012679.1_4518241..4621031_00103 | 504 | G_tfc19 | typeG | accessory | 1.500e-222 | 732.400 | 0.984 | 0.990 |
| NZ_CP012679.1_4518241..4621031_00108 | 639 | MOBH | typeG | mandatory | 2.800e-44 | 143.900 | 0.868 | 0.268 |
| NZ_CP013245.1_3408411..3521196_00002 | 608 | MOBH | typeG | mandatory | 1.700e-43 | 141.400 | 0.843 | 0.270 |
| NZ_CP013245.1_3408411..3521196_00014 | 503 | G_tfc19 | typeG | accessory | 2.500e-222 | 731.700 | 0.986 | 0.994 |
| NZ_CP013245.1_3408411..3521196_00015 | 115 | G_tfc18 | typeG | accessory | 6.000e-29 | 93.100 | 0.912 | 0.887 |
| NZ_CP013245.1_3408411..3521196_00016 | 460 | G_tfc22 | typeG | accessory | 9.000e-180 | 591.000 | 0.987 | 0.974 |
| NZ_CP013245.1_3408411..3521196_00017 | 312 | G_tfc23 | typeG | accessory | 1.000e-133 | 438.400 | 0.973 | 0.987 |
| NZ_CP013245.1_3408411..3521196_00030 | 980 | virb4 | typeG | mandatory | 1.100e-89 | 294.700 | 0.807 | 0.899 |
| NZ_CP013245.1_3408411..3521196_00031 | 147 | G_tfc15 | typeG | accessory | 1.500e-59 | 192.900 | 0.957 | 0.925 |
| NZ_CP013245.1_3408411..3521196_00032 | 501 | G_tfc14 | typeG | accessory | 6.000e-185 | 608.700 | 0.996 | 0.992 |
| NZ_CP013245.1_3408411..3521196_00033 | 293 | G_tfc13 | typeG | accessory | 4.200e-118 | 386.600 | 0.955 | 0.956 |
| NZ_CP013245.1_3408411..3521196_00034 | 219 | G_tfc12 | typeG | accessory | 1.500e-91 | 298.400 | 0.950 | 0.968 |
| NZ_CP013245.1_3408411..3521196_00035 | 128 | G_tfc11 | typeG | accessory | 7.500e-50 | 160.800 | 0.923 | 0.938 |
| NZ_CP013245.1_3408411..3521196_00036 | 118 | G_tfc10 | typeG | accessory | 1.400e-44 | 143.300 | 0.883 | 0.890 |
| NZ_CP013245.1_3408411..3521196_00037 | 79 | G_tfc9 | typeG | accessory | 2.500e-30 | 97.600 | 1.000 | 1.000 |
| NZ_CP013245.1_3408411..3521196_00038 | 112 | G_tfc8 | typeG | accessory | 1.500e-40 | 130.600 | 0.922 | 0.929 |
| NZ_CP013245.1_3408411..3521196_00045 | 248 | G_tfc7 | typeG | accessory | 1.000e-103 | 339.100 | 1.000 | 1.000 |
| NZ_CP013245.1_3408411..3521196_00046 | 743 | t4cp2 | typeG | mandatory | 4.700e-31 | 100.600 | 0.955 | 0.318 |
| NZ_CP013245.1_3408411..3521196_00048 | 166 | G_tfc5 | typeG | accessory | 1.500e-56 | 183.700 | 0.977 | 1.000 |
| NZ_CP013245.1_3408411..3521196_00050 | 251 | G_tfc3 | typeG | accessory | 1.100e-94 | 308.800 | 0.967 | 0.976 |
| NZ_CP013993.1_5007879..5102356_00077 | 251 | G_tfc3 | typeG | accessory | 3.900e-96 | 313.600 | 0.988 | 0.996 |
| NZ_CP013993.1_5007879..5102356_00079 | 166 | G_tfc5 | typeG | accessory | 2.800e-57 | 186.100 | 0.971 | 0.994 |
| NZ_CP013993.1_5007879..5102356_00081 | 743 | t4cp2 | typeG | mandatory | 4.700e-31 | 100.700 | 0.955 | 0.318 |
| NZ_CP013993.1_5007879..5102356_00082 | 249 | G_tfc7 | typeG | accessory | 1.100e-103 | 339.100 | 1.000 | 0.996 |
| NZ_CP013993.1_5007879..5102356_00096 | 112 | G_tfc8 | typeG | accessory | 1.500e-40 | 130.600 | 0.914 | 0.920 |
| NZ_CP013993.1_5007879..5102356_00097 | 79 | G_tfc9 | typeG | accessory | 1.200e-30 | 98.700 | 1.000 | 1.000 |
| NZ_CP013993.1_5007879..5102356_00098 | 118 | G_tfc10 | typeG | accessory | 7.600e-44 | 141.000 | 0.875 | 0.890 |
| NZ_CP013993.1_5007879..5102356_00099 | 128 | G_tfc11 | typeG | accessory | 5.400e-50 | 161.300 | 0.923 | 0.938 |
| NZ_CP013993.1_5007879..5102356_00100 | 219 | G_tfc12 | typeG | accessory | 8.500e-92 | 299.200 | 0.950 | 0.968 |
| NZ_CP013993.1_5007879..5102356_00101 | 294 | G_tfc13 | typeG | accessory | 1.800e-118 | 387.800 | 0.955 | 0.956 |
| NZ_CP013993.1_5007879..5102356_00102 | 501 | G_tfc14 | typeG | accessory | 1.200e-184 | 607.800 | 0.996 | 0.992 |
| NZ_CP013993.1_5007879..5102356_00103 | 147 | G_tfc15 | typeG | accessory | 4.300e-60 | 194.700 | 0.957 | 0.925 |
| NZ_CP013993.1_5007879..5102356_00104 | 980 | virb4 | typeG | mandatory | 4.300e-90 | 296.100 | 0.806 | 0.898 |
| NZ_CP013993.1_5007879..5102356_00110 | 143 | G_tfc24 | typeG | accessory | 9.100e-51 | 164.400 | 0.907 | 0.888 |
| NZ_CP013993.1_5007879..5102356_00111 | 312 | G_tfc23 | typeG | accessory | 3.600e-134 | 440.000 | 0.973 | 0.987 |
| NZ_CP013993.1_5007879..5102356_00112 | 460 | G_tfc22 | typeG | accessory | 5.300e-180 | 591.800 | 0.987 | 0.974 |
| NZ_CP013993.1_5007879..5102356_00113 | 115 | G_tfc18 | typeG | accessory | 4.100e-29 | 93.700 | 0.912 | 0.887 |
| NZ_CP013993.1_5007879..5102356_00114 | 504 | G_tfc19 | typeG | accessory | 1.500e-222 | 732.500 | 0.984 | 0.990 |
| NZ_CP013993.1_5007879..5102356_00122 | 639 | MOBH | typeG | mandatory | 4.500e-44 | 143.400 | 0.868 | 0.268 |
| NZ_CP014948.1_4463713..4568937_00028 | 374 | G_tfc2 | typeG | accessory | 2.000e-71 | 232.500 | 0.782 | 0.570 |
| NZ_CP014948.1_4463713..4568937_00058 | 251 | G_tfc3 | typeG | accessory | 3.900e-95 | 310.300 | 0.988 | 0.996 |
| NZ_CP014948.1_4463713..4568937_00060 | 166 | G_tfc5 | typeG | accessory | 4.400e-57 | 185.400 | 0.971 | 0.994 |
| NZ_CP014948.1_4463713..4568937_00062 | 743 | t4cp2 | typeG | mandatory | 4.400e-31 | 100.700 | 0.955 | 0.318 |
| NZ_CP014948.1_4463713..4568937_00063 | 248 | G_tfc7 | typeG | accessory | 1.100e-103 | 339.000 | 1.000 | 1.000 |
| NZ_CP014948.1_4463713..4568937_00067 | 112 | G_tfc8 | typeG | accessory | 6.600e-41 | 131.700 | 0.922 | 0.929 |
| NZ_CP014948.1_4463713..4568937_00068 | 79 | G_tfc9 | typeG | accessory | 8.900e-31 | 99.000 | 1.000 | 1.000 |
| NZ_CP014948.1_4463713..4568937_00069 | 118 | G_tfc10 | typeG | accessory | 5.800e-42 | 134.900 | 0.933 | 0.932 |
| NZ_CP014948.1_4463713..4568937_00070 | 128 | G_tfc11 | typeG | accessory | 6.500e-50 | 160.900 | 0.923 | 0.938 |
| NZ_CP014948.1_4463713..4568937_00071 | 219 | G_tfc12 | typeG | accessory | 6.200e-91 | 296.300 | 0.950 | 0.968 |
| NZ_CP014948.1_4463713..4568937_00072 | 294 | G_tfc13 | typeG | accessory | 2.600e-118 | 387.200 | 0.955 | 0.956 |
| NZ_CP014948.1_4463713..4568937_00073 | 501 | G_tfc14 | typeG | accessory | 4.800e-184 | 605.700 | 0.996 | 0.992 |
| NZ_CP014948.1_4463713..4568937_00074 | 147 | G_tfc15 | typeG | accessory | 2.300e-60 | 195.600 | 0.957 | 0.925 |
| NZ_CP014948.1_4463713..4568937_00075 | 982 | virb4 | typeG | mandatory | 3.000e-89 | 293.300 | 0.807 | 0.899 |
| NZ_CP014948.1_4463713..4568937_00082 | 312 | G_tfc23 | typeG | accessory | 1.700e-133 | 437.700 | 0.973 | 0.987 |
| NZ_CP014948.1_4463713..4568937_00083 | 460 | G_tfc22 | typeG | accessory | 3.900e-180 | 592.200 | 0.987 | 0.974 |
| NZ_CP014948.1_4463713..4568937_00084 | 105 | G_tfc18 | typeG | accessory | 5.600e-20 | 64.200 | 0.912 | 0.876 |
| NZ_CP014948.1_4463713..4568937_00085 | 503 | G_tfc19 | typeG | accessory | 1.300e-221 | 729.300 | 0.986 | 0.994 |
| NZ_CP014948.1_4463713..4568937_00116 | 639 | MOBH | typeG | mandatory | 8.800e-44 | 142.400 | 0.868 | 0.268 |
| NZ_CP014999.1_5635771..5781235_00135 | 251 | G_tfc3 | typeG | accessory | 4.200e-95 | 310.800 | 0.967 | 0.976 |
| NZ_CP014999.1_5635771..5781235_00137 | 166 | G_tfc5 | typeG | accessory | 3.800e-57 | 186.200 | 0.977 | 1.000 |
| NZ_CP014999.1_5635771..5781235_00139 | 743 | t4cp2 | typeG | mandatory | 7.000e-31 | 100.700 | 0.955 | 0.318 |
| NZ_CP014999.1_5635771..5781235_00140 | 249 | G_tfc7 | typeG | accessory | 1.600e-103 | 339.100 | 1.000 | 0.996 |
| NZ_CP014999.1_5635771..5781235_00146 | 112 | G_tfc8 | typeG | accessory | 2.300e-40 | 130.600 | 0.914 | 0.920 |
| NZ_CP014999.1_5635771..5781235_00147 | 79 | G_tfc9 | typeG | accessory | 1.800e-30 | 98.700 | 1.000 | 1.000 |
| NZ_CP014999.1_5635771..5781235_00148 | 118 | G_tfc10 | typeG | accessory | 1.100e-43 | 141.000 | 0.875 | 0.890 |
| NZ_CP014999.1_5635771..5781235_00149 | 128 | G_tfc11 | typeG | accessory | 8.000e-50 | 161.300 | 0.923 | 0.938 |
| NZ_CP014999.1_5635771..5781235_00150 | 219 | G_tfc12 | typeG | accessory | 1.200e-91 | 299.200 | 0.950 | 0.968 |
| NZ_CP014999.1_5635771..5781235_00151 | 294 | G_tfc13 | typeG | accessory | 6.100e-118 | 386.700 | 0.955 | 0.956 |
| NZ_CP014999.1_5635771..5781235_00152 | 501 | G_tfc14 | typeG | accessory | 1.600e-183 | 604.500 | 0.996 | 0.992 |
| NZ_CP014999.1_5635771..5781235_00153 | 147 | G_tfc15 | typeG | accessory | 6.300e-60 | 194.700 | 0.957 | 0.925 |
| NZ_CP014999.1_5635771..5781235_00154 | 980 | virb4 | typeG | mandatory | 2.000e-90 | 297.800 | 0.806 | 0.898 |
| NZ_CP014999.1_5635771..5781235_00158 | 143 | G_tfc24 | typeG | accessory | 1.400e-50 | 164.300 | 0.907 | 0.888 |
| NZ_CP014999.1_5635771..5781235_00159 | 312 | G_tfc23 | typeG | accessory | 2.900e-133 | 437.500 | 0.973 | 0.987 |
| NZ_CP014999.1_5635771..5781235_00160 | 460 | G_tfc22 | typeG | accessory | 4.500e-180 | 592.600 | 0.987 | 0.974 |
| NZ_CP014999.1_5635771..5781235_00161 | 105 | G_tfc18 | typeG | accessory | 1.500e-19 | 63.500 | 0.895 | 0.867 |
| NZ_CP014999.1_5635771..5781235_00162 | 508 | G_tfc19 | typeG | accessory | 1.400e-220 | 726.600 | 0.992 | 0.990 |
| NZ_CP014999.1_5635771..5781235_00180 | 639 | MOBH | typeG | mandatory | 3.000e-44 | 144.500 | 0.868 | 0.268 |
| NZ_CP015001.1_5366986..5515052_00097 | 251 | G_tfc3 | typeG | accessory | 4.200e-95 | 310.800 | 0.967 | 0.976 |
| NZ_CP015001.1_5366986..5515052_00099 | 166 | G_tfc5 | typeG | accessory | 3.800e-57 | 186.200 | 0.977 | 1.000 |
| NZ_CP015001.1_5366986..5515052_00101 | 743 | t4cp2 | typeG | mandatory | 7.000e-31 | 100.700 | 0.955 | 0.318 |
| NZ_CP015001.1_5366986..5515052_00102 | 249 | G_tfc7 | typeG | accessory | 1.600e-103 | 339.100 | 1.000 | 0.996 |
| NZ_CP015001.1_5366986..5515052_00108 | 112 | G_tfc8 | typeG | accessory | 2.200e-40 | 130.600 | 0.914 | 0.920 |
| NZ_CP015001.1_5366986..5515052_00109 | 79 | G_tfc9 | typeG | accessory | 1.800e-30 | 98.700 | 1.000 | 1.000 |
| NZ_CP015001.1_5366986..5515052_00110 | 118 | G_tfc10 | typeG | accessory | 1.100e-43 | 141.000 | 0.875 | 0.890 |
| NZ_CP015001.1_5366986..5515052_00111 | 128 | G_tfc11 | typeG | accessory | 7.900e-50 | 161.300 | 0.923 | 0.938 |
| NZ_CP015001.1_5366986..5515052_00112 | 219 | G_tfc12 | typeG | accessory | 1.200e-91 | 299.200 | 0.950 | 0.968 |
| NZ_CP015001.1_5366986..5515052_00113 | 294 | G_tfc13 | typeG | accessory | 6.100e-118 | 386.700 | 0.955 | 0.956 |
| NZ_CP015001.1_5366986..5515052_00114 | 501 | G_tfc14 | typeG | accessory | 1.600e-183 | 604.500 | 0.996 | 0.992 |
| NZ_CP015001.1_5366986..5515052_00115 | 147 | G_tfc15 | typeG | accessory | 6.300e-60 | 194.700 | 0.957 | 0.925 |
| NZ_CP015001.1_5366986..5515052_00116 | 980 | virb4 | typeG | mandatory | 2.000e-90 | 297.800 | 0.806 | 0.898 |
| NZ_CP015001.1_5366986..5515052_00120 | 143 | G_tfc24 | typeG | accessory | 1.400e-50 | 164.300 | 0.907 | 0.888 |
| NZ_CP015001.1_5366986..5515052_00121 | 312 | G_tfc23 | typeG | accessory | 2.900e-133 | 437.500 | 0.973 | 0.987 |
| NZ_CP015001.1_5366986..5515052_00122 | 460 | G_tfc22 | typeG | accessory | 4.400e-180 | 592.600 | 0.987 | 0.974 |
| NZ_CP015001.1_5366986..5515052_00123 | 105 | G_tfc18 | typeG | accessory | 1.400e-19 | 63.500 | 0.895 | 0.867 |
| NZ_CP015001.1_5366986..5515052_00124 | 508 | G_tfc19 | typeG | accessory | 1.400e-220 | 726.600 | 0.992 | 0.990 |
| NZ_CP015001.1_5366986..5515052_00142 | 639 | MOBH | typeG | mandatory | 3.000e-44 | 144.500 | 0.868 | 0.268 |
| NZ_CP015002.1_5572504..5717969_00135 | 251 | G_tfc3 | typeG | accessory | 4.200e-95 | 310.800 | 0.967 | 0.976 |
| NZ_CP015002.1_5572504..5717969_00137 | 166 | G_tfc5 | typeG | accessory | 3.800e-57 | 186.200 | 0.977 | 1.000 |
| NZ_CP015002.1_5572504..5717969_00139 | 743 | t4cp2 | typeG | mandatory | 7.000e-31 | 100.700 | 0.955 | 0.318 |
| NZ_CP015002.1_5572504..5717969_00140 | 249 | G_tfc7 | typeG | accessory | 1.600e-103 | 339.100 | 1.000 | 0.996 |
| NZ_CP015002.1_5572504..5717969_00146 | 112 | G_tfc8 | typeG | accessory | 2.300e-40 | 130.600 | 0.914 | 0.920 |
| NZ_CP015002.1_5572504..5717969_00147 | 79 | G_tfc9 | typeG | accessory | 1.800e-30 | 98.700 | 1.000 | 1.000 |
| NZ_CP015002.1_5572504..5717969_00148 | 118 | G_tfc10 | typeG | accessory | 1.100e-43 | 141.000 | 0.875 | 0.890 |
| NZ_CP015002.1_5572504..5717969_00149 | 128 | G_tfc11 | typeG | accessory | 8.000e-50 | 161.300 | 0.923 | 0.938 |
| NZ_CP015002.1_5572504..5717969_00150 | 219 | G_tfc12 | typeG | accessory | 1.200e-91 | 299.200 | 0.950 | 0.968 |
| NZ_CP015002.1_5572504..5717969_00151 | 294 | G_tfc13 | typeG | accessory | 6.100e-118 | 386.700 | 0.955 | 0.956 |
| NZ_CP015002.1_5572504..5717969_00152 | 501 | G_tfc14 | typeG | accessory | 1.600e-183 | 604.500 | 0.996 | 0.992 |
| NZ_CP015002.1_5572504..5717969_00153 | 147 | G_tfc15 | typeG | accessory | 6.300e-60 | 194.700 | 0.957 | 0.925 |
| NZ_CP015002.1_5572504..5717969_00154 | 980 | virb4 | typeG | mandatory | 2.000e-90 | 297.800 | 0.806 | 0.898 |
| NZ_CP015002.1_5572504..5717969_00158 | 143 | G_tfc24 | typeG | accessory | 1.400e-50 | 164.300 | 0.907 | 0.888 |
| NZ_CP015002.1_5572504..5717969_00159 | 312 | G_tfc23 | typeG | accessory | 2.900e-133 | 437.500 | 0.973 | 0.987 |
| NZ_CP015002.1_5572504..5717969_00160 | 460 | G_tfc22 | typeG | accessory | 4.500e-180 | 592.600 | 0.987 | 0.974 |
| NZ_CP015002.1_5572504..5717969_00161 | 105 | G_tfc18 | typeG | accessory | 1.500e-19 | 63.500 | 0.895 | 0.867 |
| NZ_CP015002.1_5572504..5717969_00162 | 508 | G_tfc19 | typeG | accessory | 1.400e-220 | 726.600 | 0.992 | 0.990 |
| NZ_CP015002.1_5572504..5717969_00180 | 639 | MOBH | typeG | mandatory | 3.000e-44 | 144.500 | 0.868 | 0.268 |
| NZ_CP015117.1_2266451..2368432_00072 | 251 | G_tfc3 | typeG | accessory | 4.000e-96 | 313.600 | 0.988 | 0.996 |
| NZ_CP015117.1_2266451..2368432_00074 | 166 | G_tfc5 | typeG | accessory | 2.900e-57 | 186.100 | 0.971 | 0.994 |
| NZ_CP015117.1_2266451..2368432_00076 | 743 | t4cp2 | typeG | mandatory | 4.800e-31 | 100.700 | 0.955 | 0.318 |
| NZ_CP015117.1_2266451..2368432_00077 | 249 | G_tfc7 | typeG | accessory | 1.100e-103 | 339.100 | 1.000 | 0.996 |
| NZ_CP015117.1_2266451..2368432_00102 | 112 | G_tfc8 | typeG | accessory | 7.500e-41 | 131.700 | 0.914 | 0.920 |
| NZ_CP015117.1_2266451..2368432_00103 | 79 | G_tfc9 | typeG | accessory | 1.400e-30 | 98.500 | 1.000 | 1.000 |
| NZ_CP015117.1_2266451..2368432_00104 | 118 | G_tfc10 | typeG | accessory | 7.800e-44 | 141.000 | 0.875 | 0.890 |
| NZ_CP015117.1_2266451..2368432_00105 | 128 | G_tfc11 | typeG | accessory | 5.600e-50 | 161.300 | 0.923 | 0.938 |
| NZ_CP015117.1_2266451..2368432_00106 | 219 | G_tfc12 | typeG | accessory | 8.700e-92 | 299.200 | 0.950 | 0.968 |
| NZ_CP015117.1_2266451..2368432_00107 | 294 | G_tfc13 | typeG | accessory | 4.300e-118 | 386.700 | 0.955 | 0.956 |
| NZ_CP015117.1_2266451..2368432_00108 | 501 | G_tfc14 | typeG | accessory | 1.100e-183 | 604.600 | 0.996 | 0.992 |
| NZ_CP015117.1_2266451..2368432_00109 | 147 | G_tfc15 | typeG | accessory | 4.400e-60 | 194.700 | 0.957 | 0.925 |
| NZ_CP015117.1_2266451..2368432_00110 | 980 | virb4 | typeG | mandatory | 1.300e-90 | 297.900 | 0.806 | 0.898 |
| NZ_CP015117.1_2266451..2368432_00115 | 143 | G_tfc24 | typeG | accessory | 1.400e-49 | 160.600 | 0.907 | 0.888 |
| NZ_CP015117.1_2266451..2368432_00116 | 312 | G_tfc23 | typeG | accessory | 3.700e-134 | 440.000 | 0.973 | 0.987 |
| NZ_CP015117.1_2266451..2368432_00117 | 460 | G_tfc22 | typeG | accessory | 5.400e-180 | 591.800 | 0.987 | 0.974 |
| NZ_CP015117.1_2266451..2368432_00118 | 115 | G_tfc18 | typeG | accessory | 4.200e-29 | 93.700 | 0.912 | 0.887 |
| NZ_CP015117.1_2266451..2368432_00119 | 504 | G_tfc19 | typeG | accessory | 1.600e-222 | 732.500 | 0.984 | 0.990 |
| NZ_CP015117.1_2266451..2368432_00126 | 639 | MOBH | typeG | mandatory | 4.600e-44 | 143.400 | 0.868 | 0.268 |
| NZ_CP016955.1_1326863..1412944_00002 | 640 | MOBH | typeG | mandatory | 2.700e-44 | 143.900 | 0.868 | 0.267 |
| NZ_CP016955.1_1326863..1412944_00009 | 504 | G_tfc19 | typeG | accessory | 2.200e-223 | 735.000 | 0.988 | 0.994 |
| NZ_CP016955.1_1326863..1412944_00010 | 115 | G_tfc18 | typeG | accessory | 3.600e-29 | 93.600 | 0.912 | 0.887 |
| NZ_CP016955.1_1326863..1412944_00011 | 460 | G_tfc22 | typeG | accessory | 5.800e-180 | 591.400 | 0.989 | 0.976 |
| NZ_CP016955.1_1326863..1412944_00012 | 312 | G_tfc23 | typeG | accessory | 2.700e-134 | 440.100 | 0.973 | 0.987 |
| NZ_CP016955.1_1326863..1412944_00013 | 143 | G_tfc24 | typeG | accessory | 3.600e-51 | 165.400 | 0.907 | 0.888 |
| NZ_CP016955.1_1326863..1412944_00019 | 983 | virb4 | typeG | mandatory | 1.200e-89 | 294.500 | 0.810 | 0.899 |
| NZ_CP016955.1_1326863..1412944_00020 | 147 | G_tfc15 | typeG | accessory | 1.700e-59 | 192.600 | 0.957 | 0.925 |
| NZ_CP016955.1_1326863..1412944_00021 | 501 | G_tfc14 | typeG | accessory | 2.200e-184 | 606.600 | 0.996 | 0.992 |
| NZ_CP016955.1_1326863..1412944_00022 | 294 | G_tfc13 | typeG | accessory | 1.000e-118 | 388.400 | 0.955 | 0.956 |
| NZ_CP016955.1_1326863..1412944_00023 | 219 | G_tfc12 | typeG | accessory | 1.300e-91 | 298.400 | 0.950 | 0.968 |
| NZ_CP016955.1_1326863..1412944_00024 | 128 | G_tfc11 | typeG | accessory | 9.900e-50 | 160.200 | 0.923 | 0.938 |
| NZ_CP016955.1_1326863..1412944_00025 | 118 | G_tfc10 | typeG | accessory | 5.400e-44 | 141.300 | 0.925 | 0.924 |
| NZ_CP016955.1_1326863..1412944_00026 | 79 | G_tfc9 | typeG | accessory | 1.900e-30 | 97.800 | 1.000 | 1.000 |
| NZ_CP016955.1_1326863..1412944_00027 | 112 | G_tfc8 | typeG | accessory | 5.700e-41 | 131.800 | 0.931 | 0.938 |
| NZ_CP016955.1_1326863..1412944_00033 | 248 | G_tfc7 | typeG | accessory | 9.600e-104 | 339.000 | 1.000 | 1.000 |
| NZ_CP016955.1_1326863..1412944_00034 | 743 | t4cp2 | typeG | mandatory | 4.000e-31 | 100.700 | 0.955 | 0.318 |
| NZ_CP016955.1_1326863..1412944_00036 | 166 | G_tfc5 | typeG | accessory | 2.500e-57 | 186.000 | 0.971 | 0.994 |
| NZ_CP016955.1_1326863..1412944_00038 | 251 | G_tfc3 | typeG | accessory | 7.000e-96 | 312.600 | 0.967 | 0.976 |
| NZ_CP017099.1_5362518..5455159_00081 | 250 | G_tfc3 | typeG | accessory | 6.300e-96 | 312.900 | 0.971 | 0.984 |
| NZ_CP017099.1_5362518..5455159_00083 | 166 | G_tfc5 | typeG | accessory | 2.600e-57 | 186.100 | 0.971 | 0.994 |
| NZ_CP017099.1_5362518..5455159_00085 | 743 | t4cp2 | typeG | mandatory | 4.300e-31 | 100.700 | 0.955 | 0.318 |
| NZ_CP017099.1_5362518..5455159_00086 | 248 | G_tfc7 | typeG | accessory | 6.300e-104 | 339.700 | 1.000 | 1.000 |
| NZ_CP017099.1_5362518..5455159_00090 | 112 | G_tfc8 | typeG | accessory | 3.200e-40 | 129.500 | 0.931 | 0.938 |
| NZ_CP017099.1_5362518..5455159_00091 | 79 | G_tfc9 | typeG | accessory | 5.200e-31 | 99.700 | 1.000 | 1.000 |
| NZ_CP017099.1_5362518..5455159_00092 | 118 | G_tfc10 | typeG | accessory | 4.200e-44 | 141.700 | 0.925 | 0.924 |
| NZ_CP017099.1_5362518..5455159_00093 | 128 | G_tfc11 | typeG | accessory | 1.100e-49 | 160.100 | 0.923 | 0.938 |
| NZ_CP017099.1_5362518..5455159_00094 | 219 | G_tfc12 | typeG | accessory | 2.700e-91 | 297.400 | 0.950 | 0.968 |
| NZ_CP017099.1_5362518..5455159_00095 | 294 | G_tfc13 | typeG | accessory | 2.400e-118 | 387.300 | 0.941 | 0.956 |
| NZ_CP017099.1_5362518..5455159_00096 | 501 | G_tfc14 | typeG | accessory | 1.000e-184 | 607.800 | 0.996 | 0.992 |
| NZ_CP017099.1_5362518..5455159_00097 | 147 | G_tfc15 | typeG | accessory | 4.000e-60 | 194.700 | 0.957 | 0.925 |
| NZ_CP017099.1_5362518..5455159_00098 | 980 | virb4 | typeG | mandatory | 4.000e-90 | 296.100 | 0.806 | 0.898 |
| NZ_CP017099.1_5362518..5455159_00104 | 143 | G_tfc24 | typeG | accessory | 8.400e-51 | 164.400 | 0.907 | 0.888 |
| NZ_CP017099.1_5362518..5455159_00105 | 312 | G_tfc23 | typeG | accessory | 3.300e-134 | 440.000 | 0.973 | 0.987 |
| NZ_CP017099.1_5362518..5455159_00106 | 463 | G_tfc22 | typeG | accessory | 3.700e-180 | 592.200 | 0.989 | 0.970 |
| NZ_CP017099.1_5362518..5455159_00107 | 121 | G_tfc18 | typeG | accessory | 1.300e-27 | 88.700 | 0.930 | 0.860 |
| NZ_CP017099.1_5362518..5455159_00108 | 510 | G_tfc19 | typeG | accessory | 3.400e-221 | 727.900 | 0.994 | 0.988 |
| NZ_CP017099.1_5362518..5455159_00112 | 629 | MOBH | typeG | mandatory | 2.800e-44 | 143.900 | 0.868 | 0.272 |
| NZ_CP017293.1_4783902..4873008_00037 | 374 | G_tfc2 | typeG | accessory | 2.000e-71 | 232.400 | 0.782 | 0.570 |
| NZ_CP017293.1_4783902..4873008_00068 | 251 | G_tfc3 | typeG | accessory | 3.300e-96 | 313.600 | 0.988 | 0.996 |
| NZ_CP017293.1_4783902..4873008_00070 | 166 | G_tfc5 | typeG | accessory | 2.300e-57 | 186.100 | 0.971 | 0.994 |
| NZ_CP017293.1_4783902..4873008_00072 | 743 | t4cp2 | typeG | mandatory | 3.900e-31 | 100.700 | 0.955 | 0.318 |
| NZ_CP017293.1_4783902..4873008_00073 | 249 | G_tfc7 | typeG | accessory | 1.400e-103 | 338.500 | 1.000 | 0.996 |
| NZ_CP017293.1_4783902..4873008_00078 | 112 | G_tfc8 | typeG | accessory | 1.300e-40 | 130.600 | 0.914 | 0.920 |
| NZ_CP017293.1_4783902..4873008_00079 | 49 | G_tfc9 | typeG | accessory | 7.500e-18 | 57.400 | 0.620 | 1.000 |
| NZ_CP017293.1_4783902..4873008_00080 | 118 | G_tfc10 | typeG | accessory | 6.400e-44 | 141.000 | 0.875 | 0.890 |
| NZ_CP017293.1_4783902..4873008_00081 | 128 | G_tfc11 | typeG | accessory | 4.500e-50 | 161.300 | 0.923 | 0.938 |
| NZ_CP017293.1_4783902..4873008_00082 | 219 | G_tfc12 | typeG | accessory | 7.100e-92 | 299.200 | 0.950 | 0.968 |
| NZ_CP017293.1_4783902..4873008_00083 | 294 | G_tfc13 | typeG | accessory | 1.500e-118 | 387.800 | 0.955 | 0.956 |
| NZ_CP017293.1_4783902..4873008_00084 | 501 | G_tfc14 | typeG | accessory | 9.800e-185 | 607.800 | 0.996 | 0.992 |
| NZ_CP017293.1_4783902..4873008_00085 | 147 | G_tfc15 | typeG | accessory | 3.400e-60 | 194.800 | 0.957 | 0.925 |
| NZ_CP017293.1_4783902..4873008_00086 | 980 | virb4 | typeG | mandatory | 8.200e-90 | 295.000 | 0.807 | 0.899 |
| NZ_CP017293.1_4783902..4873008_00091 | 143 | G_tfc24 | typeG | accessory | 3.900e-51 | 165.300 | 0.914 | 0.895 |
| NZ_CP017293.1_4783902..4873008_00092 | 312 | G_tfc23 | typeG | accessory | 9.600e-134 | 438.300 | 0.973 | 0.987 |
| NZ_CP017293.1_4783902..4873008_00093 | 462 | G_tfc22 | typeG | accessory | 4.100e-180 | 591.900 | 0.987 | 0.970 |
| NZ_CP017293.1_4783902..4873008_00094 | 115 | G_tfc18 | typeG | accessory | 3.200e-29 | 93.800 | 0.912 | 0.887 |
| NZ_CP017293.1_4783902..4873008_00095 | 508 | G_tfc19 | typeG | accessory | 1.100e-220 | 726.100 | 0.992 | 0.990 |
| NZ_CP017293.1_4783902..4873008_00102 | 640 | MOBH | typeG | mandatory | 2.700e-44 | 143.900 | 0.868 | 0.267 |
| NZ_CP017353.1_599732..698016_00085 | 251 | G_tfc3 | typeG | accessory | 8.700e-96 | 312.500 | 0.967 | 0.976 |
| NZ_CP017353.1_599732..698016_00087 | 166 | G_tfc5 | typeG | accessory | 3.700e-57 | 185.700 | 0.971 | 0.994 |
| NZ_CP017353.1_599732..698016_00089 | 743 | t4cp2 | typeG | mandatory | 4.700e-31 | 100.700 | 0.955 | 0.318 |
| NZ_CP017353.1_599732..698016_00090 | 248 | G_tfc7 | typeG | accessory | 4.700e-103 | 337.000 | 1.000 | 1.000 |
| NZ_CP017353.1_599732..698016_00095 | 112 | G_tfc8 | typeG | accessory | 2.000e-41 | 133.500 | 0.931 | 0.938 |
| NZ_CP017353.1_599732..698016_00096 | 79 | G_tfc9 | typeG | accessory | 1.400e-30 | 98.500 | 1.000 | 1.000 |
| NZ_CP017353.1_599732..698016_00097 | 118 | G_tfc10 | typeG | accessory | 7.600e-44 | 141.000 | 0.875 | 0.890 |
| NZ_CP017353.1_599732..698016_00098 | 128 | G_tfc11 | typeG | accessory | 5.500e-50 | 161.300 | 0.923 | 0.938 |
| NZ_CP017353.1_599732..698016_00099 | 219 | G_tfc12 | typeG | accessory | 8.500e-92 | 299.200 | 0.950 | 0.968 |
| NZ_CP017353.1_599732..698016_00100 | 294 | G_tfc13 | typeG | accessory | 4.200e-118 | 386.700 | 0.955 | 0.956 |
| NZ_CP017353.1_599732..698016_00101 | 501 | G_tfc14 | typeG | accessory | 1.100e-183 | 604.600 | 0.996 | 0.992 |
| NZ_CP017353.1_599732..698016_00102 | 147 | G_tfc15 | typeG | accessory | 4.300e-60 | 194.700 | 0.957 | 0.925 |
| NZ_CP017353.1_599732..698016_00103 | 980 | virb4 | typeG | mandatory | 1.300e-90 | 297.900 | 0.806 | 0.898 |
| NZ_CP017353.1_599732..698016_00109 | 143 | G_tfc24 | typeG | accessory | 9.200e-51 | 164.400 | 0.907 | 0.888 |
| NZ_CP017353.1_599732..698016_00110 | 312 | G_tfc23 | typeG | accessory | 9.200e-134 | 438.600 | 0.973 | 0.987 |
| NZ_CP017353.1_599732..698016_00112 | 356 | G_tfc22 | typeG | accessory | 4.000e-138 | 453.700 | 0.753 | 0.966 |
| NZ_CP017353.1_599732..698016_00113 | 115 | G_tfc18 | typeG | accessory | 3.900e-29 | 93.800 | 0.912 | 0.887 |
| NZ_CP017353.1_599732..698016_00114 | 511 | G_tfc19 | typeG | accessory | 7.000e-223 | 733.600 | 0.990 | 0.982 |
| NZ_CP017353.1_599732..698016_00123 | 646 | MOBH | typeG | mandatory | 4.800e-44 | 143.300 | 0.868 | 0.265 |
| NZ_CP017969.1_4492177..4610785_00084 | 251 | G_tfc3 | typeG | accessory | 2.100e-94 | 308.000 | 0.967 | 0.976 |
| NZ_CP017969.1_4492177..4610785_00086 | 166 | G_tfc5 | typeG | accessory | 9.500e-56 | 181.200 | 0.971 | 0.994 |
| NZ_CP017969.1_4492177..4610785_00088 | 743 | t4cp2 | typeG | mandatory | 5.000e-31 | 100.700 | 0.955 | 0.318 |
| NZ_CP017969.1_4492177..4610785_00089 | 248 | G_tfc7 | typeG | accessory | 7.100e-103 | 336.500 | 1.000 | 1.000 |
| NZ_CP017969.1_4492177..4610785_00094 | 112 | G_tfc8 | typeG | accessory | 5.400e-41 | 132.200 | 0.931 | 0.938 |
| NZ_CP017969.1_4492177..4610785_00095 | 79 | G_tfc9 | typeG | accessory | 1.000e-30 | 99.000 | 1.000 | 1.000 |
| NZ_CP017969.1_4492177..4610785_00096 | 118 | G_tfc10 | typeG | accessory | 3.900e-42 | 135.600 | 0.925 | 0.924 |
| NZ_CP017969.1_4492177..4610785_00097 | 128 | G_tfc11 | typeG | accessory | 8.300e-50 | 160.800 | 0.923 | 0.938 |
| NZ_CP017969.1_4492177..4610785_00098 | 219 | G_tfc12 | typeG | accessory | 1.600e-91 | 298.400 | 0.950 | 0.968 |
| NZ_CP017969.1_4492177..4610785_00099 | 294 | G_tfc13 | typeG | accessory | 1.800e-118 | 388.000 | 0.955 | 0.956 |
| NZ_CP017969.1_4492177..4610785_00100 | 501 | G_tfc14 | typeG | accessory | 1.200e-184 | 607.800 | 0.996 | 0.992 |
| NZ_CP017969.1_4492177..4610785_00101 | 147 | G_tfc15 | typeG | accessory | 1.600e-59 | 193.000 | 0.957 | 0.925 |
| NZ_CP017969.1_4492177..4610785_00102 | 980 | virb4 | typeG | mandatory | 4.000e-89 | 293.000 | 0.806 | 0.898 |
| NZ_CP017969.1_4492177..4610785_00114 | 143 | G_tfc24 | typeG | accessory | 3.600e-51 | 165.800 | 0.914 | 0.895 |
| NZ_CP017969.1_4492177..4610785_00115 | 312 | G_tfc23 | typeG | accessory | 9.800e-134 | 438.600 | 0.976 | 0.990 |
| NZ_CP017969.1_4492177..4610785_00116 | 460 | G_tfc22 | typeG | accessory | 3.400e-180 | 592.500 | 0.987 | 0.974 |
| NZ_CP017969.1_4492177..4610785_00117 | 115 | G_tfc18 | typeG | accessory | 5.600e-29 | 93.300 | 0.912 | 0.887 |
| NZ_CP017969.1_4492177..4610785_00118 | 514 | G_tfc19 | typeG | accessory | 3.200e-222 | 731.500 | 0.992 | 0.979 |
| NZ_CP017969.1_4492177..4610785_00130 | 639 | MOBH | typeG | mandatory | 4.800e-44 | 143.400 | 0.868 | 0.268 |
| NZ_CP020703.1_4867806..4986414_00083 | 251 | G_tfc3 | typeG | accessory | 2.100e-94 | 308.000 | 0.967 | 0.976 |
| NZ_CP020703.1_4867806..4986414_00085 | 166 | G_tfc5 | typeG | accessory | 9.500e-56 | 181.200 | 0.971 | 0.994 |
| NZ_CP020703.1_4867806..4986414_00087 | 743 | t4cp2 | typeG | mandatory | 5.000e-31 | 100.700 | 0.955 | 0.318 |
| NZ_CP020703.1_4867806..4986414_00088 | 248 | G_tfc7 | typeG | accessory | 7.100e-103 | 336.500 | 1.000 | 1.000 |
| NZ_CP020703.1_4867806..4986414_00093 | 112 | G_tfc8 | typeG | accessory | 5.400e-41 | 132.200 | 0.931 | 0.938 |
| NZ_CP020703.1_4867806..4986414_00094 | 79 | G_tfc9 | typeG | accessory | 1.000e-30 | 99.000 | 1.000 | 1.000 |
| NZ_CP020703.1_4867806..4986414_00095 | 118 | G_tfc10 | typeG | accessory | 3.900e-42 | 135.600 | 0.925 | 0.924 |
| NZ_CP020703.1_4867806..4986414_00096 | 128 | G_tfc11 | typeG | accessory | 8.300e-50 | 160.800 | 0.923 | 0.938 |
| NZ_CP020703.1_4867806..4986414_00097 | 219 | G_tfc12 | typeG | accessory | 1.600e-91 | 298.400 | 0.950 | 0.968 |
| NZ_CP020703.1_4867806..4986414_00098 | 294 | G_tfc13 | typeG | accessory | 1.800e-118 | 388.000 | 0.955 | 0.956 |
| NZ_CP020703.1_4867806..4986414_00099 | 501 | G_tfc14 | typeG | accessory | 1.200e-184 | 607.800 | 0.996 | 0.992 |
| NZ_CP020703.1_4867806..4986414_00100 | 147 | G_tfc15 | typeG | accessory | 1.000e-57 | 187.100 | 0.957 | 0.925 |
| NZ_CP020703.1_4867806..4986414_00101 | 980 | virb4 | typeG | mandatory | 4.000e-89 | 293.000 | 0.806 | 0.898 |
| NZ_CP020703.1_4867806..4986414_00113 | 143 | G_tfc24 | typeG | accessory | 3.600e-51 | 165.800 | 0.914 | 0.895 |
| NZ_CP020703.1_4867806..4986414_00114 | 312 | G_tfc23 | typeG | accessory | 9.800e-134 | 438.600 | 0.976 | 0.990 |
| NZ_CP020703.1_4867806..4986414_00115 | 279 | G_tfc22 | typeG | accessory | 1.700e-105 | 346.300 | 0.558 | 0.910 |
| NZ_CP020703.1_4867806..4986414_00117 | 115 | G_tfc18 | typeG | accessory | 5.600e-29 | 93.300 | 0.912 | 0.887 |
| NZ_CP020703.1_4867806..4986414_00118 | 514 | G_tfc19 | typeG | accessory | 3.200e-222 | 731.500 | 0.992 | 0.979 |
| NZ_CP020703.1_4867806..4986414_00130 | 639 | MOBH | typeG | mandatory | 4.800e-44 | 143.400 | 0.868 | 0.268 |
| NZ_CP020704.1_4986378..5078349_00080 | 251 | G_tfc3 | typeG | accessory | 3.600e-96 | 313.600 | 0.988 | 0.996 |
| NZ_CP020704.1_4986378..5078349_00082 | 166 | G_tfc5 | typeG | accessory | 2.600e-57 | 186.100 | 0.971 | 0.994 |
| NZ_CP020704.1_4986378..5078349_00084 | 743 | t4cp2 | typeG | mandatory | 4.300e-31 | 100.700 | 0.955 | 0.318 |
| NZ_CP020704.1_4986378..5078349_00085 | 249 | G_tfc7 | typeG | accessory | 9.900e-104 | 339.100 | 1.000 | 0.996 |
| NZ_CP020704.1_4986378..5078349_00089 | 112 | G_tfc8 | typeG | accessory | 3.000e-41 | 132.800 | 0.922 | 0.929 |
| NZ_CP020704.1_4986378..5078349_00090 | 79 | G_tfc9 | typeG | accessory | 1.000e-30 | 98.800 | 1.000 | 1.000 |
| NZ_CP020704.1_4986378..5078349_00091 | 118 | G_tfc10 | typeG | accessory | 1.100e-44 | 143.700 | 0.883 | 0.890 |
| NZ_CP020704.1_4986378..5078349_00092 | 128 | G_tfc11 | typeG | accessory | 7.200e-50 | 160.700 | 0.923 | 0.938 |
| NZ_CP020704.1_4986378..5078349_00093 | 219 | G_tfc12 | typeG | accessory | 1.400e-91 | 298.400 | 0.950 | 0.968 |
| NZ_CP020704.1_4986378..5078349_00094 | 294 | G_tfc13 | typeG | accessory | 6.100e-118 | 386.000 | 0.955 | 0.956 |
| NZ_CP020704.1_4986378..5078349_00095 | 501 | G_tfc14 | typeG | accessory | 8.400e-184 | 604.800 | 0.996 | 0.992 |
| NZ_CP020704.1_4986378..5078349_00096 | 147 | G_tfc15 | typeG | accessory | 3.900e-60 | 194.700 | 0.957 | 0.925 |
| NZ_CP020704.1_4986378..5078349_00097 | 980 | virb4 | typeG | mandatory | 6.300e-90 | 295.500 | 0.807 | 0.899 |
| NZ_CP020704.1_4986378..5078349_00101 | 143 | G_tfc24 | typeG | accessory | 5.600e-51 | 164.900 | 0.914 | 0.895 |
| NZ_CP020704.1_4986378..5078349_00102 | 312 | G_tfc23 | typeG | accessory | 5.400e-134 | 439.300 | 0.976 | 0.990 |
| NZ_CP020704.1_4986378..5078349_00103 | 460 | G_tfc22 | typeG | accessory | 1.400e-179 | 590.200 | 0.987 | 0.974 |
| NZ_CP020704.1_4986378..5078349_00104 | 115 | G_tfc18 | typeG | accessory | 8.200e-29 | 92.600 | 0.895 | 0.878 |
| NZ_CP020704.1_4986378..5078349_00105 | 503 | G_tfc19 | typeG | accessory | 9.800e-222 | 729.700 | 0.984 | 0.992 |
| NZ_CP020704.1_4986378..5078349_00111 | 639 | MOBH | typeG | mandatory | 7.300e-44 | 142.600 | 0.868 | 0.268 |
| NZ_CP021775.1_5906126..5994299_00037 | 374 | G_tfc2 | typeG | accessory | 9.000e-73 | 236.700 | 0.891 | 0.711 |
| NZ_CP021775.1_5906126..5994299_00068 | 250 | G_tfc3 | typeG | accessory | 5.300e-96 | 313.000 | 0.967 | 0.980 |
| NZ_CP021775.1_5906126..5994299_00070 | 166 | G_tfc5 | typeG | accessory | 2.500e-57 | 186.000 | 0.971 | 0.994 |
| NZ_CP021775.1_5906126..5994299_00072 | 743 | t4cp2 | typeG | mandatory | 3.900e-31 | 100.700 | 0.955 | 0.318 |
| NZ_CP021775.1_5906126..5994299_00073 | 248 | G_tfc7 | typeG | accessory | 9.500e-104 | 339.000 | 1.000 | 1.000 |
| NZ_CP021775.1_5906126..5994299_00078 | 112 | G_tfc8 | typeG | accessory | 1.600e-41 | 133.500 | 0.931 | 0.938 |
| NZ_CP021775.1_5906126..5994299_00079 | 79 | G_tfc9 | typeG | accessory | 1.200e-30 | 98.500 | 1.000 | 1.000 |
| NZ_CP021775.1_5906126..5994299_00080 | 118 | G_tfc10 | typeG | accessory | 6.400e-44 | 141.000 | 0.875 | 0.890 |
| NZ_CP021775.1_5906126..5994299_00081 | 128 | G_tfc11 | typeG | accessory | 4.500e-50 | 161.300 | 0.923 | 0.938 |
| NZ_CP021775.1_5906126..5994299_00082 | 219 | G_tfc12 | typeG | accessory | 7.100e-92 | 299.200 | 0.950 | 0.968 |
| NZ_CP021775.1_5906126..5994299_00083 | 294 | G_tfc13 | typeG | accessory | 3.500e-118 | 386.700 | 0.955 | 0.956 |
| NZ_CP021775.1_5906126..5994299_00084 | 501 | G_tfc14 | typeG | accessory | 8.900e-184 | 604.600 | 0.996 | 0.992 |
| NZ_CP021775.1_5906126..5994299_00085 | 147 | G_tfc15 | typeG | accessory | 3.600e-60 | 194.700 | 0.957 | 0.925 |
| NZ_CP021775.1_5906126..5994299_00086 | 980 | virb4 | typeG | mandatory | 1.100e-90 | 297.900 | 0.806 | 0.898 |
| NZ_CP021775.1_5906126..5994299_00091 | 143 | G_tfc24 | typeG | accessory | 7.600e-51 | 164.400 | 0.907 | 0.888 |
| NZ_CP021775.1_5906126..5994299_00092 | 312 | G_tfc23 | typeG | accessory | 7.600e-134 | 438.600 | 0.973 | 0.987 |
| NZ_CP021775.1_5906126..5994299_00093 | 462 | G_tfc22 | typeG | accessory | 4.500e-180 | 591.800 | 0.987 | 0.970 |
| NZ_CP021775.1_5906126..5994299_00094 | 115 | G_tfc18 | typeG | accessory | 3.200e-29 | 93.800 | 0.912 | 0.887 |
| NZ_CP021775.1_5906126..5994299_00095 | 511 | G_tfc19 | typeG | accessory | 5.800e-223 | 733.600 | 0.990 | 0.982 |
| NZ_CP021775.1_5906126..5994299_00102 | 640 | MOBH | typeG | mandatory | 2.700e-44 | 143.900 | 0.868 | 0.267 |
| NZ_CP021999.1_5296771..5387683_00067 | 251 | G_tfc3 | typeG | accessory | 6.500e-97 | 315.900 | 0.988 | 0.996 |
| NZ_CP021999.1_5296771..5387683_00069 | 166 | G_tfc5 | typeG | accessory | 2.300e-57 | 186.100 | 0.971 | 0.994 |
| NZ_CP021999.1_5296771..5387683_00071 | 743 | t4cp2 | typeG | mandatory | 3.900e-31 | 100.700 | 0.955 | 0.318 |
| NZ_CP021999.1_5296771..5387683_00072 | 248 | G_tfc7 | typeG | accessory | 7.200e-104 | 339.400 | 1.000 | 1.000 |
| NZ_CP021999.1_5296771..5387683_00076 | 112 | G_tfc8 | typeG | accessory | 5.800e-41 | 131.700 | 0.914 | 0.920 |
| NZ_CP021999.1_5296771..5387683_00077 | 79 | G_tfc9 | typeG | accessory | 7.800e-31 | 99.000 | 1.000 | 1.000 |
| NZ_CP021999.1_5296771..5387683_00078 | 118 | G_tfc10 | typeG | accessory | 6.400e-44 | 141.000 | 0.875 | 0.890 |
| NZ_CP021999.1_5296771..5387683_00079 | 128 | G_tfc11 | typeG | accessory | 7.800e-50 | 160.500 | 0.923 | 0.938 |
| NZ_CP021999.1_5296771..5387683_00080 | 219 | G_tfc12 | typeG | accessory | 5.800e-91 | 296.200 | 0.941 | 0.959 |
| NZ_CP021999.1_5296771..5387683_00081 | 294 | G_tfc13 | typeG | accessory | 6.300e-117 | 382.500 | 0.941 | 0.952 |
| NZ_CP021999.1_5296771..5387683_00082 | 501 | G_tfc14 | typeG | accessory | 6.900e-185 | 608.300 | 0.996 | 0.992 |
| NZ_CP021999.1_5296771..5387683_00083 | 147 | G_tfc15 | typeG | accessory | 3.200e-60 | 194.900 | 0.957 | 0.925 |
| NZ_CP021999.1_5296771..5387683_00084 | 980 | virb4 | typeG | mandatory | 2.800e-90 | 296.500 | 0.808 | 0.900 |
| NZ_CP021999.1_5296771..5387683_00090 | 143 | G_tfc24 | typeG | accessory | 3.000e-50 | 162.400 | 0.907 | 0.888 |
| NZ_CP021999.1_5296771..5387683_00091 | 312 | G_tfc23 | typeG | accessory | 7.000e-134 | 438.800 | 0.973 | 0.987 |
| NZ_CP021999.1_5296771..5387683_00092 | 460 | G_tfc22 | typeG | accessory | 2.100e-179 | 589.600 | 0.987 | 0.974 |
| NZ_CP021999.1_5296771..5387683_00093 | 115 | G_tfc18 | typeG | accessory | 5.900e-29 | 92.900 | 0.912 | 0.887 |
| NZ_CP021999.1_5296771..5387683_00094 | 503 | G_tfc19 | typeG | accessory | 1.500e-222 | 732.200 | 0.986 | 0.994 |
| NZ_CP021999.1_5296771..5387683_00102 | 639 | MOBH | typeG | mandatory | 2.200e-44 | 144.200 | 0.868 | 0.268 |
| NZ_CP022001.1_1735211..1838035_00002 | 639 | MOBH | typeG | mandatory | 4.800e-44 | 143.400 | 0.868 | 0.268 |
| NZ_CP022001.1_1735211..1838035_00009 | 504 | G_tfc19 | typeG | accessory | 1.600e-222 | 732.500 | 0.984 | 0.990 |
| NZ_CP022001.1_1735211..1838035_00010 | 115 | G_tfc18 | typeG | accessory | 4.400e-29 | 93.700 | 0.912 | 0.887 |
| NZ_CP022001.1_1735211..1838035_00012 | 279 | G_tfc22 | typeG | accessory | 2.600e-105 | 345.700 | 0.558 | 0.910 |
| NZ_CP022001.1_1735211..1838035_00013 | 312 | G_tfc23 | typeG | accessory | 3.800e-134 | 440.000 | 0.973 | 0.987 |
| NZ_CP022001.1_1735211..1838035_00014 | 166 | G_tfc24 | typeG | accessory | 1.700e-50 | 163.600 | 0.900 | 0.759 |
| NZ_CP022001.1_1735211..1838035_00019 | 980 | virb4 | typeG | mandatory | 1.400e-90 | 297.900 | 0.806 | 0.898 |
| NZ_CP022001.1_1735211..1838035_00020 | 147 | G_tfc15 | typeG | accessory | 4.600e-60 | 194.700 | 0.957 | 0.925 |
| NZ_CP022001.1_1735211..1838035_00021 | 501 | G_tfc14 | typeG | accessory | 1.100e-183 | 604.600 | 0.996 | 0.992 |
| NZ_CP022001.1_1735211..1838035_00022 | 294 | G_tfc13 | typeG | accessory | 4.500e-118 | 386.700 | 0.955 | 0.956 |
| NZ_CP022001.1_1735211..1838035_00023 | 219 | G_tfc12 | typeG | accessory | 9.100e-92 | 299.200 | 0.950 | 0.968 |
| NZ_CP022001.1_1735211..1838035_00024 | 128 | G_tfc11 | typeG | accessory | 5.800e-50 | 161.300 | 0.923 | 0.938 |
| NZ_CP022001.1_1735211..1838035_00025 | 118 | G_tfc10 | typeG | accessory | 8.100e-44 | 141.000 | 0.875 | 0.890 |
| NZ_CP022001.1_1735211..1838035_00026 | 79 | G_tfc9 | typeG | accessory | 1.500e-30 | 98.500 | 1.000 | 1.000 |
| NZ_CP022001.1_1735211..1838035_00027 | 112 | G_tfc8 | typeG | accessory | 7.800e-41 | 131.700 | 0.914 | 0.920 |
| NZ_CP022001.1_1735211..1838035_00052 | 249 | G_tfc7 | typeG | accessory | 1.200e-103 | 339.100 | 1.000 | 0.996 |
| NZ_CP022001.1_1735211..1838035_00053 | 743 | t4cp2 | typeG | mandatory | 5.000e-31 | 100.700 | 0.955 | 0.318 |
| NZ_CP022001.1_1735211..1838035_00055 | 166 | G_tfc5 | typeG | accessory | 3.000e-57 | 186.100 | 0.971 | 0.994 |
| NZ_CP022001.1_1735211..1838035_00057 | 251 | G_tfc3 | typeG | accessory | 4.200e-96 | 313.600 | 0.988 | 0.996 |
| NZ_CP022002.1_1701475..1808580_00002 | 639 | MOBH | typeG | mandatory | 4.600e-44 | 143.400 | 0.868 | 0.268 |
| NZ_CP022002.1_1701475..1808580_00011 | 511 | G_tfc19 | typeG | accessory | 6.900e-223 | 733.600 | 0.990 | 0.982 |
| NZ_CP022002.1_1701475..1808580_00012 | 115 | G_tfc18 | typeG | accessory | 3.800e-29 | 93.800 | 0.912 | 0.887 |
| NZ_CP022002.1_1701475..1808580_00014 | 279 | G_tfc22 | typeG | accessory | 2.400e-105 | 345.700 | 0.558 | 0.910 |
| NZ_CP022002.1_1701475..1808580_00015 | 312 | G_tfc23 | typeG | accessory | 9.000e-134 | 438.600 | 0.973 | 0.987 |
| NZ_CP022002.1_1701475..1808580_00016 | 166 | G_tfc24 | typeG | accessory | 1.500e-50 | 163.600 | 0.900 | 0.759 |
| NZ_CP022002.1_1701475..1808580_00021 | 980 | virb4 | typeG | mandatory | 2.300e-90 | 297.100 | 0.806 | 0.898 |
| NZ_CP022002.1_1701475..1808580_00022 | 147 | G_tfc15 | typeG | accessory | 4.300e-60 | 194.700 | 0.957 | 0.925 |
| NZ_CP022002.1_1701475..1808580_00023 | 501 | G_tfc14 | typeG | accessory | 1.100e-183 | 604.600 | 0.996 | 0.992 |
| NZ_CP022002.1_1701475..1808580_00024 | 294 | G_tfc13 | typeG | accessory | 4.100e-118 | 386.700 | 0.955 | 0.956 |
| NZ_CP022002.1_1701475..1808580_00025 | 219 | G_tfc12 | typeG | accessory | 8.400e-92 | 299.200 | 0.950 | 0.968 |
| NZ_CP022002.1_1701475..1808580_00026 | 128 | G_tfc11 | typeG | accessory | 5.400e-50 | 161.300 | 0.923 | 0.938 |
| NZ_CP022002.1_1701475..1808580_00027 | 118 | G_tfc10 | typeG | accessory | 7.500e-44 | 141.000 | 0.875 | 0.890 |
| NZ_CP022002.1_1701475..1808580_00028 | 79 | G_tfc9 | typeG | accessory | 1.400e-30 | 98.500 | 1.000 | 1.000 |
| NZ_CP022002.1_1701475..1808580_00029 | 112 | G_tfc8 | typeG | accessory | 1.900e-41 | 133.500 | 0.931 | 0.938 |
| NZ_CP022002.1_1701475..1808580_00034 | 248 | G_tfc7 | typeG | accessory | 4.700e-103 | 337.000 | 1.000 | 1.000 |
| NZ_CP022002.1_1701475..1808580_00035 | 743 | t4cp2 | typeG | mandatory | 4.600e-31 | 100.700 | 0.955 | 0.318 |
| NZ_CP022002.1_1701475..1808580_00037 | 166 | G_tfc5 | typeG | accessory | 3.600e-57 | 185.700 | 0.971 | 0.994 |
| NZ_CP022002.1_1701475..1808580_00039 | 251 | G_tfc3 | typeG | accessory | 8.600e-96 | 312.500 | 0.967 | 0.976 |
| NZ_CP022478.1_4249539..4333185_00071 | 250 | G_tfc3 | typeG | accessory | 5.200e-96 | 313.000 | 0.967 | 0.980 |
| NZ_CP022478.1_4249539..4333185_00073 | 166 | G_tfc5 | typeG | accessory | 2.300e-57 | 186.100 | 0.971 | 0.994 |
| NZ_CP022478.1_4249539..4333185_00075 | 743 | t4cp2 | typeG | mandatory | 3.800e-31 | 100.700 | 0.955 | 0.318 |
| NZ_CP022478.1_4249539..4333185_00076 | 248 | G_tfc7 | typeG | accessory | 9.300e-104 | 339.000 | 1.000 | 1.000 |
| NZ_CP022478.1_4249539..4333185_00080 | 112 | G_tfc8 | typeG | accessory | 5.700e-41 | 131.700 | 0.922 | 0.929 |
| NZ_CP022478.1_4249539..4333185_00081 | 79 | G_tfc9 | typeG | accessory | 7.700e-31 | 99.000 | 1.000 | 1.000 |
| NZ_CP022478.1_4249539..4333185_00082 | 118 | G_tfc10 | typeG | accessory | 1.100e-44 | 143.500 | 0.883 | 0.890 |
| NZ_CP022478.1_4249539..4333185_00083 | 128 | G_tfc11 | typeG | accessory | 6.400e-50 | 160.800 | 0.923 | 0.938 |
| NZ_CP022478.1_4249539..4333185_00084 | 219 | G_tfc12 | typeG | accessory | 1.200e-91 | 298.400 | 0.950 | 0.968 |
| NZ_CP022478.1_4249539..4333185_00085 | 294 | G_tfc13 | typeG | accessory | 1.600e-118 | 387.800 | 0.944 | 0.956 |
| NZ_CP022478.1_4249539..4333185_00086 | 501 | G_tfc14 | typeG | accessory | 2.100e-184 | 606.600 | 0.996 | 0.992 |
| NZ_CP022478.1_4249539..4333185_00087 | 147 | G_tfc15 | typeG | accessory | 4.700e-60 | 194.300 | 0.957 | 0.925 |
| NZ_CP022478.1_4249539..4333185_00088 | 980 | virb4 | typeG | mandatory | 2.000e-90 | 297.000 | 0.807 | 0.899 |
| NZ_CP022478.1_4249539..4333185_00092 | 143 | G_tfc24 | typeG | accessory | 2.700e-51 | 165.800 | 0.914 | 0.895 |
| NZ_CP022478.1_4249539..4333185_00093 | 312 | G_tfc23 | typeG | accessory | 3.300e-133 | 436.500 | 0.973 | 0.987 |
| NZ_CP022478.1_4249539..4333185_00094 | 460 | G_tfc22 | typeG | accessory | 4.600e-180 | 591.700 | 0.993 | 0.980 |
| NZ_CP022478.1_4249539..4333185_00095 | 118 | G_tfc18 | typeG | accessory | 2.700e-28 | 90.800 | 0.877 | 0.839 |
| NZ_CP022478.1_4249539..4333185_00096 | 514 | G_tfc19 | typeG | accessory | 1.400e-221 | 729.000 | 0.992 | 0.979 |
| NZ_CP022478.1_4249539..4333185_00100 | 639 | MOBH | typeG | mandatory | 4.200e-44 | 143.200 | 0.868 | 0.268 |
| NZ_CP022525.1_3108007..3190184_00002 | 639 | MOBH | typeG | mandatory | 2.800e-44 | 143.900 | 0.868 | 0.268 |
| NZ_CP022525.1_3108007..3190184_00006 | 504 | G_tfc19 | typeG | accessory | 1.300e-222 | 732.500 | 0.984 | 0.990 |
| NZ_CP022525.1_3108007..3190184_00007 | 115 | G_tfc18 | typeG | accessory | 3.600e-29 | 93.700 | 0.912 | 0.887 |
| NZ_CP022525.1_3108007..3190184_00008 | 460 | G_tfc22 | typeG | accessory | 5.900e-180 | 591.400 | 0.987 | 0.974 |
| NZ_CP022525.1_3108007..3190184_00009 | 312 | G_tfc23 | typeG | accessory | 3.100e-134 | 440.000 | 0.973 | 0.987 |
| NZ_CP022525.1_3108007..3190184_00010 | 143 | G_tfc24 | typeG | accessory | 8.000e-51 | 164.400 | 0.907 | 0.888 |
| NZ_CP022525.1_3108007..3190184_00016 | 980 | virb4 | typeG | mandatory | 1.100e-90 | 297.900 | 0.806 | 0.898 |
| NZ_CP022525.1_3108007..3190184_00017 | 147 | G_tfc15 | typeG | accessory | 3.800e-60 | 194.700 | 0.957 | 0.925 |
| NZ_CP022525.1_3108007..3190184_00018 | 501 | G_tfc14 | typeG | accessory | 1.000e-184 | 607.800 | 0.996 | 0.992 |
| NZ_CP022525.1_3108007..3190184_00019 | 294 | G_tfc13 | typeG | accessory | 1.600e-118 | 387.800 | 0.955 | 0.956 |
| NZ_CP022525.1_3108007..3190184_00020 | 219 | G_tfc12 | typeG | accessory | 7.400e-92 | 299.200 | 0.950 | 0.968 |
| NZ_CP022525.1_3108007..3190184_00021 | 128 | G_tfc11 | typeG | accessory | 4.700e-50 | 161.300 | 0.923 | 0.938 |
| NZ_CP022525.1_3108007..3190184_00022 | 118 | G_tfc10 | typeG | accessory | 6.700e-44 | 141.000 | 0.875 | 0.890 |
| NZ_CP022525.1_3108007..3190184_00023 | 79 | G_tfc9 | typeG | accessory | 1.100e-30 | 98.700 | 1.000 | 1.000 |
| NZ_CP022525.1_3108007..3190184_00024 | 112 | G_tfc8 | typeG | accessory | 1.300e-40 | 130.600 | 0.914 | 0.920 |
| NZ_CP022525.1_3108007..3190184_00030 | 249 | G_tfc7 | typeG | accessory | 9.500e-104 | 339.100 | 1.000 | 0.996 |
| NZ_CP022525.1_3108007..3190184_00031 | 743 | t4cp2 | typeG | mandatory | 4.100e-31 | 100.700 | 0.955 | 0.318 |
| NZ_CP022525.1_3108007..3190184_00033 | 166 | G_tfc5 | typeG | accessory | 2.500e-57 | 186.100 | 0.971 | 0.994 |
| NZ_CP022525.1_3108007..3190184_00035 | 251 | G_tfc3 | typeG | accessory | 3.400e-96 | 313.600 | 0.988 | 0.996 |
| NZ_CP022526.1_2424411..2506587_00074 | 251 | G_tfc3 | typeG | accessory | 3.400e-96 | 313.600 | 0.988 | 0.996 |
| NZ_CP022526.1_2424411..2506587_00076 | 166 | G_tfc5 | typeG | accessory | 2.500e-57 | 186.100 | 0.971 | 0.994 |
| NZ_CP022526.1_2424411..2506587_00078 | 743 | t4cp2 | typeG | mandatory | 4.100e-31 | 100.700 | 0.955 | 0.318 |
| NZ_CP022526.1_2424411..2506587_00079 | 249 | G_tfc7 | typeG | accessory | 9.500e-104 | 339.100 | 1.000 | 0.996 |
| NZ_CP022526.1_2424411..2506587_00085 | 112 | G_tfc8 | typeG | accessory | 1.300e-40 | 130.600 | 0.914 | 0.920 |
| NZ_CP022526.1_2424411..2506587_00086 | 79 | G_tfc9 | typeG | accessory | 1.100e-30 | 98.700 | 1.000 | 1.000 |
| NZ_CP022526.1_2424411..2506587_00087 | 118 | G_tfc10 | typeG | accessory | 6.700e-44 | 141.000 | 0.875 | 0.890 |
| NZ_CP022526.1_2424411..2506587_00088 | 128 | G_tfc11 | typeG | accessory | 4.700e-50 | 161.300 | 0.923 | 0.938 |
| NZ_CP022526.1_2424411..2506587_00089 | 219 | G_tfc12 | typeG | accessory | 7.400e-92 | 299.200 | 0.950 | 0.968 |
| NZ_CP022526.1_2424411..2506587_00090 | 294 | G_tfc13 | typeG | accessory | 1.600e-118 | 387.800 | 0.955 | 0.956 |
| NZ_CP022526.1_2424411..2506587_00091 | 501 | G_tfc14 | typeG | accessory | 1.000e-184 | 607.800 | 0.996 | 0.992 |
| NZ_CP022526.1_2424411..2506587_00092 | 147 | G_tfc15 | typeG | accessory | 3.800e-60 | 194.700 | 0.957 | 0.925 |
| NZ_CP022526.1_2424411..2506587_00093 | 980 | virb4 | typeG | mandatory | 1.100e-90 | 297.900 | 0.806 | 0.898 |
| NZ_CP022526.1_2424411..2506587_00099 | 143 | G_tfc24 | typeG | accessory | 8.000e-51 | 164.400 | 0.907 | 0.888 |
| NZ_CP022526.1_2424411..2506587_00100 | 312 | G_tfc23 | typeG | accessory | 3.100e-134 | 440.000 | 0.973 | 0.987 |
| NZ_CP022526.1_2424411..2506587_00101 | 460 | G_tfc22 | typeG | accessory | 5.900e-180 | 591.400 | 0.987 | 0.974 |
| NZ_CP022526.1_2424411..2506587_00102 | 115 | G_tfc18 | typeG | accessory | 3.600e-29 | 93.700 | 0.912 | 0.887 |
| NZ_CP022526.1_2424411..2506587_00103 | 504 | G_tfc19 | typeG | accessory | 1.300e-222 | 732.500 | 0.984 | 0.990 |
| NZ_CP022526.1_2424411..2506587_00107 | 639 | MOBH | typeG | mandatory | 2.800e-44 | 143.900 | 0.868 | 0.268 |
| NZ_CP023255.1_2762227..2856704_00077 | 251 | G_tfc3 | typeG | accessory | 4.000e-96 | 313.600 | 0.988 | 0.996 |
| NZ_CP023255.1_2762227..2856704_00079 | 166 | G_tfc5 | typeG | accessory | 2.800e-57 | 186.100 | 0.971 | 0.994 |
| NZ_CP023255.1_2762227..2856704_00081 | 743 | t4cp2 | typeG | mandatory | 4.700e-31 | 100.700 | 0.955 | 0.318 |
| NZ_CP023255.1_2762227..2856704_00082 | 249 | G_tfc7 | typeG | accessory | 1.100e-103 | 339.100 | 1.000 | 0.996 |
| NZ_CP023255.1_2762227..2856704_00097 | 112 | G_tfc8 | typeG | accessory | 1.500e-40 | 130.600 | 0.914 | 0.920 |
| NZ_CP023255.1_2762227..2856704_00098 | 79 | G_tfc9 | typeG | accessory | 1.200e-30 | 98.700 | 1.000 | 1.000 |
| NZ_CP023255.1_2762227..2856704_00099 | 118 | G_tfc10 | typeG | accessory | 7.600e-44 | 141.000 | 0.875 | 0.890 |
| NZ_CP023255.1_2762227..2856704_00100 | 128 | G_tfc11 | typeG | accessory | 5.500e-50 | 161.300 | 0.923 | 0.938 |
| NZ_CP023255.1_2762227..2856704_00101 | 219 | G_tfc12 | typeG | accessory | 8.500e-92 | 299.200 | 0.950 | 0.968 |
| NZ_CP023255.1_2762227..2856704_00102 | 294 | G_tfc13 | typeG | accessory | 1.900e-118 | 387.800 | 0.955 | 0.956 |
| NZ_CP023255.1_2762227..2856704_00103 | 501 | G_tfc14 | typeG | accessory | 1.200e-184 | 607.800 | 0.996 | 0.992 |
| NZ_CP023255.1_2762227..2856704_00104 | 147 | G_tfc15 | typeG | accessory | 4.300e-60 | 194.700 | 0.957 | 0.925 |
| NZ_CP023255.1_2762227..2856704_00105 | 980 | virb4 | typeG | mandatory | 4.300e-90 | 296.100 | 0.806 | 0.898 |
| NZ_CP023255.1_2762227..2856704_00111 | 143 | G_tfc24 | typeG | accessory | 9.200e-51 | 164.400 | 0.907 | 0.888 |
| NZ_CP023255.1_2762227..2856704_00112 | 312 | G_tfc23 | typeG | accessory | 3.600e-134 | 440.000 | 0.973 | 0.987 |
| NZ_CP023255.1_2762227..2856704_00113 | 460 | G_tfc22 | typeG | accessory | 5.300e-180 | 591.800 | 0.987 | 0.974 |
| NZ_CP023255.1_2762227..2856704_00114 | 115 | G_tfc18 | typeG | accessory | 4.100e-29 | 93.700 | 0.912 | 0.887 |
| NZ_CP023255.1_2762227..2856704_00115 | 504 | G_tfc19 | typeG | accessory | 1.500e-222 | 732.500 | 0.984 | 0.990 |
| NZ_CP023255.1_2762227..2856704_00123 | 639 | MOBH | typeG | mandatory | 4.500e-44 | 143.400 | 0.868 | 0.268 |
| NZ_CP023316.1_5557899..5656637_00086 | 251 | G_tfc3 | typeG | accessory | 4.900e-96 | 313.200 | 0.988 | 0.996 |
| NZ_CP023316.1_5557899..5656637_00088 | 166 | G_tfc5 | typeG | accessory | 3.600e-57 | 185.600 | 0.977 | 1.000 |
| NZ_CP023316.1_5557899..5656637_00090 | 743 | t4cp2 | typeG | mandatory | 4.100e-31 | 100.800 | 0.955 | 0.318 |
| NZ_CP023316.1_5557899..5656637_00091 | 248 | G_tfc7 | typeG | accessory | 1.700e-103 | 338.400 | 1.000 | 1.000 |
| NZ_CP023316.1_5557899..5656637_00095 | 112 | G_tfc8 | typeG | accessory | 3.000e-41 | 132.800 | 0.922 | 0.929 |
| NZ_CP023316.1_5557899..5656637_00096 | 79 | G_tfc9 | typeG | accessory | 1.000e-30 | 98.800 | 1.000 | 1.000 |
| NZ_CP023316.1_5557899..5656637_00097 | 118 | G_tfc10 | typeG | accessory | 5.200e-45 | 144.700 | 0.925 | 0.924 |
| NZ_CP023316.1_5557899..5656637_00098 | 128 | G_tfc11 | typeG | accessory | 1.100e-49 | 160.100 | 0.923 | 0.938 |
| NZ_CP023316.1_5557899..5656637_00099 | 219 | G_tfc12 | typeG | accessory | 2.700e-91 | 297.400 | 0.950 | 0.968 |
| NZ_CP023316.1_5557899..5656637_00100 | 294 | G_tfc13 | typeG | accessory | 2.600e-118 | 387.200 | 0.941 | 0.956 |
| NZ_CP023316.1_5557899..5656637_00101 | 501 | G_tfc14 | typeG | accessory | 1.100e-184 | 607.800 | 0.996 | 0.992 |
| NZ_CP023316.1_5557899..5656637_00102 | 147 | G_tfc15 | typeG | accessory | 3.900e-60 | 194.700 | 0.957 | 0.925 |
| NZ_CP023316.1_5557899..5656637_00103 | 980 | virb4 | typeG | mandatory | 3.200e-90 | 296.500 | 0.807 | 0.899 |
| NZ_CP023316.1_5557899..5656637_00107 | 143 | G_tfc24 | typeG | accessory | 9.000e-51 | 164.300 | 0.914 | 0.895 |
| NZ_CP023316.1_5557899..5656637_00108 | 312 | G_tfc23 | typeG | accessory | 6.100e-134 | 439.100 | 0.973 | 0.987 |
| NZ_CP023316.1_5557899..5656637_00109 | 460 | G_tfc22 | typeG | accessory | 7.000e-180 | 591.300 | 0.987 | 0.974 |
| NZ_CP023316.1_5557899..5656637_00110 | 115 | G_tfc18 | typeG | accessory | 1.800e-28 | 91.500 | 0.912 | 0.887 |
| NZ_CP023316.1_5557899..5656637_00111 | 514 | G_tfc19 | typeG | accessory | 1.200e-221 | 729.400 | 0.992 | 0.979 |
| NZ_CP023316.1_5557899..5656637_00113 | 639 | MOBH | typeG | mandatory | 2.900e-44 | 143.900 | 0.868 | 0.268 |
| NZ_CP024477.1_5256358..5348471_00077 | 251 | G_tfc3 | typeG | accessory | 3.600e-96 | 313.600 | 0.988 | 0.996 |
| NZ_CP024477.1_5256358..5348471_00079 | 166 | G_tfc5 | typeG | accessory | 1.900e-57 | 186.500 | 0.971 | 0.994 |
| NZ_CP024477.1_5256358..5348471_00081 | 743 | t4cp2 | typeG | mandatory | 4.300e-31 | 100.700 | 0.955 | 0.318 |
| NZ_CP024477.1_5256358..5348471_00082 | 249 | G_tfc7 | typeG | accessory | 9.900e-104 | 339.100 | 1.000 | 0.996 |
| NZ_CP024477.1_5256358..5348471_00088 | 112 | G_tfc8 | typeG | accessory | 7.100e-41 | 131.600 | 0.922 | 0.929 |
| NZ_CP024477.1_5256358..5348471_00089 | 79 | G_tfc9 | typeG | accessory | 4.000e-31 | 100.100 | 1.000 | 1.000 |
| NZ_CP024477.1_5256358..5348471_00090 | 118 | G_tfc10 | typeG | accessory | 3.500e-44 | 142.000 | 0.925 | 0.924 |
| NZ_CP024477.1_5256358..5348471_00091 | 128 | G_tfc11 | typeG | accessory | 6.300e-50 | 160.900 | 0.923 | 0.938 |
| NZ_CP024477.1_5256358..5348471_00092 | 219 | G_tfc12 | typeG | accessory | 1.500e-91 | 298.300 | 0.950 | 0.968 |
| NZ_CP024477.1_5256358..5348471_00093 | 294 | G_tfc13 | typeG | accessory | 5.400e-119 | 389.400 | 0.944 | 0.956 |
| NZ_CP024477.1_5256358..5348471_00094 | 501 | G_tfc14 | typeG | accessory | 1.800e-184 | 607.000 | 0.996 | 0.992 |
| NZ_CP024477.1_5256358..5348471_00095 | 147 | G_tfc15 | typeG | accessory | 3.900e-60 | 194.700 | 0.957 | 0.925 |
| NZ_CP024477.1_5256358..5348471_00096 | 980 | virb4 | typeG | mandatory | 1.700e-89 | 294.000 | 0.806 | 0.898 |
| NZ_CP024477.1_5256358..5348471_00100 | 143 | G_tfc24 | typeG | accessory | 8.800e-51 | 164.300 | 0.914 | 0.895 |
| NZ_CP024477.1_5256358..5348471_00101 | 312 | G_tfc23 | typeG | accessory | 6.100e-134 | 439.100 | 0.973 | 0.987 |
| NZ_CP024477.1_5256358..5348471_00102 | 460 | G_tfc22 | typeG | accessory | 1.300e-179 | 590.300 | 0.989 | 0.976 |
| NZ_CP024477.1_5256358..5348471_00103 | 115 | G_tfc18 | typeG | accessory | 3.900e-29 | 93.600 | 0.912 | 0.887 |
| NZ_CP024477.1_5256358..5348471_00104 | 504 | G_tfc19 | typeG | accessory | 6.300e-223 | 733.600 | 0.984 | 0.990 |
| NZ_CP024477.1_5256358..5348471_00111 | 640 | MOBH | typeG | mandatory | 2.900e-44 | 143.900 | 0.868 | 0.267 |
| NZ_CP024630.1_5489910..5616157_00095 | 251 | G_tfc3 | typeG | accessory | 1.600e-95 | 311.700 | 0.988 | 0.996 |
| NZ_CP024630.1_5489910..5616157_00097 | 166 | G_tfc5 | typeG | accessory | 9.800e-57 | 184.400 | 0.977 | 1.000 |
| NZ_CP024630.1_5489910..5616157_00099 | 743 | t4cp2 | typeG | mandatory | 4.900e-31 | 100.700 | 0.955 | 0.318 |
| NZ_CP024630.1_5489910..5616157_00100 | 248 | G_tfc7 | typeG | accessory | 1.800e-103 | 338.500 | 1.000 | 1.000 |
| NZ_CP024630.1_5489910..5616157_00105 | 112 | G_tfc8 | typeG | accessory | 2.100e-41 | 133.500 | 0.931 | 0.938 |
| NZ_CP024630.1_5489910..5616157_00106 | 79 | G_tfc9 | typeG | accessory | 1.500e-30 | 98.500 | 1.000 | 1.000 |
| NZ_CP024630.1_5489910..5616157_00107 | 118 | G_tfc10 | typeG | accessory | 4.800e-44 | 141.700 | 0.925 | 0.924 |
| NZ_CP024630.1_5489910..5616157_00108 | 128 | G_tfc11 | typeG | accessory | 8.200e-50 | 160.800 | 0.923 | 0.938 |
| NZ_CP024630.1_5489910..5616157_00109 | 219 | G_tfc12 | typeG | accessory | 9.300e-91 | 295.900 | 0.941 | 0.959 |
| NZ_CP024630.1_5489910..5616157_00110 | 294 | G_tfc13 | typeG | accessory | 4.200e-118 | 386.700 | 0.944 | 0.959 |
| NZ_CP024630.1_5489910..5616157_00111 | 501 | G_tfc14 | typeG | accessory | 9.800e-184 | 604.800 | 0.996 | 0.992 |
| NZ_CP024630.1_5489910..5616157_00112 | 147 | G_tfc15 | typeG | accessory | 4.500e-60 | 194.700 | 0.957 | 0.925 |
| NZ_CP024630.1_5489910..5616157_00113 | 980 | virb4 | typeG | mandatory | 1.000e-89 | 295.000 | 0.807 | 0.899 |
| NZ_CP024630.1_5489910..5616157_00118 | 143 | G_tfc24 | typeG | accessory | 9.600e-51 | 164.400 | 0.907 | 0.888 |
| NZ_CP024630.1_5489910..5616157_00119 | 312 | G_tfc23 | typeG | accessory | 8.500e-134 | 438.800 | 0.973 | 0.987 |
| NZ_CP024630.1_5489910..5616157_00120 | 460 | G_tfc22 | typeG | accessory | 4.500e-179 | 588.800 | 0.982 | 0.970 |
| NZ_CP024630.1_5489910..5616157_00121 | 115 | G_tfc18 | typeG | accessory | 3.200e-29 | 94.100 | 0.912 | 0.887 |
| NZ_CP024630.1_5489910..5616157_00122 | 510 | G_tfc19 | typeG | accessory | 1.600e-221 | 729.200 | 0.992 | 0.986 |
| NZ_CP024630.1_5489910..5616157_00129 | 640 | MOBH | typeG | mandatory | 3.400e-44 | 143.900 | 0.868 | 0.267 |
| NZ_CP025229.1_6234155..6327996_00003 | 365 | MOBH | typeG | mandatory | 8.300e-45 | 145.600 | 0.868 | 0.468 |
| NZ_CP025229.1_6234155..6327996_00009 | 503 | G_tfc19 | typeG | accessory | 3.100e-216 | 711.500 | 0.984 | 0.992 |
| NZ_CP025229.1_6234155..6327996_00010 | 118 | G_tfc18 | typeG | accessory | 1.500e-27 | 88.500 | 0.912 | 0.890 |
| NZ_CP025229.1_6234155..6327996_00011 | 460 | G_tfc22 | typeG | accessory | 2.300e-178 | 586.200 | 0.987 | 0.974 |
| NZ_CP025229.1_6234155..6327996_00012 | 312 | G_tfc23 | typeG | accessory | 3.100e-134 | 440.000 | 0.973 | 0.987 |
| NZ_CP025229.1_6234155..6327996_00017 | 980 | virb4 | typeG | mandatory | 5.300e-90 | 295.700 | 0.806 | 0.898 |
| NZ_CP025229.1_6234155..6327996_00018 | 147 | G_tfc15 | typeG | accessory | 3.800e-60 | 194.700 | 0.957 | 0.925 |
| NZ_CP025229.1_6234155..6327996_00019 | 501 | G_tfc14 | typeG | accessory | 1.000e-184 | 607.800 | 0.996 | 0.992 |
| NZ_CP025229.1_6234155..6327996_00020 | 294 | G_tfc13 | typeG | accessory | 2.200e-117 | 384.100 | 0.955 | 0.956 |
| NZ_CP025229.1_6234155..6327996_00021 | 128 | G_tfc11 | typeG | accessory | 4.800e-50 | 161.300 | 0.923 | 0.938 |
| NZ_CP025229.1_6234155..6327996_00022 | 118 | G_tfc10 | typeG | accessory | 6.700e-44 | 141.000 | 0.875 | 0.890 |
| NZ_CP025229.1_6234155..6327996_00023 | 49 | G_tfc9 | typeG | accessory | 7.900e-18 | 57.400 | 0.620 | 1.000 |
| NZ_CP025229.1_6234155..6327996_00024 | 112 | G_tfc8 | typeG | accessory | 1.400e-40 | 130.600 | 0.914 | 0.920 |
| NZ_CP025229.1_6234155..6327996_00037 | 249 | G_tfc7 | typeG | accessory | 1.000e-103 | 339.000 | 1.000 | 0.996 |
| NZ_CP025229.1_6234155..6327996_00038 | 743 | t4cp2 | typeG | mandatory | 4.100e-31 | 100.700 | 0.955 | 0.318 |
| NZ_CP025229.1_6234155..6327996_00040 | 166 | G_tfc5 | typeG | accessory | 2.500e-57 | 186.100 | 0.971 | 0.994 |
| NZ_CP025229.1_6234155..6327996_00042 | 250 | G_tfc3 | typeG | accessory | 5.600e-96 | 313.000 | 0.967 | 0.980 |
| NZ_CP026680.1_5281383..5371346_00032 | 374 | G_tfc2 | typeG | accessory | 1.400e-71 | 232.800 | 0.782 | 0.570 |
| NZ_CP026680.1_5281383..5371346_00061 | 251 | G_tfc3 | typeG | accessory | 3.200e-96 | 313.600 | 0.988 | 0.996 |
| NZ_CP026680.1_5281383..5371346_00063 | 166 | G_tfc5 | typeG | accessory | 3.200e-56 | 182.300 | 0.971 | 0.994 |
| NZ_CP026680.1_5281383..5371346_00065 | 743 | t4cp2 | typeG | mandatory | 3.800e-31 | 100.700 | 0.955 | 0.318 |
| NZ_CP026680.1_5281383..5371346_00066 | 248 | G_tfc7 | typeG | accessory | 8.100e-104 | 339.200 | 1.000 | 1.000 |
| NZ_CP026680.1_5281383..5371346_00071 | 112 | G_tfc8 | typeG | accessory | 6.200e-41 | 131.600 | 0.922 | 0.929 |
| NZ_CP026680.1_5281383..5371346_00072 | 79 | G_tfc9 | typeG | accessory | 3.600e-31 | 100.100 | 1.000 | 1.000 |
| NZ_CP026680.1_5281383..5371346_00073 | 118 | G_tfc10 | typeG | accessory | 3.100e-44 | 142.000 | 0.925 | 0.924 |
| NZ_CP026680.1_5281383..5371346_00074 | 128 | G_tfc11 | typeG | accessory | 3.900e-50 | 161.400 | 0.923 | 0.938 |
| NZ_CP026680.1_5281383..5371346_00075 | 219 | G_tfc12 | typeG | accessory | 1.000e-91 | 298.600 | 0.941 | 0.959 |
| NZ_CP026680.1_5281383..5371346_00076 | 294 | G_tfc13 | typeG | accessory | 3.200e-119 | 390.000 | 0.944 | 0.959 |
| NZ_CP026680.1_5281383..5371346_00077 | 501 | G_tfc14 | typeG | accessory | 9.400e-185 | 607.800 | 0.996 | 0.992 |
| NZ_CP026680.1_5281383..5371346_00078 | 147 | G_tfc15 | typeG | accessory | 2.000e-60 | 195.500 | 0.957 | 0.925 |
| NZ_CP026680.1_5281383..5371346_00079 | 980 | virb4 | typeG | mandatory | 4.100e-90 | 295.900 | 0.807 | 0.899 |
| NZ_CP026680.1_5281383..5371346_00088 | 143 | G_tfc24 | typeG | accessory | 1.500e-50 | 163.300 | 0.914 | 0.895 |
| NZ_CP026680.1_5281383..5371346_00089 | 312 | G_tfc23 | typeG | accessory | 9.800e-134 | 438.200 | 0.973 | 0.987 |
| NZ_CP026680.1_5281383..5371346_00090 | 279 | G_tfc22 | typeG | accessory | 3.000e-105 | 345.100 | 0.558 | 0.910 |
| NZ_CP026680.1_5281383..5371346_00092 | 114 | G_tfc18 | typeG | accessory | 9.300e-28 | 89.000 | 0.912 | 0.886 |
| NZ_CP026680.1_5281383..5371346_00093 | 503 | G_tfc19 | typeG | accessory | 6.600e-222 | 730.100 | 0.984 | 0.992 |
| NZ_CP026680.1_5281383..5371346_00098 | 639 | MOBH | typeG | mandatory | 2.400e-44 | 144.000 | 0.868 | 0.268 |
| NZ_CP027165.1_3947007..4046995_00070 | 251 | G_tfc3 | typeG | accessory | 2.600e-95 | 310.800 | 0.967 | 0.976 |
| NZ_CP027165.1_3947007..4046995_00072 | 166 | G_tfc5 | typeG | accessory | 3.400e-57 | 185.700 | 0.971 | 0.994 |
| NZ_CP027165.1_3947007..4046995_00074 | 743 | t4cp2 | typeG | mandatory | 4.300e-31 | 100.700 | 0.955 | 0.318 |
| NZ_CP027165.1_3947007..4046995_00075 | 248 | G_tfc7 | typeG | accessory | 6.600e-104 | 339.700 | 1.000 | 1.000 |
| NZ_CP027165.1_3947007..4046995_00081 | 112 | G_tfc8 | typeG | accessory | 6.500e-41 | 131.700 | 0.922 | 0.929 |
| NZ_CP027165.1_3947007..4046995_00082 | 79 | G_tfc9 | typeG | accessory | 8.700e-31 | 99.000 | 1.000 | 1.000 |
| NZ_CP027165.1_3947007..4046995_00083 | 118 | G_tfc10 | typeG | accessory | 3.000e-44 | 142.200 | 0.875 | 0.890 |
| NZ_CP027165.1_3947007..4046995_00084 | 128 | G_tfc11 | typeG | accessory | 8.600e-50 | 160.500 | 0.923 | 0.938 |
| NZ_CP027165.1_3947007..4046995_00085 | 219 | G_tfc12 | typeG | accessory | 6.400e-91 | 296.200 | 0.941 | 0.959 |
| NZ_CP027165.1_3947007..4046995_00086 | 294 | G_tfc13 | typeG | accessory | 7.500e-119 | 389.000 | 0.944 | 0.959 |
| NZ_CP027165.1_3947007..4046995_00087 | 501 | G_tfc14 | typeG | accessory | 1.400e-184 | 607.400 | 0.996 | 0.992 |
| NZ_CP027165.1_3947007..4046995_00088 | 147 | G_tfc15 | typeG | accessory | 7.200e-60 | 193.900 | 0.957 | 0.925 |
| NZ_CP027165.1_3947007..4046995_00089 | 980 | virb4 | typeG | mandatory | 2.200e-89 | 293.700 | 0.807 | 0.899 |
| NZ_CP027165.1_3947007..4046995_00096 | 143 | G_tfc24 | typeG | accessory | 8.900e-51 | 164.300 | 0.914 | 0.895 |
| NZ_CP027165.1_3947007..4046995_00097 | 312 | G_tfc23 | typeG | accessory | 3.400e-134 | 439.900 | 0.973 | 0.987 |
| NZ_CP027165.1_3947007..4046995_00098 | 460 | G_tfc22 | typeG | accessory | 8.700e-180 | 591.000 | 0.987 | 0.974 |
| NZ_CP027165.1_3947007..4046995_00099 | 115 | G_tfc18 | typeG | accessory | 9.600e-29 | 92.400 | 0.912 | 0.887 |
| NZ_CP027165.1_3947007..4046995_00100 | 511 | G_tfc19 | typeG | accessory | 1.200e-222 | 732.700 | 0.990 | 0.982 |
| NZ_CP027165.1_3947007..4046995_00113 | 639 | MOBH | typeG | mandatory | 3.000e-44 | 143.900 | 0.873 | 0.271 |
| NZ_CP027166.1_3795236..3886516_00002 | 639 | MOBH | typeG | mandatory | 1.200e-44 | 145.000 | 0.868 | 0.268 |
| NZ_CP027166.1_3795236..3886516_00009 | 504 | G_tfc19 | typeG | accessory | 1.800e-222 | 732.000 | 0.988 | 0.994 |
| NZ_CP027166.1_3795236..3886516_00010 | 115 | G_tfc18 | typeG | accessory | 3.500e-29 | 93.700 | 0.912 | 0.887 |
| NZ_CP027166.1_3795236..3886516_00011 | 460 | G_tfc22 | typeG | accessory | 5.800e-180 | 591.400 | 0.987 | 0.974 |
| NZ_CP027166.1_3795236..3886516_00012 | 312 | G_tfc23 | typeG | accessory | 2.200e-134 | 440.400 | 0.973 | 0.987 |
| NZ_CP027166.1_3795236..3886516_00013 | 143 | G_tfc24 | typeG | accessory | 2.700e-51 | 165.900 | 0.914 | 0.895 |
| NZ_CP027166.1_3795236..3886516_00019 | 980 | virb4 | typeG | mandatory | 2.900e-90 | 296.500 | 0.806 | 0.898 |
| NZ_CP027166.1_3795236..3886516_00020 | 147 | G_tfc15 | typeG | accessory | 1.200e-58 | 189.900 | 0.957 | 0.925 |
| NZ_CP027166.1_3795236..3886516_00021 | 501 | G_tfc14 | typeG | accessory | 1.700e-184 | 607.000 | 0.996 | 0.992 |
| NZ_CP027166.1_3795236..3886516_00022 | 294 | G_tfc13 | typeG | accessory | 5.100e-119 | 389.400 | 0.944 | 0.956 |
| NZ_CP027166.1_3795236..3886516_00023 | 219 | G_tfc12 | typeG | accessory | 1.400e-91 | 298.300 | 0.950 | 0.968 |
| NZ_CP027166.1_3795236..3886516_00024 | 128 | G_tfc11 | typeG | accessory | 5.900e-50 | 160.900 | 0.923 | 0.938 |
| NZ_CP027166.1_3795236..3886516_00025 | 118 | G_tfc10 | typeG | accessory | 3.300e-44 | 142.000 | 0.925 | 0.924 |
| NZ_CP027166.1_3795236..3886516_00026 | 79 | G_tfc9 | typeG | accessory | 3.800e-31 | 100.100 | 1.000 | 1.000 |
| NZ_CP027166.1_3795236..3886516_00027 | 112 | G_tfc8 | typeG | accessory | 1.800e-40 | 130.200 | 0.922 | 0.929 |
| NZ_CP027166.1_3795236..3886516_00033 | 248 | G_tfc7 | typeG | accessory | 8.700e-104 | 339.200 | 1.000 | 1.000 |
| NZ_CP027166.1_3795236..3886516_00034 | 743 | t4cp2 | typeG | mandatory | 4.000e-31 | 100.700 | 0.955 | 0.318 |
| NZ_CP027166.1_3795236..3886516_00036 | 166 | G_tfc5 | typeG | accessory | 3.400e-56 | 182.300 | 0.971 | 0.994 |
| NZ_CP027166.1_3795236..3886516_00038 | 251 | G_tfc3 | typeG | accessory | 3.400e-96 | 313.600 | 0.988 | 0.996 |
| NZ_CP027171.1_3284186..3383318_00091 | 251 | G_tfc3 | typeG | accessory | 4.000e-96 | 313.600 | 0.988 | 0.996 |
| NZ_CP027171.1_3284186..3383318_00093 | 166 | G_tfc5 | typeG | accessory | 2.800e-57 | 186.100 | 0.971 | 0.994 |
| NZ_CP027171.1_3284186..3383318_00095 | 743 | t4cp2 | typeG | mandatory | 1.300e-30 | 99.300 | 0.955 | 0.318 |
| NZ_CP027171.1_3284186..3383318_00096 | 248 | G_tfc7 | typeG | accessory | 3.000e-103 | 337.600 | 1.000 | 1.000 |
| NZ_CP027171.1_3284186..3383318_00101 | 112 | G_tfc8 | typeG | accessory | 7.000e-41 | 131.700 | 0.922 | 0.929 |
| NZ_CP027171.1_3284186..3383318_00102 | 79 | G_tfc9 | typeG | accessory | 9.400e-31 | 99.000 | 1.000 | 1.000 |
| NZ_CP027171.1_3284186..3383318_00103 | 118 | G_tfc10 | typeG | accessory | 3.200e-44 | 142.200 | 0.875 | 0.890 |
| NZ_CP027171.1_3284186..3383318_00104 | 128 | G_tfc11 | typeG | accessory | 9.400e-50 | 160.500 | 0.923 | 0.938 |
| NZ_CP027171.1_3284186..3383318_00105 | 219 | G_tfc12 | typeG | accessory | 6.900e-91 | 296.200 | 0.941 | 0.959 |
| NZ_CP027171.1_3284186..3383318_00106 | 294 | G_tfc13 | typeG | accessory | 8.100e-117 | 382.400 | 0.941 | 0.952 |
| NZ_CP027171.1_3284186..3383318_00107 | 501 | G_tfc14 | typeG | accessory | 8.100e-185 | 608.300 | 0.996 | 0.992 |
| NZ_CP027171.1_3284186..3383318_00108 | 147 | G_tfc15 | typeG | accessory | 3.900e-60 | 194.900 | 0.957 | 0.925 |
| NZ_CP027171.1_3284186..3383318_00109 | 980 | virb4 | typeG | mandatory | 6.800e-91 | 298.800 | 0.808 | 0.900 |
| NZ_CP027171.1_3284186..3383318_00115 | 143 | G_tfc24 | typeG | accessory | 3.900e-51 | 165.500 | 0.914 | 0.895 |
| NZ_CP027171.1_3284186..3383318_00116 | 321 | G_tfc23 | typeG | accessory | 5.700e-134 | 439.300 | 0.973 | 0.960 |
| NZ_CP027171.1_3284186..3383318_00117 | 460 | G_tfc22 | typeG | accessory | 6.100e-180 | 591.600 | 0.987 | 0.974 |
| NZ_CP027171.1_3284186..3383318_00118 | 115 | G_tfc18 | typeG | accessory | 4.300e-29 | 93.600 | 0.912 | 0.887 |
| NZ_CP027171.1_3284186..3383318_00119 | 503 | G_tfc19 | typeG | accessory | 8.000e-222 | 730.100 | 0.984 | 0.992 |
| NZ_CP027171.1_3284186..3383318_00123 | 639 | MOBH | typeG | mandatory | 3.200e-44 | 143.900 | 0.868 | 0.268 |
| NZ_CP027172.1_1359551..1445795_00002 | 640 | MOBH | typeG | mandatory | 2.600e-44 | 143.900 | 0.868 | 0.267 |
| NZ_CP027172.1_1359551..1445795_00009 | 511 | G_tfc19 | typeG | accessory | 1.100e-222 | 732.700 | 0.990 | 0.982 |
| NZ_CP027172.1_1359551..1445795_00010 | 115 | G_tfc18 | typeG | accessory | 8.500e-29 | 92.400 | 0.912 | 0.887 |
| NZ_CP027172.1_1359551..1445795_00011 | 460 | G_tfc22 | typeG | accessory | 1.500e-179 | 590.000 | 0.987 | 0.974 |
| NZ_CP027172.1_1359551..1445795_00012 | 312 | G_tfc23 | typeG | accessory | 2.900e-134 | 440.000 | 0.973 | 0.987 |
| NZ_CP027172.1_1359551..1445795_00013 | 143 | G_tfc24 | typeG | accessory | 7.500e-51 | 164.400 | 0.907 | 0.888 |
| NZ_CP027172.1_1359551..1445795_00018 | 980 | virb4 | typeG | mandatory | 1.100e-90 | 297.900 | 0.806 | 0.898 |
| NZ_CP027172.1_1359551..1445795_00019 | 147 | G_tfc15 | typeG | accessory | 3.500e-60 | 194.700 | 0.957 | 0.925 |
| NZ_CP027172.1_1359551..1445795_00020 | 501 | G_tfc14 | typeG | accessory | 7.600e-184 | 604.800 | 0.996 | 0.992 |
| NZ_CP027172.1_1359551..1445795_00021 | 294 | G_tfc13 | typeG | accessory | 3.300e-118 | 386.700 | 0.944 | 0.959 |
| NZ_CP027172.1_1359551..1445795_00022 | 219 | G_tfc12 | typeG | accessory | 7.200e-91 | 295.900 | 0.941 | 0.959 |
| NZ_CP027172.1_1359551..1445795_00023 | 128 | G_tfc11 | typeG | accessory | 6.400e-50 | 160.800 | 0.923 | 0.938 |
| NZ_CP027172.1_1359551..1445795_00024 | 118 | G_tfc10 | typeG | accessory | 7.100e-44 | 140.900 | 0.875 | 0.890 |
| NZ_CP027172.1_1359551..1445795_00025 | 79 | G_tfc9 | typeG | accessory | 9.000e-31 | 98.800 | 1.000 | 1.000 |
| NZ_CP027172.1_1359551..1445795_00026 | 112 | G_tfc8 | typeG | accessory | 3.600e-41 | 132.400 | 0.931 | 0.938 |
| NZ_CP027172.1_1359551..1445795_00031 | 248 | G_tfc7 | typeG | accessory | 8.700e-104 | 339.100 | 1.000 | 1.000 |
| NZ_CP027172.1_1359551..1445795_00032 | 743 | t4cp2 | typeG | mandatory | 3.800e-31 | 100.700 | 0.955 | 0.318 |
| NZ_CP027172.1_1359551..1445795_00034 | 166 | G_tfc5 | typeG | accessory | 2.400e-57 | 186.000 | 0.971 | 0.994 |
| NZ_CP027172.1_1359551..1445795_00036 | 250 | G_tfc3 | typeG | accessory | 5.200e-96 | 313.000 | 0.967 | 0.980 |
| NZ_CP027172.1_1359551..1445795_00067 | 374 | G_tfc2 | typeG | accessory | 5.700e-73 | 237.400 | 0.911 | 0.701 |
| NZ_CP027174.1_3429923..3519029_00037 | 374 | G_tfc2 | typeG | accessory | 2.000e-71 | 232.400 | 0.782 | 0.570 |
| NZ_CP027174.1_3429923..3519029_00068 | 251 | G_tfc3 | typeG | accessory | 3.300e-96 | 313.600 | 0.988 | 0.996 |
| NZ_CP027174.1_3429923..3519029_00070 | 166 | G_tfc5 | typeG | accessory | 2.300e-57 | 186.100 | 0.971 | 0.994 |
| NZ_CP027174.1_3429923..3519029_00072 | 743 | t4cp2 | typeG | mandatory | 3.900e-31 | 100.700 | 0.955 | 0.318 |
| NZ_CP027174.1_3429923..3519029_00073 | 249 | G_tfc7 | typeG | accessory | 1.400e-103 | 338.500 | 1.000 | 0.996 |
| NZ_CP027174.1_3429923..3519029_00078 | 112 | G_tfc8 | typeG | accessory | 1.300e-40 | 130.600 | 0.914 | 0.920 |
| NZ_CP027174.1_3429923..3519029_00079 | 49 | G_tfc9 | typeG | accessory | 7.500e-18 | 57.400 | 0.620 | 1.000 |
| NZ_CP027174.1_3429923..3519029_00080 | 118 | G_tfc10 | typeG | accessory | 6.400e-44 | 141.000 | 0.875 | 0.890 |
| NZ_CP027174.1_3429923..3519029_00081 | 128 | G_tfc11 | typeG | accessory | 4.500e-50 | 161.300 | 0.923 | 0.938 |
| NZ_CP027174.1_3429923..3519029_00082 | 219 | G_tfc12 | typeG | accessory | 7.100e-92 | 299.200 | 0.950 | 0.968 |
| NZ_CP027174.1_3429923..3519029_00083 | 294 | G_tfc13 | typeG | accessory | 1.500e-118 | 387.800 | 0.955 | 0.956 |
| NZ_CP027174.1_3429923..3519029_00084 | 501 | G_tfc14 | typeG | accessory | 9.800e-185 | 607.800 | 0.996 | 0.992 |
| NZ_CP027174.1_3429923..3519029_00085 | 147 | G_tfc15 | typeG | accessory | 3.400e-60 | 194.800 | 0.957 | 0.925 |
| NZ_CP027174.1_3429923..3519029_00086 | 980 | virb4 | typeG | mandatory | 8.200e-90 | 295.000 | 0.807 | 0.899 |
| NZ_CP027174.1_3429923..3519029_00091 | 143 | G_tfc24 | typeG | accessory | 3.900e-51 | 165.300 | 0.914 | 0.895 |
| NZ_CP027174.1_3429923..3519029_00092 | 312 | G_tfc23 | typeG | accessory | 9.600e-134 | 438.300 | 0.973 | 0.987 |
| NZ_CP027174.1_3429923..3519029_00093 | 462 | G_tfc22 | typeG | accessory | 4.100e-180 | 591.900 | 0.987 | 0.970 |
| NZ_CP027174.1_3429923..3519029_00094 | 115 | G_tfc18 | typeG | accessory | 3.200e-29 | 93.800 | 0.912 | 0.887 |
| NZ_CP027174.1_3429923..3519029_00095 | 508 | G_tfc19 | typeG | accessory | 1.100e-220 | 726.100 | 0.992 | 0.990 |
| NZ_CP027174.1_3429923..3519029_00102 | 640 | MOBH | typeG | mandatory | 2.700e-44 | 143.900 | 0.868 | 0.267 |
| NZ_CP028162.1_4865466..4953317_00077 | 251 | G_tfc3 | typeG | accessory | 3.700e-96 | 313.600 | 0.988 | 0.996 |
| NZ_CP028162.1_4865466..4953317_00079 | 166 | G_tfc5 | typeG | accessory | 2.600e-57 | 186.100 | 0.971 | 0.994 |
| NZ_CP028162.1_4865466..4953317_00081 | 743 | t4cp2 | typeG | mandatory | 4.400e-31 | 100.700 | 0.955 | 0.318 |
| NZ_CP028162.1_4865466..4953317_00082 | 249 | G_tfc7 | typeG | accessory | 1.000e-103 | 339.100 | 1.000 | 0.996 |
| NZ_CP028162.1_4865466..4953317_00088 | 112 | G_tfc8 | typeG | accessory | 1.400e-40 | 130.600 | 0.914 | 0.920 |
| NZ_CP028162.1_4865466..4953317_00089 | 79 | G_tfc9 | typeG | accessory | 1.100e-30 | 98.700 | 1.000 | 1.000 |
| NZ_CP028162.1_4865466..4953317_00090 | 118 | G_tfc10 | typeG | accessory | 7.100e-44 | 141.000 | 0.875 | 0.890 |
| NZ_CP028162.1_4865466..4953317_00091 | 128 | G_tfc11 | typeG | accessory | 5.100e-50 | 161.300 | 0.923 | 0.938 |
| NZ_CP028162.1_4865466..4953317_00092 | 219 | G_tfc12 | typeG | accessory | 7.900e-92 | 299.200 | 0.950 | 0.968 |
| NZ_CP028162.1_4865466..4953317_00093 | 294 | G_tfc13 | typeG | accessory | 5.500e-119 | 389.400 | 0.955 | 0.956 |
| NZ_CP028162.1_4865466..4953317_00094 | 501 | G_tfc14 | typeG | accessory | 1.600e-184 | 607.300 | 0.990 | 0.984 |
| NZ_CP028162.1_4865466..4953317_00095 | 147 | G_tfc15 | typeG | accessory | 1.100e-60 | 196.600 | 0.957 | 0.925 |
| NZ_CP028162.1_4865466..4953317_00096 | 980 | virb4 | typeG | mandatory | 1.700e-90 | 297.400 | 0.807 | 0.899 |
| NZ_CP028162.1_4865466..4953317_00102 | 143 | G_tfc24 | typeG | accessory | 5.900e-51 | 164.900 | 0.914 | 0.895 |
| NZ_CP028162.1_4865466..4953317_00103 | 312 | G_tfc23 | typeG | accessory | 2.400e-134 | 440.400 | 0.973 | 0.987 |
| NZ_CP028162.1_4865466..4953317_00104 | 460 | G_tfc22 | typeG | accessory | 5.700e-180 | 591.600 | 0.987 | 0.974 |
| NZ_CP028162.1_4865466..4953317_00105 | 115 | G_tfc18 | typeG | accessory | 4.000e-29 | 93.600 | 0.912 | 0.887 |
| NZ_CP028162.1_4865466..4953317_00106 | 503 | G_tfc19 | typeG | accessory | 7.400e-222 | 730.100 | 0.984 | 0.992 |
| NZ_CP028162.1_4865466..4953317_00114 | 639 | MOBH | typeG | mandatory | 7.400e-44 | 142.600 | 0.868 | 0.268 |
| NZ_CP028584.2_5567129..5674073_00081 | 251 | G_tfc3 | typeG | accessory | 8.200e-96 | 312.500 | 0.967 | 0.976 |
| NZ_CP028584.2_5567129..5674073_00083 | 166 | G_tfc5 | typeG | accessory | 3.500e-57 | 185.700 | 0.971 | 0.994 |
| NZ_CP028584.2_5567129..5674073_00085 | 743 | t4cp2 | typeG | mandatory | 4.400e-31 | 100.700 | 0.955 | 0.318 |
| NZ_CP028584.2_5567129..5674073_00086 | 248 | G_tfc7 | typeG | accessory | 4.500e-103 | 337.000 | 1.000 | 1.000 |
| NZ_CP028584.2_5567129..5674073_00091 | 112 | G_tfc8 | typeG | accessory | 1.900e-41 | 133.500 | 0.931 | 0.938 |
| NZ_CP028584.2_5567129..5674073_00092 | 79 | G_tfc9 | typeG | accessory | 1.300e-30 | 98.500 | 1.000 | 1.000 |
| NZ_CP028584.2_5567129..5674073_00093 | 118 | G_tfc10 | typeG | accessory | 7.200e-44 | 141.000 | 0.875 | 0.890 |
| NZ_CP028584.2_5567129..5674073_00094 | 128 | G_tfc11 | typeG | accessory | 5.100e-50 | 161.300 | 0.923 | 0.938 |
| NZ_CP028584.2_5567129..5674073_00095 | 219 | G_tfc12 | typeG | accessory | 8.100e-92 | 299.200 | 0.950 | 0.968 |
| NZ_CP028584.2_5567129..5674073_00096 | 294 | G_tfc13 | typeG | accessory | 3.900e-118 | 386.700 | 0.955 | 0.956 |
| NZ_CP028584.2_5567129..5674073_00097 | 501 | G_tfc14 | typeG | accessory | 1.000e-183 | 604.600 | 0.996 | 0.992 |
| NZ_CP028584.2_5567129..5674073_00098 | 147 | G_tfc15 | typeG | accessory | 4.100e-60 | 194.700 | 0.957 | 0.925 |
| NZ_CP028584.2_5567129..5674073_00099 | 980 | virb4 | typeG | mandatory | 1.200e-90 | 297.900 | 0.806 | 0.898 |
| NZ_CP028584.2_5567129..5674073_00104 | 143 | G_tfc24 | typeG | accessory | 8.700e-51 | 164.400 | 0.907 | 0.888 |
| NZ_CP028584.2_5567129..5674073_00105 | 312 | G_tfc23 | typeG | accessory | 8.700e-134 | 438.600 | 0.973 | 0.987 |
| NZ_CP028584.2_5567129..5674073_00106 | 462 | G_tfc22 | typeG | accessory | 5.100e-180 | 591.800 | 0.987 | 0.970 |
| NZ_CP028584.2_5567129..5674073_00107 | 115 | G_tfc18 | typeG | accessory | 3.700e-29 | 93.800 | 0.912 | 0.887 |
| NZ_CP028584.2_5567129..5674073_00108 | 511 | G_tfc19 | typeG | accessory | 6.600e-223 | 733.600 | 0.990 | 0.982 |
| NZ_CP028584.2_5567129..5674073_00116 | 639 | MOBH | typeG | mandatory | 4.400e-44 | 143.400 | 0.868 | 0.268 |
| NZ_CP028848.1_5164794..5276868_00071 | 251 | G_tfc3 | typeG | accessory | 3.500e-95 | 310.500 | 0.988 | 0.996 |
| NZ_CP028848.1_5164794..5276868_00073 | 166 | G_tfc5 | typeG | accessory | 1.700e-56 | 183.500 | 0.971 | 0.994 |
| NZ_CP028848.1_5164794..5276868_00075 | 743 | t4cp2 | typeG | mandatory | 4.600e-31 | 100.700 | 0.955 | 0.318 |
| NZ_CP028848.1_5164794..5276868_00076 | 248 | G_tfc7 | typeG | accessory | 1.400e-103 | 338.700 | 1.000 | 1.000 |
| NZ_CP028848.1_5164794..5276868_00079 | 112 | G_tfc8 | typeG | accessory | 1.100e-40 | 131.000 | 0.922 | 0.929 |
| NZ_CP028848.1_5164794..5276868_00080 | 79 | G_tfc9 | typeG | accessory | 9.200e-31 | 99.000 | 1.000 | 1.000 |
| NZ_CP028848.1_5164794..5276868_00081 | 118 | G_tfc10 | typeG | accessory | 7.500e-45 | 144.200 | 0.900 | 0.898 |
| NZ_CP028848.1_5164794..5276868_00082 | 128 | G_tfc11 | typeG | accessory | 6.800e-50 | 160.900 | 0.923 | 0.938 |
| NZ_CP028848.1_5164794..5276868_00083 | 219 | G_tfc12 | typeG | accessory | 1.700e-91 | 298.200 | 0.950 | 0.968 |
| NZ_CP028848.1_5164794..5276868_00084 | 294 | G_tfc13 | typeG | accessory | 2.400e-118 | 387.400 | 0.955 | 0.956 |
| NZ_CP028848.1_5164794..5276868_00085 | 501 | G_tfc14 | typeG | accessory | 7.400e-185 | 608.400 | 0.996 | 0.992 |
| NZ_CP028848.1_5164794..5276868_00086 | 147 | G_tfc15 | typeG | accessory | 1.500e-59 | 192.900 | 0.957 | 0.925 |
| NZ_CP028848.1_5164794..5276868_00087 | 982 | virb4 | typeG | mandatory | 6.800e-89 | 292.200 | 0.806 | 0.898 |
| NZ_CP028848.1_5164794..5276868_00104 | 143 | G_tfc24 | typeG | accessory | 9.400e-51 | 164.300 | 0.914 | 0.895 |
| NZ_CP028848.1_5164794..5276868_00105 | 312 | G_tfc23 | typeG | accessory | 3.600e-134 | 439.900 | 0.973 | 0.987 |
| NZ_CP028848.1_5164794..5276868_00106 | 460 | G_tfc22 | typeG | accessory | 9.200e-180 | 591.000 | 0.987 | 0.974 |
| NZ_CP028848.1_5164794..5276868_00107 | 115 | G_tfc18 | typeG | accessory | 1.000e-28 | 92.400 | 0.912 | 0.887 |
| NZ_CP028848.1_5164794..5276868_00108 | 516 | G_tfc19 | typeG | accessory | 9.000e-222 | 729.900 | 0.988 | 0.971 |
| NZ_CP028848.1_5164794..5276868_00120 | 639 | MOBH | typeG | mandatory | 3.200e-44 | 143.900 | 0.873 | 0.271 |
| NZ_CP028849.1_5164090..5276164_00071 | 251 | G_tfc3 | typeG | accessory | 3.500e-95 | 310.500 | 0.988 | 0.996 |
| NZ_CP028849.1_5164090..5276164_00073 | 166 | G_tfc5 | typeG | accessory | 1.700e-56 | 183.500 | 0.971 | 0.994 |
| NZ_CP028849.1_5164090..5276164_00075 | 743 | t4cp2 | typeG | mandatory | 4.600e-31 | 100.700 | 0.955 | 0.318 |
| NZ_CP028849.1_5164090..5276164_00076 | 248 | G_tfc7 | typeG | accessory | 1.400e-103 | 338.700 | 1.000 | 1.000 |
| NZ_CP028849.1_5164090..5276164_00079 | 112 | G_tfc8 | typeG | accessory | 1.100e-40 | 131.000 | 0.922 | 0.929 |
| NZ_CP028849.1_5164090..5276164_00080 | 79 | G_tfc9 | typeG | accessory | 9.200e-31 | 99.000 | 1.000 | 1.000 |
| NZ_CP028849.1_5164090..5276164_00081 | 118 | G_tfc10 | typeG | accessory | 7.500e-45 | 144.200 | 0.900 | 0.898 |
| NZ_CP028849.1_5164090..5276164_00082 | 128 | G_tfc11 | typeG | accessory | 6.800e-50 | 160.900 | 0.923 | 0.938 |
| NZ_CP028849.1_5164090..5276164_00083 | 219 | G_tfc12 | typeG | accessory | 1.700e-91 | 298.200 | 0.950 | 0.968 |
| NZ_CP028849.1_5164090..5276164_00084 | 294 | G_tfc13 | typeG | accessory | 2.400e-118 | 387.400 | 0.955 | 0.956 |
| NZ_CP028849.1_5164090..5276164_00085 | 501 | G_tfc14 | typeG | accessory | 7.400e-185 | 608.400 | 0.996 | 0.992 |
| NZ_CP028849.1_5164090..5276164_00086 | 147 | G_tfc15 | typeG | accessory | 1.500e-59 | 192.900 | 0.957 | 0.925 |
| NZ_CP028849.1_5164090..5276164_00087 | 982 | virb4 | typeG | mandatory | 6.800e-89 | 292.200 | 0.806 | 0.898 |
| NZ_CP028849.1_5164090..5276164_00104 | 143 | G_tfc24 | typeG | accessory | 9.400e-51 | 164.300 | 0.914 | 0.895 |
| NZ_CP028849.1_5164090..5276164_00105 | 312 | G_tfc23 | typeG | accessory | 3.600e-134 | 439.900 | 0.973 | 0.987 |
| NZ_CP028849.1_5164090..5276164_00106 | 460 | G_tfc22 | typeG | accessory | 9.200e-180 | 591.000 | 0.987 | 0.974 |
| NZ_CP028849.1_5164090..5276164_00107 | 115 | G_tfc18 | typeG | accessory | 1.000e-28 | 92.400 | 0.912 | 0.887 |
| NZ_CP028849.1_5164090..5276164_00108 | 516 | G_tfc19 | typeG | accessory | 9.000e-222 | 729.900 | 0.988 | 0.971 |
| NZ_CP028849.1_5164090..5276164_00120 | 639 | MOBH | typeG | mandatory | 3.200e-44 | 143.900 | 0.873 | 0.271 |
| NZ_CP028917.1_1699886..1790283_00002 | 639 | MOBH | typeG | mandatory | 2.500e-44 | 144.000 | 0.868 | 0.268 |
| NZ_CP028917.1_1699886..1790283_00008 | 503 | G_tfc19 | typeG | accessory | 7.000e-222 | 730.100 | 0.984 | 0.992 |
| NZ_CP028917.1_1699886..1790283_00009 | 114 | G_tfc18 | typeG | accessory | 9.900e-28 | 89.000 | 0.912 | 0.886 |
| NZ_CP028917.1_1699886..1790283_00010 | 462 | G_tfc22 | typeG | accessory | 6.400e-180 | 591.300 | 0.987 | 0.970 |
| NZ_CP028917.1_1699886..1790283_00011 | 312 | G_tfc23 | typeG | accessory | 1.000e-133 | 438.200 | 0.973 | 0.987 |
| NZ_CP028917.1_1699886..1790283_00012 | 143 | G_tfc24 | typeG | accessory | 1.600e-50 | 163.300 | 0.914 | 0.895 |
| NZ_CP028917.1_1699886..1790283_00022 | 980 | virb4 | typeG | mandatory | 4.300e-90 | 295.900 | 0.807 | 0.899 |
| NZ_CP028917.1_1699886..1790283_00023 | 147 | G_tfc15 | typeG | accessory | 2.100e-60 | 195.500 | 0.957 | 0.925 |
| NZ_CP028917.1_1699886..1790283_00024 | 501 | G_tfc14 | typeG | accessory | 1.000e-184 | 607.800 | 0.996 | 0.992 |
| NZ_CP028917.1_1699886..1790283_00025 | 294 | G_tfc13 | typeG | accessory | 3.400e-119 | 390.000 | 0.944 | 0.959 |
| NZ_CP028917.1_1699886..1790283_00026 | 219 | G_tfc12 | typeG | accessory | 1.100e-91 | 298.600 | 0.941 | 0.959 |
| NZ_CP028917.1_1699886..1790283_00027 | 128 | G_tfc11 | typeG | accessory | 4.200e-50 | 161.400 | 0.923 | 0.938 |
| NZ_CP028917.1_1699886..1790283_00028 | 118 | G_tfc10 | typeG | accessory | 2.200e-42 | 136.100 | 0.933 | 0.932 |
| NZ_CP028917.1_1699886..1790283_00029 | 79 | G_tfc9 | typeG | accessory | 8.000e-31 | 99.000 | 1.000 | 1.000 |
| NZ_CP028917.1_1699886..1790283_00030 | 112 | G_tfc8 | typeG | accessory | 1.700e-41 | 133.500 | 0.931 | 0.938 |
| NZ_CP028917.1_1699886..1790283_00035 | 248 | G_tfc7 | typeG | accessory | 3.400e-104 | 340.500 | 1.000 | 1.000 |
| NZ_CP028917.1_1699886..1790283_00036 | 743 | t4cp2 | typeG | mandatory | 4.000e-31 | 100.700 | 0.955 | 0.318 |
| NZ_CP028917.1_1699886..1790283_00038 | 166 | G_tfc5 | typeG | accessory | 7.900e-57 | 184.400 | 0.977 | 1.000 |
| NZ_CP028917.1_1699886..1790283_00040 | 251 | G_tfc3 | typeG | accessory | 3.700e-96 | 313.500 | 0.988 | 0.996 |
| NZ_CP028917.1_1699886..1790283_00071 | 374 | G_tfc2 | typeG | accessory | 1.400e-71 | 232.800 | 0.782 | 0.570 |
| NZ_CP028959.1_5179005..5291079_00071 | 251 | G_tfc3 | typeG | accessory | 3.500e-95 | 310.500 | 0.988 | 0.996 |
| NZ_CP028959.1_5179005..5291079_00073 | 166 | G_tfc5 | typeG | accessory | 1.700e-56 | 183.500 | 0.971 | 0.994 |
| NZ_CP028959.1_5179005..5291079_00075 | 743 | t4cp2 | typeG | mandatory | 4.600e-31 | 100.700 | 0.955 | 0.318 |
| NZ_CP028959.1_5179005..5291079_00076 | 248 | G_tfc7 | typeG | accessory | 1.400e-103 | 338.700 | 1.000 | 1.000 |
| NZ_CP028959.1_5179005..5291079_00079 | 112 | G_tfc8 | typeG | accessory | 1.100e-40 | 131.000 | 0.922 | 0.929 |
| NZ_CP028959.1_5179005..5291079_00080 | 79 | G_tfc9 | typeG | accessory | 9.200e-31 | 99.000 | 1.000 | 1.000 |
| NZ_CP028959.1_5179005..5291079_00081 | 118 | G_tfc10 | typeG | accessory | 7.500e-45 | 144.200 | 0.900 | 0.898 |
| NZ_CP028959.1_5179005..5291079_00082 | 128 | G_tfc11 | typeG | accessory | 6.800e-50 | 160.900 | 0.923 | 0.938 |
| NZ_CP028959.1_5179005..5291079_00083 | 219 | G_tfc12 | typeG | accessory | 1.700e-91 | 298.200 | 0.950 | 0.968 |
| NZ_CP028959.1_5179005..5291079_00084 | 294 | G_tfc13 | typeG | accessory | 2.400e-118 | 387.400 | 0.955 | 0.956 |
| NZ_CP028959.1_5179005..5291079_00085 | 501 | G_tfc14 | typeG | accessory | 7.400e-185 | 608.400 | 0.996 | 0.992 |
| NZ_CP028959.1_5179005..5291079_00086 | 147 | G_tfc15 | typeG | accessory | 1.500e-59 | 192.900 | 0.957 | 0.925 |
| NZ_CP028959.1_5179005..5291079_00087 | 982 | virb4 | typeG | mandatory | 6.800e-89 | 292.200 | 0.806 | 0.898 |
| NZ_CP028959.1_5179005..5291079_00104 | 143 | G_tfc24 | typeG | accessory | 9.400e-51 | 164.300 | 0.914 | 0.895 |
| NZ_CP028959.1_5179005..5291079_00105 | 312 | G_tfc23 | typeG | accessory | 3.600e-134 | 439.900 | 0.973 | 0.987 |
| NZ_CP028959.1_5179005..5291079_00106 | 460 | G_tfc22 | typeG | accessory | 9.200e-180 | 591.000 | 0.987 | 0.974 |
| NZ_CP028959.1_5179005..5291079_00107 | 115 | G_tfc18 | typeG | accessory | 1.000e-28 | 92.400 | 0.912 | 0.887 |
| NZ_CP028959.1_5179005..5291079_00108 | 516 | G_tfc19 | typeG | accessory | 9.000e-222 | 729.900 | 0.988 | 0.971 |
| NZ_CP028959.1_5179005..5291079_00120 | 639 | MOBH | typeG | mandatory | 3.200e-44 | 143.900 | 0.873 | 0.271 |
| NZ_CP029088.1_5757738..5841631_00035 | 374 | G_tfc2 | typeG | accessory | 1.300e-71 | 232.900 | 0.782 | 0.631 |
| NZ_CP029088.1_5757738..5841631_00066 | 251 | G_tfc3 | typeG | accessory | 6.900e-96 | 312.600 | 0.967 | 0.976 |
| NZ_CP029088.1_5757738..5841631_00068 | 166 | G_tfc5 | typeG | accessory | 2.500e-57 | 186.000 | 0.971 | 0.994 |
| NZ_CP029088.1_5757738..5841631_00070 | 743 | t4cp2 | typeG | mandatory | 3.900e-31 | 100.700 | 0.955 | 0.318 |
| NZ_CP029088.1_5757738..5841631_00071 | 248 | G_tfc7 | typeG | accessory | 9.500e-104 | 339.000 | 1.000 | 1.000 |
| NZ_CP029088.1_5757738..5841631_00077 | 112 | G_tfc8 | typeG | accessory | 5.600e-41 | 131.800 | 0.931 | 0.938 |
| NZ_CP029088.1_5757738..5841631_00078 | 79 | G_tfc9 | typeG | accessory | 1.900e-30 | 97.800 | 1.000 | 1.000 |
| NZ_CP029088.1_5757738..5841631_00079 | 118 | G_tfc10 | typeG | accessory | 5.300e-44 | 141.300 | 0.925 | 0.924 |
| NZ_CP029088.1_5757738..5841631_00080 | 128 | G_tfc11 | typeG | accessory | 9.800e-50 | 160.200 | 0.923 | 0.938 |
| NZ_CP029088.1_5757738..5841631_00081 | 219 | G_tfc12 | typeG | accessory | 1.300e-91 | 298.400 | 0.950 | 0.968 |
| NZ_CP029088.1_5757738..5841631_00082 | 294 | G_tfc13 | typeG | accessory | 9.900e-119 | 388.400 | 0.955 | 0.956 |
| NZ_CP029088.1_5757738..5841631_00083 | 501 | G_tfc14 | typeG | accessory | 2.200e-184 | 606.600 | 0.996 | 0.992 |
| NZ_CP029088.1_5757738..5841631_00084 | 147 | G_tfc15 | typeG | accessory | 1.600e-59 | 192.600 | 0.957 | 0.925 |
| NZ_CP029088.1_5757738..5841631_00085 | 983 | virb4 | typeG | mandatory | 1.200e-89 | 294.500 | 0.810 | 0.899 |
| NZ_CP029088.1_5757738..5841631_00091 | 143 | G_tfc24 | typeG | accessory | 3.600e-51 | 165.400 | 0.907 | 0.888 |
| NZ_CP029088.1_5757738..5841631_00092 | 312 | G_tfc23 | typeG | accessory | 2.700e-134 | 440.100 | 0.973 | 0.987 |
| NZ_CP029088.1_5757738..5841631_00093 | 460 | G_tfc22 | typeG | accessory | 5.800e-180 | 591.400 | 0.989 | 0.976 |
| NZ_CP029088.1_5757738..5841631_00094 | 115 | G_tfc18 | typeG | accessory | 3.600e-29 | 93.600 | 0.912 | 0.887 |
| NZ_CP029088.1_5757738..5841631_00095 | 504 | G_tfc19 | typeG | accessory | 2.200e-223 | 735.000 | 0.988 | 0.994 |
| NZ_CP029088.1_5757738..5841631_00102 | 640 | MOBH | typeG | mandatory | 2.700e-44 | 143.900 | 0.868 | 0.267 |
| NZ_CP029089.1_6693279..6782711_00002 | 640 | MOBH | typeG | mandatory | 2.700e-44 | 143.900 | 0.868 | 0.267 |
| NZ_CP029089.1_6693279..6782711_00009 | 508 | G_tfc19 | typeG | accessory | 1.200e-220 | 726.100 | 0.992 | 0.990 |
| NZ_CP029089.1_6693279..6782711_00010 | 115 | G_tfc18 | typeG | accessory | 3.300e-29 | 93.800 | 0.912 | 0.887 |
| NZ_CP029089.1_6693279..6782711_00011 | 462 | G_tfc22 | typeG | accessory | 4.200e-180 | 591.900 | 0.987 | 0.970 |
| NZ_CP029089.1_6693279..6782711_00012 | 312 | G_tfc23 | typeG | accessory | 9.900e-134 | 438.300 | 0.973 | 0.987 |
| NZ_CP029089.1_6693279..6782711_00013 | 143 | G_tfc24 | typeG | accessory | 4.000e-51 | 165.300 | 0.914 | 0.895 |
| NZ_CP029089.1_6693279..6782711_00019 | 980 | virb4 | typeG | mandatory | 8.400e-90 | 295.000 | 0.807 | 0.899 |
| NZ_CP029089.1_6693279..6782711_00020 | 147 | G_tfc15 | typeG | accessory | 3.500e-60 | 194.800 | 0.957 | 0.925 |
| NZ_CP029089.1_6693279..6782711_00021 | 501 | G_tfc14 | typeG | accessory | 1.000e-184 | 607.800 | 0.996 | 0.992 |
| NZ_CP029089.1_6693279..6782711_00022 | 294 | G_tfc13 | typeG | accessory | 1.600e-118 | 387.800 | 0.955 | 0.956 |
| NZ_CP029089.1_6693279..6782711_00023 | 219 | G_tfc12 | typeG | accessory | 7.300e-92 | 299.200 | 0.950 | 0.968 |
| NZ_CP029089.1_6693279..6782711_00024 | 128 | G_tfc11 | typeG | accessory | 4.700e-50 | 161.300 | 0.923 | 0.938 |
| NZ_CP029089.1_6693279..6782711_00025 | 118 | G_tfc10 | typeG | accessory | 6.500e-44 | 141.000 | 0.875 | 0.890 |
| NZ_CP029089.1_6693279..6782711_00026 | 79 | G_tfc9 | typeG | accessory | 1.000e-30 | 98.700 | 1.000 | 1.000 |
| NZ_CP029089.1_6693279..6782711_00027 | 112 | G_tfc8 | typeG | accessory | 1.300e-40 | 130.600 | 0.914 | 0.920 |
| NZ_CP029089.1_6693279..6782711_00033 | 249 | G_tfc7 | typeG | accessory | 1.400e-103 | 338.500 | 1.000 | 0.996 |
| NZ_CP029089.1_6693279..6782711_00034 | 743 | t4cp2 | typeG | mandatory | 4.000e-31 | 100.700 | 0.955 | 0.318 |
| NZ_CP029089.1_6693279..6782711_00036 | 166 | G_tfc5 | typeG | accessory | 2.400e-57 | 186.100 | 0.971 | 0.994 |
| NZ_CP029089.1_6693279..6782711_00038 | 251 | G_tfc3 | typeG | accessory | 3.400e-96 | 313.600 | 0.988 | 0.996 |
| NZ_CP029090.1_4891592..4986069_00077 | 251 | G_tfc3 | typeG | accessory | 3.900e-96 | 313.600 | 0.988 | 0.996 |
| NZ_CP029090.1_4891592..4986069_00079 | 166 | G_tfc5 | typeG | accessory | 2.800e-57 | 186.100 | 0.971 | 0.994 |
| NZ_CP029090.1_4891592..4986069_00081 | 743 | t4cp2 | typeG | mandatory | 4.700e-31 | 100.700 | 0.955 | 0.318 |
| NZ_CP029090.1_4891592..4986069_00082 | 249 | G_tfc7 | typeG | accessory | 1.100e-103 | 339.100 | 1.000 | 0.996 |
| NZ_CP029090.1_4891592..4986069_00096 | 112 | G_tfc8 | typeG | accessory | 1.500e-40 | 130.600 | 0.914 | 0.920 |
| NZ_CP029090.1_4891592..4986069_00097 | 79 | G_tfc9 | typeG | accessory | 1.200e-30 | 98.700 | 1.000 | 1.000 |
| NZ_CP029090.1_4891592..4986069_00098 | 118 | G_tfc10 | typeG | accessory | 7.600e-44 | 141.000 | 0.875 | 0.890 |
| NZ_CP029090.1_4891592..4986069_00099 | 128 | G_tfc11 | typeG | accessory | 5.400e-50 | 161.300 | 0.923 | 0.938 |
| NZ_CP029090.1_4891592..4986069_00100 | 219 | G_tfc12 | typeG | accessory | 8.500e-92 | 299.200 | 0.950 | 0.968 |
| NZ_CP029090.1_4891592..4986069_00101 | 294 | G_tfc13 | typeG | accessory | 1.800e-118 | 387.800 | 0.955 | 0.956 |
| NZ_CP029090.1_4891592..4986069_00102 | 501 | G_tfc14 | typeG | accessory | 1.200e-184 | 607.800 | 0.996 | 0.992 |
| NZ_CP029090.1_4891592..4986069_00103 | 147 | G_tfc15 | typeG | accessory | 4.300e-60 | 194.700 | 0.957 | 0.925 |
| NZ_CP029090.1_4891592..4986069_00104 | 980 | virb4 | typeG | mandatory | 4.300e-90 | 296.100 | 0.806 | 0.898 |
| NZ_CP029090.1_4891592..4986069_00110 | 143 | G_tfc24 | typeG | accessory | 9.100e-51 | 164.400 | 0.907 | 0.888 |
| NZ_CP029090.1_4891592..4986069_00111 | 312 | G_tfc23 | typeG | accessory | 3.600e-134 | 440.000 | 0.973 | 0.987 |
| NZ_CP029090.1_4891592..4986069_00112 | 460 | G_tfc22 | typeG | accessory | 5.300e-180 | 591.800 | 0.987 | 0.974 |
| NZ_CP029090.1_4891592..4986069_00113 | 115 | G_tfc18 | typeG | accessory | 4.100e-29 | 93.700 | 0.912 | 0.887 |
| NZ_CP029090.1_4891592..4986069_00114 | 504 | G_tfc19 | typeG | accessory | 1.500e-222 | 732.500 | 0.984 | 0.990 |
| NZ_CP029090.1_4891592..4986069_00122 | 639 | MOBH | typeG | mandatory | 4.500e-44 | 143.400 | 0.868 | 0.268 |
| NZ_CP029097.1_1479588..1567435_00002 | 639 | MOBH | typeG | mandatory | 3.000e-44 | 143.900 | 0.868 | 0.268 |
| NZ_CP029097.1_1479588..1567435_00010 | 503 | G_tfc19 | typeG | accessory | 7.400e-222 | 730.100 | 0.984 | 0.992 |
| NZ_CP029097.1_1479588..1567435_00011 | 115 | G_tfc18 | typeG | accessory | 4.000e-29 | 93.600 | 0.912 | 0.887 |
| NZ_CP029097.1_1479588..1567435_00012 | 460 | G_tfc22 | typeG | accessory | 5.700e-180 | 591.600 | 0.987 | 0.974 |
| NZ_CP029097.1_1479588..1567435_00013 | 312 | G_tfc23 | typeG | accessory | 2.400e-134 | 440.400 | 0.973 | 0.987 |
| NZ_CP029097.1_1479588..1567435_00014 | 143 | G_tfc24 | typeG | accessory | 5.900e-51 | 164.900 | 0.914 | 0.895 |
| NZ_CP029097.1_1479588..1567435_00020 | 980 | virb4 | typeG | mandatory | 1.700e-90 | 297.400 | 0.807 | 0.899 |
| NZ_CP029097.1_1479588..1567435_00021 | 147 | G_tfc15 | typeG | accessory | 1.100e-60 | 196.600 | 0.957 | 0.925 |
| NZ_CP029097.1_1479588..1567435_00022 | 501 | G_tfc14 | typeG | accessory | 1.600e-184 | 607.300 | 0.990 | 0.984 |
| NZ_CP029097.1_1479588..1567435_00023 | 294 | G_tfc13 | typeG | accessory | 5.500e-119 | 389.400 | 0.955 | 0.956 |
| NZ_CP029097.1_1479588..1567435_00024 | 219 | G_tfc12 | typeG | accessory | 7.900e-92 | 299.200 | 0.950 | 0.968 |
| NZ_CP029097.1_1479588..1567435_00025 | 128 | G_tfc11 | typeG | accessory | 5.100e-50 | 161.300 | 0.923 | 0.938 |
| NZ_CP029097.1_1479588..1567435_00026 | 118 | G_tfc10 | typeG | accessory | 7.100e-44 | 141.000 | 0.875 | 0.890 |
| NZ_CP029097.1_1479588..1567435_00027 | 79 | G_tfc9 | typeG | accessory | 1.100e-30 | 98.700 | 1.000 | 1.000 |
| NZ_CP029097.1_1479588..1567435_00028 | 112 | G_tfc8 | typeG | accessory | 1.400e-40 | 130.600 | 0.914 | 0.920 |
| NZ_CP029097.1_1479588..1567435_00034 | 249 | G_tfc7 | typeG | accessory | 1.000e-103 | 339.100 | 1.000 | 0.996 |
| NZ_CP029097.1_1479588..1567435_00035 | 743 | t4cp2 | typeG | mandatory | 4.400e-31 | 100.700 | 0.955 | 0.318 |
| NZ_CP029097.1_1479588..1567435_00037 | 166 | G_tfc5 | typeG | accessory | 2.600e-57 | 186.100 | 0.971 | 0.994 |
| NZ_CP029097.1_1479588..1567435_00039 | 251 | G_tfc3 | typeG | accessory | 3.700e-96 | 313.600 | 0.988 | 0.996 |
| NZ_CP029605.1_5752794..5854462_00043 | 374 | G_tfc2 | typeG | accessory | 6.800e-73 | 237.400 | 0.911 | 0.701 |
| NZ_CP029605.1_5752794..5854462_00074 | 250 | G_tfc3 | typeG | accessory | 6.300e-96 | 313.000 | 0.967 | 0.980 |
| NZ_CP029605.1_5752794..5854462_00076 | 166 | G_tfc5 | typeG | accessory | 2.800e-57 | 186.100 | 0.971 | 0.994 |
| NZ_CP029605.1_5752794..5854462_00078 | 743 | t4cp2 | typeG | mandatory | 4.600e-31 | 100.700 | 0.955 | 0.318 |
| NZ_CP029605.1_5752794..5854462_00079 | 249 | G_tfc7 | typeG | accessory | 1.100e-103 | 339.000 | 1.000 | 0.996 |
| NZ_CP029605.1_5752794..5854462_00098 | 112 | G_tfc8 | typeG | accessory | 1.500e-40 | 130.600 | 0.914 | 0.920 |
| NZ_CP029605.1_5752794..5854462_00099 | 79 | G_tfc9 | typeG | accessory | 1.200e-30 | 98.700 | 1.000 | 1.000 |
| NZ_CP029605.1_5752794..5854462_00100 | 118 | G_tfc10 | typeG | accessory | 7.500e-44 | 141.000 | 0.875 | 0.890 |
| NZ_CP029605.1_5752794..5854462_00101 | 128 | G_tfc11 | typeG | accessory | 5.400e-50 | 161.300 | 0.923 | 0.938 |
| NZ_CP029605.1_5752794..5854462_00102 | 219 | G_tfc12 | typeG | accessory | 8.400e-92 | 299.200 | 0.950 | 0.968 |
| NZ_CP029605.1_5752794..5854462_00103 | 294 | G_tfc13 | typeG | accessory | 1.800e-118 | 387.800 | 0.955 | 0.956 |
| NZ_CP029605.1_5752794..5854462_00104 | 501 | G_tfc14 | typeG | accessory | 1.200e-184 | 607.800 | 0.996 | 0.992 |
| NZ_CP029605.1_5752794..5854462_00105 | 147 | G_tfc15 | typeG | accessory | 4.300e-60 | 194.700 | 0.957 | 0.925 |
| NZ_CP029605.1_5752794..5854462_00106 | 980 | virb4 | typeG | mandatory | 1.300e-90 | 297.800 | 0.806 | 0.898 |
| NZ_CP029605.1_5752794..5854462_00111 | 166 | G_tfc24 | typeG | accessory | 1.500e-50 | 163.600 | 0.900 | 0.759 |
| NZ_CP029605.1_5752794..5854462_00112 | 312 | G_tfc23 | typeG | accessory | 3.500e-134 | 440.000 | 0.973 | 0.987 |
| NZ_CP029605.1_5752794..5854462_00113 | 460 | G_tfc22 | typeG | accessory | 1.500e-179 | 590.300 | 0.987 | 0.974 |
| NZ_CP029605.1_5752794..5854462_00114 | 118 | G_tfc18 | typeG | accessory | 1.700e-27 | 88.500 | 0.912 | 0.890 |
| NZ_CP029605.1_5752794..5854462_00115 | 503 | G_tfc19 | typeG | accessory | 1.100e-221 | 729.700 | 0.984 | 0.992 |
| NZ_CP029605.1_5752794..5854462_00121 | 640 | MOBH | typeG | mandatory | 3.100e-44 | 143.900 | 0.868 | 0.267 |
| NZ_CP029707.1_4153870..4238811_00075 | 251 | G_tfc3 | typeG | accessory | 3.300e-96 | 313.600 | 0.988 | 0.996 |
| NZ_CP029707.1_4153870..4238811_00077 | 166 | G_tfc5 | typeG | accessory | 2.200e-57 | 186.200 | 0.971 | 0.994 |
| NZ_CP029707.1_4153870..4238811_00079 | 743 | t4cp2 | typeG | mandatory | 4.000e-31 | 100.700 | 0.955 | 0.318 |
| NZ_CP029707.1_4153870..4238811_00080 | 248 | G_tfc7 | typeG | accessory | 8.500e-104 | 339.200 | 1.000 | 1.000 |
| NZ_CP029707.1_4153870..4238811_00084 | 112 | G_tfc8 | typeG | accessory | 2.800e-41 | 132.800 | 0.922 | 0.929 |
| NZ_CP029707.1_4153870..4238811_00085 | 79 | G_tfc9 | typeG | accessory | 9.300e-31 | 98.800 | 1.000 | 1.000 |
| NZ_CP029707.1_4153870..4238811_00086 | 118 | G_tfc10 | typeG | accessory | 3.400e-45 | 145.100 | 0.925 | 0.924 |
| NZ_CP029707.1_4153870..4238811_00087 | 128 | G_tfc11 | typeG | accessory | 1.000e-49 | 160.100 | 0.923 | 0.938 |
| NZ_CP029707.1_4153870..4238811_00088 | 219 | G_tfc12 | typeG | accessory | 4.400e-91 | 296.600 | 0.946 | 0.963 |
| NZ_CP029707.1_4153870..4238811_00089 | 294 | G_tfc13 | typeG | accessory | 1.100e-118 | 388.300 | 0.944 | 0.959 |
| NZ_CP029707.1_4153870..4238811_00090 | 501 | G_tfc14 | typeG | accessory | 7.500e-185 | 608.200 | 0.996 | 0.992 |
| NZ_CP029707.1_4153870..4238811_00091 | 147 | G_tfc15 | typeG | accessory | 3.400e-60 | 194.800 | 0.957 | 0.925 |
| NZ_CP029707.1_4153870..4238811_00092 | 980 | virb4 | typeG | mandatory | 3.700e-89 | 292.800 | 0.807 | 0.899 |
| NZ_CP029707.1_4153870..4238811_00096 | 143 | G_tfc24 | typeG | accessory | 1.300e-51 | 166.900 | 0.914 | 0.895 |
| NZ_CP029707.1_4153870..4238811_00097 | 312 | G_tfc23 | typeG | accessory | 8.400e-134 | 438.500 | 0.976 | 0.990 |
| NZ_CP029707.1_4153870..4238811_00098 | 460 | G_tfc22 | typeG | accessory | 7.900e-180 | 591.000 | 0.987 | 0.974 |
| NZ_CP029707.1_4153870..4238811_00099 | 117 | G_tfc18 | typeG | accessory | 3.400e-28 | 90.500 | 0.912 | 0.889 |
| NZ_CP029707.1_4153870..4238811_00100 | 510 | G_tfc19 | typeG | accessory | 4.000e-221 | 727.600 | 0.992 | 0.986 |
| NZ_CP029707.1_4153870..4238811_00103 | 639 | MOBH | typeG | mandatory | 6.500e-44 | 142.600 | 0.868 | 0.268 |
| NZ_CP029745.1_4637689..4726795_00037 | 374 | G_tfc2 | typeG | accessory | 2.000e-71 | 232.400 | 0.782 | 0.570 |
| NZ_CP029745.1_4637689..4726795_00068 | 251 | G_tfc3 | typeG | accessory | 3.300e-96 | 313.600 | 0.988 | 0.996 |
| NZ_CP029745.1_4637689..4726795_00070 | 166 | G_tfc5 | typeG | accessory | 2.300e-57 | 186.100 | 0.971 | 0.994 |
| NZ_CP029745.1_4637689..4726795_00072 | 743 | t4cp2 | typeG | mandatory | 3.900e-31 | 100.700 | 0.955 | 0.318 |
| NZ_CP029745.1_4637689..4726795_00073 | 249 | G_tfc7 | typeG | accessory | 1.400e-103 | 338.500 | 1.000 | 0.996 |
| NZ_CP029745.1_4637689..4726795_00078 | 112 | G_tfc8 | typeG | accessory | 1.300e-40 | 130.600 | 0.914 | 0.920 |
| NZ_CP029745.1_4637689..4726795_00079 | 49 | G_tfc9 | typeG | accessory | 7.500e-18 | 57.400 | 0.620 | 1.000 |
| NZ_CP029745.1_4637689..4726795_00080 | 118 | G_tfc10 | typeG | accessory | 6.400e-44 | 141.000 | 0.875 | 0.890 |
| NZ_CP029745.1_4637689..4726795_00081 | 128 | G_tfc11 | typeG | accessory | 4.500e-50 | 161.300 | 0.923 | 0.938 |
| NZ_CP029745.1_4637689..4726795_00082 | 219 | G_tfc12 | typeG | accessory | 7.100e-92 | 299.200 | 0.950 | 0.968 |
| NZ_CP029745.1_4637689..4726795_00083 | 294 | G_tfc13 | typeG | accessory | 1.500e-118 | 387.800 | 0.955 | 0.956 |
| NZ_CP029745.1_4637689..4726795_00084 | 501 | G_tfc14 | typeG | accessory | 9.800e-185 | 607.800 | 0.996 | 0.992 |
| NZ_CP029745.1_4637689..4726795_00085 | 147 | G_tfc15 | typeG | accessory | 3.400e-60 | 194.800 | 0.957 | 0.925 |
| NZ_CP029745.1_4637689..4726795_00086 | 980 | virb4 | typeG | mandatory | 8.200e-90 | 295.000 | 0.807 | 0.899 |
| NZ_CP029745.1_4637689..4726795_00091 | 143 | G_tfc24 | typeG | accessory | 3.900e-51 | 165.300 | 0.914 | 0.895 |
| NZ_CP029745.1_4637689..4726795_00092 | 312 | G_tfc23 | typeG | accessory | 9.600e-134 | 438.300 | 0.973 | 0.987 |
| NZ_CP029745.1_4637689..4726795_00093 | 462 | G_tfc22 | typeG | accessory | 4.100e-180 | 591.900 | 0.987 | 0.970 |
| NZ_CP029745.1_4637689..4726795_00094 | 115 | G_tfc18 | typeG | accessory | 3.200e-29 | 93.800 | 0.912 | 0.887 |
| NZ_CP029745.1_4637689..4726795_00095 | 508 | G_tfc19 | typeG | accessory | 1.100e-220 | 726.100 | 0.992 | 0.990 |
| NZ_CP029745.1_4637689..4726795_00102 | 640 | MOBH | typeG | mandatory | 2.700e-44 | 143.900 | 0.868 | 0.267 |
| NZ_CP030328.1_6407..111863_00037 | 374 | G_tfc2 | typeG | accessory | 6.900e-73 | 237.400 | 0.911 | 0.701 |
| NZ_CP030328.1_6407..111863_00067 | 251 | G_tfc3 | typeG | accessory | 5.300e-96 | 313.200 | 0.988 | 0.996 |
| NZ_CP030328.1_6407..111863_00069 | 166 | G_tfc5 | typeG | accessory | 3.000e-57 | 186.000 | 0.971 | 0.994 |
| NZ_CP030328.1_6407..111863_00071 | 743 | t4cp2 | typeG | mandatory | 4.700e-31 | 100.700 | 0.955 | 0.318 |
| NZ_CP030328.1_6407..111863_00072 | 248 | G_tfc7 | typeG | accessory | 1.100e-103 | 339.000 | 1.000 | 1.000 |
| NZ_CP030328.1_6407..111863_00098 | 112 | G_tfc8 | typeG | accessory | 1.500e-40 | 130.600 | 0.914 | 0.920 |
| NZ_CP030328.1_6407..111863_00099 | 79 | G_tfc9 | typeG | accessory | 1.200e-30 | 98.700 | 1.000 | 1.000 |
| NZ_CP030328.1_6407..111863_00100 | 118 | G_tfc10 | typeG | accessory | 7.600e-44 | 141.000 | 0.875 | 0.890 |
| NZ_CP030328.1_6407..111863_00101 | 128 | G_tfc11 | typeG | accessory | 5.500e-50 | 161.300 | 0.923 | 0.938 |
| NZ_CP030328.1_6407..111863_00102 | 219 | G_tfc12 | typeG | accessory | 8.500e-92 | 299.200 | 0.950 | 0.968 |
| NZ_CP030328.1_6407..111863_00103 | 294 | G_tfc13 | typeG | accessory | 1.900e-118 | 387.800 | 0.955 | 0.956 |
| NZ_CP030328.1_6407..111863_00104 | 501 | G_tfc14 | typeG | accessory | 1.200e-184 | 607.800 | 0.996 | 0.992 |
| NZ_CP030328.1_6407..111863_00105 | 147 | G_tfc15 | typeG | accessory | 4.300e-60 | 194.700 | 0.957 | 0.925 |
| NZ_CP030328.1_6407..111863_00106 | 980 | virb4 | typeG | mandatory | 4.300e-90 | 296.100 | 0.806 | 0.898 |
| NZ_CP030328.1_6407..111863_00112 | 143 | G_tfc24 | typeG | accessory | 1.900e-50 | 163.300 | 0.914 | 0.895 |
| NZ_CP030328.1_6407..111863_00113 | 312 | G_tfc23 | typeG | accessory | 1.200e-133 | 438.200 | 0.973 | 0.987 |
| NZ_CP030328.1_6407..111863_00114 | 462 | G_tfc22 | typeG | accessory | 7.500e-180 | 591.300 | 0.987 | 0.970 |
| NZ_CP030328.1_6407..111863_00115 | 114 | G_tfc18 | typeG | accessory | 1.200e-27 | 89.000 | 0.912 | 0.886 |
| NZ_CP030328.1_6407..111863_00116 | 503 | G_tfc19 | typeG | accessory | 8.300e-222 | 730.100 | 0.984 | 0.992 |
| NZ_CP030328.1_6407..111863_00123 | 639 | MOBH | typeG | mandatory | 4.500e-44 | 143.400 | 0.868 | 0.268 |
| NZ_CP030861.1_4536891..4635175_00003 | 639 | MOBH | typeG | mandatory | 4.700e-44 | 143.400 | 0.868 | 0.268 |
| NZ_CP030861.1_4536891..4635175_00013 | 511 | G_tfc19 | typeG | accessory | 7.000e-223 | 733.600 | 0.990 | 0.982 |
| NZ_CP030861.1_4536891..4635175_00014 | 115 | G_tfc18 | typeG | accessory | 3.900e-29 | 93.800 | 0.912 | 0.887 |
| NZ_CP030861.1_4536891..4635175_00016 | 279 | G_tfc22 | typeG | accessory | 2.400e-105 | 345.700 | 0.558 | 0.910 |
| NZ_CP030861.1_4536891..4635175_00017 | 312 | G_tfc23 | typeG | accessory | 9.200e-134 | 438.600 | 0.973 | 0.987 |
| NZ_CP030861.1_4536891..4635175_00018 | 143 | G_tfc24 | typeG | accessory | 9.200e-51 | 164.400 | 0.907 | 0.888 |
| NZ_CP030861.1_4536891..4635175_00024 | 980 | virb4 | typeG | mandatory | 1.300e-90 | 297.900 | 0.806 | 0.898 |
| NZ_CP030861.1_4536891..4635175_00025 | 147 | G_tfc15 | typeG | accessory | 4.300e-60 | 194.700 | 0.957 | 0.925 |
| NZ_CP030861.1_4536891..4635175_00026 | 501 | G_tfc14 | typeG | accessory | 1.100e-183 | 604.600 | 0.996 | 0.992 |
| NZ_CP030861.1_4536891..4635175_00027 | 294 | G_tfc13 | typeG | accessory | 4.200e-118 | 386.700 | 0.955 | 0.956 |
| NZ_CP030861.1_4536891..4635175_00028 | 219 | G_tfc12 | typeG | accessory | 8.500e-92 | 299.200 | 0.950 | 0.968 |
| NZ_CP030861.1_4536891..4635175_00029 | 128 | G_tfc11 | typeG | accessory | 5.500e-50 | 161.300 | 0.923 | 0.938 |
| NZ_CP030861.1_4536891..4635175_00030 | 118 | G_tfc10 | typeG | accessory | 7.600e-44 | 141.000 | 0.875 | 0.890 |
| NZ_CP030861.1_4536891..4635175_00031 | 79 | G_tfc9 | typeG | accessory | 1.400e-30 | 98.500 | 1.000 | 1.000 |
| NZ_CP030861.1_4536891..4635175_00032 | 112 | G_tfc8 | typeG | accessory | 2.000e-41 | 133.500 | 0.931 | 0.938 |
| NZ_CP030861.1_4536891..4635175_00037 | 248 | G_tfc7 | typeG | accessory | 4.700e-103 | 337.000 | 1.000 | 1.000 |
| NZ_CP030861.1_4536891..4635175_00038 | 743 | t4cp2 | typeG | mandatory | 4.700e-31 | 100.700 | 0.955 | 0.318 |
| NZ_CP030861.1_4536891..4635175_00040 | 166 | G_tfc5 | typeG | accessory | 3.700e-57 | 185.700 | 0.971 | 0.994 |
| NZ_CP030861.1_4536891..4635175_00042 | 251 | G_tfc3 | typeG | accessory | 8.700e-96 | 312.500 | 0.967 | 0.976 |
| NZ_CP030910.1_1166409..1272911_00002 | 639 | MOBH | typeG | mandatory | 3.100e-44 | 143.900 | 0.873 | 0.271 |
| NZ_CP030910.1_1166409..1272911_00013 | 511 | G_tfc19 | typeG | accessory | 1.200e-222 | 732.700 | 0.990 | 0.982 |
| NZ_CP030910.1_1166409..1272911_00014 | 115 | G_tfc18 | typeG | accessory | 3.400e-29 | 93.900 | 0.895 | 0.878 |
| NZ_CP030910.1_1166409..1272911_00015 | 460 | G_tfc22 | typeG | accessory | 1.800e-179 | 589.900 | 0.987 | 0.974 |
| NZ_CP030910.1_1166409..1272911_00016 | 312 | G_tfc23 | typeG | accessory | 6.300e-134 | 439.100 | 0.973 | 0.987 |
| NZ_CP030910.1_1166409..1272911_00017 | 143 | G_tfc24 | typeG | accessory | 4.900e-50 | 161.900 | 0.914 | 0.895 |
| NZ_CP030910.1_1166409..1272911_00023 | 980 | virb4 | typeG | mandatory | 8.100e-91 | 298.500 | 0.808 | 0.900 |
| NZ_CP030910.1_1166409..1272911_00024 | 147 | G_tfc15 | typeG | accessory | 3.600e-60 | 194.900 | 0.957 | 0.925 |
| NZ_CP030910.1_1166409..1272911_00025 | 501 | G_tfc14 | typeG | accessory | 7.900e-185 | 608.300 | 0.996 | 0.992 |
| NZ_CP030910.1_1166409..1272911_00026 | 294 | G_tfc13 | typeG | accessory | 7.200e-117 | 382.500 | 0.941 | 0.952 |
| NZ_CP030910.1_1166409..1272911_00027 | 219 | G_tfc12 | typeG | accessory | 6.600e-91 | 296.200 | 0.941 | 0.959 |
| NZ_CP030910.1_1166409..1272911_00028 | 128 | G_tfc11 | typeG | accessory | 8.800e-50 | 160.500 | 0.923 | 0.938 |
| NZ_CP030910.1_1166409..1272911_00029 | 118 | G_tfc10 | typeG | accessory | 3.100e-44 | 142.200 | 0.875 | 0.890 |
| NZ_CP030910.1_1166409..1272911_00030 | 79 | G_tfc9 | typeG | accessory | 8.900e-31 | 99.000 | 1.000 | 1.000 |
| NZ_CP030910.1_1166409..1272911_00031 | 112 | G_tfc8 | typeG | accessory | 6.600e-41 | 131.700 | 0.922 | 0.929 |
| NZ_CP030910.1_1166409..1272911_00035 | 248 | G_tfc7 | typeG | accessory | 8.200e-104 | 339.400 | 1.000 | 1.000 |
| NZ_CP030910.1_1166409..1272911_00036 | 743 | t4cp2 | typeG | mandatory | 4.400e-31 | 100.700 | 0.955 | 0.318 |
| NZ_CP030910.1_1166409..1272911_00038 | 166 | G_tfc5 | typeG | accessory | 2.700e-57 | 186.100 | 0.971 | 0.994 |
| NZ_CP030910.1_1166409..1272911_00040 | 251 | G_tfc3 | typeG | accessory | 7.400e-97 | 315.900 | 0.988 | 0.996 |
| NZ_CP030911.1_5555242..5641369_00032 | 374 | G_tfc2 | typeG | accessory | 2.400e-71 | 232.100 | 0.782 | 0.599 |
| NZ_CP030911.1_5555242..5641369_00062 | 251 | G_tfc3 | typeG | accessory | 9.200e-96 | 312.200 | 0.967 | 0.976 |
| NZ_CP030911.1_5555242..5641369_00064 | 166 | G_tfc5 | typeG | accessory | 2.400e-57 | 186.000 | 0.971 | 0.994 |
| NZ_CP030911.1_5555242..5641369_00066 | 743 | t4cp2 | typeG | mandatory | 3.900e-31 | 100.700 | 0.955 | 0.318 |
| NZ_CP030911.1_5555242..5641369_00067 | 248 | G_tfc7 | typeG | accessory | 9.400e-104 | 339.000 | 1.000 | 1.000 |
| NZ_CP030911.1_5555242..5641369_00073 | 112 | G_tfc8 | typeG | accessory | 5.600e-41 | 131.800 | 0.931 | 0.938 |
| NZ_CP030911.1_5555242..5641369_00074 | 79 | G_tfc9 | typeG | accessory | 1.800e-30 | 97.800 | 1.000 | 1.000 |
| NZ_CP030911.1_5555242..5641369_00075 | 118 | G_tfc10 | typeG | accessory | 5.300e-44 | 141.300 | 0.925 | 0.924 |
| NZ_CP030911.1_5555242..5641369_00076 | 128 | G_tfc11 | typeG | accessory | 9.700e-50 | 160.200 | 0.923 | 0.938 |
| NZ_CP030911.1_5555242..5641369_00077 | 219 | G_tfc12 | typeG | accessory | 1.300e-91 | 298.400 | 0.950 | 0.968 |
| NZ_CP030911.1_5555242..5641369_00078 | 294 | G_tfc13 | typeG | accessory | 9.800e-119 | 388.400 | 0.955 | 0.956 |
| NZ_CP030911.1_5555242..5641369_00079 | 501 | G_tfc14 | typeG | accessory | 2.200e-184 | 606.600 | 0.996 | 0.992 |
| NZ_CP030911.1_5555242..5641369_00080 | 147 | G_tfc15 | typeG | accessory | 1.600e-59 | 192.600 | 0.957 | 0.925 |
| NZ_CP030911.1_5555242..5641369_00081 | 983 | virb4 | typeG | mandatory | 1.200e-89 | 294.500 | 0.810 | 0.899 |
| NZ_CP030911.1_5555242..5641369_00087 | 143 | G_tfc24 | typeG | accessory | 3.500e-51 | 165.400 | 0.907 | 0.888 |
| NZ_CP030911.1_5555242..5641369_00088 | 312 | G_tfc23 | typeG | accessory | 2.700e-134 | 440.100 | 0.973 | 0.987 |
| NZ_CP030911.1_5555242..5641369_00089 | 460 | G_tfc22 | typeG | accessory | 5.200e-180 | 591.500 | 0.989 | 0.976 |
| NZ_CP030911.1_5555242..5641369_00090 | 115 | G_tfc18 | typeG | accessory | 3.500e-29 | 93.600 | 0.912 | 0.887 |
| NZ_CP030911.1_5555242..5641369_00091 | 504 | G_tfc19 | typeG | accessory | 2.200e-223 | 735.000 | 0.988 | 0.994 |
| NZ_CP030911.1_5555242..5641369_00101 | 640 | MOBH | typeG | mandatory | 2.600e-44 | 143.900 | 0.868 | 0.267 |
| NZ_CP030912.1_4834779..4921886_00033 | 374 | G_tfc2 | typeG | accessory | 1.300e-71 | 232.900 | 0.782 | 0.631 |
| NZ_CP030912.1_4834779..4921886_00063 | 251 | G_tfc3 | typeG | accessory | 8.700e-96 | 312.200 | 0.967 | 0.976 |
| NZ_CP030912.1_4834779..4921886_00065 | 166 | G_tfc5 | typeG | accessory | 2.300e-57 | 186.000 | 0.971 | 0.994 |
| NZ_CP030912.1_4834779..4921886_00067 | 743 | t4cp2 | typeG | mandatory | 3.700e-31 | 100.700 | 0.955 | 0.318 |
| NZ_CP030912.1_4834779..4921886_00068 | 248 | G_tfc7 | typeG | accessory | 9.000e-104 | 339.000 | 1.000 | 1.000 |
| NZ_CP030912.1_4834779..4921886_00074 | 112 | G_tfc8 | typeG | accessory | 5.300e-41 | 131.800 | 0.931 | 0.938 |
| NZ_CP030912.1_4834779..4921886_00075 | 79 | G_tfc9 | typeG | accessory | 1.800e-30 | 97.800 | 1.000 | 1.000 |
| NZ_CP030912.1_4834779..4921886_00076 | 118 | G_tfc10 | typeG | accessory | 5.000e-44 | 141.300 | 0.925 | 0.924 |
| NZ_CP030912.1_4834779..4921886_00077 | 128 | G_tfc11 | typeG | accessory | 4.300e-50 | 161.300 | 0.923 | 0.938 |
| NZ_CP030912.1_4834779..4921886_00078 | 219 | G_tfc12 | typeG | accessory | 6.700e-92 | 299.200 | 0.950 | 0.968 |
| NZ_CP030912.1_4834779..4921886_00079 | 294 | G_tfc13 | typeG | accessory | 1.500e-118 | 387.800 | 0.955 | 0.956 |
| NZ_CP030912.1_4834779..4921886_00080 | 501 | G_tfc14 | typeG | accessory | 9.200e-185 | 607.800 | 0.996 | 0.992 |
| NZ_CP030912.1_4834779..4921886_00081 | 147 | G_tfc15 | typeG | accessory | 3.400e-60 | 194.700 | 0.957 | 0.925 |
| NZ_CP030912.1_4834779..4921886_00082 | 980 | virb4 | typeG | mandatory | 5.100e-91 | 298.900 | 0.806 | 0.898 |
| NZ_CP030912.1_4834779..4921886_00088 | 143 | G_tfc24 | typeG | accessory | 7.200e-51 | 164.400 | 0.907 | 0.888 |
| NZ_CP030912.1_4834779..4921886_00089 | 312 | G_tfc23 | typeG | accessory | 7.200e-134 | 438.600 | 0.973 | 0.987 |
| NZ_CP030912.1_4834779..4921886_00090 | 463 | G_tfc22 | typeG | accessory | 3.200e-180 | 592.200 | 0.989 | 0.970 |
| NZ_CP030912.1_4834779..4921886_00091 | 121 | G_tfc18 | typeG | accessory | 1.100e-27 | 88.700 | 0.930 | 0.860 |
| NZ_CP030912.1_4834779..4921886_00092 | 510 | G_tfc19 | typeG | accessory | 2.900e-221 | 727.900 | 0.994 | 0.988 |
| NZ_CP030912.1_4834779..4921886_00096 | 629 | MOBH | typeG | mandatory | 2.400e-44 | 143.900 | 0.868 | 0.272 |
| NZ_CP030913.1_5492151..5581179_00036 | 374 | G_tfc2 | typeG | accessory | 2.400e-71 | 232.100 | 0.782 | 0.599 |
| NZ_CP030913.1_5492151..5581179_00065 | 251 | G_tfc3 | typeG | accessory | 9.400e-96 | 312.200 | 0.967 | 0.976 |
| NZ_CP030913.1_5492151..5581179_00067 | 166 | G_tfc5 | typeG | accessory | 2.500e-57 | 186.000 | 0.971 | 0.994 |
| NZ_CP030913.1_5492151..5581179_00069 | 743 | t4cp2 | typeG | mandatory | 4.000e-31 | 100.700 | 0.955 | 0.318 |
| NZ_CP030913.1_5492151..5581179_00070 | 248 | G_tfc7 | typeG | accessory | 9.700e-104 | 339.000 | 1.000 | 1.000 |
| NZ_CP030913.1_5492151..5581179_00076 | 112 | G_tfc8 | typeG | accessory | 5.700e-41 | 131.800 | 0.931 | 0.938 |
| NZ_CP030913.1_5492151..5581179_00077 | 79 | G_tfc9 | typeG | accessory | 1.900e-30 | 97.800 | 1.000 | 1.000 |
| NZ_CP030913.1_5492151..5581179_00078 | 118 | G_tfc10 | typeG | accessory | 5.400e-44 | 141.300 | 0.925 | 0.924 |
| NZ_CP030913.1_5492151..5581179_00079 | 128 | G_tfc11 | typeG | accessory | 1.000e-49 | 160.200 | 0.923 | 0.938 |
| NZ_CP030913.1_5492151..5581179_00080 | 219 | G_tfc12 | typeG | accessory | 1.300e-91 | 298.400 | 0.950 | 0.968 |
| NZ_CP030913.1_5492151..5581179_00081 | 294 | G_tfc13 | typeG | accessory | 1.000e-118 | 388.400 | 0.955 | 0.956 |
| NZ_CP030913.1_5492151..5581179_00082 | 501 | G_tfc14 | typeG | accessory | 2.200e-184 | 606.600 | 0.996 | 0.992 |
| NZ_CP030913.1_5492151..5581179_00083 | 147 | G_tfc15 | typeG | accessory | 1.700e-59 | 192.600 | 0.957 | 0.925 |
| NZ_CP030913.1_5492151..5581179_00084 | 983 | virb4 | typeG | mandatory | 1.200e-89 | 294.500 | 0.810 | 0.899 |
| NZ_CP030913.1_5492151..5581179_00090 | 143 | G_tfc24 | typeG | accessory | 3.700e-51 | 165.400 | 0.907 | 0.888 |
| NZ_CP030913.1_5492151..5581179_00091 | 312 | G_tfc23 | typeG | accessory | 2.800e-134 | 440.100 | 0.973 | 0.987 |
| NZ_CP030913.1_5492151..5581179_00092 | 460 | G_tfc22 | typeG | accessory | 6.200e-180 | 591.300 | 0.987 | 0.974 |
| NZ_CP030913.1_5492151..5581179_00093 | 115 | G_tfc18 | typeG | accessory | 2.600e-29 | 94.100 | 0.912 | 0.887 |
| NZ_CP030913.1_5492151..5581179_00094 | 510 | G_tfc19 | typeG | accessory | 1.100e-221 | 729.500 | 0.992 | 0.986 |
| NZ_CP030913.1_5492151..5581179_00104 | 640 | MOBH | typeG | mandatory | 2.700e-44 | 143.900 | 0.868 | 0.267 |
| NZ_CP031449.2_5597669..5708907_00068 | 251 | G_tfc3 | typeG | accessory | 1.700e-95 | 311.500 | 0.988 | 0.996 |
| NZ_CP031449.2_5597669..5708907_00070 | 166 | G_tfc5 | typeG | accessory | 2.600e-57 | 186.100 | 0.971 | 0.994 |
| NZ_CP031449.2_5597669..5708907_00072 | 743 | t4cp2 | typeG | mandatory | 4.300e-31 | 100.700 | 0.955 | 0.318 |
| NZ_CP031449.2_5597669..5708907_00073 | 248 | G_tfc7 | typeG | accessory | 1.500e-103 | 338.500 | 1.000 | 1.000 |
| NZ_CP031449.2_5597669..5708907_00078 | 112 | G_tfc8 | typeG | accessory | 2.200e-40 | 130.000 | 0.922 | 0.929 |
| NZ_CP031449.2_5597669..5708907_00079 | 79 | G_tfc9 | typeG | accessory | 8.700e-31 | 99.000 | 1.000 | 1.000 |
| NZ_CP031449.2_5597669..5708907_00080 | 118 | G_tfc10 | typeG | accessory | 7.000e-45 | 144.300 | 0.933 | 0.932 |
| NZ_CP031449.2_5597669..5708907_00081 | 128 | G_tfc11 | typeG | accessory | 4.000e-50 | 161.600 | 0.923 | 0.938 |
| NZ_CP031449.2_5597669..5708907_00082 | 219 | G_tfc12 | typeG | accessory | 2.700e-91 | 297.400 | 0.950 | 0.968 |
| NZ_CP031449.2_5597669..5708907_00083 | 294 | G_tfc13 | typeG | accessory | 5.600e-118 | 386.100 | 0.944 | 0.956 |
| NZ_CP031449.2_5597669..5708907_00084 | 501 | G_tfc14 | typeG | accessory | 1.300e-184 | 607.600 | 0.996 | 0.992 |
| NZ_CP031449.2_5597669..5708907_00085 | 147 | G_tfc15 | typeG | accessory | 7.400e-60 | 193.900 | 0.957 | 0.925 |
| NZ_CP031449.2_5597669..5708907_00086 | 980 | virb4 | typeG | mandatory | 1.300e-89 | 294.500 | 0.808 | 0.900 |
| NZ_CP031449.2_5597669..5708907_00104 | 129 | G_tfc24 | typeG | accessory | 2.800e-50 | 162.700 | 0.900 | 0.977 |
| NZ_CP031449.2_5597669..5708907_00105 | 312 | G_tfc23 | typeG | accessory | 1.100e-133 | 438.200 | 0.973 | 0.987 |
| NZ_CP031449.2_5597669..5708907_00106 | 460 | G_tfc22 | typeG | accessory | 5.700e-180 | 591.600 | 0.987 | 0.974 |
| NZ_CP031449.2_5597669..5708907_00107 | 115 | G_tfc18 | typeG | accessory | 3.900e-29 | 93.700 | 0.912 | 0.887 |
| NZ_CP031449.2_5597669..5708907_00108 | 503 | G_tfc19 | typeG | accessory | 1.000e-222 | 733.000 | 0.984 | 0.992 |
| NZ_CP031449.2_5597669..5708907_00113 | 639 | MOBH | typeG | mandatory | 3.100e-44 | 143.800 | 0.868 | 0.268 |
| NZ_CP031659.1_5260858..5365171_00061 | 250 | G_tfc3 | typeG | accessory | 8.800e-96 | 312.300 | 0.963 | 0.976 |
| NZ_CP031659.1_5260858..5365171_00063 | 166 | G_tfc5 | typeG | accessory | 9.500e-57 | 184.100 | 0.977 | 1.000 |
| NZ_CP031659.1_5260858..5365171_00065 | 743 | t4cp2 | typeG | mandatory | 7.200e-30 | 96.600 | 0.955 | 0.318 |
| NZ_CP031659.1_5260858..5365171_00066 | 248 | G_tfc7 | typeG | accessory | 9.100e-103 | 335.800 | 1.000 | 1.000 |
| NZ_CP031659.1_5260858..5365171_00076 | 112 | G_tfc8 | typeG | accessory | 5.900e-41 | 131.700 | 0.922 | 0.929 |
| NZ_CP031659.1_5260858..5365171_00077 | 79 | G_tfc9 | typeG | accessory | 3.800e-31 | 100.100 | 1.000 | 1.000 |
| NZ_CP031659.1_5260858..5365171_00078 | 118 | G_tfc10 | typeG | accessory | 6.900e-45 | 144.100 | 0.883 | 0.890 |
| NZ_CP031659.1_5260858..5365171_00079 | 128 | G_tfc11 | typeG | accessory | 3.900e-49 | 158.200 | 0.923 | 0.938 |
| NZ_CP031659.1_5260858..5365171_00080 | 219 | G_tfc12 | typeG | accessory | 1.300e-91 | 298.400 | 0.950 | 0.968 |
| NZ_CP031659.1_5260858..5365171_00081 | 293 | G_tfc13 | typeG | accessory | 8.800e-119 | 388.600 | 0.955 | 0.956 |
| NZ_CP031659.1_5260858..5365171_00082 | 501 | G_tfc14 | typeG | accessory | 1.000e-184 | 607.700 | 0.996 | 0.992 |
| NZ_CP031659.1_5260858..5365171_00083 | 147 | G_tfc15 | typeG | accessory | 1.300e-59 | 192.900 | 0.957 | 0.925 |
| NZ_CP031659.1_5260858..5365171_00084 | 980 | virb4 | typeG | mandatory | 2.500e-89 | 293.400 | 0.806 | 0.898 |
| NZ_CP031659.1_5260858..5365171_00089 | 312 | G_tfc23 | typeG | accessory | 7.100e-134 | 438.800 | 0.973 | 0.987 |
| NZ_CP031659.1_5260858..5365171_00090 | 460 | G_tfc22 | typeG | accessory | 7.900e-180 | 591.000 | 0.987 | 0.974 |
| NZ_CP031659.1_5260858..5365171_00091 | 115 | G_tfc18 | typeG | accessory | 5.300e-29 | 93.100 | 0.912 | 0.887 |
| NZ_CP031659.1_5260858..5365171_00092 | 503 | G_tfc19 | typeG | accessory | 1.500e-221 | 728.900 | 0.986 | 0.994 |
| NZ_CP031659.1_5260858..5365171_00103 | 639 | MOBH | typeG | mandatory | 5.900e-44 | 142.800 | 0.868 | 0.268 |
| NZ_CP031660.1_5228262..5320455_00070 | 251 | G_tfc3 | typeG | accessory | 2.500e-95 | 310.800 | 0.967 | 0.976 |
| NZ_CP031660.1_5228262..5320455_00072 | 166 | G_tfc5 | typeG | accessory | 4.000e-57 | 185.400 | 0.971 | 0.994 |
| NZ_CP031660.1_5228262..5320455_00074 | 743 | t4cp2 | typeG | mandatory | 4.100e-31 | 100.700 | 0.955 | 0.318 |
| NZ_CP031660.1_5228262..5320455_00075 | 249 | G_tfc7 | typeG | accessory | 9.400e-104 | 339.100 | 1.000 | 0.996 |
| NZ_CP031660.1_5228262..5320455_00080 | 112 | G_tfc8 | typeG | accessory | 1.300e-40 | 130.600 | 0.914 | 0.920 |
| NZ_CP031660.1_5228262..5320455_00081 | 49 | G_tfc9 | typeG | accessory | 7.800e-18 | 57.400 | 0.620 | 1.000 |
| NZ_CP031660.1_5228262..5320455_00082 | 118 | G_tfc10 | typeG | accessory | 7.500e-44 | 140.900 | 0.875 | 0.890 |
| NZ_CP031660.1_5228262..5320455_00083 | 128 | G_tfc11 | typeG | accessory | 5.500e-50 | 161.100 | 0.923 | 0.938 |
| NZ_CP031660.1_5228262..5320455_00084 | 219 | G_tfc12 | typeG | accessory | 2.600e-91 | 297.400 | 0.950 | 0.968 |
| NZ_CP031660.1_5228262..5320455_00085 | 294 | G_tfc13 | typeG | accessory | 3.100e-118 | 386.900 | 0.941 | 0.956 |
| NZ_CP031660.1_5228262..5320455_00086 | 501 | G_tfc14 | typeG | accessory | 1.000e-184 | 607.800 | 0.996 | 0.992 |
| NZ_CP031660.1_5228262..5320455_00087 | 147 | G_tfc15 | typeG | accessory | 3.500e-60 | 194.800 | 0.957 | 0.925 |
| NZ_CP031660.1_5228262..5320455_00088 | 980 | virb4 | typeG | mandatory | 1.900e-90 | 297.100 | 0.807 | 0.899 |
| NZ_CP031660.1_5228262..5320455_00093 | 143 | G_tfc24 | typeG | accessory | 1.300e-51 | 166.900 | 0.914 | 0.895 |
| NZ_CP031660.1_5228262..5320455_00094 | 312 | G_tfc23 | typeG | accessory | 6.400e-134 | 439.000 | 0.976 | 0.990 |
| NZ_CP031660.1_5228262..5320455_00095 | 460 | G_tfc22 | typeG | accessory | 1.100e-179 | 590.600 | 0.985 | 0.972 |
| NZ_CP031660.1_5228262..5320455_00096 | 115 | G_tfc18 | typeG | accessory | 6.100e-29 | 92.900 | 0.912 | 0.887 |
| NZ_CP031660.1_5228262..5320455_00097 | 504 | G_tfc19 | typeG | accessory | 5.800e-223 | 733.700 | 0.984 | 0.990 |
| NZ_CP031660.1_5228262..5320455_00106 | 639 | MOBH | typeG | mandatory | 2.300e-44 | 144.200 | 0.868 | 0.268 |
| NZ_CP032126.1_5086423..5194320_00085 | 251 | G_tfc3 | typeG | accessory | 1.500e-95 | 311.700 | 0.988 | 0.996 |
| NZ_CP032126.1_5086423..5194320_00087 | 166 | G_tfc5 | typeG | accessory | 9.000e-57 | 184.400 | 0.977 | 1.000 |
| NZ_CP032126.1_5086423..5194320_00089 | 743 | t4cp2 | typeG | mandatory | 4.600e-31 | 100.700 | 0.955 | 0.318 |
| NZ_CP032126.1_5086423..5194320_00090 | 248 | G_tfc7 | typeG | accessory | 1.600e-103 | 338.500 | 1.000 | 1.000 |
| NZ_CP032126.1_5086423..5194320_00095 | 112 | G_tfc8 | typeG | accessory | 1.900e-41 | 133.500 | 0.931 | 0.938 |
| NZ_CP032126.1_5086423..5194320_00096 | 79 | G_tfc9 | typeG | accessory | 1.400e-30 | 98.500 | 1.000 | 1.000 |
| NZ_CP032126.1_5086423..5194320_00097 | 118 | G_tfc10 | typeG | accessory | 4.500e-44 | 141.700 | 0.925 | 0.924 |
| NZ_CP032126.1_5086423..5194320_00098 | 128 | G_tfc11 | typeG | accessory | 7.600e-50 | 160.800 | 0.923 | 0.938 |
| NZ_CP032126.1_5086423..5194320_00099 | 219 | G_tfc12 | typeG | accessory | 8.500e-91 | 295.900 | 0.941 | 0.959 |
| NZ_CP032126.1_5086423..5194320_00100 | 294 | G_tfc13 | typeG | accessory | 3.900e-118 | 386.700 | 0.944 | 0.959 |
| NZ_CP032126.1_5086423..5194320_00101 | 501 | G_tfc14 | typeG | accessory | 9.000e-184 | 604.800 | 0.996 | 0.992 |
| NZ_CP032126.1_5086423..5194320_00102 | 147 | G_tfc15 | typeG | accessory | 4.100e-60 | 194.700 | 0.957 | 0.925 |
| NZ_CP032126.1_5086423..5194320_00103 | 980 | virb4 | typeG | mandatory | 9.500e-90 | 295.000 | 0.807 | 0.899 |
| NZ_CP032126.1_5086423..5194320_00108 | 143 | G_tfc24 | typeG | accessory | 8.900e-51 | 164.400 | 0.907 | 0.888 |
| NZ_CP032126.1_5086423..5194320_00109 | 312 | G_tfc23 | typeG | accessory | 7.800e-134 | 438.800 | 0.973 | 0.987 |
| NZ_CP032126.1_5086423..5194320_00110 | 460 | G_tfc22 | typeG | accessory | 4.100e-179 | 588.800 | 0.982 | 0.970 |
| NZ_CP032126.1_5086423..5194320_00111 | 115 | G_tfc18 | typeG | accessory | 3.000e-29 | 94.100 | 0.912 | 0.887 |
| NZ_CP032126.1_5086423..5194320_00112 | 510 | G_tfc19 | typeG | accessory | 1.500e-221 | 729.200 | 0.992 | 0.986 |
| NZ_CP032126.1_5086423..5194320_00119 | 640 | MOBH | typeG | mandatory | 3.100e-44 | 143.900 | 0.868 | 0.267 |
| NZ_CP032257.1_1227760..1316873_00037 | 374 | G_tfc2 | typeG | accessory | 2.000e-71 | 232.400 | 0.782 | 0.570 |
| NZ_CP032257.1_1227760..1316873_00068 | 251 | G_tfc3 | typeG | accessory | 6.900e-96 | 312.600 | 0.967 | 0.976 |
| NZ_CP032257.1_1227760..1316873_00070 | 166 | G_tfc5 | typeG | accessory | 2.500e-57 | 186.000 | 0.971 | 0.994 |
| NZ_CP032257.1_1227760..1316873_00072 | 743 | t4cp2 | typeG | mandatory | 3.900e-31 | 100.700 | 0.955 | 0.318 |
| NZ_CP032257.1_1227760..1316873_00073 | 249 | G_tfc7 | typeG | accessory | 1.400e-103 | 338.500 | 1.000 | 0.996 |
| NZ_CP032257.1_1227760..1316873_00078 | 112 | G_tfc8 | typeG | accessory | 1.300e-40 | 130.600 | 0.914 | 0.920 |
| NZ_CP032257.1_1227760..1316873_00079 | 49 | G_tfc9 | typeG | accessory | 7.500e-18 | 57.400 | 0.620 | 1.000 |
| NZ_CP032257.1_1227760..1316873_00080 | 118 | G_tfc10 | typeG | accessory | 6.400e-44 | 141.000 | 0.875 | 0.890 |
| NZ_CP032257.1_1227760..1316873_00081 | 128 | G_tfc11 | typeG | accessory | 4.500e-50 | 161.300 | 0.923 | 0.938 |
| NZ_CP032257.1_1227760..1316873_00082 | 219 | G_tfc12 | typeG | accessory | 7.100e-92 | 299.200 | 0.950 | 0.968 |
| NZ_CP032257.1_1227760..1316873_00083 | 294 | G_tfc13 | typeG | accessory | 1.500e-118 | 387.800 | 0.955 | 0.956 |
| NZ_CP032257.1_1227760..1316873_00084 | 501 | G_tfc14 | typeG | accessory | 9.800e-185 | 607.800 | 0.996 | 0.992 |
| NZ_CP032257.1_1227760..1316873_00085 | 147 | G_tfc15 | typeG | accessory | 3.400e-60 | 194.800 | 0.957 | 0.925 |
| NZ_CP032257.1_1227760..1316873_00086 | 980 | virb4 | typeG | mandatory | 8.200e-90 | 295.000 | 0.807 | 0.899 |
| NZ_CP032257.1_1227760..1316873_00091 | 143 | G_tfc24 | typeG | accessory | 3.900e-51 | 165.300 | 0.914 | 0.895 |
| NZ_CP032257.1_1227760..1316873_00092 | 312 | G_tfc23 | typeG | accessory | 9.600e-134 | 438.300 | 0.973 | 0.987 |
| NZ_CP032257.1_1227760..1316873_00093 | 462 | G_tfc22 | typeG | accessory | 4.100e-180 | 591.900 | 0.987 | 0.970 |
| NZ_CP032257.1_1227760..1316873_00094 | 115 | G_tfc18 | typeG | accessory | 3.200e-29 | 93.800 | 0.912 | 0.887 |
| NZ_CP032257.1_1227760..1316873_00095 | 508 | G_tfc19 | typeG | accessory | 1.100e-220 | 726.100 | 0.992 | 0.990 |
| NZ_CP032257.1_1227760..1316873_00102 | 640 | MOBH | typeG | mandatory | 2.700e-44 | 143.900 | 0.868 | 0.267 |
| NZ_CP032552.1_5459604..5545649_00041 | 370 | G_tfc2 | typeG | accessory | 1.100e-71 | 233.300 | 0.901 | 0.692 |
| NZ_CP032552.1_5459604..5545649_00071 | 251 | G_tfc3 | typeG | accessory | 1.300e-95 | 311.700 | 0.967 | 0.976 |
| NZ_CP032552.1_5459604..5545649_00073 | 166 | G_tfc5 | typeG | accessory | 4.200e-57 | 185.300 | 0.971 | 0.994 |
| NZ_CP032552.1_5459604..5545649_00075 | 743 | t4cp2 | typeG | mandatory | 4.100e-31 | 100.700 | 0.955 | 0.318 |
| NZ_CP032552.1_5459604..5545649_00076 | 248 | G_tfc7 | typeG | accessory | 1.000e-103 | 339.000 | 1.000 | 1.000 |
| NZ_CP032552.1_5459604..5545649_00082 | 112 | G_tfc8 | typeG | accessory | 1.700e-41 | 133.500 | 0.931 | 0.938 |
| NZ_CP032552.1_5459604..5545649_00083 | 79 | G_tfc9 | typeG | accessory | 1.200e-30 | 98.500 | 1.000 | 1.000 |
| NZ_CP032552.1_5459604..5545649_00084 | 118 | G_tfc10 | typeG | accessory | 4.000e-44 | 141.700 | 0.925 | 0.924 |
| NZ_CP032552.1_5459604..5545649_00085 | 128 | G_tfc11 | typeG | accessory | 6.800e-50 | 160.800 | 0.923 | 0.938 |
| NZ_CP032552.1_5459604..5545649_00086 | 219 | G_tfc12 | typeG | accessory | 7.700e-91 | 295.900 | 0.941 | 0.959 |
| NZ_CP032552.1_5459604..5545649_00087 | 294 | G_tfc13 | typeG | accessory | 3.500e-118 | 386.700 | 0.944 | 0.959 |
| NZ_CP032552.1_5459604..5545649_00088 | 501 | G_tfc14 | typeG | accessory | 8.100e-184 | 604.800 | 0.996 | 0.992 |
| NZ_CP032552.1_5459604..5545649_00089 | 147 | G_tfc15 | typeG | accessory | 3.700e-60 | 194.700 | 0.957 | 0.925 |
| NZ_CP032552.1_5459604..5545649_00090 | 980 | virb4 | typeG | mandatory | 8.600e-90 | 295.000 | 0.807 | 0.899 |
| NZ_CP032552.1_5459604..5545649_00096 | 143 | G_tfc24 | typeG | accessory | 1.700e-50 | 163.300 | 0.914 | 0.895 |
| NZ_CP032552.1_5459604..5545649_00097 | 312 | G_tfc23 | typeG | accessory | 1.100e-133 | 438.200 | 0.973 | 0.987 |
| NZ_CP032552.1_5459604..5545649_00098 | 462 | G_tfc22 | typeG | accessory | 6.600e-180 | 591.300 | 0.987 | 0.970 |
| NZ_CP032552.1_5459604..5545649_00099 | 114 | G_tfc18 | typeG | accessory | 1.000e-27 | 89.000 | 0.912 | 0.886 |
| NZ_CP032552.1_5459604..5545649_00100 | 510 | G_tfc19 | typeG | accessory | 1.900e-221 | 728.700 | 0.992 | 0.986 |
| NZ_CP032552.1_5459604..5545649_00107 | 640 | MOBH | typeG | mandatory | 2.800e-44 | 143.900 | 0.868 | 0.267 |
| NZ_CP032569.1_5058369..5152012_00041 | 374 | G_tfc2 | typeG | accessory | 2.200e-71 | 232.400 | 0.782 | 0.570 |
| NZ_CP032569.1_5058369..5152012_00072 | 251 | G_tfc3 | typeG | accessory | 3.700e-96 | 313.600 | 0.988 | 0.996 |
| NZ_CP032569.1_5058369..5152012_00074 | 166 | G_tfc5 | typeG | accessory | 2.600e-57 | 186.100 | 0.971 | 0.994 |
| NZ_CP032569.1_5058369..5152012_00076 | 743 | t4cp2 | typeG | mandatory | 4.400e-31 | 100.700 | 0.955 | 0.318 |
| NZ_CP032569.1_5058369..5152012_00077 | 249 | G_tfc7 | typeG | accessory | 1.600e-103 | 338.500 | 1.000 | 0.996 |
| NZ_CP032569.1_5058369..5152012_00086 | 112 | G_tfc8 | typeG | accessory | 1.400e-40 | 130.600 | 0.914 | 0.920 |
| NZ_CP032569.1_5058369..5152012_00087 | 79 | G_tfc9 | typeG | accessory | 1.100e-30 | 98.700 | 1.000 | 1.000 |
| NZ_CP032569.1_5058369..5152012_00088 | 118 | G_tfc10 | typeG | accessory | 7.100e-44 | 141.000 | 0.875 | 0.890 |
| NZ_CP032569.1_5058369..5152012_00089 | 128 | G_tfc11 | typeG | accessory | 5.100e-50 | 161.300 | 0.923 | 0.938 |
| NZ_CP032569.1_5058369..5152012_00090 | 219 | G_tfc12 | typeG | accessory | 7.900e-92 | 299.200 | 0.950 | 0.968 |
| NZ_CP032569.1_5058369..5152012_00091 | 294 | G_tfc13 | typeG | accessory | 1.700e-118 | 387.800 | 0.955 | 0.956 |
| NZ_CP032569.1_5058369..5152012_00092 | 501 | G_tfc14 | typeG | accessory | 1.100e-184 | 607.800 | 0.996 | 0.992 |
| NZ_CP032569.1_5058369..5152012_00093 | 147 | G_tfc15 | typeG | accessory | 3.800e-60 | 194.800 | 0.957 | 0.925 |
| NZ_CP032569.1_5058369..5152012_00094 | 980 | virb4 | typeG | mandatory | 9.100e-90 | 295.000 | 0.807 | 0.899 |
| NZ_CP032569.1_5058369..5152012_00100 | 143 | G_tfc24 | typeG | accessory | 4.400e-51 | 165.300 | 0.914 | 0.895 |
| NZ_CP032569.1_5058369..5152012_00101 | 312 | G_tfc23 | typeG | accessory | 1.100e-133 | 438.300 | 0.973 | 0.987 |
| NZ_CP032569.1_5058369..5152012_00102 | 545 | G_tfc22 | typeG | accessory | 8.100e-181 | 594.400 | 0.987 | 0.822 |
| NZ_CP032569.1_5058369..5152012_00103 | 508 | G_tfc19 | typeG | accessory | 1.300e-220 | 726.100 | 0.992 | 0.990 |
| NZ_CP032569.1_5058369..5152012_00114 | 640 | MOBH | typeG | mandatory | 3.000e-44 | 143.900 | 0.868 | 0.267 |
| NZ_CP033439.1_1065389..1155743_00002 | 640 | MOBH | typeG | mandatory | 2.700e-44 | 143.900 | 0.868 | 0.267 |
| NZ_CP033439.1_1065389..1155743_00009 | 511 | G_tfc19 | typeG | accessory | 7.500e-223 | 733.300 | 0.990 | 0.982 |
| NZ_CP033439.1_1065389..1155743_00010 | 114 | G_tfc18 | typeG | accessory | 1.000e-27 | 89.000 | 0.912 | 0.886 |
| NZ_CP033439.1_1065389..1155743_00011 | 462 | G_tfc22 | typeG | accessory | 6.500e-180 | 591.300 | 0.987 | 0.970 |
| NZ_CP033439.1_1065389..1155743_00012 | 312 | G_tfc23 | typeG | accessory | 1.000e-133 | 438.200 | 0.973 | 0.987 |
| NZ_CP033439.1_1065389..1155743_00013 | 143 | G_tfc24 | typeG | accessory | 1.600e-50 | 163.300 | 0.914 | 0.895 |
| NZ_CP033439.1_1065389..1155743_00019 | 980 | virb4 | typeG | mandatory | 6.900e-90 | 295.300 | 0.807 | 0.899 |
| NZ_CP033439.1_1065389..1155743_00020 | 147 | G_tfc15 | typeG | accessory | 3.700e-60 | 194.700 | 0.957 | 0.925 |
| NZ_CP033439.1_1065389..1155743_00021 | 501 | G_tfc14 | typeG | accessory | 1.000e-184 | 607.800 | 0.996 | 0.992 |
| NZ_CP033439.1_1065389..1155743_00022 | 294 | G_tfc13 | typeG | accessory | 1.600e-118 | 387.800 | 0.955 | 0.956 |
| NZ_CP033439.1_1065389..1155743_00023 | 219 | G_tfc12 | typeG | accessory | 7.300e-92 | 299.200 | 0.950 | 0.968 |
| NZ_CP033439.1_1065389..1155743_00024 | 128 | G_tfc11 | typeG | accessory | 4.700e-50 | 161.300 | 0.923 | 0.938 |
| NZ_CP033439.1_1065389..1155743_00025 | 118 | G_tfc10 | typeG | accessory | 6.500e-44 | 141.000 | 0.875 | 0.890 |
| NZ_CP033439.1_1065389..1155743_00026 | 79 | G_tfc9 | typeG | accessory | 1.000e-30 | 98.700 | 1.000 | 1.000 |
| NZ_CP033439.1_1065389..1155743_00027 | 112 | G_tfc8 | typeG | accessory | 3.000e-40 | 129.500 | 0.931 | 0.938 |
| NZ_CP033439.1_1065389..1155743_00031 | 248 | G_tfc7 | typeG | accessory | 6.600e-104 | 339.600 | 1.000 | 1.000 |
| NZ_CP033439.1_1065389..1155743_00032 | 743 | t4cp2 | typeG | mandatory | 4.000e-31 | 100.700 | 0.955 | 0.318 |
| NZ_CP033439.1_1065389..1155743_00034 | 166 | G_tfc5 | typeG | accessory | 2.400e-57 | 186.100 | 0.971 | 0.994 |
| NZ_CP033439.1_1065389..1155743_00036 | 251 | G_tfc3 | typeG | accessory | 3.400e-96 | 313.600 | 0.988 | 0.996 |
| NZ_CP033684.1_5743498..5837210_00080 | 251 | G_tfc3 | typeG | accessory | 3.900e-96 | 313.600 | 0.988 | 0.996 |
| NZ_CP033684.1_5743498..5837210_00082 | 166 | G_tfc5 | typeG | accessory | 2.800e-57 | 186.100 | 0.971 | 0.994 |
| NZ_CP033684.1_5743498..5837210_00084 | 743 | t4cp2 | typeG | mandatory | 4.600e-31 | 100.700 | 0.955 | 0.318 |
| NZ_CP033684.1_5743498..5837210_00085 | 249 | G_tfc7 | typeG | accessory | 1.100e-103 | 339.100 | 1.000 | 0.996 |
| NZ_CP033684.1_5743498..5837210_00091 | 112 | G_tfc8 | typeG | accessory | 1.500e-40 | 130.600 | 0.914 | 0.920 |
| NZ_CP033684.1_5743498..5837210_00092 | 79 | G_tfc9 | typeG | accessory | 1.200e-30 | 98.700 | 1.000 | 1.000 |
| NZ_CP033684.1_5743498..5837210_00093 | 118 | G_tfc10 | typeG | accessory | 3.200e-44 | 142.200 | 0.875 | 0.890 |
| NZ_CP033684.1_5743498..5837210_00094 | 128 | G_tfc11 | typeG | accessory | 9.200e-50 | 160.500 | 0.923 | 0.938 |
| NZ_CP033684.1_5743498..5837210_00095 | 219 | G_tfc12 | typeG | accessory | 6.800e-91 | 296.200 | 0.941 | 0.959 |
| NZ_CP033684.1_5743498..5837210_00096 | 294 | G_tfc13 | typeG | accessory | 8.000e-119 | 389.000 | 0.944 | 0.959 |
| NZ_CP033684.1_5743498..5837210_00097 | 501 | G_tfc14 | typeG | accessory | 1.500e-184 | 607.400 | 0.996 | 0.992 |
| NZ_CP033684.1_5743498..5837210_00098 | 147 | G_tfc15 | typeG | accessory | 7.700e-60 | 193.900 | 0.957 | 0.925 |
| NZ_CP033684.1_5743498..5837210_00099 | 980 | virb4 | typeG | mandatory | 6.200e-91 | 298.900 | 0.807 | 0.899 |
| NZ_CP033684.1_5743498..5837210_00113 | 143 | G_tfc24 | typeG | accessory | 9.000e-51 | 164.400 | 0.907 | 0.888 |
| NZ_CP033684.1_5743498..5837210_00114 | 312 | G_tfc23 | typeG | accessory | 3.500e-134 | 440.000 | 0.973 | 0.987 |
| NZ_CP033684.1_5743498..5837210_00115 | 460 | G_tfc22 | typeG | accessory | 5.200e-180 | 591.800 | 0.987 | 0.974 |
| NZ_CP033684.1_5743498..5837210_00116 | 115 | G_tfc18 | typeG | accessory | 4.100e-29 | 93.700 | 0.912 | 0.887 |
| NZ_CP033684.1_5743498..5837210_00117 | 504 | G_tfc19 | typeG | accessory | 1.500e-222 | 732.500 | 0.984 | 0.990 |
| NZ_CP033684.1_5743498..5837210_00121 | 639 | MOBH | typeG | mandatory | 4.400e-44 | 143.400 | 0.868 | 0.268 |
| NZ_CP033832.1_37920..127485_00036 | 374 | G_tfc2 | typeG | accessory | 8.600e-72 | 233.500 | 0.782 | 0.580 |
| NZ_CP033832.1_37920..127485_00064 | 251 | G_tfc3 | typeG | accessory | 3.200e-96 | 313.600 | 0.988 | 0.996 |
| NZ_CP033832.1_37920..127485_00066 | 166 | G_tfc5 | typeG | accessory | 3.300e-56 | 182.300 | 0.971 | 0.994 |
| NZ_CP033832.1_37920..127485_00068 | 743 | t4cp2 | typeG | mandatory | 3.800e-31 | 100.700 | 0.955 | 0.318 |
| NZ_CP033832.1_37920..127485_00069 | 248 | G_tfc7 | typeG | accessory | 8.200e-104 | 339.200 | 1.000 | 1.000 |
| NZ_CP033832.1_37920..127485_00074 | 112 | G_tfc8 | typeG | accessory | 1.300e-40 | 130.600 | 0.914 | 0.920 |
| NZ_CP033832.1_37920..127485_00075 | 49 | G_tfc9 | typeG | accessory | 7.300e-18 | 57.400 | 0.620 | 1.000 |
| NZ_CP033832.1_37920..127485_00076 | 118 | G_tfc10 | typeG | accessory | 6.200e-44 | 141.000 | 0.875 | 0.890 |
| NZ_CP033832.1_37920..127485_00077 | 128 | G_tfc11 | typeG | accessory | 4.400e-50 | 161.300 | 0.923 | 0.938 |
| NZ_CP033832.1_37920..127485_00078 | 219 | G_tfc12 | typeG | accessory | 6.900e-92 | 299.200 | 0.950 | 0.968 |
| NZ_CP033832.1_37920..127485_00079 | 294 | G_tfc13 | typeG | accessory | 1.500e-118 | 387.800 | 0.955 | 0.956 |
| NZ_CP033832.1_37920..127485_00080 | 501 | G_tfc14 | typeG | accessory | 9.600e-185 | 607.800 | 0.996 | 0.992 |
| NZ_CP033832.1_37920..127485_00081 | 147 | G_tfc15 | typeG | accessory | 3.500e-60 | 194.700 | 0.957 | 0.925 |
| NZ_CP033832.1_37920..127485_00082 | 980 | virb4 | typeG | mandatory | 1.100e-90 | 297.900 | 0.806 | 0.898 |
| NZ_CP033832.1_37920..127485_00094 | 143 | G_tfc24 | typeG | accessory | 7.500e-51 | 164.400 | 0.907 | 0.888 |
| NZ_CP033832.1_37920..127485_00095 | 312 | G_tfc23 | typeG | accessory | 2.900e-134 | 440.000 | 0.973 | 0.987 |
| NZ_CP033832.1_37920..127485_00096 | 460 | G_tfc22 | typeG | accessory | 1.300e-179 | 590.200 | 0.991 | 0.978 |
| NZ_CP033832.1_37920..127485_00097 | 115 | G_tfc18 | typeG | accessory | 4.500e-29 | 93.300 | 0.912 | 0.896 |
| NZ_CP033832.1_37920..127485_00098 | 514 | G_tfc19 | typeG | accessory | 8.000e-223 | 733.100 | 0.992 | 0.979 |
| NZ_CP033832.1_37920..127485_00100 | 639 | MOBH | typeG | mandatory | 2.600e-44 | 143.900 | 0.868 | 0.268 |
| NZ_CP033835.1_1337211..1429410_00002 | 640 | MOBH | typeG | mandatory | 2.700e-44 | 143.900 | 0.868 | 0.267 |
| NZ_CP033835.1_1337211..1429410_00009 | 508 | G_tfc19 | typeG | accessory | 1.100e-220 | 726.100 | 0.992 | 0.990 |
| NZ_CP033835.1_1337211..1429410_00010 | 115 | G_tfc18 | typeG | accessory | 3.200e-29 | 93.800 | 0.912 | 0.887 |
| NZ_CP033835.1_1337211..1429410_00011 | 462 | G_tfc22 | typeG | accessory | 4.100e-180 | 591.900 | 0.987 | 0.970 |
| NZ_CP033835.1_1337211..1429410_00012 | 312 | G_tfc23 | typeG | accessory | 9.600e-134 | 438.300 | 0.973 | 0.987 |
| NZ_CP033835.1_1337211..1429410_00013 | 143 | G_tfc24 | typeG | accessory | 3.900e-51 | 165.300 | 0.914 | 0.895 |
| NZ_CP033835.1_1337211..1429410_00019 | 980 | virb4 | typeG | mandatory | 1.100e-89 | 294.600 | 0.808 | 0.900 |
| NZ_CP033835.1_1337211..1429410_00020 | 147 | G_tfc15 | typeG | accessory | 6.600e-60 | 193.900 | 0.957 | 0.925 |
| NZ_CP033835.1_1337211..1429410_00021 | 501 | G_tfc14 | typeG | accessory | 5.700e-185 | 608.600 | 0.996 | 0.992 |
| NZ_CP033835.1_1337211..1429410_00022 | 294 | G_tfc13 | typeG | accessory | 1.900e-118 | 387.500 | 0.941 | 0.956 |
| NZ_CP033835.1_1337211..1429410_00023 | 219 | G_tfc12 | typeG | accessory | 7.300e-91 | 295.900 | 0.941 | 0.959 |
| NZ_CP033835.1_1337211..1429410_00024 | 128 | G_tfc11 | typeG | accessory | 6.500e-50 | 160.800 | 0.923 | 0.938 |
| NZ_CP033835.1_1337211..1429410_00025 | 118 | G_tfc10 | typeG | accessory | 3.800e-44 | 141.700 | 0.925 | 0.924 |
| NZ_CP033835.1_1337211..1429410_00026 | 79 | G_tfc9 | typeG | accessory | 1.200e-30 | 98.500 | 1.000 | 1.000 |
| NZ_CP033835.1_1337211..1429410_00027 | 112 | G_tfc8 | typeG | accessory | 1.600e-41 | 133.500 | 0.931 | 0.938 |
| NZ_CP033835.1_1337211..1429410_00033 | 249 | G_tfc7 | typeG | accessory | 1.400e-103 | 338.500 | 1.000 | 0.996 |
| NZ_CP033835.1_1337211..1429410_00034 | 743 | t4cp2 | typeG | mandatory | 3.900e-31 | 100.700 | 0.955 | 0.318 |
| NZ_CP033835.1_1337211..1429410_00036 | 166 | G_tfc5 | typeG | accessory | 2.300e-57 | 186.100 | 0.971 | 0.994 |
| NZ_CP033835.1_1337211..1429410_00038 | 251 | G_tfc3 | typeG | accessory | 3.300e-96 | 313.600 | 0.988 | 0.996 |
| NZ_CP033835.1_1337211..1429410_00068 | 374 | G_tfc2 | typeG | accessory | 2.000e-71 | 232.400 | 0.782 | 0.570 |
| NZ_CP034354.1_910412..1013390_00068 | 251 | G_tfc3 | typeG | accessory | 6.000e-96 | 312.800 | 0.988 | 0.996 |
| NZ_CP034354.1_910412..1013390_00070 | 166 | G_tfc5 | typeG | accessory | 1.300e-55 | 180.500 | 0.977 | 1.000 |
| NZ_CP034354.1_910412..1013390_00072 | 743 | t4cp2 | typeG | mandatory | 4.000e-31 | 100.700 | 0.955 | 0.318 |
| NZ_CP034354.1_910412..1013390_00073 | 248 | G_tfc7 | typeG | accessory | 8.400e-104 | 339.200 | 1.000 | 1.000 |
| NZ_CP034354.1_910412..1013390_00078 | 112 | G_tfc8 | typeG | accessory | 2.000e-40 | 130.000 | 0.922 | 0.929 |
| NZ_CP034354.1_910412..1013390_00079 | 79 | G_tfc9 | typeG | accessory | 8.000e-31 | 99.000 | 1.000 | 1.000 |
| NZ_CP034354.1_910412..1013390_00080 | 118 | G_tfc10 | typeG | accessory | 6.400e-45 | 144.300 | 0.933 | 0.932 |
| NZ_CP034354.1_910412..1013390_00081 | 128 | G_tfc11 | typeG | accessory | 3.700e-50 | 161.600 | 0.923 | 0.938 |
| NZ_CP034354.1_910412..1013390_00082 | 219 | G_tfc12 | typeG | accessory | 2.500e-91 | 297.400 | 0.950 | 0.968 |
| NZ_CP034354.1_910412..1013390_00083 | 294 | G_tfc13 | typeG | accessory | 5.100e-118 | 386.100 | 0.944 | 0.956 |
| NZ_CP034354.1_910412..1013390_00084 | 501 | G_tfc14 | typeG | accessory | 1.200e-184 | 607.600 | 0.996 | 0.992 |
| NZ_CP034354.1_910412..1013390_00085 | 147 | G_tfc15 | typeG | accessory | 8.400e-60 | 193.600 | 0.957 | 0.925 |
| NZ_CP034354.1_910412..1013390_00086 | 980 | virb4 | typeG | mandatory | 8.800e-91 | 298.200 | 0.807 | 0.899 |
| NZ_CP034354.1_910412..1013390_00090 | 312 | G_tfc23 | typeG | accessory | 8.200e-134 | 438.600 | 0.973 | 0.987 |
| NZ_CP034354.1_910412..1013390_00091 | 460 | G_tfc22 | typeG | accessory | 8.000e-180 | 591.000 | 0.987 | 0.974 |
| NZ_CP034354.1_910412..1013390_00092 | 115 | G_tfc18 | typeG | accessory | 5.300e-29 | 93.100 | 0.912 | 0.887 |
| NZ_CP034354.1_910412..1013390_00093 | 504 | G_tfc19 | typeG | accessory | 1.600e-222 | 732.200 | 0.984 | 0.990 |
| NZ_CP034354.1_910412..1013390_00104 | 608 | MOBH | typeG | mandatory | 1.900e-43 | 141.100 | 0.843 | 0.270 |
| NZ_CP034369.1_4706492..4795934_00071 | 251 | G_tfc3 | typeG | accessory | 3.400e-96 | 313.600 | 0.988 | 0.996 |
| NZ_CP034369.1_4706492..4795934_00073 | 166 | G_tfc5 | typeG | accessory | 2.400e-57 | 186.100 | 0.971 | 0.994 |
| NZ_CP034369.1_4706492..4795934_00075 | 743 | t4cp2 | typeG | mandatory | 4.000e-31 | 100.700 | 0.955 | 0.318 |
| NZ_CP034369.1_4706492..4795934_00076 | 248 | G_tfc7 | typeG | accessory | 6.600e-104 | 339.600 | 1.000 | 1.000 |
| NZ_CP034369.1_4706492..4795934_00080 | 112 | G_tfc8 | typeG | accessory | 3.000e-40 | 129.500 | 0.931 | 0.938 |
| NZ_CP034369.1_4706492..4795934_00081 | 79 | G_tfc9 | typeG | accessory | 1.000e-30 | 98.700 | 1.000 | 1.000 |
| NZ_CP034369.1_4706492..4795934_00082 | 118 | G_tfc10 | typeG | accessory | 6.500e-44 | 141.000 | 0.875 | 0.890 |
| NZ_CP034369.1_4706492..4795934_00083 | 128 | G_tfc11 | typeG | accessory | 4.700e-50 | 161.300 | 0.923 | 0.938 |
| NZ_CP034369.1_4706492..4795934_00084 | 219 | G_tfc12 | typeG | accessory | 7.300e-92 | 299.200 | 0.950 | 0.968 |
| NZ_CP034369.1_4706492..4795934_00085 | 294 | G_tfc13 | typeG | accessory | 1.600e-118 | 387.800 | 0.955 | 0.956 |
| NZ_CP034369.1_4706492..4795934_00086 | 501 | G_tfc14 | typeG | accessory | 1.000e-184 | 607.800 | 0.996 | 0.992 |
| NZ_CP034369.1_4706492..4795934_00087 | 147 | G_tfc15 | typeG | accessory | 3.700e-60 | 194.700 | 0.957 | 0.925 |
| NZ_CP034369.1_4706492..4795934_00088 | 980 | virb4 | typeG | mandatory | 6.900e-90 | 295.300 | 0.807 | 0.899 |
| NZ_CP034369.1_4706492..4795934_00094 | 143 | G_tfc24 | typeG | accessory | 1.600e-50 | 163.300 | 0.914 | 0.895 |
| NZ_CP034369.1_4706492..4795934_00095 | 312 | G_tfc23 | typeG | accessory | 1.000e-133 | 438.200 | 0.973 | 0.987 |
| NZ_CP034369.1_4706492..4795934_00096 | 462 | G_tfc22 | typeG | accessory | 6.500e-180 | 591.300 | 0.987 | 0.970 |
| NZ_CP034369.1_4706492..4795934_00097 | 114 | G_tfc18 | typeG | accessory | 1.000e-27 | 89.000 | 0.912 | 0.886 |
| NZ_CP034369.1_4706492..4795934_00098 | 511 | G_tfc19 | typeG | accessory | 7.500e-223 | 733.300 | 0.990 | 0.982 |
| NZ_CP034369.1_4706492..4795934_00105 | 640 | MOBH | typeG | mandatory | 2.700e-44 | 143.900 | 0.868 | 0.267 |
| NZ_CP034409.1_3802866..3893221_00072 | 251 | G_tfc3 | typeG | accessory | 3.400e-96 | 313.600 | 0.988 | 0.996 |
| NZ_CP034409.1_3802866..3893221_00074 | 166 | G_tfc5 | typeG | accessory | 2.400e-57 | 186.100 | 0.971 | 0.994 |
| NZ_CP034409.1_3802866..3893221_00076 | 743 | t4cp2 | typeG | mandatory | 4.100e-31 | 100.700 | 0.955 | 0.318 |
| NZ_CP034409.1_3802866..3893221_00077 | 248 | G_tfc7 | typeG | accessory | 6.700e-104 | 339.600 | 1.000 | 1.000 |
| NZ_CP034409.1_3802866..3893221_00081 | 112 | G_tfc8 | typeG | accessory | 3.000e-40 | 129.500 | 0.931 | 0.938 |
| NZ_CP034409.1_3802866..3893221_00082 | 79 | G_tfc9 | typeG | accessory | 1.100e-30 | 98.700 | 1.000 | 1.000 |
| NZ_CP034409.1_3802866..3893221_00083 | 118 | G_tfc10 | typeG | accessory | 6.600e-44 | 141.000 | 0.875 | 0.890 |
| NZ_CP034409.1_3802866..3893221_00084 | 128 | G_tfc11 | typeG | accessory | 4.700e-50 | 161.300 | 0.923 | 0.938 |
| NZ_CP034409.1_3802866..3893221_00085 | 219 | G_tfc12 | typeG | accessory | 7.400e-92 | 299.200 | 0.950 | 0.968 |
| NZ_CP034409.1_3802866..3893221_00086 | 294 | G_tfc13 | typeG | accessory | 1.600e-118 | 387.800 | 0.955 | 0.956 |
| NZ_CP034409.1_3802866..3893221_00087 | 501 | G_tfc14 | typeG | accessory | 1.000e-184 | 607.800 | 0.996 | 0.992 |
| NZ_CP034409.1_3802866..3893221_00088 | 147 | G_tfc15 | typeG | accessory | 3.700e-60 | 194.700 | 0.957 | 0.925 |
| NZ_CP034409.1_3802866..3893221_00089 | 980 | virb4 | typeG | mandatory | 7.000e-90 | 295.300 | 0.807 | 0.899 |
| NZ_CP034409.1_3802866..3893221_00095 | 143 | G_tfc24 | typeG | accessory | 1.700e-50 | 163.300 | 0.914 | 0.895 |
| NZ_CP034409.1_3802866..3893221_00096 | 312 | G_tfc23 | typeG | accessory | 1.100e-133 | 438.200 | 0.973 | 0.987 |
| NZ_CP034409.1_3802866..3893221_00097 | 462 | G_tfc22 | typeG | accessory | 6.500e-180 | 591.300 | 0.987 | 0.970 |
| NZ_CP034409.1_3802866..3893221_00098 | 114 | G_tfc18 | typeG | accessory | 1.000e-27 | 89.000 | 0.912 | 0.886 |
| NZ_CP034409.1_3802866..3893221_00099 | 511 | G_tfc19 | typeG | accessory | 7.600e-223 | 733.300 | 0.990 | 0.982 |
| NZ_CP034409.1_3802866..3893221_00106 | 640 | MOBH | typeG | mandatory | 2.800e-44 | 143.900 | 0.868 | 0.267 |
| NZ_CP034435.1_1001650..1092003_00002 | 640 | MOBH | typeG | mandatory | 2.700e-44 | 143.900 | 0.868 | 0.267 |
| NZ_CP034435.1_1001650..1092003_00009 | 511 | G_tfc19 | typeG | accessory | 7.500e-223 | 733.300 | 0.990 | 0.982 |
| NZ_CP034435.1_1001650..1092003_00010 | 114 | G_tfc18 | typeG | accessory | 1.000e-27 | 89.000 | 0.912 | 0.886 |
| NZ_CP034435.1_1001650..1092003_00011 | 462 | G_tfc22 | typeG | accessory | 6.500e-180 | 591.300 | 0.987 | 0.970 |
| NZ_CP034435.1_1001650..1092003_00012 | 312 | G_tfc23 | typeG | accessory | 1.000e-133 | 438.200 | 0.973 | 0.987 |
| NZ_CP034435.1_1001650..1092003_00013 | 143 | G_tfc24 | typeG | accessory | 1.600e-50 | 163.300 | 0.914 | 0.895 |
| NZ_CP034435.1_1001650..1092003_00019 | 980 | virb4 | typeG | mandatory | 6.900e-90 | 295.300 | 0.807 | 0.899 |
| NZ_CP034435.1_1001650..1092003_00020 | 147 | G_tfc15 | typeG | accessory | 3.700e-60 | 194.700 | 0.957 | 0.925 |
| NZ_CP034435.1_1001650..1092003_00021 | 501 | G_tfc14 | typeG | accessory | 1.000e-184 | 607.800 | 0.996 | 0.992 |
| NZ_CP034435.1_1001650..1092003_00022 | 294 | G_tfc13 | typeG | accessory | 1.600e-118 | 387.800 | 0.955 | 0.956 |
| NZ_CP034435.1_1001650..1092003_00023 | 219 | G_tfc12 | typeG | accessory | 7.300e-92 | 299.200 | 0.950 | 0.968 |
| NZ_CP034435.1_1001650..1092003_00024 | 128 | G_tfc11 | typeG | accessory | 4.700e-50 | 161.300 | 0.923 | 0.938 |
| NZ_CP034435.1_1001650..1092003_00025 | 118 | G_tfc10 | typeG | accessory | 6.500e-44 | 141.000 | 0.875 | 0.890 |
| NZ_CP034435.1_1001650..1092003_00026 | 79 | G_tfc9 | typeG | accessory | 1.000e-30 | 98.700 | 1.000 | 1.000 |
| NZ_CP034435.1_1001650..1092003_00027 | 112 | G_tfc8 | typeG | accessory | 3.000e-40 | 129.500 | 0.931 | 0.938 |
| NZ_CP034435.1_1001650..1092003_00031 | 248 | G_tfc7 | typeG | accessory | 6.600e-104 | 339.600 | 1.000 | 1.000 |
| NZ_CP034435.1_1001650..1092003_00032 | 743 | t4cp2 | typeG | mandatory | 4.000e-31 | 100.700 | 0.955 | 0.318 |
| NZ_CP034435.1_1001650..1092003_00034 | 166 | G_tfc5 | typeG | accessory | 2.400e-57 | 186.100 | 0.971 | 0.994 |
| NZ_CP034435.1_1001650..1092003_00036 | 251 | G_tfc3 | typeG | accessory | 3.400e-96 | 313.600 | 0.988 | 0.996 |
| NZ_CP039293.1_5578776..5666623_00077 | 251 | G_tfc3 | typeG | accessory | 3.700e-96 | 313.600 | 0.988 | 0.996 |
| NZ_CP039293.1_5578776..5666623_00079 | 166 | G_tfc5 | typeG | accessory | 2.600e-57 | 186.100 | 0.971 | 0.994 |
| NZ_CP039293.1_5578776..5666623_00081 | 743 | t4cp2 | typeG | mandatory | 4.400e-31 | 100.700 | 0.955 | 0.318 |
| NZ_CP039293.1_5578776..5666623_00082 | 249 | G_tfc7 | typeG | accessory | 1.000e-103 | 339.100 | 1.000 | 0.996 |
| NZ_CP039293.1_5578776..5666623_00088 | 112 | G_tfc8 | typeG | accessory | 1.400e-40 | 130.600 | 0.914 | 0.920 |
| NZ_CP039293.1_5578776..5666623_00089 | 79 | G_tfc9 | typeG | accessory | 1.100e-30 | 98.700 | 1.000 | 1.000 |
| NZ_CP039293.1_5578776..5666623_00090 | 118 | G_tfc10 | typeG | accessory | 7.100e-44 | 141.000 | 0.875 | 0.890 |
| NZ_CP039293.1_5578776..5666623_00091 | 128 | G_tfc11 | typeG | accessory | 5.100e-50 | 161.300 | 0.923 | 0.938 |
| NZ_CP039293.1_5578776..5666623_00092 | 219 | G_tfc12 | typeG | accessory | 7.900e-92 | 299.200 | 0.950 | 0.968 |
| NZ_CP039293.1_5578776..5666623_00093 | 294 | G_tfc13 | typeG | accessory | 5.500e-119 | 389.400 | 0.955 | 0.956 |
| NZ_CP039293.1_5578776..5666623_00094 | 501 | G_tfc14 | typeG | accessory | 1.600e-184 | 607.300 | 0.990 | 0.984 |
| NZ_CP039293.1_5578776..5666623_00095 | 147 | G_tfc15 | typeG | accessory | 1.100e-60 | 196.600 | 0.957 | 0.925 |
| NZ_CP039293.1_5578776..5666623_00096 | 980 | virb4 | typeG | mandatory | 1.700e-90 | 297.400 | 0.807 | 0.899 |
| NZ_CP039293.1_5578776..5666623_00102 | 143 | G_tfc24 | typeG | accessory | 5.900e-51 | 164.900 | 0.914 | 0.895 |
| NZ_CP039293.1_5578776..5666623_00103 | 312 | G_tfc23 | typeG | accessory | 2.400e-134 | 440.400 | 0.973 | 0.987 |
| NZ_CP039293.1_5578776..5666623_00104 | 460 | G_tfc22 | typeG | accessory | 5.700e-180 | 591.600 | 0.987 | 0.974 |
| NZ_CP039293.1_5578776..5666623_00105 | 115 | G_tfc18 | typeG | accessory | 4.000e-29 | 93.600 | 0.912 | 0.887 |
| NZ_CP039293.1_5578776..5666623_00106 | 503 | G_tfc19 | typeG | accessory | 7.400e-222 | 730.100 | 0.984 | 0.992 |
| NZ_CP039293.1_5578776..5666623_00114 | 639 | MOBH | typeG | mandatory | 3.000e-44 | 143.900 | 0.868 | 0.268 |
| NZ_CP039988.1_5454570..5547141_00070 | 251 | G_tfc3 | typeG | accessory | 5.700e-95 | 309.600 | 0.988 | 0.996 |
| NZ_CP039988.1_5454570..5547141_00072 | 166 | G_tfc5 | typeG | accessory | 1.300e-56 | 183.700 | 0.977 | 1.000 |
| NZ_CP039988.1_5454570..5547141_00074 | 743 | t4cp2 | typeG | mandatory | 6.300e-31 | 100.100 | 0.955 | 0.318 |
| NZ_CP039988.1_5454570..5547141_00075 | 248 | G_tfc7 | typeG | accessory | 8.700e-104 | 339.200 | 1.000 | 1.000 |
| NZ_CP039988.1_5454570..5547141_00081 | 112 | G_tfc8 | typeG | accessory | 6.700e-41 | 131.600 | 0.922 | 0.929 |
| NZ_CP039988.1_5454570..5547141_00082 | 79 | G_tfc9 | typeG | accessory | 3.900e-31 | 100.100 | 1.000 | 1.000 |
| NZ_CP039988.1_5454570..5547141_00083 | 118 | G_tfc10 | typeG | accessory | 3.300e-44 | 142.000 | 0.925 | 0.924 |
| NZ_CP039988.1_5454570..5547141_00084 | 128 | G_tfc11 | typeG | accessory | 6.000e-50 | 160.900 | 0.923 | 0.938 |
| NZ_CP039988.1_5454570..5547141_00085 | 219 | G_tfc12 | typeG | accessory | 1.400e-91 | 298.300 | 0.950 | 0.968 |
| NZ_CP039988.1_5454570..5547141_00086 | 294 | G_tfc13 | typeG | accessory | 4.200e-119 | 389.700 | 0.944 | 0.956 |
| NZ_CP039988.1_5454570..5547141_00087 | 501 | G_tfc14 | typeG | accessory | 2.400e-184 | 606.500 | 0.990 | 0.984 |
| NZ_CP039988.1_5454570..5547141_00088 | 147 | G_tfc15 | typeG | accessory | 1.000e-60 | 196.600 | 0.957 | 0.925 |
| NZ_CP039988.1_5454570..5547141_00089 | 980 | virb4 | typeG | mandatory | 1.800e-89 | 293.900 | 0.807 | 0.899 |
| NZ_CP039988.1_5454570..5547141_00095 | 143 | G_tfc24 | typeG | accessory | 2.700e-51 | 165.900 | 0.914 | 0.895 |
| NZ_CP039988.1_5454570..5547141_00096 | 312 | G_tfc23 | typeG | accessory | 2.300e-134 | 440.400 | 0.973 | 0.987 |
| NZ_CP039988.1_5454570..5547141_00097 | 460 | G_tfc22 | typeG | accessory | 4.600e-179 | 588.500 | 0.987 | 0.974 |
| NZ_CP039988.1_5454570..5547141_00098 | 115 | G_tfc18 | typeG | accessory | 6.600e-29 | 92.800 | 0.912 | 0.887 |
| NZ_CP039988.1_5454570..5547141_00099 | 504 | G_tfc19 | typeG | accessory | 4.300e-223 | 734.100 | 0.988 | 0.994 |
| NZ_CP039988.1_5454570..5547141_00106 | 639 | MOBH | typeG | mandatory | 1.400e-43 | 141.500 | 0.868 | 0.268 |
| NZ_CP039990.1_5251404..5335058_00062 | 251 | G_tfc3 | typeG | accessory | 1.000e-95 | 311.900 | 0.988 | 0.996 |
| NZ_CP039990.1_5251404..5335058_00064 | 166 | G_tfc5 | typeG | accessory | 4.200e-57 | 185.200 | 0.971 | 0.994 |
| NZ_CP039990.1_5251404..5335058_00066 | 743 | t4cp2 | typeG | mandatory | 3.600e-31 | 100.700 | 0.955 | 0.318 |
| NZ_CP039990.1_5251404..5335058_00067 | 248 | G_tfc7 | typeG | accessory | 6.400e-104 | 339.500 | 1.000 | 1.000 |
| NZ_CP039990.1_5251404..5335058_00070 | 112 | G_tfc8 | typeG | accessory | 3.000e-40 | 129.300 | 0.922 | 0.929 |
| NZ_CP039990.1_5251404..5335058_00071 | 79 | G_tfc9 | typeG | accessory | 2.800e-31 | 100.400 | 1.000 | 1.000 |
| NZ_CP039990.1_5251404..5335058_00072 | 118 | G_tfc10 | typeG | accessory | 3.600e-44 | 141.700 | 0.925 | 0.924 |
| NZ_CP039990.1_5251404..5335058_00073 | 128 | G_tfc11 | typeG | accessory | 4.200e-50 | 161.300 | 0.923 | 0.938 |
| NZ_CP039990.1_5251404..5335058_00074 | 219 | G_tfc12 | typeG | accessory | 1.700e-91 | 297.800 | 0.941 | 0.959 |
| NZ_CP039990.1_5251404..5335058_00075 | 294 | G_tfc13 | typeG | accessory | 2.100e-118 | 387.300 | 0.944 | 0.959 |
| NZ_CP039990.1_5251404..5335058_00076 | 501 | G_tfc14 | typeG | accessory | 4.300e-185 | 608.900 | 0.996 | 0.992 |
| NZ_CP039990.1_5251404..5335058_00077 | 147 | G_tfc15 | typeG | accessory | 1.900e-60 | 195.500 | 0.957 | 0.925 |
| NZ_CP039990.1_5251404..5335058_00078 | 980 | virb4 | typeG | mandatory | 6.000e-90 | 295.300 | 0.807 | 0.899 |
| NZ_CP039990.1_5251404..5335058_00083 | 143 | G_tfc24 | typeG | accessory | 7.600e-51 | 164.300 | 0.914 | 0.895 |
| NZ_CP039990.1_5251404..5335058_00084 | 312 | G_tfc23 | typeG | accessory | 5.200e-134 | 439.100 | 0.973 | 0.987 |
| NZ_CP039990.1_5251404..5335058_00085 | 460 | G_tfc22 | typeG | accessory | 5.100e-180 | 591.500 | 0.987 | 0.974 |
| NZ_CP039990.1_5251404..5335058_00086 | 115 | G_tfc18 | typeG | accessory | 1.100e-28 | 91.900 | 0.912 | 0.887 |
| NZ_CP039990.1_5251404..5335058_00087 | 518 | G_tfc19 | typeG | accessory | 3.600e-221 | 727.600 | 0.988 | 0.967 |
| NZ_CP039990.1_5251404..5335058_00095 | 639 | MOBH | typeG | mandatory | 1.200e-44 | 144.900 | 0.873 | 0.271 |
| NZ_CP040127.1_5251106..5357222_00083 | 251 | G_tfc3 | typeG | accessory | 3.000e-95 | 310.800 | 0.967 | 0.976 |
| NZ_CP040127.1_5251106..5357222_00085 | 166 | G_tfc5 | typeG | accessory | 2.700e-57 | 186.200 | 0.977 | 1.000 |
| NZ_CP040127.1_5251106..5357222_00087 | 743 | t4cp2 | typeG | mandatory | 5.000e-31 | 100.700 | 0.955 | 0.318 |
| NZ_CP040127.1_5251106..5357222_00088 | 249 | G_tfc7 | typeG | accessory | 1.100e-103 | 339.100 | 1.000 | 0.996 |
| NZ_CP040127.1_5251106..5357222_00094 | 112 | G_tfc8 | typeG | accessory | 1.600e-40 | 130.600 | 0.914 | 0.920 |
| NZ_CP040127.1_5251106..5357222_00095 | 79 | G_tfc9 | typeG | accessory | 1.300e-30 | 98.700 | 1.000 | 1.000 |
| NZ_CP040127.1_5251106..5357222_00096 | 118 | G_tfc10 | typeG | accessory | 8.000e-44 | 141.000 | 0.875 | 0.890 |
| NZ_CP040127.1_5251106..5357222_00097 | 128 | G_tfc11 | typeG | accessory | 5.700e-50 | 161.300 | 0.923 | 0.938 |
| NZ_CP040127.1_5251106..5357222_00098 | 219 | G_tfc12 | typeG | accessory | 8.900e-92 | 299.200 | 0.950 | 0.968 |
| NZ_CP040127.1_5251106..5357222_00099 | 294 | G_tfc13 | typeG | accessory | 4.400e-118 | 386.700 | 0.955 | 0.956 |
| NZ_CP040127.1_5251106..5357222_00100 | 501 | G_tfc14 | typeG | accessory | 1.200e-183 | 604.500 | 0.996 | 0.992 |
| NZ_CP040127.1_5251106..5357222_00101 | 147 | G_tfc15 | typeG | accessory | 4.500e-60 | 194.700 | 0.957 | 0.925 |
| NZ_CP040127.1_5251106..5357222_00102 | 980 | virb4 | typeG | mandatory | 1.400e-90 | 297.800 | 0.806 | 0.898 |
| NZ_CP040127.1_5251106..5357222_00106 | 143 | G_tfc24 | typeG | accessory | 1.000e-50 | 164.300 | 0.907 | 0.888 |
| NZ_CP040127.1_5251106..5357222_00107 | 312 | G_tfc23 | typeG | accessory | 2.100e-133 | 437.500 | 0.973 | 0.987 |
| NZ_CP040127.1_5251106..5357222_00108 | 460 | G_tfc22 | typeG | accessory | 3.200e-180 | 592.600 | 0.987 | 0.974 |
| NZ_CP040127.1_5251106..5357222_00109 | 105 | G_tfc18 | typeG | accessory | 1.000e-19 | 63.500 | 0.895 | 0.867 |
| NZ_CP040127.1_5251106..5357222_00110 | 508 | G_tfc19 | typeG | accessory | 1.000e-220 | 726.600 | 0.992 | 0.990 |
| NZ_CP040127.1_5251106..5357222_00128 | 639 | MOBH | typeG | mandatory | 2.100e-44 | 144.500 | 0.868 | 0.268 |
| NZ_CP040684.1_5922289..6005409_00004 | 629 | MOBH | typeG | mandatory | 2.400e-44 | 143.900 | 0.868 | 0.272 |
| NZ_CP040684.1_5922289..6005409_00007 | 503 | G_tfc19 | typeG | accessory | 2.200e-222 | 731.600 | 0.984 | 0.992 |
| NZ_CP040684.1_5922289..6005409_00008 | 115 | G_tfc18 | typeG | accessory | 2.900e-29 | 93.800 | 0.912 | 0.887 |
| NZ_CP040684.1_5922289..6005409_00009 | 462 | G_tfc22 | typeG | accessory | 2.100e-179 | 589.400 | 0.987 | 0.970 |
| NZ_CP040684.1_5922289..6005409_00010 | 312 | G_tfc23 | typeG | accessory | 4.200e-133 | 436.100 | 0.973 | 0.987 |
| NZ_CP040684.1_5922289..6005409_00011 | 143 | G_tfc24 | typeG | accessory | 4.500e-51 | 165.000 | 0.914 | 0.895 |
| NZ_CP040684.1_5922289..6005409_00015 | 980 | virb4 | typeG | mandatory | 1.500e-90 | 297.300 | 0.807 | 0.899 |
| NZ_CP040684.1_5922289..6005409_00016 | 147 | G_tfc15 | typeG | accessory | 3.200e-60 | 194.700 | 0.957 | 0.925 |
| NZ_CP040684.1_5922289..6005409_00017 | 501 | G_tfc14 | typeG | accessory | 7.100e-184 | 604.800 | 0.996 | 0.992 |
| NZ_CP040684.1_5922289..6005409_00018 | 294 | G_tfc13 | typeG | accessory | 3.000e-118 | 386.700 | 0.944 | 0.959 |
| NZ_CP040684.1_5922289..6005409_00019 | 219 | G_tfc12 | typeG | accessory | 6.700e-91 | 295.900 | 0.941 | 0.959 |
| NZ_CP040684.1_5922289..6005409_00020 | 128 | G_tfc11 | typeG | accessory | 5.900e-50 | 160.800 | 0.923 | 0.938 |
| NZ_CP040684.1_5922289..6005409_00021 | 118 | G_tfc10 | typeG | accessory | 3.500e-44 | 141.700 | 0.925 | 0.924 |
| NZ_CP040684.1_5922289..6005409_00022 | 79 | G_tfc9 | typeG | accessory | 1.100e-30 | 98.500 | 1.000 | 1.000 |
| NZ_CP040684.1_5922289..6005409_00023 | 112 | G_tfc8 | typeG | accessory | 1.500e-41 | 133.500 | 0.931 | 0.938 |
| NZ_CP040684.1_5922289..6005409_00028 | 248 | G_tfc7 | typeG | accessory | 7.700e-104 | 339.200 | 1.000 | 1.000 |
| NZ_CP040684.1_5922289..6005409_00029 | 743 | t4cp2 | typeG | mandatory | 3.600e-31 | 100.700 | 0.955 | 0.318 |
| NZ_CP040684.1_5922289..6005409_00031 | 166 | G_tfc5 | typeG | accessory | 3.100e-56 | 182.300 | 0.971 | 0.994 |
| NZ_CP040684.1_5922289..6005409_00033 | 251 | G_tfc3 | typeG | accessory | 3.000e-96 | 313.600 | 0.988 | 0.996 |
| NZ_CP040684.1_5922289..6005409_00060 | 374 | G_tfc2 | typeG | accessory | 1.300e-71 | 232.800 | 0.782 | 0.570 |
| NZ_CP041013.1_1254133..1357505_00002 | 640 | MOBH | typeG | mandatory | 3.300e-44 | 143.900 | 0.868 | 0.267 |
| NZ_CP041013.1_1254133..1357505_00027 | 510 | G_tfc19 | typeG | accessory | 1.600e-221 | 729.200 | 0.992 | 0.986 |
| NZ_CP041013.1_1254133..1357505_00028 | 115 | G_tfc18 | typeG | accessory | 3.200e-29 | 94.100 | 0.912 | 0.887 |
| NZ_CP041013.1_1254133..1357505_00029 | 460 | G_tfc22 | typeG | accessory | 7.500e-180 | 591.300 | 0.987 | 0.974 |
| NZ_CP041013.1_1254133..1357505_00030 | 312 | G_tfc23 | typeG | accessory | 3.700e-134 | 440.000 | 0.973 | 0.987 |
| NZ_CP041013.1_1254133..1357505_00031 | 143 | G_tfc24 | typeG | accessory | 9.500e-51 | 164.400 | 0.907 | 0.888 |
| NZ_CP041013.1_1254133..1357505_00036 | 980 | virb4 | typeG | mandatory | 4.700e-90 | 296.100 | 0.806 | 0.898 |
| NZ_CP041013.1_1254133..1357505_00037 | 147 | G_tfc15 | typeG | accessory | 4.500e-60 | 194.700 | 0.957 | 0.925 |
| NZ_CP041013.1_1254133..1357505_00038 | 501 | G_tfc14 | typeG | accessory | 1.200e-184 | 607.800 | 0.996 | 0.992 |
| NZ_CP041013.1_1254133..1357505_00039 | 294 | G_tfc13 | typeG | accessory | 1.900e-118 | 387.800 | 0.955 | 0.956 |
| NZ_CP041013.1_1254133..1357505_00040 | 219 | G_tfc12 | typeG | accessory | 8.800e-92 | 299.200 | 0.950 | 0.968 |
| NZ_CP041013.1_1254133..1357505_00041 | 128 | G_tfc11 | typeG | accessory | 5.600e-50 | 161.300 | 0.923 | 0.938 |
| NZ_CP041013.1_1254133..1357505_00042 | 118 | G_tfc10 | typeG | accessory | 7.900e-44 | 141.000 | 0.875 | 0.890 |
| NZ_CP041013.1_1254133..1357505_00043 | 79 | G_tfc9 | typeG | accessory | 1.300e-30 | 98.700 | 1.000 | 1.000 |
| NZ_CP041013.1_1254133..1357505_00044 | 112 | G_tfc8 | typeG | accessory | 1.600e-40 | 130.600 | 0.914 | 0.920 |
| NZ_CP041013.1_1254133..1357505_00050 | 249 | G_tfc7 | typeG | accessory | 1.100e-103 | 339.100 | 1.000 | 0.996 |
| NZ_CP041013.1_1254133..1357505_00051 | 743 | t4cp2 | typeG | mandatory | 4.900e-31 | 100.700 | 0.955 | 0.318 |
| NZ_CP041013.1_1254133..1357505_00053 | 166 | G_tfc5 | typeG | accessory | 2.900e-57 | 186.100 | 0.971 | 0.994 |
| NZ_CP041013.1_1254133..1357505_00055 | 251 | G_tfc3 | typeG | accessory | 4.100e-96 | 313.600 | 0.988 | 0.996 |
| NZ_CP041354.1_5208858..5312808_00084 | 250 | G_tfc3 | typeG | accessory | 6.600e-96 | 312.900 | 0.971 | 0.984 |
| NZ_CP041354.1_5208858..5312808_00086 | 166 | G_tfc5 | typeG | accessory | 2.700e-57 | 186.100 | 0.971 | 0.994 |
| NZ_CP041354.1_5208858..5312808_00088 | 743 | t4cp2 | typeG | mandatory | 4.600e-31 | 100.700 | 0.955 | 0.318 |
| NZ_CP041354.1_5208858..5312808_00089 | 248 | G_tfc7 | typeG | accessory | 4.600e-103 | 337.000 | 1.000 | 1.000 |
| NZ_CP041354.1_5208858..5312808_00094 | 112 | G_tfc8 | typeG | accessory | 1.900e-41 | 133.500 | 0.931 | 0.938 |
| NZ_CP041354.1_5208858..5312808_00095 | 79 | G_tfc9 | typeG | accessory | 1.400e-30 | 98.500 | 1.000 | 1.000 |
| NZ_CP041354.1_5208858..5312808_00096 | 118 | G_tfc10 | typeG | accessory | 7.400e-44 | 141.000 | 0.875 | 0.890 |
| NZ_CP041354.1_5208858..5312808_00097 | 128 | G_tfc11 | typeG | accessory | 5.300e-50 | 161.300 | 0.923 | 0.938 |
| NZ_CP041354.1_5208858..5312808_00098 | 219 | G_tfc12 | typeG | accessory | 8.300e-92 | 299.200 | 0.950 | 0.968 |
| NZ_CP041354.1_5208858..5312808_00099 | 294 | G_tfc13 | typeG | accessory | 4.000e-118 | 386.700 | 0.955 | 0.956 |
| NZ_CP041354.1_5208858..5312808_00100 | 501 | G_tfc14 | typeG | accessory | 1.000e-183 | 604.600 | 0.996 | 0.992 |
| NZ_CP041354.1_5208858..5312808_00101 | 147 | G_tfc15 | typeG | accessory | 4.200e-60 | 194.700 | 0.957 | 0.925 |
| NZ_CP041354.1_5208858..5312808_00102 | 980 | virb4 | typeG | mandatory | 1.300e-90 | 297.900 | 0.806 | 0.898 |
| NZ_CP041354.1_5208858..5312808_00107 | 143 | G_tfc24 | typeG | accessory | 8.900e-51 | 164.400 | 0.907 | 0.888 |
| NZ_CP041354.1_5208858..5312808_00108 | 312 | G_tfc23 | typeG | accessory | 8.900e-134 | 438.600 | 0.973 | 0.987 |
| NZ_CP041354.1_5208858..5312808_00109 | 462 | G_tfc22 | typeG | accessory | 5.200e-180 | 591.800 | 0.987 | 0.970 |
| NZ_CP041354.1_5208858..5312808_00110 | 115 | G_tfc18 | typeG | accessory | 3.800e-29 | 93.800 | 0.912 | 0.887 |
| NZ_CP041354.1_5208858..5312808_00111 | 511 | G_tfc19 | typeG | accessory | 6.700e-223 | 733.600 | 0.990 | 0.982 |
| NZ_CP041354.1_5208858..5312808_00119 | 639 | MOBH | typeG | mandatory | 4.500e-44 | 143.400 | 0.868 | 0.268 |
| NZ_CP041771.1_4614086..4707996_00065 | 252 | G_tfc3 | typeG | accessory | 8.700e-96 | 312.300 | 0.988 | 0.996 |
| NZ_CP041771.1_4614086..4707996_00067 | 166 | G_tfc5 | typeG | accessory | 1.000e-57 | 187.300 | 0.977 | 1.000 |
| NZ_CP041771.1_4614086..4707996_00069 | 743 | t4cp2 | typeG | mandatory | 4.000e-31 | 100.700 | 0.955 | 0.318 |
| NZ_CP041771.1_4614086..4707996_00070 | 248 | G_tfc7 | typeG | accessory | 4.000e-103 | 337.000 | 1.000 | 1.000 |
| NZ_CP041771.1_4614086..4707996_00075 | 112 | G_tfc8 | typeG | accessory | 1.600e-41 | 133.500 | 0.931 | 0.938 |
| NZ_CP041771.1_4614086..4707996_00076 | 79 | G_tfc9 | typeG | accessory | 1.200e-30 | 98.500 | 1.000 | 1.000 |
| NZ_CP041771.1_4614086..4707996_00077 | 118 | G_tfc10 | typeG | accessory | 6.400e-44 | 141.000 | 0.875 | 0.890 |
| NZ_CP041771.1_4614086..4707996_00078 | 128 | G_tfc11 | typeG | accessory | 4.600e-50 | 161.300 | 0.923 | 0.938 |
| NZ_CP041771.1_4614086..4707996_00079 | 219 | G_tfc12 | typeG | accessory | 7.200e-92 | 299.200 | 0.950 | 0.968 |
| NZ_CP041771.1_4614086..4707996_00080 | 294 | G_tfc13 | typeG | accessory | 3.500e-118 | 386.700 | 0.955 | 0.956 |
| NZ_CP041771.1_4614086..4707996_00081 | 501 | G_tfc14 | typeG | accessory | 9.000e-184 | 604.600 | 0.996 | 0.992 |
| NZ_CP041771.1_4614086..4707996_00082 | 147 | G_tfc15 | typeG | accessory | 3.600e-60 | 194.700 | 0.957 | 0.925 |
| NZ_CP041771.1_4614086..4707996_00083 | 980 | virb4 | typeG | mandatory | 1.100e-90 | 297.900 | 0.806 | 0.898 |
| NZ_CP041771.1_4614086..4707996_00089 | 143 | G_tfc24 | typeG | accessory | 7.700e-51 | 164.400 | 0.907 | 0.888 |
| NZ_CP041771.1_4614086..4707996_00090 | 312 | G_tfc23 | typeG | accessory | 7.700e-134 | 438.600 | 0.973 | 0.987 |
| NZ_CP041771.1_4614086..4707996_00091 | 462 | G_tfc22 | typeG | accessory | 4.500e-180 | 591.800 | 0.987 | 0.970 |
| NZ_CP041771.1_4614086..4707996_00092 | 115 | G_tfc18 | typeG | accessory | 3.300e-29 | 93.800 | 0.912 | 0.887 |
| NZ_CP041771.1_4614086..4707996_00093 | 511 | G_tfc19 | typeG | accessory | 5.800e-223 | 733.600 | 0.990 | 0.982 |
| NZ_CP041771.1_4614086..4707996_00103 | 639 | MOBH | typeG | mandatory | 3.900e-44 | 143.400 | 0.868 | 0.268 |
| NZ_CP041774.1_5519535..5613671_00071 | 251 | G_tfc3 | typeG | accessory | 3.600e-96 | 313.600 | 0.988 | 0.996 |
| NZ_CP041774.1_5519535..5613671_00073 | 166 | G_tfc5 | typeG | accessory | 2.600e-57 | 186.100 | 0.971 | 0.994 |
| NZ_CP041774.1_5519535..5613671_00075 | 743 | t4cp2 | typeG | mandatory | 4.300e-31 | 100.700 | 0.955 | 0.318 |
| NZ_CP041774.1_5519535..5613671_00076 | 249 | G_tfc7 | typeG | accessory | 1.000e-103 | 339.100 | 1.000 | 0.996 |
| NZ_CP041774.1_5519535..5613671_00082 | 112 | G_tfc8 | typeG | accessory | 1.400e-40 | 130.600 | 0.914 | 0.920 |
| NZ_CP041774.1_5519535..5613671_00083 | 79 | G_tfc9 | typeG | accessory | 1.100e-30 | 98.700 | 1.000 | 1.000 |
| NZ_CP041774.1_5519535..5613671_00084 | 118 | G_tfc10 | typeG | accessory | 7.000e-44 | 141.000 | 0.875 | 0.890 |
| NZ_CP041774.1_5519535..5613671_00085 | 128 | G_tfc11 | typeG | accessory | 5.000e-50 | 161.300 | 0.923 | 0.938 |
| NZ_CP041774.1_5519535..5613671_00086 | 219 | G_tfc12 | typeG | accessory | 7.800e-92 | 299.200 | 0.950 | 0.968 |
| NZ_CP041774.1_5519535..5613671_00087 | 294 | G_tfc13 | typeG | accessory | 1.700e-118 | 387.800 | 0.955 | 0.956 |
| NZ_CP041774.1_5519535..5613671_00088 | 501 | G_tfc14 | typeG | accessory | 1.100e-184 | 607.800 | 0.996 | 0.992 |
| NZ_CP041774.1_5519535..5613671_00089 | 147 | G_tfc15 | typeG | accessory | 4.000e-60 | 194.700 | 0.957 | 0.925 |
| NZ_CP041774.1_5519535..5613671_00090 | 980 | virb4 | typeG | mandatory | 1.200e-90 | 297.900 | 0.806 | 0.898 |
| NZ_CP041774.1_5519535..5613671_00095 | 143 | G_tfc24 | typeG | accessory | 8.400e-51 | 164.400 | 0.907 | 0.888 |
| NZ_CP041774.1_5519535..5613671_00096 | 312 | G_tfc23 | typeG | accessory | 8.400e-134 | 438.600 | 0.973 | 0.987 |
| NZ_CP041774.1_5519535..5613671_00097 | 460 | G_tfc22 | typeG | accessory | 8.700e-180 | 591.000 | 0.987 | 0.974 |
| NZ_CP041774.1_5519535..5613671_00098 | 118 | G_tfc18 | typeG | accessory | 2.300e-27 | 87.900 | 0.912 | 0.890 |
| NZ_CP041774.1_5519535..5613671_00099 | 504 | G_tfc19 | typeG | accessory | 8.600e-222 | 729.900 | 0.984 | 0.990 |
| NZ_CP041774.1_5519535..5613671_00113 | 639 | MOBH | typeG | mandatory | 3.000e-44 | 143.900 | 0.868 | 0.268 |
| NZ_CP043328.1_5638612..5757809_00093 | 251 | G_tfc3 | typeG | accessory | 3.200e-95 | 310.800 | 0.967 | 0.976 |
| NZ_CP043328.1_5638612..5757809_00095 | 166 | G_tfc5 | typeG | accessory | 2.900e-57 | 186.200 | 0.977 | 1.000 |
| NZ_CP043328.1_5638612..5757809_00097 | 743 | t4cp2 | typeG | mandatory | 5.300e-31 | 100.700 | 0.955 | 0.318 |
| NZ_CP043328.1_5638612..5757809_00098 | 249 | G_tfc7 | typeG | accessory | 1.200e-103 | 339.100 | 1.000 | 0.996 |
| NZ_CP043328.1_5638612..5757809_00104 | 112 | G_tfc8 | typeG | accessory | 1.700e-40 | 130.600 | 0.914 | 0.920 |
| NZ_CP043328.1_5638612..5757809_00105 | 79 | G_tfc9 | typeG | accessory | 1.400e-30 | 98.700 | 1.000 | 1.000 |
| NZ_CP043328.1_5638612..5757809_00106 | 118 | G_tfc10 | typeG | accessory | 8.400e-44 | 141.000 | 0.875 | 0.890 |
| NZ_CP043328.1_5638612..5757809_00107 | 128 | G_tfc11 | typeG | accessory | 6.000e-50 | 161.300 | 0.923 | 0.938 |
| NZ_CP043328.1_5638612..5757809_00108 | 219 | G_tfc12 | typeG | accessory | 9.400e-92 | 299.200 | 0.950 | 0.968 |
| NZ_CP043328.1_5638612..5757809_00109 | 294 | G_tfc13 | typeG | accessory | 4.600e-118 | 386.700 | 0.955 | 0.956 |
| NZ_CP043328.1_5638612..5757809_00110 | 501 | G_tfc14 | typeG | accessory | 1.200e-183 | 604.500 | 0.996 | 0.992 |
| NZ_CP043328.1_5638612..5757809_00111 | 147 | G_tfc15 | typeG | accessory | 4.800e-60 | 194.700 | 0.957 | 0.925 |
| NZ_CP043328.1_5638612..5757809_00112 | 980 | virb4 | typeG | mandatory | 1.500e-90 | 297.800 | 0.806 | 0.898 |
| NZ_CP043328.1_5638612..5757809_00116 | 143 | G_tfc24 | typeG | accessory | 1.100e-50 | 164.300 | 0.907 | 0.888 |
| NZ_CP043328.1_5638612..5757809_00117 | 312 | G_tfc23 | typeG | accessory | 2.200e-133 | 437.500 | 0.973 | 0.987 |
| NZ_CP043328.1_5638612..5757809_00118 | 460 | G_tfc22 | typeG | accessory | 3.400e-180 | 592.600 | 0.987 | 0.974 |
| NZ_CP043328.1_5638612..5757809_00119 | 105 | G_tfc18 | typeG | accessory | 1.100e-19 | 63.500 | 0.895 | 0.867 |
| NZ_CP043328.1_5638612..5757809_00120 | 508 | G_tfc19 | typeG | accessory | 1.100e-220 | 726.600 | 0.992 | 0.990 |
| NZ_CP043328.1_5638612..5757809_00136 | 639 | MOBH | typeG | mandatory | 2.300e-44 | 144.500 | 0.868 | 0.268 |
| NZ_CP044006.1_4742387..4839256_00079 | 251 | G_tfc3 | typeG | accessory | 4.000e-96 | 313.600 | 0.988 | 0.996 |
| NZ_CP044006.1_4742387..4839256_00081 | 166 | G_tfc5 | typeG | accessory | 2.900e-57 | 186.100 | 0.971 | 0.994 |
| NZ_CP044006.1_4742387..4839256_00083 | 743 | t4cp2 | typeG | mandatory | 4.800e-31 | 100.700 | 0.955 | 0.318 |
| NZ_CP044006.1_4742387..4839256_00084 | 249 | G_tfc7 | typeG | accessory | 1.100e-103 | 339.100 | 1.000 | 0.996 |
| NZ_CP044006.1_4742387..4839256_00099 | 112 | G_tfc8 | typeG | accessory | 6.100e-41 | 131.900 | 0.914 | 0.920 |
| NZ_CP044006.1_4742387..4839256_00100 | 79 | G_tfc9 | typeG | accessory | 9.600e-31 | 99.000 | 1.000 | 1.000 |
| NZ_CP044006.1_4742387..4839256_00101 | 118 | G_tfc10 | typeG | accessory | 2.700e-42 | 136.100 | 0.933 | 0.932 |
| NZ_CP044006.1_4742387..4839256_00102 | 128 | G_tfc11 | typeG | accessory | 5.000e-50 | 161.400 | 0.923 | 0.938 |
| NZ_CP044006.1_4742387..4839256_00103 | 219 | G_tfc12 | typeG | accessory | 1.300e-91 | 298.600 | 0.941 | 0.959 |
| NZ_CP044006.1_4742387..4839256_00104 | 294 | G_tfc13 | typeG | accessory | 4.100e-119 | 390.000 | 0.944 | 0.959 |
| NZ_CP044006.1_4742387..4839256_00105 | 501 | G_tfc14 | typeG | accessory | 1.200e-184 | 607.800 | 0.996 | 0.992 |
| NZ_CP044006.1_4742387..4839256_00106 | 147 | G_tfc15 | typeG | accessory | 4.400e-60 | 194.700 | 0.957 | 0.925 |
| NZ_CP044006.1_4742387..4839256_00107 | 980 | virb4 | typeG | mandatory | 7.700e-90 | 295.400 | 0.806 | 0.898 |
| NZ_CP044006.1_4742387..4839256_00113 | 143 | G_tfc24 | typeG | accessory | 9.300e-51 | 164.400 | 0.907 | 0.888 |
| NZ_CP044006.1_4742387..4839256_00114 | 312 | G_tfc23 | typeG | accessory | 3.600e-134 | 440.000 | 0.973 | 0.987 |
| NZ_CP044006.1_4742387..4839256_00115 | 460 | G_tfc22 | typeG | accessory | 5.400e-180 | 591.800 | 0.987 | 0.974 |
| NZ_CP044006.1_4742387..4839256_00116 | 115 | G_tfc18 | typeG | accessory | 4.200e-29 | 93.700 | 0.912 | 0.887 |
| NZ_CP044006.1_4742387..4839256_00117 | 504 | G_tfc19 | typeG | accessory | 1.600e-222 | 732.500 | 0.984 | 0.990 |
| NZ_CP044006.1_4742387..4839256_00125 | 639 | MOBH | typeG | mandatory | 4.600e-44 | 143.400 | 0.868 | 0.268 |
| NZ_CP045739.1_5788614..5873476_00035 | 374 | G_tfc2 | typeG | accessory | 1.300e-71 | 232.900 | 0.782 | 0.631 |
| NZ_CP045739.1_5788614..5873476_00066 | 251 | G_tfc3 | typeG | accessory | 6.900e-96 | 312.600 | 0.967 | 0.976 |
| NZ_CP045739.1_5788614..5873476_00068 | 166 | G_tfc5 | typeG | accessory | 2.500e-57 | 186.000 | 0.971 | 0.994 |
| NZ_CP045739.1_5788614..5873476_00070 | 743 | t4cp2 | typeG | mandatory | 3.900e-31 | 100.700 | 0.955 | 0.318 |
| NZ_CP045739.1_5788614..5873476_00071 | 248 | G_tfc7 | typeG | accessory | 9.500e-104 | 339.000 | 1.000 | 1.000 |
| NZ_CP045739.1_5788614..5873476_00077 | 112 | G_tfc8 | typeG | accessory | 5.600e-41 | 131.800 | 0.931 | 0.938 |
| NZ_CP045739.1_5788614..5873476_00078 | 79 | G_tfc9 | typeG | accessory | 1.900e-30 | 97.800 | 1.000 | 1.000 |
| NZ_CP045739.1_5788614..5873476_00079 | 118 | G_tfc10 | typeG | accessory | 5.300e-44 | 141.300 | 0.925 | 0.924 |
| NZ_CP045739.1_5788614..5873476_00080 | 128 | G_tfc11 | typeG | accessory | 9.800e-50 | 160.200 | 0.923 | 0.938 |
| NZ_CP045739.1_5788614..5873476_00081 | 219 | G_tfc12 | typeG | accessory | 1.300e-91 | 298.400 | 0.950 | 0.968 |
| NZ_CP045739.1_5788614..5873476_00082 | 294 | G_tfc13 | typeG | accessory | 9.900e-119 | 388.400 | 0.955 | 0.956 |
| NZ_CP045739.1_5788614..5873476_00083 | 501 | G_tfc14 | typeG | accessory | 2.200e-184 | 606.600 | 0.996 | 0.992 |
| NZ_CP045739.1_5788614..5873476_00084 | 147 | G_tfc15 | typeG | accessory | 1.600e-59 | 192.600 | 0.957 | 0.925 |
| NZ_CP045739.1_5788614..5873476_00085 | 983 | virb4 | typeG | mandatory | 1.200e-89 | 294.500 | 0.810 | 0.899 |
| NZ_CP045739.1_5788614..5873476_00091 | 143 | G_tfc24 | typeG | accessory | 3.600e-51 | 165.400 | 0.907 | 0.888 |
| NZ_CP045739.1_5788614..5873476_00092 | 312 | G_tfc23 | typeG | accessory | 2.700e-134 | 440.100 | 0.973 | 0.987 |
| NZ_CP045739.1_5788614..5873476_00093 | 460 | G_tfc22 | typeG | accessory | 5.800e-180 | 591.400 | 0.989 | 0.976 |
| NZ_CP045739.1_5788614..5873476_00094 | 115 | G_tfc18 | typeG | accessory | 3.600e-29 | 93.600 | 0.912 | 0.887 |
| NZ_CP045739.1_5788614..5873476_00095 | 504 | G_tfc19 | typeG | accessory | 2.200e-223 | 735.000 | 0.988 | 0.994 |
| NZ_CP045739.1_5788614..5873476_00102 | 640 | MOBH | typeG | mandatory | 2.700e-44 | 143.900 | 0.868 | 0.267 |
| NZ_CP046060.1_5988162..6087613_00082 | 251 | G_tfc3 | typeG | accessory | 4.200e-96 | 313.600 | 0.988 | 0.996 |
| NZ_CP046060.1_5988162..6087613_00084 | 166 | G_tfc5 | typeG | accessory | 3.000e-57 | 186.100 | 0.971 | 0.994 |
| NZ_CP046060.1_5988162..6087613_00086 | 743 | t4cp2 | typeG | mandatory | 5.000e-31 | 100.700 | 0.955 | 0.318 |
| NZ_CP046060.1_5988162..6087613_00087 | 249 | G_tfc7 | typeG | accessory | 1.200e-103 | 339.100 | 1.000 | 0.996 |
| NZ_CP046060.1_5988162..6087613_00104 | 112 | G_tfc8 | typeG | accessory | 1.600e-40 | 130.600 | 0.914 | 0.920 |
| NZ_CP046060.1_5988162..6087613_00105 | 79 | G_tfc9 | typeG | accessory | 1.300e-30 | 98.700 | 1.000 | 1.000 |
| NZ_CP046060.1_5988162..6087613_00106 | 118 | G_tfc10 | typeG | accessory | 8.100e-44 | 141.000 | 0.875 | 0.890 |
| NZ_CP046060.1_5988162..6087613_00107 | 128 | G_tfc11 | typeG | accessory | 5.800e-50 | 161.300 | 0.923 | 0.938 |
| NZ_CP046060.1_5988162..6087613_00108 | 219 | G_tfc12 | typeG | accessory | 9.000e-92 | 299.200 | 0.950 | 0.968 |
| NZ_CP046060.1_5988162..6087613_00109 | 294 | G_tfc13 | typeG | accessory | 2.000e-118 | 387.800 | 0.955 | 0.956 |
| NZ_CP046060.1_5988162..6087613_00110 | 501 | G_tfc14 | typeG | accessory | 1.200e-184 | 607.800 | 0.996 | 0.992 |
| NZ_CP046060.1_5988162..6087613_00111 | 147 | G_tfc15 | typeG | accessory | 4.600e-60 | 194.700 | 0.957 | 0.925 |
| NZ_CP046060.1_5988162..6087613_00112 | 980 | virb4 | typeG | mandatory | 4.600e-90 | 296.100 | 0.806 | 0.898 |
| NZ_CP046060.1_5988162..6087613_00118 | 143 | G_tfc24 | typeG | accessory | 9.700e-51 | 164.400 | 0.907 | 0.888 |
| NZ_CP046060.1_5988162..6087613_00119 | 312 | G_tfc23 | typeG | accessory | 3.800e-134 | 440.000 | 0.973 | 0.987 |
| NZ_CP046060.1_5988162..6087613_00120 | 460 | G_tfc22 | typeG | accessory | 5.600e-180 | 591.800 | 0.987 | 0.974 |
| NZ_CP046060.1_5988162..6087613_00121 | 115 | G_tfc18 | typeG | accessory | 4.400e-29 | 93.700 | 0.912 | 0.887 |
| NZ_CP046060.1_5988162..6087613_00122 | 504 | G_tfc19 | typeG | accessory | 1.600e-222 | 732.500 | 0.984 | 0.990 |
| NZ_CP046060.1_5988162..6087613_00130 | 639 | MOBH | typeG | mandatory | 4.800e-44 | 143.400 | 0.868 | 0.268 |
| NZ_CP046061.1_5987958..6087409_00082 | 251 | G_tfc3 | typeG | accessory | 4.200e-96 | 313.600 | 0.988 | 0.996 |
| NZ_CP046061.1_5987958..6087409_00084 | 166 | G_tfc5 | typeG | accessory | 3.000e-57 | 186.100 | 0.971 | 0.994 |
| NZ_CP046061.1_5987958..6087409_00086 | 743 | t4cp2 | typeG | mandatory | 5.000e-31 | 100.700 | 0.955 | 0.318 |
| NZ_CP046061.1_5987958..6087409_00087 | 249 | G_tfc7 | typeG | accessory | 1.200e-103 | 339.100 | 1.000 | 0.996 |
| NZ_CP046061.1_5987958..6087409_00104 | 112 | G_tfc8 | typeG | accessory | 1.600e-40 | 130.600 | 0.914 | 0.920 |
| NZ_CP046061.1_5987958..6087409_00105 | 79 | G_tfc9 | typeG | accessory | 1.300e-30 | 98.700 | 1.000 | 1.000 |
| NZ_CP046061.1_5987958..6087409_00106 | 118 | G_tfc10 | typeG | accessory | 8.100e-44 | 141.000 | 0.875 | 0.890 |
| NZ_CP046061.1_5987958..6087409_00107 | 128 | G_tfc11 | typeG | accessory | 5.800e-50 | 161.300 | 0.923 | 0.938 |
| NZ_CP046061.1_5987958..6087409_00108 | 219 | G_tfc12 | typeG | accessory | 9.000e-92 | 299.200 | 0.950 | 0.968 |
| NZ_CP046061.1_5987958..6087409_00109 | 294 | G_tfc13 | typeG | accessory | 2.000e-118 | 387.800 | 0.955 | 0.956 |
| NZ_CP046061.1_5987958..6087409_00110 | 501 | G_tfc14 | typeG | accessory | 1.200e-184 | 607.800 | 0.996 | 0.992 |
| NZ_CP046061.1_5987958..6087409_00111 | 147 | G_tfc15 | typeG | accessory | 4.600e-60 | 194.700 | 0.957 | 0.925 |
| NZ_CP046061.1_5987958..6087409_00112 | 980 | virb4 | typeG | mandatory | 4.600e-90 | 296.100 | 0.806 | 0.898 |
| NZ_CP046061.1_5987958..6087409_00118 | 143 | G_tfc24 | typeG | accessory | 9.700e-51 | 164.400 | 0.907 | 0.888 |
| NZ_CP046061.1_5987958..6087409_00119 | 312 | G_tfc23 | typeG | accessory | 3.800e-134 | 440.000 | 0.973 | 0.987 |
| NZ_CP046061.1_5987958..6087409_00120 | 460 | G_tfc22 | typeG | accessory | 5.600e-180 | 591.800 | 0.987 | 0.974 |
| NZ_CP046061.1_5987958..6087409_00121 | 115 | G_tfc18 | typeG | accessory | 4.400e-29 | 93.700 | 0.912 | 0.887 |
| NZ_CP046061.1_5987958..6087409_00122 | 504 | G_tfc19 | typeG | accessory | 1.600e-222 | 732.500 | 0.984 | 0.990 |
| NZ_CP046061.1_5987958..6087409_00130 | 639 | MOBH | typeG | mandatory | 4.800e-44 | 143.400 | 0.868 | 0.268 |
| NZ_CP046069.1_5366781..5465070_00083 | 251 | G_tfc3 | typeG | accessory | 8.600e-96 | 312.500 | 0.967 | 0.976 |
| NZ_CP046069.1_5366781..5465070_00085 | 166 | G_tfc5 | typeG | accessory | 3.600e-57 | 185.700 | 0.971 | 0.994 |
| NZ_CP046069.1_5366781..5465070_00087 | 743 | t4cp2 | typeG | mandatory | 4.600e-31 | 100.700 | 0.955 | 0.318 |
| NZ_CP046069.1_5366781..5465070_00088 | 248 | G_tfc7 | typeG | accessory | 4.700e-103 | 337.000 | 1.000 | 1.000 |
| NZ_CP046069.1_5366781..5465070_00093 | 112 | G_tfc8 | typeG | accessory | 1.900e-41 | 133.500 | 0.931 | 0.938 |
| NZ_CP046069.1_5366781..5465070_00094 | 79 | G_tfc9 | typeG | accessory | 1.400e-30 | 98.500 | 1.000 | 1.000 |
| NZ_CP046069.1_5366781..5465070_00095 | 118 | G_tfc10 | typeG | accessory | 7.500e-44 | 141.000 | 0.875 | 0.890 |
| NZ_CP046069.1_5366781..5465070_00096 | 128 | G_tfc11 | typeG | accessory | 5.400e-50 | 161.300 | 0.923 | 0.938 |
| NZ_CP046069.1_5366781..5465070_00097 | 219 | G_tfc12 | typeG | accessory | 8.400e-92 | 299.200 | 0.950 | 0.968 |
| NZ_CP046069.1_5366781..5465070_00098 | 294 | G_tfc13 | typeG | accessory | 4.100e-118 | 386.700 | 0.955 | 0.956 |
| NZ_CP046069.1_5366781..5465070_00099 | 501 | G_tfc14 | typeG | accessory | 1.100e-183 | 604.600 | 0.996 | 0.992 |
| NZ_CP046069.1_5366781..5465070_00100 | 147 | G_tfc15 | typeG | accessory | 4.300e-60 | 194.700 | 0.957 | 0.925 |
| NZ_CP046069.1_5366781..5465070_00101 | 980 | virb4 | typeG | mandatory | 1.300e-90 | 297.900 | 0.806 | 0.898 |
| NZ_CP046069.1_5366781..5465070_00107 | 143 | G_tfc24 | typeG | accessory | 9.000e-51 | 164.400 | 0.907 | 0.888 |
| NZ_CP046069.1_5366781..5465070_00108 | 312 | G_tfc23 | typeG | accessory | 9.000e-134 | 438.600 | 0.973 | 0.987 |
| NZ_CP046069.1_5366781..5465070_00109 | 462 | G_tfc22 | typeG | accessory | 5.300e-180 | 591.800 | 0.987 | 0.970 |
| NZ_CP046069.1_5366781..5465070_00110 | 115 | G_tfc18 | typeG | accessory | 3.800e-29 | 93.800 | 0.912 | 0.887 |
| NZ_CP046069.1_5366781..5465070_00111 | 511 | G_tfc19 | typeG | accessory | 6.900e-223 | 733.600 | 0.990 | 0.982 |
| NZ_CP046069.1_5366781..5465070_00121 | 639 | MOBH | typeG | mandatory | 4.600e-44 | 143.400 | 0.868 | 0.268 |
| NZ_CP047592.1_5607949..5701975_00072 | 251 | G_tfc3 | typeG | accessory | 4.500e-95 | 310.000 | 0.967 | 0.976 |
| NZ_CP047592.1_5607949..5701975_00074 | 166 | G_tfc5 | typeG | accessory | 2.500e-56 | 182.800 | 0.971 | 0.994 |
| NZ_CP047592.1_5607949..5701975_00076 | 743 | t4cp2 | typeG | mandatory | 4.200e-31 | 100.700 | 0.955 | 0.318 |
| NZ_CP047592.1_5607949..5701975_00077 | 248 | G_tfc7 | typeG | accessory | 7.700e-104 | 339.400 | 1.000 | 1.000 |
| NZ_CP047592.1_5607949..5701975_00082 | 112 | G_tfc8 | typeG | accessory | 6.300e-41 | 131.700 | 0.922 | 0.929 |
| NZ_CP047592.1_5607949..5701975_00083 | 79 | G_tfc9 | typeG | accessory | 8.400e-31 | 99.000 | 1.000 | 1.000 |
| NZ_CP047592.1_5607949..5701975_00084 | 118 | G_tfc10 | typeG | accessory | 2.900e-44 | 142.200 | 0.875 | 0.890 |
| NZ_CP047592.1_5607949..5701975_00085 | 128 | G_tfc11 | typeG | accessory | 8.300e-50 | 160.500 | 0.923 | 0.938 |
| NZ_CP047592.1_5607949..5701975_00086 | 219 | G_tfc12 | typeG | accessory | 6.200e-91 | 296.200 | 0.941 | 0.959 |
| NZ_CP047592.1_5607949..5701975_00087 | 294 | G_tfc13 | typeG | accessory | 6.700e-117 | 382.500 | 0.941 | 0.952 |
| NZ_CP047592.1_5607949..5701975_00088 | 501 | G_tfc14 | typeG | accessory | 7.400e-185 | 608.300 | 0.996 | 0.992 |
| NZ_CP047592.1_5607949..5701975_00089 | 147 | G_tfc15 | typeG | accessory | 3.400e-60 | 194.900 | 0.957 | 0.925 |
| NZ_CP047592.1_5607949..5701975_00090 | 980 | virb4 | typeG | mandatory | 7.600e-91 | 298.500 | 0.808 | 0.900 |
| NZ_CP047592.1_5607949..5701975_00096 | 143 | G_tfc24 | typeG | accessory | 4.600e-50 | 161.900 | 0.914 | 0.895 |
| NZ_CP047592.1_5607949..5701975_00097 | 312 | G_tfc23 | typeG | accessory | 6.000e-134 | 439.100 | 0.973 | 0.987 |
| NZ_CP047592.1_5607949..5701975_00098 | 460 | G_tfc22 | typeG | accessory | 1.200e-179 | 590.500 | 0.987 | 0.974 |
| NZ_CP047592.1_5607949..5701975_00099 | 115 | G_tfc18 | typeG | accessory | 3.200e-29 | 93.900 | 0.895 | 0.878 |
| NZ_CP047592.1_5607949..5701975_00100 | 504 | G_tfc19 | typeG | accessory | 6.900e-223 | 733.500 | 0.984 | 0.990 |
| NZ_CP047592.1_5607949..5701975_00109 | 639 | MOBH | typeG | mandatory | 2.400e-44 | 144.200 | 0.868 | 0.268 |
| NZ_LR130527.1_1796189..1903319_00028 | 374 | G_tfc2 | typeG | accessory | 1.600e-71 | 232.900 | 0.782 | 0.631 |
| NZ_LR130527.1_1796189..1903319_00059 | 251 | G_tfc3 | typeG | accessory | 9.200e-96 | 312.400 | 0.988 | 0.996 |
| NZ_LR130527.1_1796189..1903319_00061 | 166 | G_tfc5 | typeG | accessory | 6.300e-57 | 185.000 | 0.971 | 0.988 |
| NZ_LR130527.1_1796189..1903319_00063 | 743 | t4cp2 | typeG | mandatory | 4.700e-31 | 100.700 | 0.955 | 0.318 |
| NZ_LR130527.1_1796189..1903319_00064 | 248 | G_tfc7 | typeG | accessory | 4.400e-103 | 337.100 | 1.000 | 1.000 |
| NZ_LR130527.1_1796189..1903319_00067 | 112 | G_tfc8 | typeG | accessory | 1.600e-40 | 130.600 | 0.931 | 0.938 |
| NZ_LR130527.1_1796189..1903319_00068 | 79 | G_tfc9 | typeG | accessory | 9.400e-31 | 99.000 | 1.000 | 1.000 |
| NZ_LR130527.1_1796189..1903319_00069 | 118 | G_tfc10 | typeG | accessory | 6.500e-42 | 134.800 | 0.858 | 0.873 |
| NZ_LR130527.1_1796189..1903319_00070 | 128 | G_tfc11 | typeG | accessory | 4.900e-50 | 161.400 | 0.923 | 0.938 |
| NZ_LR130527.1_1796189..1903319_00071 | 219 | G_tfc12 | typeG | accessory | 6.700e-92 | 299.500 | 0.950 | 0.968 |
| NZ_LR130527.1_1796189..1903319_00072 | 294 | G_tfc13 | typeG | accessory | 2.400e-118 | 387.400 | 0.955 | 0.956 |
| NZ_LR130527.1_1796189..1903319_00073 | 501 | G_tfc14 | typeG | accessory | 1.200e-184 | 607.800 | 0.996 | 0.992 |
| NZ_LR130527.1_1796189..1903319_00074 | 147 | G_tfc15 | typeG | accessory | 4.000e-60 | 194.800 | 0.957 | 0.925 |
| NZ_LR130527.1_1796189..1903319_00075 | 980 | virb4 | typeG | mandatory | 1.900e-90 | 297.400 | 0.807 | 0.899 |
| NZ_LR130527.1_1796189..1903319_00082 | 143 | G_tfc24 | typeG | accessory | 3.000e-51 | 165.900 | 0.914 | 0.895 |
| NZ_LR130527.1_1796189..1903319_00083 | 312 | G_tfc23 | typeG | accessory | 1.600e-133 | 437.800 | 0.976 | 0.990 |
| NZ_LR130527.1_1796189..1903319_00084 | 460 | G_tfc22 | typeG | accessory | 4.100e-180 | 592.200 | 0.987 | 0.974 |
| NZ_LR130527.1_1796189..1903319_00085 | 105 | G_tfc18 | typeG | accessory | 5.900e-20 | 64.200 | 0.912 | 0.876 |
| NZ_LR130527.1_1796189..1903319_00086 | 511 | G_tfc19 | typeG | accessory | 1.700e-222 | 732.300 | 0.990 | 0.982 |
| NZ_LR130527.1_1796189..1903319_00123 | 639 | MOBH | typeG | mandatory | 4.900e-44 | 143.300 | 0.868 | 0.268 |
| NZ_LR130530.1_5115586..5222717_00028 | 374 | G_tfc2 | typeG | accessory | 1.600e-71 | 232.900 | 0.782 | 0.631 |
| NZ_LR130530.1_5115586..5222717_00059 | 251 | G_tfc3 | typeG | accessory | 9.100e-96 | 312.400 | 0.988 | 0.996 |
| NZ_LR130530.1_5115586..5222717_00061 | 166 | G_tfc5 | typeG | accessory | 6.300e-57 | 185.000 | 0.971 | 0.988 |
| NZ_LR130530.1_5115586..5222717_00063 | 743 | t4cp2 | typeG | mandatory | 4.700e-31 | 100.700 | 0.955 | 0.318 |
| NZ_LR130530.1_5115586..5222717_00064 | 248 | G_tfc7 | typeG | accessory | 4.300e-103 | 337.100 | 1.000 | 1.000 |
| NZ_LR130530.1_5115586..5222717_00067 | 112 | G_tfc8 | typeG | accessory | 1.500e-40 | 130.600 | 0.931 | 0.938 |
| NZ_LR130530.1_5115586..5222717_00068 | 79 | G_tfc9 | typeG | accessory | 9.400e-31 | 99.000 | 1.000 | 1.000 |
| NZ_LR130530.1_5115586..5222717_00069 | 118 | G_tfc10 | typeG | accessory | 6.500e-42 | 134.800 | 0.858 | 0.873 |
| NZ_LR130530.1_5115586..5222717_00070 | 128 | G_tfc11 | typeG | accessory | 4.900e-50 | 161.400 | 0.923 | 0.938 |
| NZ_LR130530.1_5115586..5222717_00071 | 219 | G_tfc12 | typeG | accessory | 6.600e-92 | 299.500 | 0.950 | 0.968 |
| NZ_LR130530.1_5115586..5222717_00072 | 294 | G_tfc13 | typeG | accessory | 2.400e-118 | 387.400 | 0.955 | 0.956 |
| NZ_LR130530.1_5115586..5222717_00073 | 501 | G_tfc14 | typeG | accessory | 1.200e-184 | 607.800 | 0.996 | 0.992 |
| NZ_LR130530.1_5115586..5222717_00074 | 147 | G_tfc15 | typeG | accessory | 4.000e-60 | 194.800 | 0.957 | 0.925 |
| NZ_LR130530.1_5115586..5222717_00075 | 980 | virb4 | typeG | mandatory | 1.800e-90 | 297.400 | 0.807 | 0.899 |
| NZ_LR130530.1_5115586..5222717_00082 | 143 | G_tfc24 | typeG | accessory | 3.000e-51 | 165.900 | 0.914 | 0.895 |
| NZ_LR130530.1_5115586..5222717_00083 | 312 | G_tfc23 | typeG | accessory | 1.600e-133 | 437.800 | 0.976 | 0.990 |
| NZ_LR130530.1_5115586..5222717_00084 | 460 | G_tfc22 | typeG | accessory | 4.100e-180 | 592.200 | 0.987 | 0.974 |
| NZ_LR130530.1_5115586..5222717_00085 | 105 | G_tfc18 | typeG | accessory | 5.800e-20 | 64.200 | 0.912 | 0.876 |
| NZ_LR130530.1_5115586..5222717_00086 | 511 | G_tfc19 | typeG | accessory | 1.700e-222 | 732.300 | 0.990 | 0.982 |
| NZ_LR130530.1_5115586..5222717_00122 | 639 | MOBH | typeG | mandatory | 4.900e-44 | 143.300 | 0.868 | 0.268 |
| NZ_LR130531.1_2433304..2540433_00028 | 374 | G_tfc2 | typeG | accessory | 1.600e-71 | 232.900 | 0.782 | 0.631 |
| NZ_LR130531.1_2433304..2540433_00058 | 251 | G_tfc3 | typeG | accessory | 9.100e-96 | 312.400 | 0.988 | 0.996 |
| NZ_LR130531.1_2433304..2540433_00060 | 166 | G_tfc5 | typeG | accessory | 6.200e-57 | 185.000 | 0.971 | 0.988 |
| NZ_LR130531.1_2433304..2540433_00062 | 743 | t4cp2 | typeG | mandatory | 4.600e-31 | 100.700 | 0.955 | 0.318 |
| NZ_LR130531.1_2433304..2540433_00063 | 248 | G_tfc7 | typeG | accessory | 4.300e-103 | 337.100 | 1.000 | 1.000 |
| NZ_LR130531.1_2433304..2540433_00066 | 112 | G_tfc8 | typeG | accessory | 1.500e-40 | 130.600 | 0.931 | 0.938 |
| NZ_LR130531.1_2433304..2540433_00067 | 79 | G_tfc9 | typeG | accessory | 9.300e-31 | 99.000 | 1.000 | 1.000 |
| NZ_LR130531.1_2433304..2540433_00068 | 118 | G_tfc10 | typeG | accessory | 6.400e-42 | 134.800 | 0.858 | 0.873 |
| NZ_LR130531.1_2433304..2540433_00069 | 128 | G_tfc11 | typeG | accessory | 4.800e-50 | 161.400 | 0.923 | 0.938 |
| NZ_LR130531.1_2433304..2540433_00070 | 219 | G_tfc12 | typeG | accessory | 6.600e-92 | 299.500 | 0.950 | 0.968 |
| NZ_LR130531.1_2433304..2540433_00071 | 294 | G_tfc13 | typeG | accessory | 2.400e-118 | 387.400 | 0.955 | 0.956 |
| NZ_LR130531.1_2433304..2540433_00072 | 501 | G_tfc14 | typeG | accessory | 1.200e-184 | 607.800 | 0.996 | 0.992 |
| NZ_LR130531.1_2433304..2540433_00073 | 147 | G_tfc15 | typeG | accessory | 4.000e-60 | 194.800 | 0.957 | 0.925 |
| NZ_LR130531.1_2433304..2540433_00074 | 980 | virb4 | typeG | mandatory | 1.800e-90 | 297.400 | 0.807 | 0.899 |
| NZ_LR130531.1_2433304..2540433_00081 | 143 | G_tfc24 | typeG | accessory | 3.000e-51 | 165.900 | 0.914 | 0.895 |
| NZ_LR130531.1_2433304..2540433_00082 | 312 | G_tfc23 | typeG | accessory | 1.600e-133 | 437.800 | 0.976 | 0.990 |
| NZ_LR130531.1_2433304..2540433_00083 | 460 | G_tfc22 | typeG | accessory | 4.000e-180 | 592.200 | 0.987 | 0.974 |
| NZ_LR130531.1_2433304..2540433_00084 | 105 | G_tfc18 | typeG | accessory | 5.800e-20 | 64.200 | 0.912 | 0.876 |
| NZ_LR130531.1_2433304..2540433_00085 | 511 | G_tfc19 | typeG | accessory | 1.700e-222 | 732.300 | 0.990 | 0.982 |
| NZ_LR130531.1_2433304..2540433_00121 | 639 | MOBH | typeG | mandatory | 4.800e-44 | 143.300 | 0.868 | 0.268 |
| NZ_LR130535.1_5115719..5222850_00028 | 374 | G_tfc2 | typeG | accessory | 1.600e-71 | 232.900 | 0.782 | 0.631 |
| NZ_LR130535.1_5115719..5222850_00059 | 251 | G_tfc3 | typeG | accessory | 9.100e-96 | 312.400 | 0.988 | 0.996 |
| NZ_LR130535.1_5115719..5222850_00061 | 166 | G_tfc5 | typeG | accessory | 6.300e-57 | 185.000 | 0.971 | 0.988 |
| NZ_LR130535.1_5115719..5222850_00063 | 743 | t4cp2 | typeG | mandatory | 4.700e-31 | 100.700 | 0.955 | 0.318 |
| NZ_LR130535.1_5115719..5222850_00064 | 248 | G_tfc7 | typeG | accessory | 4.300e-103 | 337.100 | 1.000 | 1.000 |
| NZ_LR130535.1_5115719..5222850_00067 | 112 | G_tfc8 | typeG | accessory | 1.500e-40 | 130.600 | 0.931 | 0.938 |
| NZ_LR130535.1_5115719..5222850_00068 | 79 | G_tfc9 | typeG | accessory | 9.400e-31 | 99.000 | 1.000 | 1.000 |
| NZ_LR130535.1_5115719..5222850_00069 | 118 | G_tfc10 | typeG | accessory | 6.500e-42 | 134.800 | 0.858 | 0.873 |
| NZ_LR130535.1_5115719..5222850_00070 | 128 | G_tfc11 | typeG | accessory | 4.900e-50 | 161.400 | 0.923 | 0.938 |
| NZ_LR130535.1_5115719..5222850_00071 | 219 | G_tfc12 | typeG | accessory | 6.600e-92 | 299.500 | 0.950 | 0.968 |
| NZ_LR130535.1_5115719..5222850_00072 | 294 | G_tfc13 | typeG | accessory | 2.400e-118 | 387.400 | 0.955 | 0.956 |
| NZ_LR130535.1_5115719..5222850_00073 | 501 | G_tfc14 | typeG | accessory | 1.200e-184 | 607.800 | 0.996 | 0.992 |
| NZ_LR130535.1_5115719..5222850_00074 | 147 | G_tfc15 | typeG | accessory | 4.000e-60 | 194.800 | 0.957 | 0.925 |
| NZ_LR130535.1_5115719..5222850_00075 | 980 | virb4 | typeG | mandatory | 1.800e-90 | 297.400 | 0.807 | 0.899 |
| NZ_LR130535.1_5115719..5222850_00082 | 143 | G_tfc24 | typeG | accessory | 3.000e-51 | 165.900 | 0.914 | 0.895 |
| NZ_LR130535.1_5115719..5222850_00083 | 312 | G_tfc23 | typeG | accessory | 1.600e-133 | 437.800 | 0.976 | 0.990 |
| NZ_LR130535.1_5115719..5222850_00084 | 460 | G_tfc22 | typeG | accessory | 4.100e-180 | 592.200 | 0.987 | 0.974 |
| NZ_LR130535.1_5115719..5222850_00085 | 105 | G_tfc18 | typeG | accessory | 5.800e-20 | 64.200 | 0.912 | 0.876 |
| NZ_LR130535.1_5115719..5222850_00086 | 511 | G_tfc19 | typeG | accessory | 1.700e-222 | 732.300 | 0.990 | 0.982 |
| NZ_LR130535.1_5115719..5222850_00122 | 639 | MOBH | typeG | mandatory | 4.900e-44 | 143.300 | 0.868 | 0.268 |
| NZ_LR130536.1_5115575..5222706_00028 | 374 | G_tfc2 | typeG | accessory | 1.600e-71 | 232.900 | 0.782 | 0.631 |
| NZ_LR130536.1_5115575..5222706_00059 | 251 | G_tfc3 | typeG | accessory | 9.100e-96 | 312.400 | 0.988 | 0.996 |
| NZ_LR130536.1_5115575..5222706_00061 | 166 | G_tfc5 | typeG | accessory | 6.300e-57 | 185.000 | 0.971 | 0.988 |
| NZ_LR130536.1_5115575..5222706_00063 | 743 | t4cp2 | typeG | mandatory | 4.700e-31 | 100.700 | 0.955 | 0.318 |
| NZ_LR130536.1_5115575..5222706_00064 | 248 | G_tfc7 | typeG | accessory | 4.300e-103 | 337.100 | 1.000 | 1.000 |
| NZ_LR130536.1_5115575..5222706_00067 | 112 | G_tfc8 | typeG | accessory | 1.500e-40 | 130.600 | 0.931 | 0.938 |
| NZ_LR130536.1_5115575..5222706_00068 | 79 | G_tfc9 | typeG | accessory | 9.400e-31 | 99.000 | 1.000 | 1.000 |
| NZ_LR130536.1_5115575..5222706_00069 | 118 | G_tfc10 | typeG | accessory | 6.500e-42 | 134.800 | 0.858 | 0.873 |
| NZ_LR130536.1_5115575..5222706_00070 | 128 | G_tfc11 | typeG | accessory | 4.900e-50 | 161.400 | 0.923 | 0.938 |
| NZ_LR130536.1_5115575..5222706_00071 | 219 | G_tfc12 | typeG | accessory | 6.600e-92 | 299.500 | 0.950 | 0.968 |
| NZ_LR130536.1_5115575..5222706_00072 | 294 | G_tfc13 | typeG | accessory | 2.400e-118 | 387.400 | 0.955 | 0.956 |
| NZ_LR130536.1_5115575..5222706_00073 | 501 | G_tfc14 | typeG | accessory | 1.200e-184 | 607.800 | 0.996 | 0.992 |
| NZ_LR130536.1_5115575..5222706_00074 | 147 | G_tfc15 | typeG | accessory | 4.000e-60 | 194.800 | 0.957 | 0.925 |
| NZ_LR130536.1_5115575..5222706_00075 | 980 | virb4 | typeG | mandatory | 1.800e-90 | 297.400 | 0.807 | 0.899 |
| NZ_LR130536.1_5115575..5222706_00082 | 143 | G_tfc24 | typeG | accessory | 3.000e-51 | 165.900 | 0.914 | 0.895 |
| NZ_LR130536.1_5115575..5222706_00083 | 312 | G_tfc23 | typeG | accessory | 1.600e-133 | 437.800 | 0.976 | 0.990 |
| NZ_LR130536.1_5115575..5222706_00084 | 460 | G_tfc22 | typeG | accessory | 4.100e-180 | 592.200 | 0.987 | 0.974 |
| NZ_LR130536.1_5115575..5222706_00085 | 105 | G_tfc18 | typeG | accessory | 5.800e-20 | 64.200 | 0.912 | 0.876 |
| NZ_LR130536.1_5115575..5222706_00086 | 511 | G_tfc19 | typeG | accessory | 1.700e-222 | 732.300 | 0.990 | 0.982 |
| NZ_LR130536.1_5115575..5222706_00122 | 639 | MOBH | typeG | mandatory | 4.900e-44 | 143.300 | 0.868 | 0.268 |
| NZ_LR130537.1_5115596..5222727_00028 | 374 | G_tfc2 | typeG | accessory | 1.600e-71 | 232.900 | 0.782 | 0.631 |
| NZ_LR130537.1_5115596..5222727_00059 | 251 | G_tfc3 | typeG | accessory | 9.100e-96 | 312.400 | 0.988 | 0.996 |
| NZ_LR130537.1_5115596..5222727_00061 | 166 | G_tfc5 | typeG | accessory | 6.200e-57 | 185.000 | 0.971 | 0.988 |
| NZ_LR130537.1_5115596..5222727_00063 | 743 | t4cp2 | typeG | mandatory | 4.600e-31 | 100.700 | 0.955 | 0.318 |
| NZ_LR130537.1_5115596..5222727_00064 | 248 | G_tfc7 | typeG | accessory | 4.300e-103 | 337.100 | 1.000 | 1.000 |
| NZ_LR130537.1_5115596..5222727_00067 | 112 | G_tfc8 | typeG | accessory | 1.500e-40 | 130.600 | 0.931 | 0.938 |
| NZ_LR130537.1_5115596..5222727_00068 | 79 | G_tfc9 | typeG | accessory | 9.300e-31 | 99.000 | 1.000 | 1.000 |
| NZ_LR130537.1_5115596..5222727_00069 | 118 | G_tfc10 | typeG | accessory | 6.400e-42 | 134.800 | 0.858 | 0.873 |
| NZ_LR130537.1_5115596..5222727_00070 | 128 | G_tfc11 | typeG | accessory | 4.800e-50 | 161.400 | 0.923 | 0.938 |
| NZ_LR130537.1_5115596..5222727_00071 | 219 | G_tfc12 | typeG | accessory | 6.600e-92 | 299.500 | 0.950 | 0.968 |
| NZ_LR130537.1_5115596..5222727_00072 | 294 | G_tfc13 | typeG | accessory | 2.400e-118 | 387.400 | 0.955 | 0.956 |
| NZ_LR130537.1_5115596..5222727_00073 | 501 | G_tfc14 | typeG | accessory | 1.200e-184 | 607.800 | 0.996 | 0.992 |
| NZ_LR130537.1_5115596..5222727_00074 | 147 | G_tfc15 | typeG | accessory | 4.000e-60 | 194.800 | 0.957 | 0.925 |
| NZ_LR130537.1_5115596..5222727_00075 | 980 | virb4 | typeG | mandatory | 1.800e-90 | 297.400 | 0.807 | 0.899 |
| NZ_LR130537.1_5115596..5222727_00082 | 143 | G_tfc24 | typeG | accessory | 3.000e-51 | 165.900 | 0.914 | 0.895 |
| NZ_LR130537.1_5115596..5222727_00083 | 312 | G_tfc23 | typeG | accessory | 1.600e-133 | 437.800 | 0.976 | 0.990 |
| NZ_LR130537.1_5115596..5222727_00084 | 460 | G_tfc22 | typeG | accessory | 4.000e-180 | 592.200 | 0.987 | 0.974 |
| NZ_LR130537.1_5115596..5222727_00085 | 105 | G_tfc18 | typeG | accessory | 5.800e-20 | 64.200 | 0.912 | 0.876 |
| NZ_LR130537.1_5115596..5222727_00086 | 511 | G_tfc19 | typeG | accessory | 1.700e-222 | 732.300 | 0.990 | 0.982 |
| NZ_LR130537.1_5115596..5222727_00121 | 639 | MOBH | typeG | mandatory | 4.800e-44 | 143.300 | 0.868 | 0.268 |
| NZ_LR134300.1_3471634..3610133_00105 | 251 | G_tfc3 | typeG | accessory | 9.300e-97 | 315.900 | 0.988 | 0.996 |
| NZ_LR134300.1_3471634..3610133_00107 | 166 | G_tfc5 | typeG | accessory | 3.300e-57 | 186.100 | 0.971 | 0.994 |
| NZ_LR134300.1_3471634..3610133_00109 | 743 | t4cp2 | typeG | mandatory | 5.500e-31 | 100.700 | 0.955 | 0.318 |
| NZ_LR134300.1_3471634..3610133_00110 | 248 | G_tfc7 | typeG | accessory | 1.000e-103 | 339.400 | 1.000 | 1.000 |
| NZ_LR134300.1_3471634..3610133_00114 | 112 | G_tfc8 | typeG | accessory | 8.300e-41 | 131.700 | 0.922 | 0.929 |
| NZ_LR134300.1_3471634..3610133_00115 | 79 | G_tfc9 | typeG | accessory | 1.100e-30 | 99.000 | 1.000 | 1.000 |
| NZ_LR134300.1_3471634..3610133_00116 | 118 | G_tfc10 | typeG | accessory | 3.800e-44 | 142.200 | 0.875 | 0.890 |
| NZ_LR134300.1_3471634..3610133_00117 | 128 | G_tfc11 | typeG | accessory | 1.100e-49 | 160.500 | 0.923 | 0.938 |
| NZ_LR134300.1_3471634..3610133_00118 | 219 | G_tfc12 | typeG | accessory | 8.200e-91 | 296.200 | 0.941 | 0.959 |
| NZ_LR134300.1_3471634..3610133_00119 | 294 | G_tfc13 | typeG | accessory | 9.600e-119 | 389.000 | 0.944 | 0.959 |
| NZ_LR134300.1_3471634..3610133_00120 | 501 | G_tfc14 | typeG | accessory | 1.800e-184 | 607.400 | 0.996 | 0.992 |
| NZ_LR134300.1_3471634..3610133_00121 | 147 | G_tfc15 | typeG | accessory | 9.200e-60 | 193.900 | 0.957 | 0.925 |
| NZ_LR134300.1_3471634..3610133_00122 | 980 | virb4 | typeG | mandatory | 2.800e-89 | 293.700 | 0.807 | 0.899 |
| NZ_LR134300.1_3471634..3610133_00129 | 143 | G_tfc24 | typeG | accessory | 1.100e-50 | 164.300 | 0.914 | 0.895 |
| NZ_LR134300.1_3471634..3610133_00130 | 312 | G_tfc23 | typeG | accessory | 4.400e-134 | 439.900 | 0.973 | 0.987 |
| NZ_LR134300.1_3471634..3610133_00131 | 460 | G_tfc22 | typeG | accessory | 1.100e-179 | 591.000 | 0.987 | 0.974 |
| NZ_LR134300.1_3471634..3610133_00132 | 115 | G_tfc18 | typeG | accessory | 1.200e-28 | 92.400 | 0.912 | 0.887 |
| NZ_LR134300.1_3471634..3610133_00133 | 511 | G_tfc19 | typeG | accessory | 1.500e-222 | 732.700 | 0.990 | 0.982 |
| NZ_LR134300.1_3471634..3610133_00145 | 639 | MOBH | typeG | mandatory | 3.900e-44 | 143.900 | 0.873 | 0.271 |
| NZ_LR134308.1_718512..821839_00002 | 639 | MOBH | typeG | mandatory | 4.800e-44 | 143.400 | 0.868 | 0.268 |
| NZ_LR134308.1_718512..821839_00009 | 504 | G_tfc19 | typeG | accessory | 1.600e-222 | 732.500 | 0.984 | 0.990 |
| NZ_LR134308.1_718512..821839_00010 | 115 | G_tfc18 | typeG | accessory | 4.400e-29 | 93.700 | 0.912 | 0.887 |
| NZ_LR134308.1_718512..821839_00011 | 460 | G_tfc22 | typeG | accessory | 5.600e-180 | 591.800 | 0.987 | 0.974 |
| NZ_LR134308.1_718512..821839_00012 | 312 | G_tfc23 | typeG | accessory | 3.800e-134 | 440.000 | 0.973 | 0.987 |
| NZ_LR134308.1_718512..821839_00017 | 980 | virb4 | typeG | mandatory | 4.600e-90 | 296.100 | 0.806 | 0.898 |
| NZ_LR134308.1_718512..821839_00018 | 147 | G_tfc15 | typeG | accessory | 4.600e-60 | 194.700 | 0.957 | 0.925 |
| NZ_LR134308.1_718512..821839_00019 | 501 | G_tfc14 | typeG | accessory | 1.200e-184 | 607.800 | 0.996 | 0.992 |
| NZ_LR134308.1_718512..821839_00020 | 294 | G_tfc13 | typeG | accessory | 2.000e-118 | 387.800 | 0.955 | 0.956 |
| NZ_LR134308.1_718512..821839_00021 | 219 | G_tfc12 | typeG | accessory | 9.000e-92 | 299.200 | 0.950 | 0.968 |
| NZ_LR134308.1_718512..821839_00022 | 128 | G_tfc11 | typeG | accessory | 5.800e-50 | 161.300 | 0.923 | 0.938 |
| NZ_LR134308.1_718512..821839_00023 | 118 | G_tfc10 | typeG | accessory | 8.100e-44 | 141.000 | 0.875 | 0.890 |
| NZ_LR134308.1_718512..821839_00024 | 79 | G_tfc9 | typeG | accessory | 1.300e-30 | 98.700 | 1.000 | 1.000 |
| NZ_LR134308.1_718512..821839_00025 | 112 | G_tfc8 | typeG | accessory | 1.600e-40 | 130.600 | 0.914 | 0.920 |
| NZ_LR134308.1_718512..821839_00039 | 248 | G_tfc7 | typeG | accessory | 1.900e-103 | 338.400 | 1.000 | 1.000 |
| NZ_LR134308.1_718512..821839_00040 | 743 | t4cp2 | typeG | mandatory | 4.700e-31 | 100.800 | 0.955 | 0.318 |
| NZ_LR134308.1_718512..821839_00042 | 166 | G_tfc5 | typeG | accessory | 4.100e-57 | 185.600 | 0.977 | 1.000 |
| NZ_LR134308.1_718512..821839_00044 | 251 | G_tfc3 | typeG | accessory | 5.600e-96 | 313.200 | 0.988 | 0.996 |
| NZ_LR134309.1_5511116..5613952_00073 | 251 | G_tfc3 | typeG | accessory | 4.100e-96 | 313.600 | 0.988 | 0.996 |
| NZ_LR134309.1_5511116..5613952_00075 | 166 | G_tfc5 | typeG | accessory | 2.900e-57 | 186.100 | 0.971 | 0.994 |
| NZ_LR134309.1_5511116..5613952_00077 | 743 | t4cp2 | typeG | mandatory | 4.900e-31 | 100.700 | 0.955 | 0.318 |
| NZ_LR134309.1_5511116..5613952_00078 | 249 | G_tfc7 | typeG | accessory | 1.100e-103 | 339.100 | 1.000 | 0.996 |
| NZ_LR134309.1_5511116..5613952_00103 | 112 | G_tfc8 | typeG | accessory | 7.600e-41 | 131.700 | 0.914 | 0.920 |
| NZ_LR134309.1_5511116..5613952_00104 | 79 | G_tfc9 | typeG | accessory | 1.400e-30 | 98.500 | 1.000 | 1.000 |
| NZ_LR134309.1_5511116..5613952_00105 | 118 | G_tfc10 | typeG | accessory | 7.900e-44 | 141.000 | 0.875 | 0.890 |
| NZ_LR134309.1_5511116..5613952_00106 | 128 | G_tfc11 | typeG | accessory | 5.600e-50 | 161.300 | 0.923 | 0.938 |
| NZ_LR134309.1_5511116..5613952_00107 | 219 | G_tfc12 | typeG | accessory | 8.800e-92 | 299.200 | 0.950 | 0.968 |
| NZ_LR134309.1_5511116..5613952_00108 | 294 | G_tfc13 | typeG | accessory | 4.300e-118 | 386.700 | 0.955 | 0.956 |
| NZ_LR134309.1_5511116..5613952_00109 | 501 | G_tfc14 | typeG | accessory | 1.100e-183 | 604.600 | 0.996 | 0.992 |
| NZ_LR134309.1_5511116..5613952_00110 | 147 | G_tfc15 | typeG | accessory | 4.500e-60 | 194.700 | 0.957 | 0.925 |
| NZ_LR134309.1_5511116..5613952_00111 | 980 | virb4 | typeG | mandatory | 1.300e-90 | 297.900 | 0.806 | 0.898 |
| NZ_LR134309.1_5511116..5613952_00116 | 143 | G_tfc24 | typeG | accessory | 1.400e-49 | 160.600 | 0.907 | 0.888 |
| NZ_LR134309.1_5511116..5613952_00117 | 312 | G_tfc23 | typeG | accessory | 3.700e-134 | 440.000 | 0.973 | 0.987 |
| NZ_LR134309.1_5511116..5613952_00118 | 460 | G_tfc22 | typeG | accessory | 5.500e-180 | 591.800 | 0.987 | 0.974 |
| NZ_LR134309.1_5511116..5613952_00119 | 115 | G_tfc18 | typeG | accessory | 4.300e-29 | 93.700 | 0.912 | 0.887 |
| NZ_LR134309.1_5511116..5613952_00120 | 504 | G_tfc19 | typeG | accessory | 1.600e-222 | 732.500 | 0.984 | 0.990 |
| NZ_LR134309.1_5511116..5613952_00127 | 639 | MOBH | typeG | mandatory | 4.600e-44 | 143.400 | 0.868 | 0.268 |
| NZ_LR590473.1_5871185..5974561_00074 | 251 | G_tfc3 | typeG | accessory | 4.100e-96 | 313.600 | 0.988 | 0.996 |
| NZ_LR590473.1_5871185..5974561_00076 | 166 | G_tfc5 | typeG | accessory | 3.000e-57 | 186.100 | 0.971 | 0.994 |
| NZ_LR590473.1_5871185..5974561_00078 | 689 | t4cp2 | typeG | mandatory | 1.500e-30 | 99.200 | 0.919 | 0.332 |
| NZ_LR590473.1_5871185..5974561_00080 | 249 | G_tfc7 | typeG | accessory | 1.100e-103 | 339.100 | 1.000 | 0.996 |
| NZ_LR590473.1_5871185..5974561_00086 | 112 | G_tfc8 | typeG | accessory | 1.600e-40 | 130.600 | 0.914 | 0.920 |
| NZ_LR590473.1_5871185..5974561_00087 | 79 | G_tfc9 | typeG | accessory | 1.300e-30 | 98.700 | 1.000 | 1.000 |
| NZ_LR590473.1_5871185..5974561_00088 | 118 | G_tfc10 | typeG | accessory | 8.000e-44 | 141.000 | 0.875 | 0.890 |
| NZ_LR590473.1_5871185..5974561_00089 | 128 | G_tfc11 | typeG | accessory | 5.700e-50 | 161.300 | 0.923 | 0.938 |
| NZ_LR590473.1_5871185..5974561_00090 | 219 | G_tfc12 | typeG | accessory | 8.900e-92 | 299.200 | 0.950 | 0.968 |
| NZ_LR590473.1_5871185..5974561_00091 | 294 | G_tfc13 | typeG | accessory | 1.900e-118 | 387.800 | 0.955 | 0.956 |
| NZ_LR590473.1_5871185..5974561_00092 | 501 | G_tfc14 | typeG | accessory | 1.200e-184 | 607.800 | 0.996 | 0.992 |
| NZ_LR590473.1_5871185..5974561_00093 | 115 | G_tfc15 | typeG | accessory | 1.300e-51 | 167.300 | 0.804 | 1.000 |
| NZ_LR590473.1_5871185..5974561_00095 | 637 | virb4 | typeG | mandatory | 3.900e-78 | 256.700 | 0.540 | 0.903 |
| NZ_LR590473.1_5871185..5974561_00100 | 143 | G_tfc24 | typeG | accessory | 9.600e-51 | 164.400 | 0.907 | 0.888 |
| NZ_LR590473.1_5871185..5974561_00101 | 265 | G_tfc23 | typeG | accessory | 2.300e-109 | 358.500 | 0.819 | 0.977 |
| NZ_LR590473.1_5871185..5974561_00102 | 460 | G_tfc22 | typeG | accessory | 7.600e-180 | 591.300 | 0.987 | 0.974 |
| NZ_LR590473.1_5871185..5974561_00103 | 115 | G_tfc18 | typeG | accessory | 3.200e-29 | 94.100 | 0.912 | 0.887 |
| NZ_LR590473.1_5871185..5974561_00104 | 510 | G_tfc19 | typeG | accessory | 1.600e-221 | 729.200 | 0.992 | 0.986 |
| NZ_LR590473.1_5871185..5974561_00129 | 551 | MOBH | typeG | mandatory | 2.700e-21 | 69.100 | 0.598 | 0.207 |
| NZ_LR590474.1_4689973..4788263_00085 | 251 | G_tfc3 | typeG | accessory | 8.700e-96 | 312.500 | 0.967 | 0.976 |
| NZ_LR590474.1_4689973..4788263_00087 | 166 | G_tfc5 | typeG | accessory | 3.700e-57 | 185.700 | 0.971 | 0.994 |
| NZ_LR590474.1_4689973..4788263_00089 | 743 | t4cp2 | typeG | mandatory | 4.700e-31 | 100.700 | 0.955 | 0.318 |
| NZ_LR590474.1_4689973..4788263_00090 | 248 | G_tfc7 | typeG | accessory | 4.700e-103 | 337.000 | 1.000 | 1.000 |
| NZ_LR590474.1_4689973..4788263_00095 | 112 | G_tfc8 | typeG | accessory | 2.000e-41 | 133.500 | 0.931 | 0.938 |
| NZ_LR590474.1_4689973..4788263_00096 | 79 | G_tfc9 | typeG | accessory | 1.400e-30 | 98.500 | 1.000 | 1.000 |
| NZ_LR590474.1_4689973..4788263_00097 | 118 | G_tfc10 | typeG | accessory | 7.600e-44 | 141.000 | 0.875 | 0.890 |
| NZ_LR590474.1_4689973..4788263_00098 | 128 | G_tfc11 | typeG | accessory | 5.500e-50 | 161.300 | 0.923 | 0.938 |
| NZ_LR590474.1_4689973..4788263_00099 | 219 | G_tfc12 | typeG | accessory | 8.500e-92 | 299.200 | 0.950 | 0.968 |
| NZ_LR590474.1_4689973..4788263_00100 | 294 | G_tfc13 | typeG | accessory | 4.200e-118 | 386.700 | 0.955 | 0.956 |
| NZ_LR590474.1_4689973..4788263_00101 | 501 | G_tfc14 | typeG | accessory | 1.100e-183 | 604.600 | 0.996 | 0.992 |
| NZ_LR590474.1_4689973..4788263_00102 | 147 | G_tfc15 | typeG | accessory | 4.300e-60 | 194.700 | 0.957 | 0.925 |
| NZ_LR590474.1_4689973..4788263_00103 | 980 | virb4 | typeG | mandatory | 1.300e-90 | 297.900 | 0.806 | 0.898 |
| NZ_LR590474.1_4689973..4788263_00109 | 143 | G_tfc24 | typeG | accessory | 9.200e-51 | 164.400 | 0.907 | 0.888 |
| NZ_LR590474.1_4689973..4788263_00110 | 312 | G_tfc23 | typeG | accessory | 9.200e-134 | 438.600 | 0.973 | 0.987 |
| NZ_LR590474.1_4689973..4788263_00111 | 462 | G_tfc22 | typeG | accessory | 5.400e-180 | 591.800 | 0.987 | 0.970 |
| NZ_LR590474.1_4689973..4788263_00112 | 115 | G_tfc18 | typeG | accessory | 3.900e-29 | 93.800 | 0.912 | 0.887 |
| NZ_LR590474.1_4689973..4788263_00113 | 511 | G_tfc19 | typeG | accessory | 7.000e-223 | 733.600 | 0.990 | 0.982 |
| NZ_LR590474.1_4689973..4788263_00123 | 639 | MOBH | typeG | mandatory | 4.700e-44 | 143.400 | 0.868 | 0.268 |
| NZ_LS998783.1_5820251..5904145_00035 | 374 | G_tfc2 | typeG | accessory | 1.300e-71 | 232.900 | 0.782 | 0.631 |
| NZ_LS998783.1_5820251..5904145_00066 | 251 | G_tfc3 | typeG | accessory | 6.900e-96 | 312.600 | 0.967 | 0.976 |
| NZ_LS998783.1_5820251..5904145_00068 | 166 | G_tfc5 | typeG | accessory | 2.500e-57 | 186.000 | 0.971 | 0.994 |
| NZ_LS998783.1_5820251..5904145_00070 | 743 | t4cp2 | typeG | mandatory | 3.900e-31 | 100.700 | 0.955 | 0.318 |
| NZ_LS998783.1_5820251..5904145_00071 | 248 | G_tfc7 | typeG | accessory | 9.500e-104 | 339.000 | 1.000 | 1.000 |
| NZ_LS998783.1_5820251..5904145_00077 | 112 | G_tfc8 | typeG | accessory | 5.600e-41 | 131.800 | 0.931 | 0.938 |
| NZ_LS998783.1_5820251..5904145_00078 | 79 | G_tfc9 | typeG | accessory | 1.900e-30 | 97.800 | 1.000 | 1.000 |
| NZ_LS998783.1_5820251..5904145_00079 | 118 | G_tfc10 | typeG | accessory | 5.300e-44 | 141.300 | 0.925 | 0.924 |
| NZ_LS998783.1_5820251..5904145_00080 | 128 | G_tfc11 | typeG | accessory | 9.800e-50 | 160.200 | 0.923 | 0.938 |
| NZ_LS998783.1_5820251..5904145_00081 | 219 | G_tfc12 | typeG | accessory | 1.300e-91 | 298.400 | 0.950 | 0.968 |
| NZ_LS998783.1_5820251..5904145_00082 | 298 | G_tfc13 | typeG | accessory | 1.000e-118 | 388.400 | 0.955 | 0.943 |
| NZ_LS998783.1_5820251..5904145_00083 | 485 | G_tfc14 | typeG | accessory | 8.700e-179 | 588.200 | 0.967 | 0.996 |
| NZ_LS998783.1_5820251..5904145_00084 | 147 | G_tfc15 | typeG | accessory | 1.600e-59 | 192.600 | 0.957 | 0.925 |
| NZ_LS998783.1_5820251..5904145_00085 | 983 | virb4 | typeG | mandatory | 1.200e-89 | 294.500 | 0.810 | 0.899 |
| NZ_LS998783.1_5820251..5904145_00091 | 143 | G_tfc24 | typeG | accessory | 3.600e-51 | 165.400 | 0.907 | 0.888 |
| NZ_LS998783.1_5820251..5904145_00092 | 312 | G_tfc23 | typeG | accessory | 2.700e-134 | 440.100 | 0.973 | 0.987 |
| NZ_LS998783.1_5820251..5904145_00093 | 460 | G_tfc22 | typeG | accessory | 5.800e-180 | 591.400 | 0.989 | 0.976 |
| NZ_LS998783.1_5820251..5904145_00094 | 115 | G_tfc18 | typeG | accessory | 3.600e-29 | 93.600 | 0.912 | 0.887 |
| NZ_LS998783.1_5820251..5904145_00095 | 504 | G_tfc19 | typeG | accessory | 2.200e-223 | 735.000 | 0.988 | 0.994 |
| NZ_LS998783.1_5820251..5904145_00102 | 640 | MOBH | typeG | mandatory | 2.700e-44 | 143.900 | 0.868 | 0.267 |
| NZ_LT608330.1_5254562..5362559_00068 | 251 | G_tfc3 | typeG | accessory | 4.700e-95 | 310.000 | 0.967 | 0.976 |
| NZ_LT608330.1_5254562..5362559_00070 | 166 | G_tfc5 | typeG | accessory | 6.900e-56 | 181.500 | 0.977 | 1.000 |
| NZ_LT608330.1_5254562..5362559_00072 | 744 | t4cp2 | typeG | mandatory | 5.300e-31 | 100.400 | 0.955 | 0.319 |
| NZ_LT608330.1_5254562..5362559_00073 | 248 | G_tfc7 | typeG | accessory | 1.100e-103 | 339.000 | 1.000 | 1.000 |
| NZ_LT608330.1_5254562..5362559_00084 | 216 | G_tfc7 | typeG | accessory | 9.200e-96 | 313.000 | 0.871 | 1.000 |
| NZ_LT608330.1_5254562..5362559_00089 | 112 | G_tfc8 | typeG | accessory | 3.800e-41 | 132.500 | 0.922 | 0.929 |
| NZ_LT608330.1_5254562..5362559_00090 | 79 | G_tfc9 | typeG | accessory | 8.800e-31 | 99.000 | 1.000 | 1.000 |
| NZ_LT608330.1_5254562..5362559_00091 | 118 | G_tfc10 | typeG | accessory | 1.200e-44 | 143.600 | 0.883 | 0.890 |
| NZ_LT608330.1_5254562..5362559_00092 | 128 | G_tfc11 | typeG | accessory | 4.700e-50 | 161.400 | 0.923 | 0.938 |
| NZ_LT608330.1_5254562..5362559_00093 | 219 | G_tfc12 | typeG | accessory | 1.400e-91 | 298.400 | 0.950 | 0.968 |
| NZ_LT608330.1_5254562..5362559_00094 | 294 | G_tfc13 | typeG | accessory | 1.100e-118 | 388.500 | 0.955 | 0.956 |
| NZ_LT608330.1_5254562..5362559_00095 | 501 | G_tfc14 | typeG | accessory | 9.200e-185 | 608.000 | 0.996 | 0.992 |
| NZ_LT608330.1_5254562..5362559_00096 | 147 | G_tfc15 | typeG | accessory | 2.000e-59 | 192.500 | 0.957 | 0.925 |
| NZ_LT608330.1_5254562..5362559_00097 | 980 | virb4 | typeG | mandatory | 1.600e-89 | 294.200 | 0.807 | 0.899 |
| NZ_LT608330.1_5254562..5362559_00101 | 312 | G_tfc23 | typeG | accessory | 8.600e-134 | 438.600 | 0.973 | 0.987 |
| NZ_LT608330.1_5254562..5362559_00102 | 460 | G_tfc22 | typeG | accessory | 8.800e-180 | 591.000 | 0.987 | 0.974 |
| NZ_LT608330.1_5254562..5362559_00103 | 113 | G_tfc18 | typeG | accessory | 5.000e-28 | 90.100 | 0.912 | 0.885 |
| NZ_LT608330.1_5254562..5362559_00104 | 503 | G_tfc19 | typeG | accessory | 1.300e-222 | 732.600 | 0.986 | 0.994 |
| NZ_LT608330.1_5254562..5362559_00114 | 639 | MOBH | typeG | mandatory | 1.900e-44 | 144.500 | 0.887 | 0.275 |
| NZ_LT969520.1_5616956..5750608_00083 | 251 | G_tfc3 | typeG | accessory | 5.200e-96 | 313.600 | 0.988 | 0.996 |
| NZ_LT969520.1_5616956..5750608_00085 | 166 | G_tfc5 | typeG | accessory | 3.700e-57 | 186.100 | 0.971 | 0.994 |
| NZ_LT969520.1_5616956..5750608_00087 | 743 | t4cp2 | typeG | mandatory | 6.200e-31 | 100.700 | 0.955 | 0.318 |
| NZ_LT969520.1_5616956..5750608_00088 | 249 | G_tfc7 | typeG | accessory | 1.400e-103 | 339.100 | 1.000 | 0.996 |
| NZ_LT969520.1_5616956..5750608_00102 | 112 | G_tfc8 | typeG | accessory | 2.000e-40 | 130.600 | 0.914 | 0.920 |
| NZ_LT969520.1_5616956..5750608_00103 | 79 | G_tfc9 | typeG | accessory | 1.600e-30 | 98.700 | 1.000 | 1.000 |
| NZ_LT969520.1_5616956..5750608_00104 | 118 | G_tfc10 | typeG | accessory | 1.000e-43 | 141.000 | 0.875 | 0.890 |
| NZ_LT969520.1_5616956..5750608_00105 | 128 | G_tfc11 | typeG | accessory | 7.200e-50 | 161.300 | 0.923 | 0.938 |
| NZ_LT969520.1_5616956..5750608_00106 | 219 | G_tfc12 | typeG | accessory | 1.100e-91 | 299.200 | 0.950 | 0.968 |
| NZ_LT969520.1_5616956..5750608_00107 | 294 | G_tfc13 | typeG | accessory | 2.400e-118 | 387.800 | 0.955 | 0.956 |
| NZ_LT969520.1_5616956..5750608_00108 | 501 | G_tfc14 | typeG | accessory | 1.500e-184 | 607.800 | 0.996 | 0.992 |
| NZ_LT969520.1_5616956..5750608_00109 | 147 | G_tfc15 | typeG | accessory | 5.700e-60 | 194.700 | 0.957 | 0.925 |
| NZ_LT969520.1_5616956..5750608_00110 | 980 | virb4 | typeG | mandatory | 5.700e-90 | 296.100 | 0.806 | 0.898 |
| NZ_LT969520.1_5616956..5750608_00115 | 143 | G_tfc24 | typeG | accessory | 1.200e-50 | 164.400 | 0.907 | 0.888 |
| NZ_LT969520.1_5616956..5750608_00116 | 312 | G_tfc23 | typeG | accessory | 4.700e-134 | 440.000 | 0.973 | 0.987 |
| NZ_LT969520.1_5616956..5750608_00117 | 460 | G_tfc22 | typeG | accessory | 7.000e-180 | 591.800 | 0.987 | 0.974 |
| NZ_LT969520.1_5616956..5750608_00118 | 115 | G_tfc18 | typeG | accessory | 5.400e-29 | 93.700 | 0.912 | 0.887 |
| NZ_LT969520.1_5616956..5750608_00119 | 504 | G_tfc19 | typeG | accessory | 2.000e-222 | 732.500 | 0.984 | 0.990 |
| NZ_LT969520.1_5616956..5750608_00126 | 639 | MOBH | typeG | mandatory | 5.900e-44 | 143.400 | 0.868 | 0.268 |
