# Supplementary material for: ICEs Are the Main Reservoirs of the Ciprofloxacin-Modifying crpP Gene in Pseudomonas aeruginosa
Source: Genes (Basel). 2020 Aug 4;11(8):889. doi: 10.3390/genes11080889 (PMC7463715; doi:10.3390/genes11080889)
Supplement: Supplementary file 1 [file genes-11-00889-s001.zip › Table_S3.docx]

**Table S3**. Average nucleotide identity (ANI) comparison between non-Pseudomonas aeruginosa strains identified in this study and the P. aeruginosa reference strain.

| **Query** | **Reference** | **ANI value** | **Count of bidirectional fragment mappings** | **Total query fragments** |
| --- | --- | --- | --- | --- |
| Pseudomonas fluorescens strain NCTC10783 | Pseudomonas aeruginosa DSM 50071 | 99.2424 | 1980 | 2288 |
| Pseudomonas sp. AK6U | Pseudomonas aeruginosa DSM 50071 | 99.2771 | 1952 | 2303 |
